# Phylogeny of Annelida (Lophotrochozoa): total-evidence analysis of morphology and six genes

**DOI:** 10.1186/1471-2148-9-189

**Published:** 2009-08-06

**Authors:** Jan Zrzavý, Pavel Říha, Lubomír Piálek, Jan Janouškovec

**Affiliations:** 1 Faculty of Science, University of South Bohemia, Branišovská 31, 370 05 České Budĕjovice, Czech Republic; 2 Biology Center, Academy of Sciences, Branišovská 31, 370 05 České Budĕjovice, Czech Republic

## Abstract

**Background:**

Annelida is one of the major protostome phyla, whose deep phylogeny is very poorly understood. Recent molecular phylogenies show that Annelida may include groups once considered separate phyla (Pogonophora, Echiurida, and Sipunculida) and that Clitellata are derived polychaetes. SThe "total-evidence" analyses combining morphological and molecular characters have been published for a few annelid taxa. No attempt has yet been made to analyse simultaneously morphological and molecular information concerning the Annelida as a whole.

**Results:**

Phylogenetic relationships within Annelida were analysed on the basis of 93 morphological characters and sequences of six genes (*18S*, *28S*, and *16S rRNA*, *EF1α*, *H3*, *COI*), altogether, 87 terminals of all annelid "families" and 3,903 informative characters, by Bayesian and maximum-parsimony methods. The analysis of the combined dataset yields the following scheme of relationships: Phyllodocida and Eunicida are monophyletic groups, together probably forming monophyletic Aciculata (incl. Orbiniidae and Parergodrilidae that form a sister group of the Eunicida). The traditional "Scolecida" and "Canalipalpata" are both polyphyletic, forming instead two clades: one including Cirratuliformia and the "sabelloid-spionoid clade" (incl. *Sternaspis*, Sabellidae-Serpulidae, Sabellariidae, Spionida s.str.), the other ("terebelloid-capitelloid clade") including Terebelliformia, Arenicolidae-Maldanidae, and Capitellidae-Echiurida. The Clitellata and "clitellate-like polychaetes" (Aeolosomatidae, Potamodrilidae, *Hrabeiella*) form a monophyletic group. The position of the remaining annelid groups is uncertain – the most problematic taxa are the Opheliidae-Scalibregmatidae clade, the Amphinomida-*Aberranta *clade, *Apistobranchus*, Chaetopteridae, Myzostomida, the Sipunculida-Dinophilidae clade, and the "core Archiannelida" (= Protodrilidae, Nerillidae, Polygordiidae, Saccocirridae).

**Conclusion:**

The combined ("total-evidence") phylogenetic analysis provides a modified view of annelid evolution, with several higher-level taxa, i.e. Phyllodocida, Eunicida, orbinioid-parergodrilid clade (OPC), Cirratuliformia, sabelloid-spionoid clade (SSC), terebelloid-capitelloid clade (TCC), and "Clitellatomorpha". Two unorthodox clades, the "core Archiannelida" and Sipunculida-Dinophilidae, are proposed. Although the deep-level evolutionary relationships of Annelida remain poorly understood, we propose the monophyly of the Aciculata, sister-group relationships between the Eunicida and OPC, between the Cirratuliformia and SSC, and possibly also between the "Clitellatomorpha" and Oweniidae-Pogonophora clades.

## Background

Annelida, the segmented worms (over 16,500 species described), are distributed worldwide from the deepest marine sediments to freshwater and soil habitats. Throughout most of the 20th century they were split into three or four major groups, Polychaeta, Myzostomida, Oligochaeta and Hirudinea. It is now widely recognized that Oligochaeta and Hirudinea form a clade that is referred to as Clitellata (where leeches are only a derived subgroup of oligochaetes [1-3]). Several interstitial groups were classified as the "Archiannelida", another annelid group; however, they are now generally regarded as secondarily simplified, possibly progenetic polychaetes [4,5]. Several more groups have been hypothesized to belong into the Annelida [6], and there is a growing consensus that the Echiurida, Pogonophora (incl. Vestimentifera), and Sipunculida are actually modified annelids [7-9].

A cladistic analysis of Annelida, based on morphological characters, has resulted in a new classification [10,11], with three major clades of the Polychaeta: Scolecida, Aciculata (= Amphinomida + Eunicida + Phyllodocida), and Canalipalpata (= Terebellida + Spionida + Sabellida [incl. Pogonophora]). However, several annelid groups were left outside this classification. They include Clitellata, the freshwater and/or terrestrial "clitellate-like" worms (Parergodrilidae, *Hrabeiella*, and Aphanoneura [= Aeolosomatidae + *Potamodrilus*]), some "archiannelids" (Protodrilida and Polygordiidae, both only tentatively regarded as aberrant canalipalpatans), and Psammodrilidae.

From a molecular perspective, the sequence datasets assembled to date have usually been marked by limited numbers of both taxa and characters. Almost all annelid families are now represented by the nuclear small-subunit ribosomal RNA genes ("*18S*" hereinafter); unfortunately, even *18S *studies using the densest taxon sampling [12-14] were unable to recover a monophyletic Annelida or its major subclades. Even if several genes are concatenated to reconstruct annelid phylogeny in recent papers, none of the morphology-based higher taxa (Polychaeta, Scolecida, Aciculata, Canalipalpata) were recovered [15-17]. Recent papers by Struck *et al*. [7,8] provided the first molecular trees with several resolved higher taxa of the Annelida. They included Aciculata (excl. Amphinomida), Phyllodocida (incl. Orbiniidae), Terebelliformia, Sabellida-Spionida, Cirratuliformia, and Amphinomida.

Synthesis of molecular and morphological data from extant and potentially also extinct taxa remains the strongest test of phylogenetic hypotheses and the best summary of the common signal in the diverse data available for phylogenetics [18]. The "total-evidence" analyses have been published for a few annelid taxa, viz., Clitellata [2], Terebelliformia [19], most Canalipalpata [20], Aphroditiformia [21], and most Aciculata [22]. So far, no attempt has been made to analyse simultaneously morphological and molecular information on the Annelida as a whole.

In this paper we present the first comprehensive analysis of higher-level phylogenetic relationships in Annelida based on combined morphological and molecular (four nuclear, two mitochondrial genes) data. The purpose is to identify stable and ustable nodes of the combined annelid tree, to make up reliable phylogenetic hypothesis on Annelida, and thus test the morphology-based classification.

## Results

### The congruence of data partitions

The combined data matrix included 87 terminals and 3,903 cladistically informative characters (93 morphology [= MOR]; 630 cytochrome *c *oxidase subunit I [= *COI*]; 604 elongation factor-1α [= *EF1α*]; 132 histone *H3*; 763 *18S *ribosomal RNA; 274 *16S *ribosomal RNA; 1,407 *28S *ribosomal RNA). Out of 339,561 data matrix cells (= character states), 6% were polymorphic and 30% were missing (unknown or inapplicable). Distribution of the ambiguous character states is highly uneven, as four terminals (Histriobdellidae, Sphaerodoridae, *Aberranta*, *Parapodrilus*) are represented by two data partitions only (MOR + 18S).

**Figure 1:**
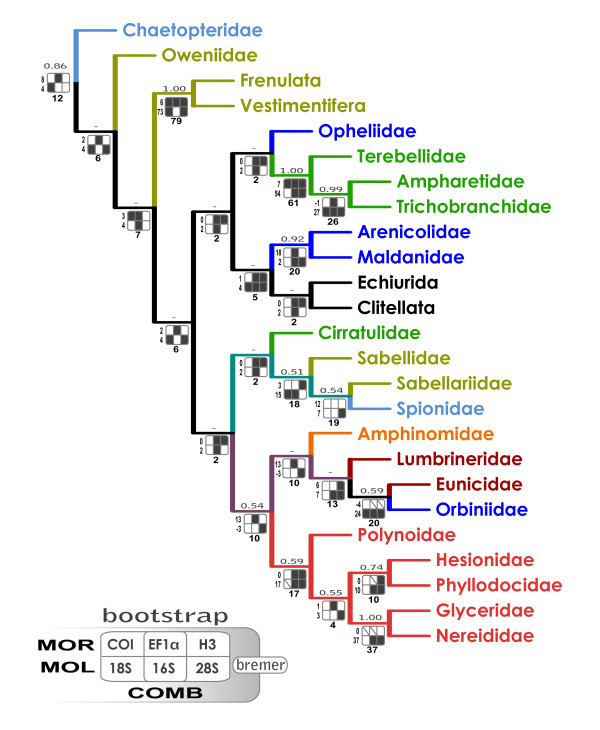
**Evaluation of the performance of data partitions in the combined maximum-parsimony tree**. The analysis was limited to the 28 terminals, represented by all seven data partitions [see Additional File 4]. Bootstrap values are above the branches (only higher than 50%); partitioned Bremer support (PBS) values under the branches: COMB (total Bremer support of all partitions), MOR (PBS of morphology), MOL (total Bremer support of the six molecular partitions). The support grids: black cells: PBS > 0; white cells: PBS < 0; diagonal: PBS = 0. For color code see Figure 2.

Evaluation of the relative quality of data partitions and their performance in the combined maximum-parsimony analysis indicated that the MOR dataset was highly influential in the simultaneous analyses of all data partitions (Figure 1). The partitioned Bremer support (PBS) values in the combined dataset, limited to the 28 composite terminals that were represented by all seven data partitions (one tree, length 18,195, CI 0.35, RI 0.24; Figure 1), revealed that despite the significant incongruence of the morphological and molecular data partitions, the former contributed positively to Bremer support values of (= supported) 15 clades (60%) and is negative for (= contradicted) two clades (8%) only: one within Terebelliformia, the other concerning position of the Orbiniidae within Aciculata. The molecular partitions analysed together are in conflict with two clades as well, both concerning the placement of the Amphinomidae as a sister group of the Eunicida. The molecular partitions are, however, by no means homogeneous. The PBS analysis showed that four data partitions contributed positively to the combined-tree topology (MOR: ΣPBS = 100; *28S*: 322; *EF1a*: 232; *16S*: 100), while the other three molecular partitions (*COI*, *H3*, *18S*) are in conflict with the combined tree.

### Bayesian (BI) tree

**Figure 2:**
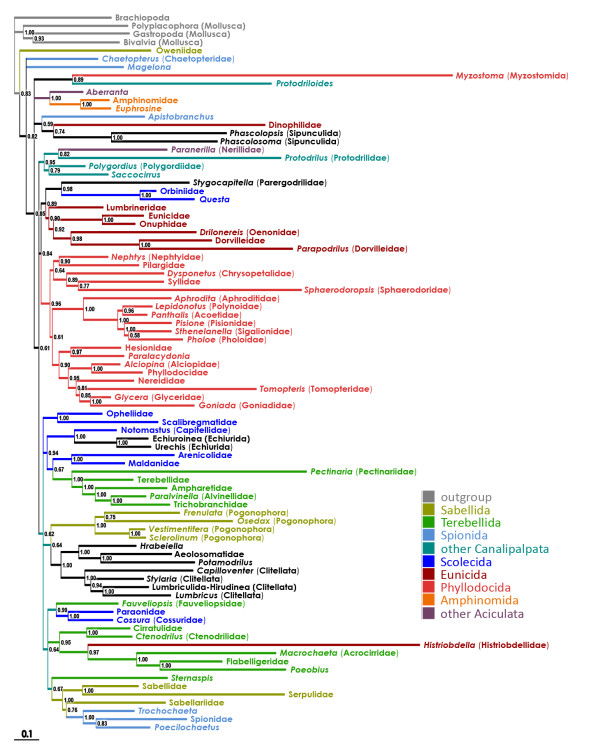
**Bayesian tree for the combined dataset (morphology + six molecular partitions)**. Posterior probabilities are shown on the branches. Terminals with just the higher-taxa names (e.g. "Flabelligeridae") indicate that the sequences from different species of that taxon were concatenated [see Additional File 4].

In the combined BI tree, the basal relationships were not fully resolved. However, there were several well-supported (pp > 0.90) clades (Figure 2): (i) the "sabelloid-spionoid clade" ("SSC" hereinafter; incl. Sabellida s.str. and Spionida s.str.); (ii) Cirratuliformia (incl. Histriobdellidae); (iii) Fauveliopsidae-Paraonidae-Cossuridae; (iv) Clitellata-Aphanoneura-*Hrabeiella *("Clitellatomorpha" hereinafter); (v) Pogonophora; (vi) Opheliidae-Scalibregmatidae; (vii) the "terebelloid-capitelloid clade" or "TCC" (incl. Terebelliformia, Maldanidae-Arenicolidae, and Capitellidae-Echiurida); (viii) Phyllodocida; (ix) Eunicida; (x) the "orbinioid-parergodrilid clade" or "OPC"; (xi) *Aberranta*-Amphinomida; and (xii) the "core Archiannelida" (Polygordiidae-Saccocirridae and Protodrilidae-Nerillidae). The traditional Aciculata and Canalipalpata roughly corresponded to the two major superclades (both, however, with rather low posterior probabilities and very short basal branches). The "Aciculata" included Phyllodocida, Eunicida, and OPC. The "Canalipalpata" included SSC, *Sternaspis*, TCC, Cirratuliformia, Fauveliopsidae-Paraonidae-Cossuridae, Pogonophora, and also "Clitellatomorpha" and Opheliidae-Scalibregmatidae. There were, in addition, several groups outside the two major sister superclades: Oweniidae, Chaetopteridae, *Magelona*, Myzostomida-*Protodriloides*, *Aberranta*-Amphinomida, *Apistobranchus*-Dinophilidae-Sipunculida, and the "core Archiannelida".

### Maximum-parsimony (MP) trees

**Figure 3:**
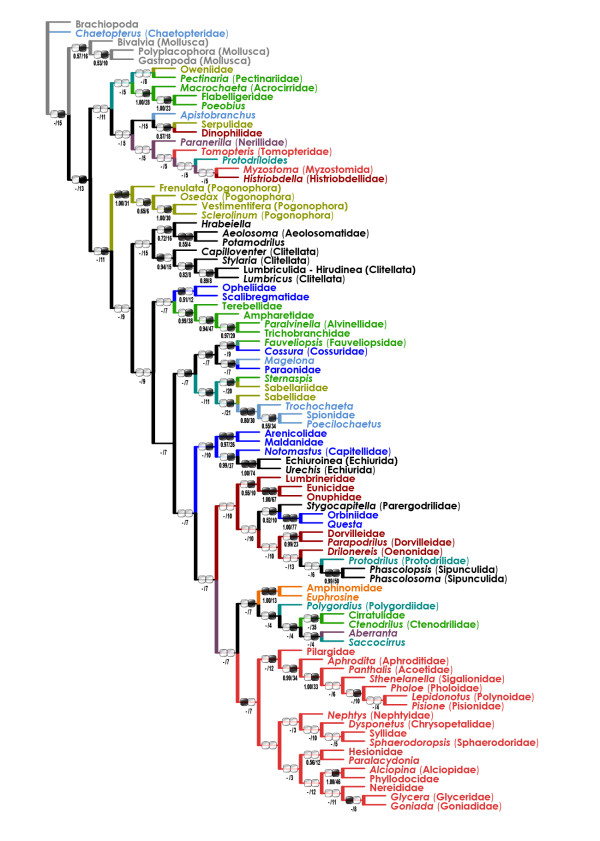
**Unweighted maximum-parsimony (uMP) tree for the combined dataset (morphology + six molecular partitions)**. Bootstrap/Bremer support values are below the branches. Black/white ellipse hashmarks indicate presence/absence of the clade in purely morphological (left) and molecular (right) trees (uMP), respectively (functionally monotypic taxa are not hashmarked). For color code see Figure 2.

In the unweighted (uMP) analysis (one tree, length 42,492, CI 0.20, RI 0.25), monophyletic Aciculata were nested within paraphyletic "Canalipalpata" (Figure 3). A few taxa seemed to be misplaced due to long-branch artifacts (e.g. Serpulidae far from the Sabellidae, Pectinariidae outside the Terebelliformia, and Tomopteridae outside the Phyllodocida); moreover, there was an obviously artifactual "basal" clade including "archiannelids", myzostomids, and several long-branch aciculatans and canalipalpatans.

**Figure 4:**
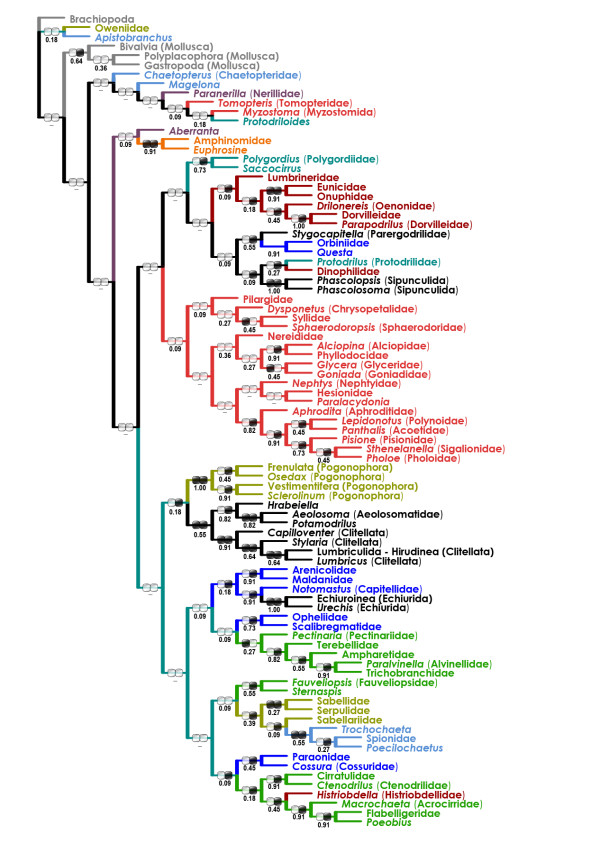
**Weighted ("slow-fast") maximum-parsimony (wMP) tree for the combined dataset (morphology + six molecular partitions)**. Presence of a clade in the 11 trees derived from the reduced datasets (see text) is shown below the branches (in %). Black/white ellipse hashmarks indicate presence/absence of the clade in purely morphological (left) and molecular (right) trees (both wMP), respectively.

The weighted (wMP, "slow-fast") tree (one tree, weighted length 129,822, CI 0.25, RI 0.27; Figure 4) was nearly identical with the BI tree (both included two major sister superclades, one mostly aciculatan, the other mostly canalipalpatan). They differed predominantly only in position of the Opheliidae-Scalibregmatidae clade, "core archiannelids", Dinophilidae-Sipunculida clade, *Apistobranchus*, and Tomopteridae.

### Taxon-exclusion tests

The preliminary results suggested that ingroup and outgroup taxon selection affected internal relationships. To highlight topological problems in placement of the most "problematic" taxa (Myzostomida, Opheliidae, Scalibregmatidae, Fauveliopsidae, Serpulidae, *Magelona*, Oweniidae, Chaetopteridae, Pectinariidae, *Apistobranchus*, Polygordiidae, Saccocirridae, Protodrilidae, *Protodriloides*, Nerillidae, Histriobdellidae, Dinophilidae, Sipunculida, Hesionidae, Paralacydoniidae, Nepthyidae, Pilargidae, Tomopteridae, *Aberranta*, Amphinomida; see Methods for selection criteria), several experimental analyses were performed.

**Figure 5:**
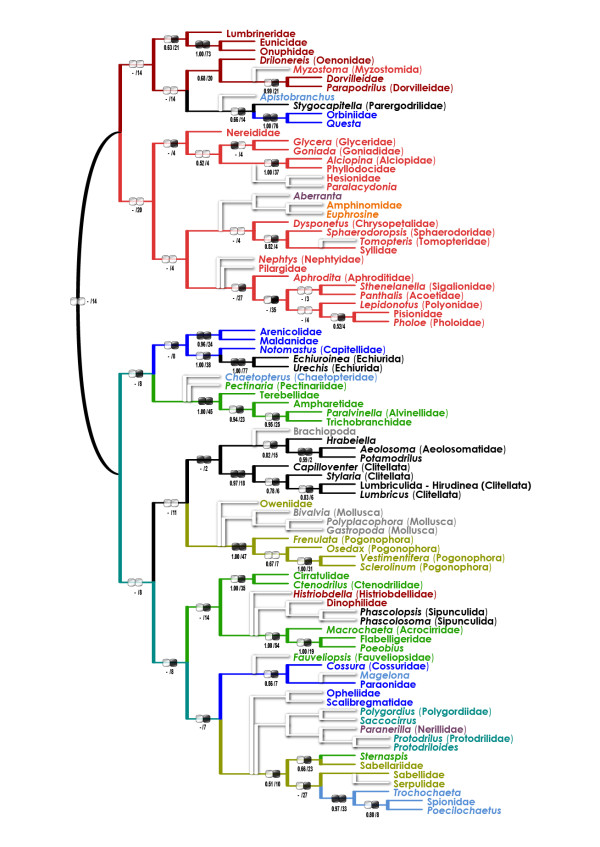
**Results of the taxon-exclusion maximum-parsimony analyses (morphology + six molecular partitions)**. The trees are unrooted (position of possible outgroups, Brachiopoda and Mollusca, indicated). The "backbone tree" is colored, the "problematic taxa" appended in one-by-one manner are in white. Unweighted maximum parsimony; bootstrap values/Bremer support values of the "backbone tree" clades are below the branches. Black/white ellipse hashmarks indicate presence/absence of the clade in purely morphological (left) and molecular (right) "backbone taxa" (uMP), respectively.

**Figure 6:**
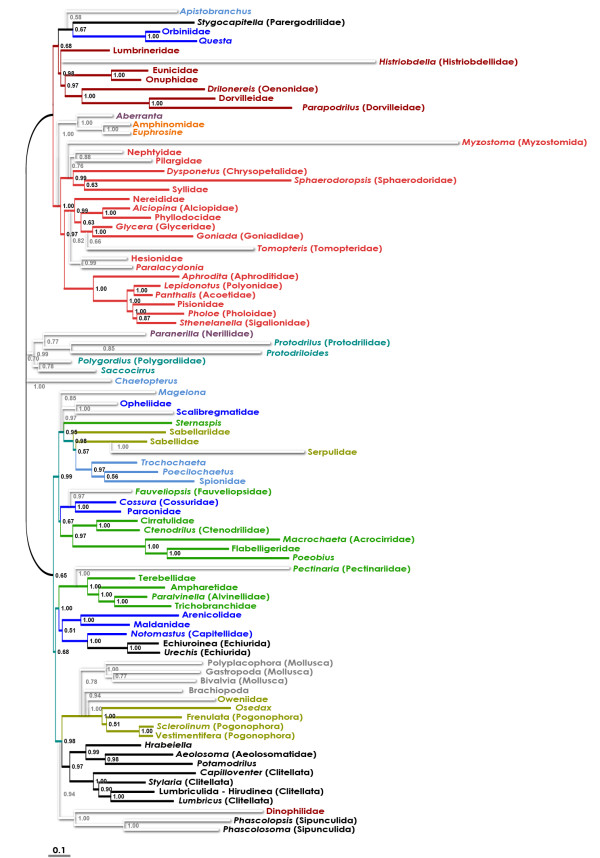
**Results of the taxon-exclusion Bayesian analyses (morphology + six molecular partitions)**. The trees are unrooted (position of possible outgroups, Brachiopoda and Mollusca, indicated). The "backbone tree" is colored, the "problematic taxa" appended in one-by-one manner are in white. Posterior probabilities are shown on the branches.

In the taxon-exclusion tests, after removing the "problematic" species/clades, the unrooted "backbone trees" were almost identical irrespective of the used methods (uMP and BI). Both included either monophyletic or paraphyletic (which naturally cannot be distinguished in an unrooted tree) "Aciculata" and "Canalipalpata", and differed only in the specific position of the Pogonophora-"Clitellatomorpha" clade within the "Canalipalpata" (Figure 5, 6). The important "problematica" grouped consistently as follows: Oweniidae as a sister group of the Pogonophora; Fauveliopsidae, *Magelona*, and Opheliidae-Scalibregmatidae within the Cirratuliformia-SSC clade; *Apistobranchus *as a sister group of OPC; and *Aberranta*-Amphinomida next to (or within) the Phyllodocida. The other "problematic species" were placed at more or less conflicting positions in BI and uMP tests (see below). The ougroups tended to be placed within the "Canalipalpata", close to the Pogonophora and/or "Clitellatomorpha".

## Discussion

The present analysis points out instability of some basal nodes within the annelid tree. However, most analyses, irrespective of the used methods and parameter sets, converge to a highly compatible set of actually (or, in the unrooted trees, potentially) monophyletic taxa. They are (i) Eunicida-OPC; (ii) Phyllodocida; (iii) SSC; (iv) Cirratuliformia; (v) TCC; (vi) "Clitellatomorpha"; and (vii) Pogonophora (Figure 2, 4, 5 &6). Several morphology-based taxa like "Scolecida" (= Orbiniidae-*Questa*, Opheliidae-Scalibregmatidae, Arenicolidae-Maldanidae, Capitellidae), "Terebellida" (= Terebelliformia and Cirratuliformia), and "Spionida" (= Spionida s.str., *Magelona*, Chaetopteridae, *Apistobranchus*) [10,11] are definitely polyphyletic.

The most problematic issue is the tree root position. The all-taxa analyses (that included mollusc and brachiopod outgroups) supported that the root is situated between chaetopterids, magelonids, and/or oweniids and the rest of the Annelida (Figure 2, 3 &4). The experimental analyses (Figures 5, 6) suggested that the non-annelid outgroups were placed next to the Oweniidae, Pogonophora, and/or "Clitellatomorpha".

### Phyllodocida, Eunicida, and the orbinioid-parergodrilid clade (OPC)

The Phyllodocida (excl. Myzostomida) is consistently a monophyletic group in all analyses, except for "basal" placement of the long-branch Tomopteridae in some MP trees. In all analyses, there is a strongly supported clade including monophyletic Eunicida (less dinophilids and sometimes also histriobdellids) and OPC, corroborated by presence of the lateral/dorsal cirrus organs and larval akrotrochs. However, the position of the lumbrinerids could be more basal, as they appeared as a sister group of the whole Eunicida-Phyllodocida in some purely molecular trees [7,8,23]. The placement of the orbiniids among the aciculatans provides a strong phylogenetic support for the hypothesis that their aciculae are homologous with those of the Euncida and Phyllodocida [24]. The sister-group relationship between Parergodrilidae and Orbiniidae-*Questa *is strongly supported by all analyses [8,24]; both groups share gonoducts with a distal glandular part [25]. No closer orbiniid-spionidan relationships [26] have been recovered by the present analyses. Also *Apistobranchus *consistently grouped with OPC in the taxon-exclusion analyses. It is, therefore, possible that also the *Apistobranchus *acicula-like chaetae [24] might be actually true aciculae.

### "Terebelloid-capitelloid clade" (TCC)

One of the most stable clade covers Terebelliformia, Capitellidae-Echiurida and Arenicolidae-Maldanidae. This clade is weakly supported morphologically, by the specific chaetal arrangement and by the presence of a gular membrane. The placement of the terebelliformians close to a generally palpless group provides a phylogenetic support for the hypothesis that terebelliformian "buccal tentacles" are not homologous with the true polychaete palps [27]. Although classified as a separate phylum until recently, there is a growing consensus that Echiurida is a subgroup of the Annelida [28], as they exhibit segmentation traits during ontogeny. The explicit hypotheses about their specific position were published only by molecular phylogeneticists who discovered close relationships of the Echiurida to the Capitellidae [7-9,13-16]. Also the Opheliidae and Scalibregmatidae group either within TCC (present MP, [7,8,15]), or as an isolated clade (present BI). However, in absence of all other problematic annelids, the Opheliidae-Scalibregmatidae clade nests close to the SSC.

### "Sabelloid-spionoid clade" (SSC) and Cirratuliformia

In all trees, Sabellidae, Spionida s.str. (= Spionidae + *Trochochaeta *+ *Poecilochaetus*), and Sabellariidae form a clade [8]. The chaetal arrangement in Sabellariidae has recently been dismissed as homologous to the sabellid-serpulid chaetal inversion pattern, and therefore provides no support for a sister-group relationship of sabellids and sabellariids with exclusion of the Spionida s.str. [29]. Fauveliopsidae, *Sternaspis*, Cossuridae and Paraonidae are consistently placed next to the SSC and/or Cirratuliformia [[7,17,20,30]; cf. [8]]. The monophyly of the "Terebellida" (joining Cirratuliformia and Terebelliformia [10,11]) has never been found in the molecular and combined trees.

### "Clitellatomorpha"

Phylogenetic position of the Clitellata and most "clitellate-like" annelids is stable and quite surprising in the present analyses: they always form a clade, further split into Clitellata and the *Hrabeiella*-Aphanoneura subclade. This clade is supported by hermaphroditism, direct development, loss of parapodia, presence of the unbranched type of ciliary ocelli, metanephridial mantle cell, possibly also by the dorsal pharynx, and by primarily freshwater/soil habitats [3] (though many of these characters are absent or unknown either in Aphanoneura or in *Hrabeiella*). The intra-clitellate position of the Aphanoneura was not supported by reliable morphological synapomorphies [[31,32]; cf. [33]], and exclusion of aphanoneurans from the Clitellata was also indicated by most previous molecular analyses [8,14,31,34-37]. The recent molecular analyses, on the contrary, seem to corroborate close relationships between Clitellata and (some of) the "clitellate-like polychaetes" [7,8,16,31,37,38]. The close relationships of the Clitellata to some eunicidans or dinophilids [15,16], or to TCC [7,8] have never been found in the present analyses.

### Pogonophora and "basal Canalipalpata"

In the present analyses, the Pogonophora (= Siboglinidae) is closely related to the "Clitellatomorpha" or, in absence of the latter group, Pogonophora alone groups as a sister group of SSC. Three groups of the traditional Canalipalpata, namely, Oweniidae, *Magelona*, and Chaetopteridae, were usually placed among the most basal annelids, or even outside the Annelida, in the present all-species analyses [7-9,15,17]. The taxon-exclusion analyses suggested that magelonids and oweniids might in fact be attracted strongly towards the remote annelid outgroups. In absence of all other "problematic" annelids, Oweniidae alone grouped consistently with the Pogonophora (supported predominantly by the intraepidermal nerve cord [7,20]), and *Magelona *either with the Paraonidae (uMP) or as a sister group of the Cirratuliformia-SSC superclade (BI). On the contrary, basal placement of the Chaetopteridae cannot be excluded [9].

### Amphinomida and *Aberranta*

In the most present analyses, *Aberranta *is a sister group of the Amphinomida, and the whole clade is one of the basalmost annelid branches, far from the other Aciculata [7,8,15]. However, in the taxon-exclusion tests, the *Aberranta*-Amphinomida clade (weakly supported by median prostomial antennae and parapodial branchiae) is placed as a sister group of the Phyllodocida (BI) or even within it (uMP). It is then possible that the *Aberranta*-Amphinomida clade is, in fact, one of subgroups of the monophyletic Aciculata [10,11], misplaced in most molecular trees (due to their strong attraction towards the outgroups?). Therefore, it seems rather premature to regard Amphinomida as a basal group, "based on the tetraneurous organization of the nervous system" [7]; moreover, the presence of several peripheral longitudinal nerves is not limited to the amphinomids [27].

### "Core archiannelids" (Nerillidae, Polygordiidae, Protodrilidae, *Protodriloides*)

Monophyly of the "Archiannelida" has been rejected by all modern authors [4], whereas the monophyly of Protodrilida (= Saccocirridae + Protodrilidae + *Protodriloidides*) as well as its spionidan affinities has been proposed recently [4]. The polygordiids were regarded as relatives of protodrilidans or of opheliids [39], or as the canalipalpatans of uncertain position [11]. Surprisingly, the molecular trees tend to include a clade covering most of the traditional "archiannelids" [8]. In the present all-taxa BI tree, there is a well-supported basal annelid clade including Nerillidae-Protodrilidae and Polygordiidae-Saccocirridae. Although Nerillidae were considered aciculatans of uncertain position [11,22], closer relationships of the Nerillidae with any Aciculata have never been retrieved in the present analyses. In conclusion, it is an interesting working hypothesis that the "Archiannelida" (less Dinophilidae and possibly also *Protodriloides*) might form a clade [8], either basal, or close to the SSC [4,11,40-43].

### Sipunculida and Dinophilidae

The Sipunculida have been considered a separate phylum by most authors [6]. Although they as adults do not exhibit any signs of segmentation, sipunculids show transitional stages of segmentation during development of their ventral nerve cord [44,45]. Analyses of the mitochondrial genomes [46-51] as well as phylogenomics [9] suggest that the sipunculids are an annelid ingroup. The unique correspondence of podocyte lining the metanephridia appears to join Sipunculida with the Sabellida and Terebelliformia [33]. The pharyngeal apparatus in the sipunculid pelagosphere larvae is similar to that found in the Cirratuliformia [52]. In the present trees, the sipunculids have always been found as an annelid ingroup, often close to the Dinophilidae. No closer relationships between sipunculids and terebelliformians, sabellidans, oweniids or chaetopterids [7,15,33] were recovered here. The enigmatic Dinophilidae were never found to be closely related to the Dorvilleidae (or Eunicida), i.e. in the placement expected by the morphologists [10,42,43]. Even *Parapodrilus*, one of the presumably dinophilid-related, "progenetic" dorvilleids [42], groups consistently with other dorvilleids within the Eunicida and shows no affinities to the Dinophilidae [25,35]. In the taxon-exclusion analyses, both Dinophilidae and Sipunculida group within the Cirratuliformia (uMP), or they are a sister group of the Pogonophora-"Clitellatomorpha" superclade (BI). In conclusion, close affinities between Dinophilidae and Sipunculida (supported by the shared loss of chaetae, parapodia, circulatory system, and palps) appear as a possible working hypothesis [8], but the precise position of this clade remains uncertain.

### Myzostomida

Relationships of the Myzostomida, traditionally regarded as aberrant polychaetes, probably phyllodocidans [6,10,11], are uncertain. They may be either basal protostomes [53] or platyzoan relatives [54,55]. Their highly aberrant sequences (the longest branch in the present BI tree) and uncertain homology of many morphological characters do not allow to deduce their relationships precisely. Nevertheless, the numerous annelid-like traits of the Myzostomida [56-58] still could corroborate some polychaete-myzostomid proximity. Recently, Bleidorn *et al*. [48,49] re-examined this issue by analysis of four nuclear genes and a mitochondrial genome and showed myzostomids are likely part of the annelid radiation. On the contrary, in the comprehensive phylogenomic analysis of the Metazoa [9], Myzostomida are placed within the Platyzoa [cf. [49]]. In the present analyses, the myzostomids seem to belong into the Annelida (possibly into the Aciculata, as suggested by the taxon-exclusion tests). However, the taxon sampling in this paper does not provide a suitable test for alternative hypotheses due to lack of most of the possible non-annelid relatives of the Myzostomida (Gnathifera, Cycliophora, Platyhelminthes [9,53-55]).

## Conclusion

The combined ("total-evidence") phylogenetic analysis provides a modified view of annelid evolution. Several higher-level annelid taxa are suggested by our analyses, i.e. Phyllodocida, Eunicida, the orbinioid-parergodrilid clade, Cirratuliformia, sabelloid-spionoid clade, terebelloid-capitellid clade (incl. Echiurida), and "Clitellatomorpha". Although the deep-level evolutionary relationships of Annelida remain poorly understood, we propose sister-group relationships between the Phyllodocida-*Aberranta*-Amphinomida and Eunicida-OPC clades, between the Cirratuliformia and SSC, and possibly also between the "Clitellatomorpha" and Oweniidae-Pogonophora clades. Two unorthodox clades, "core Archiannelida" and Dinophilidae-Sipunculida, are proposed here.

## Methods

### Taxonomy, datasets, and data combination

The present analysis was performed to include all available "families" of the Polychaeta, plus representatives of the Clitellata, Echiurida, Pogonophora, Myzostomida, Sipunculida, Mollusca, and Brachiopoda (as a rooting outgroup). All but a few annelid nominal "families" were included; the exceptions were enigmatic annelid (?) genera *Lobatocerebrum, Diurodrilus *[5], and *Jennaria*, "mesozoan" Orthonectida (annelid affinities of which were proposed occasionally [59]), Hartmaniellidae (Eunicida), and several, mostly pelagic or parasitic subgroups of the Phyllodocida, for which no molecular data were available at time.

The morphological dataset (MOR) included 93 characters [see Additional Files 1 and 2]. The gene sequences were obtained from GenBank. The dataset included six genes, both protein-coding (*EF1α*, histone *H3*, *COI*) and ribosomal (*18S*, *28S*, and *16S*), and both nuclear (*18S*, *28S*, *EF1α*, *H3*) and mitochondrial (*16S*, *COI*) [see Additional File 3]. The protein-coding sequences were translated to amino acids and then aligned with CLUSTAL W under default settings for gap costs (gap opening penalty 10.00; gap extension penalty 0.20). The alignment of sequences for ribosomal RNA genes was conducted in the on-line version of MAFFT v6 in the E-INS-i mode [60]. Ambiguous positions were excluded by using Gblocks [61]. The saturated positions were not excluded [8].

The combined datasets were completed by introducing question marks for the absent data partitions. To minimize the number of missing entries in the dataset, composite terminals were constructed with individual partitions from different species of a higher taxon, usually a nominal "family" [see Additional File 4].

### Phylogenetic analyses

Bayesian phylogenetic analysis was conducted with a Metropolis-coupled Markov chain Monte Carlo algorithm [62] as implemented in MrBayes v3.1.2 [63]. MrModelTest v2.2 [64], a simplified version of ModelTest 3.06 [65], and PAUP* v4.0b10 [66] were used to estimate GTR+Ã substitution model as the best-fitting for all molecular character sets (based on both AIC and hLRT criteria). Morphological characters were treated with the standard discrete model assuming gamma-shaped rate variation and variable coding bias. Model parameters were unlinked across partitions. Two independent runs of combined analysis with 10 Markov chains each were conducted for 10,000,000 generations with a sample frequency of 100 (heating 0.1). The first 61,000 trees from each run were discarded as burn-in; convergence between the two runs was estimated using diagnostics criteria produced by the "sump" command in MrBayes (PSRF [TL] = 1.001). The remaining 78,000 trees were used for reconstruction of a 50% majority-rule consensus tree. Testing the influence of burnin value on the consensual tree revealed a high stability of the tree topology within the whole investigated range (burn-in 61,000 to 90,000).

The maximum-parsimony (MP) analysis was applied to MOR, molecular, and combined data matrices (NONA version 2.0 [67]: heuristics, option "hold10000 mult*100 hold/100", unconstrained search strategy with TBR branch swapping). Bremer indices of branch support and bootstrap support values were calculated by NONA (options "bsupport10000" and "mult*100 hold/10" with 1,000 replications, respectively).

In addition, two "experimental" MP analyses (both in NONA: option "hold10000 mult*100 hold/100", unconstrained search strategy with TBR branch swapping) were performed.

(1) The modified "slow-fast" method [68] was used to remove characters (both morphological and molecular) that were supposedly responsible for stochastic information noise. New datasets were constructed, in which only characters with no observed variability within one of the 11 clades (that were present in both all-taxa BI and MP trees) were included. The analysed clades included Cirratuliformia (5 spp.), SSC (6), "Clitellatomorpha" (7), TCC (10), Phyllodocida (19), Eunicida (6), Pogonophora (4), Amphinomida (2), Opheliidae-Scalibregmatidae (2), Sipunculida (2), and Mollusca (3). All reduced datasets were then concatenated to a new, weighted dataset, in which each original character received a weight from 11 to zero. Moreover, all characters with more than 80% of ambiguous (unknown, inapplicable, or polymorphic) character states were excluded. The final weighted dataset included 17,268 informative "characters" (601 morphological and 16,667 molecular).

(2) To test preliminary results suggesting that ingroup and outgroup taxon selection affected internal relationships, we ran several taxon-exclusion analyses. The "problematic" species/clades (Myzostomida, Opheliidae, Scalibregmatidae, Fauveliopsidae, Serpulidae, *Magelona*, Oweniidae, Chaetopteridae, Pectinariidae, *Apistobranchus*, Polygordiidae, Saccocirridae, Protodrilidae, *Protodriloides*, Nerillidae, Histriobdellidae, Dinophilidae, Sipunculida, Hesionidae, Paralacydoniidae, Nepthyidae, Pilargidae, Tomopteridae, *Aberranta*, Amphinomida) were identified as follows: (i) their position was highly unstable in comparison of the all-taxa uMP, wMP and BI trees; and/or (ii) their presence/absence caused important topological changes; and/or (iii) they were consistenly placed basally or even outside the Annelida in both MP and BI trees. Topological effects of including/removing the species identified as "problematic" were tested by constructing an unrooted "backbone tree" to which individual "problematic" species were appended in one-by-one manner.

## Authors' contributions

JZ conceived this study, performed the MP analyses, and wrote the manuscript. PŘ and JJ edited and aligned sequences and performed some analyses. LP performed most of the Bayesian analyses. All authors read and approved the final manuscript.

## Supplementary Material

Additional file 1 **List of morphological characters**. The data provided include an annotated list of 93 morphological characters used in the combined phylogenetic analyses. Click here for file

Additional file 2 **Morphological dataset**. The data provided include a data matrix including 89 terminals (including outgroups, Psammodrilidae and *Spinther*) and 93 morphological characters. Click here for file

Additional file 3 **Concatenated molecular dataset**. The data provided include an alignment of the six concatenated molecular data partitions (COI, EF1α, H3, 18S, 16S, 28S). Click here for file

Additional file 4 **List of taxa used in the analyses**. The data provided include a list of taxa used in the analyses with GenBank accession numbers. Click here for file
