# Supplementary material for: Phylogeny of Annelida (Lophotrochozoa): total-evidence analysis of morphology and six genes
Source: BMC Evol Biol. 2009 Aug 6;9:189. doi: 10.1186/1471-2148-9-189 (PMC2732625; doi:10.1186/1471-2148-9-189)
Supplement: Additional file 1 — List of morphological characters. The data provided include an annotated list of 93 morphological characters used in the combined phylogenetic analyses. [file 1471-2148-9-189-S1.doc]

**Additional File 1**

**List of morphological characters**

The morphological dataset (MOR) included 93 characters. Unless otherwise stated, they were adopted from publications by Rouse and Fauchald [1, 2, 3, 4] and corrected according to [5] (e.g. homology of anterior body regions and appendages in the Pogonophora, presence/absence of the grooved palps in the Polygordiidae). A modified multistate coding [1] has been accepted.

**1. Segmentation**: (0) absent, (1) present. Mollusca are coded as non-segmented, Echiurida, Sipunculida, and Myzostomida as "?".

**2. Longitudinal muscles bands**: (0) absent, (1) present. Sipunculids coded as "absent" as they have more longitudinal muscles than annelids and the homology is uncertain [6].

**3. Circular muscles**: (0) present, (1) absent [7].

**4. Coelom**: (0) absent, (1) present.

**5. Head structure**: (0) prostomium and ringlike peristomium, (1) prostomium and two-ringlike peristomium, (2) prostomium and peristomium limited to lips. The structure of the peristomium in some terebellidans, spionidans, pogonophorans, and aphanoneurans was reinterpreted according to [5] (*contra* [1]) [**unordered**].

**6. Retractable head**: (0) absent, (1) present. The traditional Cirratuliformia (incl. fauveliopsids and sternaspids) coded as having retractable head [1, 5, 8], Sipunculida coded according to [10].

**7. Annulated prostomium formed by fused palp bases**: (0) absent, (1) present. The unique structure of the Glyceridae and Goniadidae is coded according to [11].

**8. First body segment**: (0) similar to following segments, or more or less reduced, (1) surrounding head, (2) fused to head, (3) elongated to form frenular/vestimental region [**unordered**].

**9. Second body segment**: (0) short, (1) elongated to form trunk. All Pogonophora (incl. *Osedax*) coded as having elongated 2nd body segment [5].

**10. Anterior cirri**: (0) absent, (1) present.

**11. Palps**: (0) absent, (1) present, (2) buccal tentacles, (3) sensory palpodes. Terebelliformia coded as having no palps but nonhomologous buccal tentacles [12]; several "scolecidan" and "archiannelidan" groups are coded as having "sensory palpodes", presumably homologous to the palps [5, 12] [**unordered**].

**12. Buccal tentacles retractable into mouth**: (0) absent, (1) present. Coded as present in Alvinellidae and Ampharetidae [9].

**13. Palp origin**: (0) prostomial, (1) peristomial.

**14. Number of palps**: (0) one pair of palps, (1) multiple palps, or branchial crown.

**15. Median prostomial antenna**: (0) absent, (1) present.

**16. Lateral prostomial antennae**: (0) absent, (1) present.

**17. Peristomial cirri**: (0) absent, (1) present.

**18. Palp structure**: (0) solid, (1) grooved, (2) internal canal [**ordered**].

**19. Parapodia**: (0) absent, (1) present.

**20. Shape of parapodia**: (0) with similar rami, (1) with neuropodia larger, (2) tori, (3) "spiomorph" [**unordered**].

**21. Dorsal cirri**: (0) absent, (1) present, (2) some transformed into elytra [**ordered**].

**22. Ventral cirri**: (0) absent, (1) present.

**23. Parapodial branchiae**: (0) absent, (1) present.

**24. Dorsal branchiae**: (0) absent, (1) present, (2) limited to a few anterior chaetigers [**ordered**].

**25. Lateral organs/dorsal cirrus organs**: (0) absent, (1) present. Both lateral and dorsal-cirrus organs considered homologous [13, 14].

**26. Dorsal organs**: (0) absent, (1) present.

**27. Epidermal papillae**: (0) absent, (1) present.

**28. Pygidial cirri**: (0) absent, (1) one pair, (2) two or more pairs [**ordered**].

**29. Ventral glandular area on anterior segments**: (0) absent, (1) present, (2) present with posterior ventral stripe. Coded according to [8], for Oweniidae [15] [**ordered**].

**30. Ventral ciliary field**: (0) absent, (1) present, (2) ciliary groove. Coded according to [5, 16] [**unordered**].

**31. Duogland system**: (0) absent, (1) present.

**32. Circulatory system**: (0) absent or limited, (1) closed.

**33. Heart body**: (0) absent, (1) present.

**34. Nervous system**: (0) subepidermal, (1) intraepidermal [5, 8].

**35. *Corpora pedunculata***: (0) absent, (1) present [5].

**36. Nuchal organs**: (0) absent, (1) present, (2) internalized [12, 13, 17] [**ordered**].

**37. Nuchal organ structure**: (0) pits or grooves, (1) posterior projections, (2) caruncles, (3) positioned underneath ventral roots of circuesophageal connect [17] [**unordered**].

**38. Branchial ocelli**: (0) absent, (1) present [13].

**39. Cerebral commissures**: (0) four (*dcvr*, *vcvr*, *dcdr*, *vcdr*, or modified), (1) none [12].

**40. Nuchal commissure**: (0) absent, (1) present [12].

**41. Commissural ganglion**: (0) absent, (1) present [12].

**42. Dorsal ganglia**: (0) absent, (1) present [11, 12].

**43. Phaosomes**: (0) absent, (1) present [13].

**44. Unpigmented ciliary ocelli**: (0) absent, (1) present with branched ciliary shafts, (2) present with unbranched cilia. Coded according to [13, 19], sipunculid ocelli coded as having branched ciliary shafts [18] [**unordered**].

**45. Spionidan/protodrilidan light-sensitive "statocysts"**: (0) absent, (1) present [13].

**46. Chaetae**: (0) absent, (1) present, (2) calcified [**ordered**].

**47. Aciculae**: (0) absent, (1) present. Aciculata, Myzostomida, and Orbiniidae coded as having aciculae; the acicula-like chaetae of *Apistobranchus* and Psammodrilidae are not considered homologous with the true aciculae [20].

**48. Compound chaetae**: (0) absent, (1) with one ligament, (2) with two ligaments, (3) with a fold [**unordered**].

**49. Uncini/hooks**: (0) absent, (1) present, simple, with intermediate filaments, (2) hooded, (3) bearded. Uncini and hooks considered homologous, lumbrinerid hooked chaetae considered non-homologous to them [21, 22] [**unordered**].

**50. Lyrate/forked chaetae**: (0) absent, (1) present. Presence of this kind of chaetae was treated as a possibly synapomorphy of some "scolecidans" [21].

**51. Chaetal arrangement**: (0) transversal rows, (1) notopodial bundles, (2) additional short rows. Some "canalipalpatan" and "scolecidan" taxa coded according to [20] [**unordered**].

**52. Neuropodial chaetal turnover**: (0) ventrad, (1) dorsad, (2) chaetal inversion. Coded for some "canalipalpatan" and "scolecidan" taxa according to [8, 9, 20]; sabellariid chaetal arrangement considered not homologous with the sabellid-serpulid chaetal inversion [23] [**unordered**].

**53. Fine silk notochaetae**: (0) absent, (1) present.

**54. Stomodaeum**: (0) absent/occluded, (1) ventral buccal organ, (2) axial muscular proboscis, (3) axial simple proboscis, (4) ventral muscular proboscis, (5) dorsal pharynx. *Hrabeiella* is coded as having clitellate-like stomodaeum [24], Sipunculida and Myzostomida coded after [10, 25] [**unordered**].

**55. Dorsolateral folds**: (0) absent, (1) present, (2) modified into tentacular crown. Clitellata coded according to [17, 26-29], Sipunculida following [29] [**ordered**].

**56. Axial-stomodaeum jaws**: (0) no jaws, (1) lateral pair of jaws, (2) DV pairs, (3) cross or circle of jaws [**unordered**].

**57. Jaw venom gland**: (0) absent, (1) present [5].

**58. Proventricle**: (0) absent, (1) present.

**59. Eversibility of ventral buccal organ**: (0) eversible, (1) non-eversible.

**60. Ventral-proboscis jaws**: (0) ctenognath, (1) prionognath, (2) labidognath, (3) absent [**unordered**].

**61. Gular membrane**: (0) absent, (1) present [1, 5, 8].

**62. Gut**: (0) straight, (1) lateral folds, (2) side branches, (3) coiled, (4) occluded, (5) coiled with anterior anus [**unordered**].

**63. Trophosome**: (0) absent, (1) present. Coded as present in all pogonophorans excluding *Osedax*.

**64. Adult nephridia**: (0) protonephridia, (1) metanephridia, (2) protonephridia with funnel (protonephromixia). For nephridial ultrastructural characters (##64-67) see [30-33] [**unordered**].

**65. Terminal cell**: (0) monociliate, (1) multiciliate.

**66. Filter of terminal cells with clefts**: (0) absent, (1) present.

**67. Microvillar filter**: (0) absent, (1) present.

**68. Ciliophagocytal organ**: (0) absent, (1) present.

**69. Distribution of segmental organs**: (0) in most segments, (1) anterior sterile nephridia and posterior gonoducts, (2) one pair of anterior nephridia, posterior gonoducts [**ordered**].

**70. Clitellum**: (0) absent, (1) present.

**71. "Head kidneys"**: (0) protonephridia, (1) metanephridia [30].

**72. Nephridial podocyte lining**: (0) absent, (1) present [30].

**73. Metanephridial mantle cell**: (0) absent, (1) present [30].

**74. Metanephridial duct**: (0) paired, (1) unpaired dorsal.

**75. Gonoducts**: (0) nephridial/coelomoduct, (1) separate, (2) with distal prostate-glandular part [34] [**unordered**].

**76. Hermaphroditism**: (0) absent, (1) present.

**77. Sperm mitochondrial interpolation**: (0) absent, (1) present. For sperm ultrastructural characters (##77-79) see [33, 35], the sperm ultrastructure of the Chrysopetalidae coded after [36].

**78. Sperm flagellum**: (0) posterior, (1) anterior, (2) absent, (3) two [**unordered**].

**79. Acrosome**: (0) absent, (1) present, (2) acrosomal tube [**ordered**].

**80. Prototroch**: (0) absent, (1) present. Larval characters (##80-88) coded after [3, 4].

**81. Metatroch**: (0) absent, (1) present.

**82. Food groove**: (0) absent, (1) present.

**83. Oral brush**: (0) absent, (1) present.

**84. Akrotroch**: (0) absent, (1) present.

**85. Meniscotroch**: (0) absent, (1) present.

**86. Telotroch**: (0) absent, (1) present.

**87. Neurotroch**: (0) absent, (1) present.

**88. Apical tuft**: (0) absent, (1) present.

**89. Building/cementing organ**: (0) absent, (1) present. Coded as a possible synapomorphy of pectinariids and sabellariids [8].

**90. Tube**: (0) absent, (1) sediment/mucous, (2) calcareous, (3) chitin-proteinaceous [**unordered**].

**91. Mitochondrial gene order *T-nad4L-nad4***: (0) absent, (1) present [37].

**92. Mitochondrial gene order *cox1-N-cox2***: (0) absent, (1) present [37].

**93. Mitochondrial gene order *Q-nad6-cob-W-atp6-R***: (0) absent, (1) present [37].

# References

1. Rouse GW, Fauchald K: **Cladistics and polychaetes**. *Zool Scr* 1997, **26**:139-204.

2. Rouse GW: **Trochophore concepts: ciliary bands and the evolution of larvae in spiralian Metazoa**. *Biol J Linn Soc* 1999, **66**:411-464.

3. Rouse GW: **Polychaetes have evolved feeding larvae numerous times**. *Bull Mar Soc* 2000, **67**:391-409.

4. Rouse GW: **The epitome of hand waving? Larval feeding and hypotheses of metazoan phylogeny**. *Evol Dev* 2000, **2**:222-233.

5. Rouse GW, Pleijel F: *Polychaetes*. London: Oxford University Press; 2001.

6. Schulze A, Cutler EB, Giribet G: **Phylogeny of sipunculan worms: a combined analysis of four gene regions and morphology**. *Mol Phylogen Evol* 2006, **42**:171-192.

7. Tzetlin AB, Filippova AV: **Muscular system in polychaetes (Annelida)**. *Hydrobiologia* 2005, **535/536**:113-126.

8. Rousset V., Rouse GW, Siddall ME, Tillier A, Pleijel F: **The phylogenetic position of Siboglinidae (Annelida) inferred from 18S rRNA, 28S rRNA and morphological data**. *Cladistics* 2004, **20**:518-533.

9. Rousset V, Rouse GW, Feral JP, Desbruyeres D, Pleijel F: **Molecular and morphological evidence of Alvinellidae relationships (Terebelliformia, Polychaeta, Annelida)**. *Zool Scr* 2003, **32**:185-197.

10. Tzetlin AB, Purschke G: **Fine structure of the pharyngeal apparatus of the pelagosphera larva in *Phascolosoma agassizii* (Sipuncula) and its phylogenetic significance**. *Zoomorphology* 2006, **125**:109-117.

11. Orrhage L, Eibye-Jacobsen D: **On the anatomy of the central nervous system of Phyllodocidae (Polychaeta) and the phylogeny of phyllodocid genera: a new alternative**. *Acta Zool* 1998, **79**:215-234.

12. Orrhage L, Müller MCM: **Morphology of the nervous system of Polychaeta (Annelida)**. *Hydrobiologia* 2005, **535**:79-111.

13. Purschke G: **Sense organs in polychaetes (Annelida)**. *Hydrobiologia* 2005, **535/536**:53-78.

14. Purschke G, Hausen H: **Lateral organs in sedentary polychaetes (Annelida) – ultrastructure and phylogenetic significance of an insufficiently known sense organ**. *Acta Zool* 2007, **88**:23-39.

15. Glasby CJ, Hutchings PA, Hall K: **Assessment of monophyly and taxon affinities within the polychaete clade Terebelliformia (Terebellida)**. *J Mar Biol Ass UK* 2004, **84**:961-971.

16. Worsaae K, Nygren A, Rouse GW, Giribet G, Persson J, Sundberg P, Pleijel F: **Phylogenetic position of Nerillidae and *Aberranta* (Polychaeta, Annelida), analysed by direct optimization of combined molecular and morphological data**. *Zool Scr* 2005, **34**:313-328.

17. Purschke G, Hessling R: **Analysis of the central nervous system and sense organs in *Potamodrilus fluviatilis* (Annelida: Potamodrilidae)**. *Zool Anz* 2002, **241**:19-35.

18. Radashevsky VI, Migotto AE: **Photoreceptive organs in larvae of Spionidae (Annelida) and Sipuncula**. *J Zool* 2006, 268:335-340.

19. Hausen H: **Ultrastructure of presumptive light sensitive ciliary organs in larvae of Poecilochaetidae, Trochochaetidae, Spionidae, Magelonidae (Annelida) and its phylogenetic significance**. *Zoomorphology* 2007, 126:185-201.

20. Hoffmann S, Hausen H: **Chaetal arrangement in Orbiniidae (Annelida, Polychaeta) and its significance for systematics**. *Zoomorphology* 2007, 126:215-227.

21. Hausen H: **Chaetae and chaetogenesis in Polychaeta (Annelida)**. *Hydrobiologia* 2005, **535/536**:37-52.

22. Bartolomaues T: **Chaetogenesis in polychaetous Annelida – significance for annelid systematics and the position of the Pogonophora**. *Zool Anal Compl Syst* 1998, **100**:348-364.

23. Kieselbach D, Hausen H: **Chaetal arrangement provides no support for a close relationship of Sabellidae and Sabellariidae (Annelida)**. *J Morphol* 2008, **269**:104-117.

24. Purschke G: **Is *Hrabeiella periglandulata* (Annelida, "Polychaeta") the sister group of Clitellata? Evidence from an ultrastructural analysis of the dorsal pharynx in *H. periglandulata* and *Enchytraeus minutus* (Annelida, Clitellata)**. *Zoomorphology* 2003, **122**:55-66.

25. Eeckhaut I, Fievez L, Müller MCM: **Larval development of *Myzostoma cirriferum* (Myzostomida)**. *J Morphol* 2003, **258**:269-283.

26. Hessling R, Purschke G: **Immunohistochemical (cLSM) and ultrastructural analysis of the central nervous system and sense organs in *Aeolosoma hemprichi* (Annelida, Aeolosomatidae)**. *Zoomorphology* 2000, **120**:65-78.

27. Purschke G, Tzetlin AB: **Dorsolateral ciliary folds in the polychaete foregut: structure, prevalence and phylogenetic significance**. *Acta Zool* 1996, 77:33-49.

28. Tzetlin A, Purschke G: **Pharynx and intestine**. *Hydrobiologia* 2005, 535:199-225.

29. Adrianov AV, Malakhov VV, Maiorova AS: **Development of the tentacular apparatus in sipunculans (Sipuncula): I. *Thysanocardia nigra* (Ikeda, 1904) and *Themiste pyroides* (Chamberlin, 1920).** *J Morphol* 2006, 267:569-583.

30. Bartolomaeus T, Quast B: **Structure and development of nephridia in Annelida and related taxa**. *Hydrobiologia* 2005, **535**:139-165.

31. Bartolomaeus T: **Structure, function and development of segmental organs in the Annelida**. *Hydrobiologia* 1999, **402**:21-37.

32. Bartolomaeus T, Ax P: **Protonephridia and metanephridia – their relation within the Bilateria**. *Z Zool Syst Evol* 1992, **30**:21-45.

33. Harrison FW, Gardiner SL: **Annelida**. In *Microscopic Anatomy of Invertebrates*. New York: Wiley-Liss; 1992. [Harrison FW (Series Editor), vol 7].

34. Purschke G, Jördens J: **Male genital organs in the eulittoral meiofaunal polychaete *Stygocapitella subterranea* (Annelida, Parergodrilidae): ultrastructure, functional and phylogenetic significance**. *Zoomorphology* 2008, 126:283-297.

35. Zrzavý J, Hypša V, Tietz DF: **Myzostomida are not annelids: molecular and morphological support for a clade of animals with anterior sperm flagella**. *Cladistics* 2001, **17**:170-198.

36. Tzetlin AB, Dahlgren T, Purschke G: **Ultrastructure of the body wall, body cavity, nephridia and spermatozoa in four species of the Chrysopetalidae (Annelida, "Polychaeta").** *Zool Anz* 2002, 241:37-55.

37. [Bleidorn](http://wos.isiknowledge.com/CIW.cgi?SID=B5ebhPbhil5aBnf3GLO&Func=OneClickSearch&field=AU&val=Bleidorn+C&curr_doc=3/143&Form=FullRecordPage&doc=3/143) C, Podsiadlowski L, Bartolomaeus T: **The complete mitochondrial genome of the orbiniid polychaete *Orbinia latreillii* (Annelida, Orbiniidae) – a novel gene order for Annelida and implications for annelid phylogeny**. *Gene* 2006, **370**:96-103.
