# Supplementary material for: Phylogeny of Annelida (Lophotrochozoa): total-evidence analysis of morphology and six genes
Source: BMC Evol Biol. 2009 Aug 6;9:189. doi: 10.1186/1471-2148-9-189 (PMC2732625; doi:10.1186/1471-2148-9-189)
Supplement: Additional file 2 — Morphological dataset. The data provided include a data matrix including 89 terminals (including outgroups, Psammodrilidae and Spinther) and 93 morphological characters. [file 1471-2148-9-189-S2.doc]

**Additional File 2**

**Morphological dataset**

**ABERR**_*Aberranta*

11?1100?001-001001111110?00?010100?100??????010001??041-00-3?00??????0??????????????????00???

**ACOET**_*Panthalis*

11?12001011-0011001121000001000??011000011??011000??120210--0201???000?000?0????????????01???

**ACROC**_*Macrochaeta*

1??10000001-100001100001?0100001100100??????010300??01?-000-??0????020???000001?????????00???

**AEOLO**_*Aeolosoma*

10010000000---000-0----0000001010101300?0?10010000??011-000-?001???000?0100100?00??0000000???

**ALCIO**_*Alciopina*

1??12000011-0011001111??00010000?00100??????011000??020000--0002001000?0??00001?????????00???

**ALVIN**_*Paralvinella*

1?0100020021--000-12?002?000000110?0-0????0001001010010-001-1101???010?0000010110??00???01???

**AMPHA**_*Ampharete-Auchenoplax-Isolda*

110100020021--000-1200021000100110010010000001001010010-001-1101???010?0000000111??0010101???

**AMPHI**_*Chloeia-Eurythoe-Paramphinome*

11012000001-001100101110?0010001000120010102021000??041-00-30001???000?0000000111100011100???

**APHRO**_*Aphrodita*

11112001011-00100011210000010001001100001100011000??120200--0201???000?00000???10??011?100???

**APIST**_*Apistobranchus*

11?1?000001-10000113?0001002000100?1?00011??01?000??011-000-0001???010?00000001?????????00???

**ARENI**_*Abarenicola-Arenicola*

11010000000---000-12001010000001000100????0001003011031-00--1001???010?0000000110000011100???

**BRACH**_*Terebratalia-Terebratula*

00?1?0---00---???-0----000000?01?100-0????00010000??0?0-00--0?01???0?0?0000000?00000000000000

**CAPIT**_*Notomastus*

11010000000---000-1200??10000000?00100????0001002000030-00--1001???0?000001000110000011100???

**CHAET**_*Chaetopterus*

11010000001-100?01120000?0000001100110100100?1?0100?00?-00--0001???010000000001?1000000101???

**CHRYS**_*Dysponetus*

11112001011-0011001011000001000100?100??????011100??020100--0001???000?0000110110000111100???

**CIRRA**_*Cirratulus-Cirriformia*

11010000001-11000110?00100000001100100?0000001000000011-000-0001???020?0000000110000011000???

**CLITE**_*Capilloventer*

11010000000---000-0----0000000010000-010??12010000??050-00--0001???0?1101011102?0??000[01]000???

**CLITE**_*Hirudo-Lumbriculus*

11010000000---000-0----0000000010000-010??12010000??050-00--0001???0?1101011102?0??0001000???

**CLITE**_*Lumbricus*

11010000000---000-0----0000000010000-010??12010000??050-00--0001???0?1101011102?0??000?000111

**CLITE**_*Stylaria*

11010000000---000-0----0000000010000-010??12010000??050-00--0001???0?1101011102?0??000?000???

**COSSU**_*Cossura*

11010000000---000-10000?0002000100?100??????010000000?0-00--000??????0?????0001?????????00???

**CTENO**_*Ctenodrilus*

10010000000---000-0----100000001100100??????010000??011-000-0001???020?0000????10??0011000???

**DINOP**_*Dinophilus-Trilobodrilus*

11001000000---000-0----000020100?0?100????0000-------11-000300001?1000?0???000111??1011100???

**DORVI**_*Microdorvillea-Ophryotrocha-Parougia-Protodorvillea*

11011000001-00010011[01][01]00100[12]000100?100????02011200??041-00-00001110000???00[01]0[02]111??1011100???

**DORVI**_*Parapodrilus*

11011000000---000-1100001001000100?100????02011000??041-00-30001110000???0000?111??1011100???

**ECHIU**_*Arhynchite-Bonellia-Listriolobus*

?001?00??00---000-0----0000000010000-0?0??0?010000??0?0-00--03010100?01000000011110??11100???

**ECHIU**_*Urechis*

?001?00??00---000-0----0000000000000-0?0??0?010000??0?0-00--03010?00?00000000011110??11100101

**EUNIC**_*Eunice-Marphysa*

11011000001-001110111110100100010001000101000112?0??041-00-20001???000?0000000111??1011100???

**EUPHR**_*Euphrosine*

11?12000001-001100101110?0010001000120??????021000??041-00-30001???000?00000?????????1??00???

**FAUVE**_*Fauveliopsis*

1??10100000---000-100000?0101001?0?100??????01?000??011-000-?10??????0?????0????????????00???

**FLABE**_*Diplocirrus-Flabelligera*

11010100001-100001100001?01000011001000011??010300??011-000-0101???020?0000000110??0010?00???

**FRENU**_*Galathealinum-Siboglinum*

11010003101-110000120000000000011100-0????100100100?000-0?--041????020???00000110??0011?03111

**GLYCE**_*Glycera*

11012010001-00010011111000010000?00100011100011100??020310--0002001100?0??00???10000101100???

**GONIA**_*Goniada*

11012010001-00010011110000010000?001?00111??011100??0203?0--??02001010?0??0????10??0101?00???

**HESIO**_*Hesione-Ophiodromus*

11012000011-00?10011110000010001001100001101011100??020000--0001000100???00000110000101100???

**HISTR**_*Histriobdella*

11?1?00?001-0011?0??????000?0010?00100??????00-------41-00-10000???000?0??100210??????0?00???

**HRABE**_*Hrabeiella*

1??10000000---000-0----000000001?00200????02010000??05?-00--0?01???000?0?0?1001?????????00???

**LUMBR**_*Lumbrineris-Ninoe*

11011000000---000-1100[01]01001000??00100??????0112?0??041-00-20001???000?0000000111??1010100???

**MAGEL**_*Magelona*

11112000001-100000130000100100011000-000110111002020011-000-000?????100????000110000000100???

**MALDA**_*Axiothella-Clymenella-Clymenura*

11012000000---000?12000?00020001000100??????010030110[13]1-00--1001???010?0000000110?00011101111

**MOLLU**_*Chaetopleura-Katharina*

000??0---00---???-0----000000?00?[01]00-0????0000-------?0-00--00011010?000000000110000000100000

**MOLLU**_*Ilyanassa-Nassarius*

000??0---00---???-0----000000?00?000-0????1000-------?0-00--00011110?000000000111100000100000

**MOLLU**_*Nuculana-Yoldia*

000??0---00---???-0----000000?00?000-0????0000-------?0-00--00011110?000000000111000000000000

**MYZOS**_*Myzostoma*

?000?00?000---000-1111?000000000?000-0??????0110?0??0??-00--020011100000?0110101100??10100111

**NEPHT**_*Nephtys*

11112000011-00010010111?00010001001100??????01100???020100--0002001100?0??00???100?0110100???

**NEREI**_*Ceratonereis-Nereis*

11012000011-00010011110000010001001100011101011100??020100--0001???100?0001000110????10100111

**NERIL**_*Paranerilla*

11112000001-0001001000000001010100?100?01?02010100??011-0?0-0000???0?0???00000110??0011100???

**OENON**_*Drilonereis*

11011000000---000-1110001001000100?100??????011000??041-00-1??0??????0?????0????????????00???

**ONUPH**_*Diopatra-Hyalinoecia*

11010000001-001110111110100100010001000111??0112?0??041-00-20001???000?0000000111001010101???

**OPHEL**_*Ophelia-Ophelina*

11110000003---000-10001010020201000100???000010000??031-00--1001???000???00000111?00011100???

**ORBIN**_*Orbinia-Phylo*

11010000000---000-10000111020001?001000100020110012?011-000-000111?000000020??111??1011000111

**OSEDA**_*Osedax*

???10003101-?100000-00-0000000011?00-0????0?010010??000-0?--?40??????0?????0???1????????0????

**OWENI**_*Myriochele-Owenia*

1111?000000-0?000-120000000010010100-0????0001001000011-000-00010000?001000000111100000101???

**PARAL**_*Paralacydonia*

1??12000001-000100111100000?000??0?100??????011100??0200?0--??0??????0?0???????10000010100???

**PARAO**_*Aricidea-Cirrophorus-Paraonis*

11012000003-?0[01]00-100001100200010001000000??010001??011-000-0001???010???00000110000011000???

**PARER**_*Stygocapitella*

11010000000---000-0----000000001100200??????010000??010-000-1?01???000?00020001?????????00???

**PECTI**_*Pectinaria*

1101?0020?20--000-1200021000200110011010000001001010011-001-1101???01001000000110000011111???

**PHOLO**_*Pholoe*

1??12001011-0011001121000001000??01??0??????011100??020210--0001?????0?000??00110010111100???

**PHYLL**_*Eteone-Phyllodoce*

11112000011-00110011110000010000?00100010001011100??0200?0--000200100000??0000110000111100???

**PILAR**_*Ancistrosyllis-Sigambra*

1??12000011-0011001111000001000??00100??????011000??0200?0--??0??????0?????0???10000110?00???

**PISIO**_*Pisione*

11112001011-0000001111??00010000?010-0????01011100??020210--0000001000?0??0000010000101100???

**POECI**_*Poecilochaetus*

11?12000001-1000011300101002000100?11000110111000020011-000-0001???010100000????00???10?01???

**POEOB**_*Poeobius*

1??1?10?001-1000010----100100001100100??????00-------11-000-?101???020?00000001?????????00???

**POLYG**_*Polygordius*

11100000003---0?0-0----000020201010100????01000000??0?1-00--00011110000000??00111100010100???

**POLYN**_*Lepidonotus*

11112001011-00110011210000010001001100001101011000??020210--020101100000000000110010101100???

**POTAM**_*Potamodrilus*

11010000000---000-0----0000001010102300?0?02010000??01?-000-0001???010?0?0?100??????????00???

**PROTD**_*Protodriloides*

11000000001-0000000----0000102110?0100????02?00000??011-000-0000???000?0?0?002010??0000000???

**PROTO**_*Protodrilus*

11110000001-0000020----0?001021101010010001110-------11-000-0000100000????1000111000011100???

**PSAMM**_Psammodrilidae

11010000000---000-1200??00000000-000-0????1001?030100??-00--?001???010???0?000110??0011100???

**QUEST**_*Questa*

1??10000000---000-1000011002000100?100??????010001??011-000-000????0??????20001?????????00???

**SABEL**_*Sabella-Schizobranchia*

11010000001-01000112000000001001000101001000010010?2000-00--0001???020?1010000111?00001001???

**SABLR**_*Gunnarea-Sabellaria*

11012002001-10000112000100000001100100001000010010??000-00--000101102000000000110000011111???

**SACCO**_*Saccocirrus*

11?10000001-0000021?000000010211010100?0???1?10000??001-000-0001???000?00000???11000011100???

**SCALI**_*Scalibregma*

1?010000001-00000-100010100200010001000010000100010?030-00--1001???000?0000?????????????00???

**SCLER**_*Sclerolinum*

11010003101-110000120000000000011100-0????0?0100100?000-0?--041????0?0????0000110??0011?0????

**SERPU**_*Protula-Salmacina-Serpula*

11010000001-0100011200000000?001101101001100010010?2000-00--000110102001010000111100001102???

**SIGAL**_*Sthenalanella*

11112001011-001100112100000100010011000011??011100??020210--0201???000?00000001100101??100???

**SIPUN**_*Phascolopsis*

0001?10??00-----0-0----000000000?00??0?0??0100-------12-00--0501???0?0?1000000111000000100?10

**SIPUN**_*Phascolosoma*

0001?10??00-----0-0----000000000?00??0?0??0100-------12-00--0501???0?0?1000000111000000100???

**SPHAE**_*Sphaerodoropsis*

11012000011-00110011?1??00010001?0?100??????011100??020001--0000001000?0000?????????????00???

**SPINT**_*Spinther*

1??12000000-----0-1001000001000100?0-0??????011?00??0??-00--020??????0??????????????????00???

**SPION**_*Marenzelleria-Polydora*

11110000001-10000113000111020001?0011000110111002020031-000-0001???01010000000110000010101???

**STERN**_*Sternaspis*

10010100000---000-0----100100001??00-0????00010000??03?-00--0101???020?00000????0??0000100???

**SYLLI**_*Eusyllis-Exogone-Proceraea-Typosyllis*

11012000011-001100111100?0010001000100001111011100??020001--00011??0000??00000110??1011100???

**TEREB**_*Amphitrite-Lanice-Pista*

110120020020--000-12000210002001100[01]0010000?01001010011-001-11011?10[01]000000000110??0011101???

**TOMOP**_*Tomopteris*

1??12000011-0000001100??00000000?00100??????01?000??0200?0--0002101000?0??0003010??0000000???

**TRICH**_*Artacamella-Terebellides*

110120020020--000-120002?000?00110?0-0??????01001010011-001-1101???010?00000???10??0011101???

**TROCH**_*Trochochaeta*

11?10000001-1000011300001102000??0?11000110111000020030-00--??01???010?00000???10000010?01???

**VESTI**_*Lamellibrachia-Riftia*

1101?003101-?10000120000000000011100?0????000100100?000-0?--041????020?00[01]0000110??0011?03111
