# Supplementary material for: Phylogeny of Annelida (Lophotrochozoa): total-evidence analysis of morphology and six genes
Source: BMC Evol Biol. 2009 Aug 6;9:189. doi: 10.1186/1471-2148-9-189 (PMC2732625; doi:10.1186/1471-2148-9-189)
Supplement: Additional file 3 — Concatenated molecular dataset. The data provided include an alignment of the six concatenated molecular data partitions (COI, EF1α, H3, 18S, 16S, 28S). [file 1471-2148-9-189-S3.doc]

**Additional File 3**

**Concatenated molecular dataset**

**DATA PARTITIONS**

**1-665 COI**

**666-1300 EF1α**

**1301-1432 H3**

**1433-2251 18S**

**2252-2529 16S**

**2530-4147 28S**

**ABERR**_*Aberranta*

??????????????????????????????????????????????????????????????????????????????????????????????????????????????????????????????????????????????????????????????????????????????????????????????????????????????????????????????????????????????????????????????????????????????????????????????????????????????????????????????????????????????????????????????????????????????????????????????????????????????????????????????????????????????????????????????????????????????????????????????????????????????????????????????????????????????????????????????????????????????????????????????????????????????????????????????????????????????????????????????????????????????????????????????????????????????????????????????????????????????????????????????????????????????????????????????????????????????????????????????????????????????????????????????????????????????????????????????????????????????????????????????????????????????????????????????????????????????????????????????????????????????????????????????????????????????????????????????????????????????????????????????????????????????????????????????????????????????????????????????????????????????????????????????????????????????????????????????????????????????????????????????????????????????????????????????????????????????????????????????????????????????????????????????????????????????????????????????????????????????????????????????????????????????????????????????????????????????????????????????????CTTATGCTGCTAATACATACTTTTACACAGTAATGTTGATAGTTATGGTCCTTAATCGTACATCCTACTTTTACAACAACCAGCTCCGACYTTTTGGGAAGAGCCTTAGATCAAACAATCGGGTCCTTTGCTGGCTTTGGGTTCAGCTCCGCCGGAGTATTTCAATTCTCCTCTTCGATGGTACGTGATATCCTACCATGTTAAAAGCCGAAACTACGCCCTAAAAAGAAAACATACGGGACTCTTTCGCCCGTATTAACTTTTCCTTTAAGTTTAGTTAGACTGTCTCAGGTCCAGGCCGGGTCCACCTCACGGGTACTGCGTCCTGACCTCCTCCCGGTTCCCTTGGGCTAAGTAGTTCTGGGTGGCCGGAACGTAATTATCAAAAGAATTGCCTGAATAATGGTAGGTGCCTCGGTTCTATTTGTTTTTCGGAACTGAGGTAAGGGAAGACGGTCGTTACAGTTTTGTCGCTTAGAATACTAATCGCATTTTAAAAACATGTGCACGACGACTACGATCCCCGGATTGTTTCATGACTCGGCGGCAGCTTCCGACAAGTTTTGGCCATAGTGRTTAGAATCCCCCCGCTTATTGGTGGGGCTCTAGCATATTCGCGGTGTCCGCGTAACAGGTGCGGTTCACAGTTTCGCCAAGGAATGCGTTGTAACCTGTCCGAGGTCGTCCGTTACCTCCTTCTATTGGGCTTTTCCCATGGACAGGGATAGTCCTTTTAATGGTTTGTGATCCTCGTTGGACCCGCGGGGGCAACCGCCGGGGTGTCGAGGACGATCACTTGATCATTA???????????????????????????????????????????????????????????????????????????????????????????????????????????????????????????????????????????????????????????????????????????????????????????????????????????????????????????????????????????????????????????????????????????????????????????????????????????????????????????????????????????????????????????????????????????????????????????????????????????????????????????????????????????????????????????????????????????????????????????????????????????????????????????????????????????????????????????????????????????????????????????????????????????????????????????????????????????????????????????????????????????????????????????????????????????????????????????????????????????????????????????????????????????????????????????????????????????????????????????????????????????????????????????????????????????????????????????????????????????????????????????????????????????????????????????????????????????????????????????????????????????????????????????????????????????????????????????????????????????????????????????????????????????????????????????????????????????????????????????????????????????????????????????????????????????????????????????????????????????????????????????????????????????????????????????????????????????????????????????????????????????????????????????????????????????????????????????????????????????????????????????????????????????????????????????????????????????????????????????????????????????????????????????????????????????????????????????????????????????????????????????????????????????????????????????????????????????????????????????????????????????????????????????????????????????????????????????????????????????????????????????????????????????????????????????????????????????????????????????????????????????????????????????????????????????????????????????????????????????????????????????????????????????????????????????????????????????

**ACOET**_*Panthalis*

????????????????????????????????????????????????????????????????????????????????????????????????????????????????????????????????????????????????????????????????????????????????????????????????AAAAGTCCCTAGCAGAAACATCGCCCGCGCCCCTCTGACCACACCACCCACGAGGCATCCTTCAGGGGCCATCACACAAGTAATCCAGGCCTGCGCCTGGAGCGAGTCCCCTTGTCAGCCGCCAAAACGGCCACCACTCCCCTCTCGGGCTCGTCCACCGCACACACGGACCAATGGCCCTCCCTGCCTGGCAACCAACCACCCC????????????????????????????????????????????????????????????????????????????????????????????????????????????????????????????????????????????????????????????????????????????????????????????????????????????????????????????????????????????????????????????????????????????????????????????????????????????????????????????????????????????????????????????????????????????????????????????????????????????????????????????????????????????????????????????????????????????????????????????????????????????????????????????????????????????????????????????????????????????????????????????????????????????????????????????????????????????????????????????????????????????????????????????????????????????????????????????????????????????????????????????????????????????????????????????????????????????????????????????????????????????????????????????????????????????????????????????????????????????????????????????????????????????????????????????????????????????????????????????????????????????????????????????????????????????????????????????????????????????????????????ATGCTGGTAATACAGACTGTACAACGGTAATGTTGATAGTTATGGTCCTTAATCGTA?ATCCTACTTTTGCAACTGAAATGTCCGACCTTCAGGGAGGACTCTTAGATCAAACAATCGGGTCCTTTGCTGGCTTTGTGCTCAGCTCAGCCGGAGAATTTCAATTCTACTAGTCGATGGTAAGTGACCTCTTACCATTCTAAAGGCCAAAACTACGCCCCGACAGGAAAACATACGGGACTCTTTCGCCCGTATTAACTTTTCCTTTAAGTTTAGTTAGACTGTCTCGGATCCGGGCAGCGTCCACCTCGCGGGTACTGCGTCCCGGTCTCCCTCTGGACCCGTCGGGCTGACTAGTCCGCGGCGGCCAGGACGTAATTATCCAAAGGCGTGCCCGAATAATGGTAGGTGCCTCGGTTCTATTTGTTTTTCGGAACGGAGGTAAGGGAGGACGGTCGTTGCGGTTTTGTCGCCCAGAATACTAATCGAATTTTAAAAACATCTGCACGACGACTACGATCCCCGGATTGTTTCATGACCCGGCGGCAGCTTCCGACAAGTCTTGGCCATAGTGATTAGAATCCCTCCGCCTTTTGGCGGGGCTCTAGCGCCTCCGCCGTGTCGGCGCGACAAGTGCGTTCCACGGTTTCGCCAAGGAATACGTTTGTGTTGGTCCGAGGTCGTCCGTTACCTCCTTCTATTGGGCTTCTCCCATGGACAGGGTTAGACCTTTTAATGGTTTGTGATCCTCGCCGGCCCCGCGGGGGCAACCGTCGCGTGGGCGGGGACGATCGCTTGATCATTA???????????????????????????????????????????????????????????????????????????????????????????????????????????????????????????????????????????????????????????????????????????????????????????????????????????????????????????????????????????????????????????????????????????????????????????????????????????????????????????????????????????????????????????????????????????????????????????????????????????????????????????????????????????????????????????????????????????????????????????????????????????????????????????????????????????????????????????????????????????????????????????????????????????????????????????????????????????????????????????????????????????????????????????????????????????????????????????????????????????????????????????????????????????????????????????????????????????????????????????????????????????????????????????????????????????????????????????????????????????????????????????????????????????????????????????????????????????????????????????????????????????????????????????????????????????????????????????????????????????????????????????????????????????????????????????????????????????????????????????????????????????????????????????????????????????????????????????????????????????????????????????????????????????????????????????????????????????????????????????????????????????????????????????????????????????????????????????????????????????????????????????????????????????????????????????????????????????????????????????????????????????????????????????????????????????????????????????????????????????????????????????????????????????????????????????????????????????????????????????????????????????????????????????????????????????????????????????????????????????????????????????????????????????????????????????????????????????????????????????????????????????????????????????????????????????????????????????????????????????????????????????????????????????????????????????????????????????

**ACROC**_*Macrochaeta*

????????????????????????????????????????????????????????????????????????????????????????????????????????????????????????????????????????????????????????????????????????????????????????????????????????????????????????????????????????????????????????????????????????????????????????????????????????????????????????????????????????????????????????????????????????????????????????????????????????????????????????????????????????????????????????????????????????????????????????????????????????????????????????????????????????????????????????????????????????????????????????????????????????????????????????????????????????????????????????????????????????????????????????????????????????????????????????????????????????????????????????????????????????????????????????????????????????????????????????????????????????????????????????????????????????????????????????????????????????????????????????????????????????????????????????????????????????????????????????????????????????????????????????????????????????????????????????????????????????????????????????????????????????????????????????????????????????????????????????????????????????????????????????????????????????????????????????????????????????????????????????????????????????????????????????????????????????????????????????????????????????????????????CTACCACGGCCCGGCGCCGCTCTGAGCCGGCCATGGCCAGGAACGTCCTGCTAGCGGAGCCGCTCGCCGCCCGCTCCCCTGCCGCGCCCGCCCGAGCCCCCCGGGCAGCGGCCCGTACGCGCCCCCCCCGCT???GTTTCCTCCTACGCTGCTAATGCACACCCTTTGATGGTAATGCTAATAGTCATGGTCATTGATCGTACATCCCACGTTTGTAACAACCAGCCCGGACCGCGCGTCCTGGGTCTCAGATC?AGCAGCCGGGTCCCCTGCTGGCACCTGGATCAGCCCAGCCGGAGTTTCTCAATTCTATTTGTCGACGGGCGGTGACTTCCTCCCGTAGCGAAGGCCAACACTACGCCCCGACAGGAAAACGTGCGGGACTCTTTGGCCCGTACCGACGCTCCTGTGTGGTTCAGTCGGCCTGTCTCTGGCGTGGGAGGTTGTGGCCTATC??GGACTGCATCCCG??CCCTTGCCGGTTCTGCGGGGCTAACCAGTCCTCGGCGGCCGGCCCGTAATTGCCAAAAGCTGGCACGAACGAATGGTAGGTGCCTCGGTTCTGTGTGTTTTCTGAAGCCGAGGTAAGGGAAGACGGTCGTTGCGGCGGTGTCGCCCAGAAAATTAATCGAATTTTAAAATCATGTGCACGACAACCGCGATGCCCGACTCGTATCAAGACTCGGCGCCAGCCCTCACTAGGTTCTGGCCATAGTGATTAGGCTCCCTCCGCTGATTGGTGGAGCTCTGGCGCATTCGCCCTGCGGGCGTAACCAGTGCTTCCCACGGTTCCACAAAGCAGGGCGATTGACTCGGTCCAAGGTTGCCCGTTACCTGGTTATACTCGCACTTTGCGACGGACAGGGATCGTCCTTTTATTGATGGGTGGCACTTGTGGGCCCCGA??CCGCAACCGG??TCTGGGCCAGGACTTCCGCTCCATTGA?????????????????????????????????????????????????????????????????????????????????????????????????????????????????????????????????????????????????????????????????????????????????????????????????????????????????????????????????????????????????????????????????????????????????????????????TAACAAACTCAGCTCGCCAGGAGACGACCGCCCCCGTCACGGGCGCGGGACAGTGTTGGGAGGGCCCGTGCCGGCGGGGTGGGGTGCCTTATCTCTCTTTTGGGGCAGCATACTCGTTGAGCCCCTGGCCGTCTGCTCTGCGCTCCTCGCGTGGAGCAGCGTAACACCTGCTGGTGCACAAACT????????????????????????????????????????????????????????????????????????????????????????????????????????????????????????????????????????????????????????????????????????????????????????????????????????????????????????????????????????????????????????????????????????????????????????????????????????????????????????????????????????????????????????????????????????????????????????????????????????????????????????????????????????????????????????????????????????????????????????????????????????????????????????????????????????????????????????????????????????????????????????????????????????????????????????????????????????????????????????????????????????????????????????????????????????????????????????????????????????????????????????????????????????????????????????????????????????????????????????????????????????????????????????????????????????????????????????????????????????????????????????????????????????????????????????????????????????????????????????????????????????????????????????????????????????????????????????????????????????????????????????????????????????????????????????????????????????????????????????????????????????????????????????????????????????????????????????????????????????????????????????????????????????????????????????????????????????????????????????????????????????????????????????????????????????????????????????????????????????????????????????????????????????????????????????????????????????????????????????

**AEOLO**_*Aeolosoma*

????????????????????????AAAATTTATATTAACATCTCAAAAGATTTAAGGAAGAGATCACTTTGTATAGACCTTCATTCAATCTCTAGCGTATTTATTTTTGTTACTTAATAAATGGAGCCCATAGTTTTCTTATTCAAATATTTGCTCCTCCCATTTATATATTCTCTTCTATGGAGAAACTGATAATGTCTATATCAGAAACTTATCTCAGGGCCAGCCGATAGCATTTCTTTAGAAGCCTCTATCAGCTCTATTTAGTCATATTAAATAGAAATAAATCCAGAAAACGTTCCTATGTTAGCAGTACTAATTACACTCTCTTACATCTCAGTTGGGACTATTATACACACAGGTCTATGTCTTTTTCTGCA?????????????????????????????????????????????????????????????????????????????????????????????????????????????????????????????????????????????????????????????????????????????????????????????????????????????????????????????????????????????????????????????????????????????????????????????????????GTGCGTTCGCCGGCACCGCTTTCTTTGCTTGATACAAAGAACTGTACGCCATTTTCTCTCTCCCATAAAATCTTGCCACTTGCCGTACTTGTGTTCTAGAACTATCTATCCCTACACCCCTGAACGACTGGAGTCAGCTCTATGAGACCTTCAGCGAAGCTCCTCAATATGTTGTAAAGCAGACGCACATTCAGTTGAAACTACTTTGTTTTTCGGAACTTCCCGAACCCAGTGAAAATGAAATGTTCAAGGGATGGACAATCGTATGATGCTTATGACATTGATCCTAAAACCACTGAAAAGCTCTCTTTATGATACATGGCTTATCACTCTCATTGTGTGGCTTATGGCAACACGTCGTCAACCTACCTAAAAGGACCACAGCTCTGAAGAACTCAAAACTTTCCGTAACACTCGTGGCGTCTTAATCTGTGGTGCTCTGAACTCACCAAAGAAACTGACGAATCAAAGTGCCACTGCCATCAAATCAAGCTCTTCGTTGTTTCCTCCTTCATCNGTGAACGGTAGAAATTCAAGGCGAAT????????????????????????????????????????????????????????????????????????????????????????????????????????????????????????????????????????????????????????????????????????????????????????????????????????????????????????CCTGTTTCCTCTTATGCTGCTAATACAAGCCTTAACACGGTAATGTTAACACCCATGATTCTTAATCGTACATCCTACTTTTGTAACC??GAGCTCCGCCCCTTCGGGAAGAGCCGTAGATCAAACAATCGGCATCTTTGCTGGCTTTGAGTTCAGCTCAGCCGGAGTATTTCAATTCTACTCTTCGATGGTACGTGATATCCTACCATGGCAAAGGCCAACACTACGCCCTGGCAGGAAAACATACAGGCTCTCTTCGCCTGTATTAACTTTTCCTTTAAGTTTAGTTAGACTGTCTCGGGTGCAGGCGACGTCCACCTCGCGGGTACTGTGACCTGTCCTTGTGCTGTGTGCGCCGGGCTCGGCTGACGTGGGGGGCAAGAGCGTAATTACCAAAAGGTGTGCTTGAATAATGGTAGGTGCCTCGGTTCTATTTGTTTTTCGGGACCGAGGTAAGGGAAAACGGTAACTACGGCTTTGTCG?CTAGAATACTAATCGTGTTTTAAAAACATCTGCACGACGACTACGATCCCCGGCTTGTCTCATGACCCGGCGGCAGCTCCCGACAAGTTTTGGCCGTAGTGATTAGAATCCCCCCGCCTATTGGTGGGGCTCTAGCACATTCGTCGCGTCGGCG?AACAAGTGCGTTTCACAGTTTCGCCAAGGAATCCGTTTTTGCTGGCCCGAGGTTGTCCGTTAACTCCTTCTATTGGGCTTATGCCATCGACAGGGGTAGTCCTTTTGATGGTTTGTGGTCCTCGTTGGACCCGC?AGGGCAACTT?CGCCGT?GCGAGGATATGCAAGACGTGTTTGGAA?????????????????????????????????????????????????????????????????????????????????AGAGAGCTGAAAAAAATTACAGAGTATAGGCTTTATTTTGATATAACAAA??????????????CAAAATCAGTGCGACAAAGGATA????ATCATCCTGAAAATAAAGAACAATTCAGTCAGATCAAAATTAGCCTTAAGCTATTTTTCTAGAGTTAACGATAGAAAAGAGGCGCAGGA?????????????????CTCAACAAACTTAGCTCATGAGGAGACGACCGTTCC?GACATGCGGTCGGCAACGTGTTGGGAAGTTCCGAGCGGCGGCGATCGGGCGTCGAGTCCCCCTGTCGGGGCTTCATTATCGCGACACC?CCGGTC?TCGTCGCCCGGTTCCCTGCGTGGAGCTGCGTAACATCTGCAGACACTCGGATTAACAGCGTTTAGAGGCGGATGGGGCGCGAGATCG?CTCGTGGGTGCGG????GTCGTCCGGCGTTCGAGATCTTCCGGG??GGCGGCGCGTGCACTTCCGCGAGGGGTCCACGACGGTTCGAGTCGTCAGAGCCCGCAGGGAGGACCGCGGCGATCGGAGTTATAACCTGC?CGTGGCGGGCCGACTTGTGGACCAGATC?GACTTCGCCGGGCTTCGCCCCCTG?TCGGGAGTTCGTCGG?AGGACTGCGCATGCTTTT????CGCGCCGGGACGGGC?ATGAGGGTCAGTGGCCTCCGTCGGTAGCCCATCAAGCTAAATTGCGCACGGGTTCTACGAAACCTAAGGCATGAGTGAAGGTTTGTC?TCTAGACCGAGTAGTCCCGCTCGCGGGCCTACGCCGCTCGATCCCCGTGGACGAGGCGGACAACGTACACTTGGGTACCTATACACAAGCTGGTCTAGACAACTTGGTTAATTCCTGCGCTCA?AGAGTATCGTATCTGACGAACACATTAAGTCCGTGTCGATTACCGGACGATCGAGTGCCGCCCTACTCCTACAACGATGATCTCATAATAATTTGTGAATACAACGCCGATCCGTGTCACTAGGGTCGGGTAGGACGTCGGGCATCGGGGTGCCTCCGCGACGGGAAGTCCGCGGCGCCGGCCTTGGCTGGCTGGCGCGCGCGGTCATACGGATATGTTGAACGTAGATAGAAACTCGTTCGGAAGCGGGGCAATGA???TGCCCGTTAATATATCGTATCGACCGGCTACGGAGAGGACGCTTCGGTGTCGAGTGTACAACGAACCAGAGCTCGGCGAGGCCCCGGACTTGAGACGGACTCTGTCGGCTTGGCGATGACGTCGTTTCCTACCGCCCCTCGGGCGTGCGCTCTCGTCGGCTAATCTGGGGACGGTGTGAATCCGTGCCGGGCTGGACCAT?CCTAACAGATTGTCATCGAGAGGCTGCCGTCTGGGGTACGAGCGGTGCGTGACCGGCAGG?GCTGGTGAGATCGCAAGGTCGACCCGC?ATTGTTGGAAACTTCCCGTGGACTGCTCACTAGCGTCGGTCTGGGCCGCCGCGTCGGCCGGATGTAAAGCCAATTTAACATATGCTGCGTTGCTGGTGTGATTAAAATCCGGATCTAACGCAGAATCAGCTATCTGCCGACTTTTAGAGAATGAGAGCACGGGAAATTTTTTACGTTTCTTATTTAGAAGCGGAGATCGGGCTTGCCCTATCCTGGAACGTCCGGCTCGGTCGGGCGCGTCCCTCTAAAGTGTCAGCGGATAGAGCTTGAAGCAGGCGCTACACATCACTTTTTCGAAGTGTTAACTATTTATTAAGGATTACAGCCTCTGATCGTCGTTAATCCCTCCCGTACATTTATTCTTGCTATAACAATTGAATTAGGATTGA?

ALCIO_Alciopina

??????????????????????????????????????????????????????????????????????????????????????????????????????????????????????????????????????????????????????????????????????????????????????????????????????????????????????????????????????????????????????????????????????????????????????????????????????????????????????????????????????????????????????????????????????????????????????????????????????????????????????????????????????????????????????????????????????????????????????????????????????????????????????????????????????????????????????????????????????????????????????????????????????????????????????????????????????????????????????????????????????????????????????????????????????TTCACGGGTGCTACTTCCTTTGCCCGGTGCCACCAGTACTATGCCATCTTCTCTCTTCCGCAAATCTTTGCGACTTGCCGTGTGTACGTTCAAGAATCGCCTGCTCCCCTACCTGTGGGCGACGTCTGCCGGCTCCACGAGCCCGACAGCGAATCCCTTCAATACAAGAGAATCCACTCACGGACTCAACCGACACCTGTTCGTGACTCGGCATACCCTGAGCCATCCAAAAATGGGCTGTACAAGGGATGGACCACCTCTGGAAGGTCGCTCCATTGACCAGCAAGCCCATGACAAGCCCGCTCCGCCGTACGTGGATTAACTTTCTTGTAGAGACGCTTATGGAAATGCGCTGTCAACGGACCAAAGTGAGTTACAGCTCCGAGAAGCTGGCTTCCACCCGTAACGCCGGAGGCGTCTAAACATGCTCTGCACCGAATCCTGCAAGGAGACCGATACGTCAACGTGCCACCGCCAAGAGACCGTAAGCCAACCGGTTCCAGCTTCCGGCAATGAACGATGGAGGATCTCTCGTTCTGTGAAGCTGAGATAACCGAGAAGGGGTCCACTTGCTCGGTGAGAAAGCAAGCGCT??????????????????????????????????????????????????????????????????????????????????????????????????????????????????????????????????????????????????????????????????TGGTTTCCTCTTATGCTGCTAATACATACTTTTACATAGTAACGTTAATAGTTATGGTCCTTAATCGTACATCCTACTTTTGCAACAAACAGCTCCGACCTTCGGGGAAGAGCCTTAGATCAGACAATACGGGCCTTTGCTGGCTTTGTGTTCAGCTCCGCCGGAGTATTTCAATTCTCCTCGTCGATGGTACGTGCTATCCTACCATGTTTAAAGCCAAAACTACGCCCTGGCAGGAAAACATACGGGACTCTTTCGCCCGTATTAACTTTTCCTTTAAGTTTAGTTGGACTGTCTCAGGTCCCGGCCGCGTTCACTTCACGGGTACTGCGTCCGGACCTCCCGCCGGTTCCCTTGGGCTCATTAGTTCTGGGTGCCCGGAACGTAATTATCAAAAGGCTTGCCTGAATAATGGTAGGTGCCTCGGTTCTATGTGTTTTTCGGAACCGAGGTAAGGGAGGCCGGTCGTTACGGTTTTGTCGCCTAGAATACTAATCGAATTTTAAAAACATCTGCACGATGACTGAGATCCCCGGATTGTTTCCTGACCCGGCTGCATCCTCTGACTAGTCATGACCATAGTGATTAGGGTCCCCCCGCCTATTGTTGGGGCTCTAGCATCTTCACCGTGTCGGTGCAACAAGTGCGTTTCACGGTTTCGCCAAGGAATTCGTT?TTGATAGTCCACGGCCGTCCGTTACCTCCTTCTATTGGGCTTTTCCCATGGACAGGGATCGTCCTTTTAATGGTTTGTGACCTTCGTTGGTCCCGCGGGGGCAACCACCTCGTGCGCGAGGATGTTCACTTGATCATTAAAA??????????????????????????????????????????????????????????????????????????????????????????????????????????????????????????????????????????????????????????????????????????????????????????????????????????????????????????????????????????????????????????????????????????????????????CCCCACAAACTAAGCYTATGCGGAGACGACCGCCCCTGTCGAGGCAGTGGGACCGTGTCAGGACAGCCTTTGGGGTTCCGCTGTCGCGTCCGGTCCTCCTGTCGGGGCTTCATACTCGTCACGGCGCGCGGCGGAGTCTCTTGAGCCCTAGCGTGGAGCAGTGTAACATCTGCCGACACTCGGATTAACAGCGTTTAGAGGCGGATAGGACGCTCAGCTGGCTCGTGGATACTGGCGCC???GTGGTGTGCTGGGATCCTCCCTCGGGGCGGTAGGTGCACTTCCGCGAGGTATCCACGACGGTTCCGGCGGTCAAAGCCCCTTAGAAGAGCTCCTGCTGGGAGTGTTATAGCTTTGGTGGCGTTGGCCCGCTAGCGGACCAGACACGTGCCGGCCCGGCTCGGCCCGTTTCGCGACGTCTCGACTGGAGGACTGTACATGCTCCGACCGCGGAAGGTGTGGGACACTCAGGGTCTGTGGCCTCGGTCGGCCCCCTATCAAGCTAAATTGCGCATTGGCTCTACGAAACCTAAAGTATGAGTAAAGGGCTTCCGTTCCGTCCTAGTAGTCCCGCCTCTGGGCCTATGCCGCTCGTCTCCTGTGGGTGAGGCGGACAACGTACACTTGGGTACCTATACACAAGCTGGTCTAGACAACTTGGTTAACTCCTGCACTCTTAGAGTATCGTATATGACGAACACATTAAGTCTGTGTCGATTACCAGGCGATCGAGTGCCGCCCTACCCCTATTACGATGATCTCATAATAATTTGTGAATACAACGCCGATCCGTGTCACTAGGGTCGGATAGGGCGTCGGGCATCGGGGCCCCTCCGCGACGGGAGGCCTGCGGAACAGGCCTTGGCTAGCTGGGGCGCGCGGTCATACGGATGCGTTGAACATAGATAGAAACTCGTTCCGAAACGGGGCAATGTCGTGCCCGTATCCTATATCGTATCGACCGGACACGGAGACGCCCTTTCGGGGGCTAATGTACAACGAACCAGAGCTCGGCGGGACCCCGGACTTGAGGCGGACTCTGTCGGCTTGCCGATGACGTTGTCGCCTACCGCTCCCCGGGCGTGCACTCCCGTCGGCTAATCTGGGGATGGTTTGAATTCGTGCCGGCCTGGACCGC?GATAACAGATTATCATCGAGAGGCTGCCGTCTGGGGCACGAGCGGCGTGGAACCGGCAGGGGCTGGCGAGGCCTCGGGGCCGACTCGCCGTCGCCGGGACCTTCCCGTGGGCTGCTCACTGGCGGCGTTCGCGCGTTTTGCTTCGGCCGGATCTAAAGCCGATTTAACAAATGCTGCGTAGCTGGTGTGATTAAAATCCGGATCTAACGCAAAATTAGCTATCTGCCGACTTTTAGAGAATGAGGGCACGGGCAATTTTTTACGTTTCTTATTTATAAGCGGAGAGCGGGCAAGCCCTGACCTAGAAGGCTCCGATCGGCCGGGCCCGTCCCTCTAAAGTGTCAGCGGATAGAGCTTGAAGCAGGCGCTACACATCACTTTTTCGAAGTGTTAACTATTTATTAAGGATTATAACCCTTGATCATCGTTAATCCCTCCCGTACATTTATTCTTGCTATAACAATTGGATTACGATTGAC

**ALVIN**_*Paralvinella*

?????????????????????????????????????????????????????????????????????????????????????????????????????????????????????????????????????????????????????????????????????????????????????????????????????????????????????????????????????????????????????????????????????????????????????????????????????????????????????????????????????????????????????????????????????????????????????????????????????????????????????????????????????????????????????????????????????????????????????????????????????????????????????????????????????????????????????????????????????????????????????????????????????????????????????????????????????????????????????????????????????????????????????????AAGGTGGCCCGTCGTTACGGGTACCGCTTCCTCTGCTTGATGCAAAGACTACTACGATGTATTTAATCTTCCGTCAAGTCTTGCAATTTGCTGTGTGTATATTCGAGAATAGCCTGCACGTGTTTCCTGGAGCGGTGTGCGCCGGTTCAATGAAACCAACAGTGAGTCCCTACTTTGCAAGAAAATCTGCCCATGAATGCAACAGACACTACCTTGTGAATCGGCATTCCATGAAGCTAGCAGCAATGCCATGTTCAAGAACTGGACTACCTCTGGAAGCCTGTGCCATTGATGCACAGCAGACTGACAAACCCGCTCTTAGGTCCGTGGCTTAAGATGCTTGTTGTCTGACATATGGTCACATGAAGTGAACAAACTTGCATGGGTCAATGCTCAGAGAAGCCGAAAACTTCCCGGAACGCCTATACCGCCTTAACCTGTGGTGTAGCGAATTCACCAAGGCCATAGAAAAATCAAGGTGGCATCCCTACCAGACCAGGCAATGCATGGTTCCTGACTCCAGAGCTGAGCCCTGAAGGATTCCTCGTAGTGTGGAGTTGAGACAACCCCTCATGGATCTTTGTGCCACGCGAGAGTCCATCAGCGAGTTGCAGCGTTGCCGATATCCTT??????????????????????????????????????????????????????????????????????????????????????????????????????????????????????????????????????????????????????ATGCTGCTAATACAAGCTCGAACTTGGTAACGTTAATAGTTGATATTATTAATCGTACCAGTTACTTTTGTAAACGCATGCTCCGACCCGAGGGGAAGAGTCTTAGATCAGACAACAGAGGTCATTGATGGCTTTGGGTTCAGCTCAGCCGGAGTATTTCAATTCTCCTCGTCGATGGTACGTGATATCCTACTATGTTAGGGGCCAAAACTATGCCCTGGCAGAGACACATACGGGACTCTATCGCCCGTATTAACTTTTCCTTTAAGTCTAGTTAGACTGTCTCGGGTCCAGGCCGCGTTCTGCTCATTGAAACTGCGTCCTGACCCCTCGCCGGTTCCCTTGGGCCAACTGGTTCTGGGTGGCCGGAACGTAATTATCAAAAGTGTCGCCTGAATAATGGTAAGTGCCTCGGGTCTATGTGTTTTTCGGAAACGAGGTAAGGGAGGACGGTCGTTACGGTTTTGTCGCCTAGAATACTAATCGGATTTTAAGAACATGTGCACGACAACTAAGATCCCCGGTTTGTTTCTTGACTCGGCGGCATCTTCCGACTAGTCATGGCCATAGTGATTAGAATCCCCCCGCCTATTGGTGGGGCTCTAGCATATTCGCCGTGTCGGCGCAACAAGTGCTTTTCACGGTTCCGCCAAGGGATGCGTTGTCTCTGGCCCGAGGTCGTCCGTTACCTCCTTCTATTGGGCTTTTCCCATGGACAGGGATAGTCCTTTTGATGGTTTGTAACCCTCGTCGGTCCCTCGGAGGCAACTGCCGCGGTGGCGAGGACGGTTGCTGTATCATCA????????????????????????????????????????????????????????????????????????????????????????????????????????????????????????????????????????????????????????????????????????????????????????????????????????????????????????????????????????????????????????????????????????????????????????????????????????????????????????????????????????????????????????????????????????????????????????????????????????????????????????????????????????????????????????????????????????????????TTTAACACCGTTTAGAGGTGGATGGGACGCAC?GGCTCCCCTCTGGCACCCGCGTCCCG?????GTGTCCCGATC?TCCGGGGGGCCGAGCGGCGCACTCCTGAGGGCTCTCCGGGACGGTTCTGGCGGCGAAAACCGAGCCCAAGAACTCGGGGGGGAGCGTTTAGGGGCTCT?CGGCGGCGGCCGCCCGGGGGACCAGACG????ACCGCCGGGCGCGGGCCCCCCGTGGCTCGTTCGACTGGTGGACTGTGCATGCTCCGACTGCCGA?GTGGGGGGGCACTCAGGGTCTCCGGCCTCTGTCGGAACCCCATCAAGCTAAATTGTGCATGGGTGG???CAAACCCAAAGCATGAGTGAAGGCCGGCTC???CGGCCCAGTGGTCCCTCCTGGGGGCCCATGCCGCTCGGTCTCCGTAGACGAGGCGGACAACATACACTTGGGTATCTATACACAAGCTGGTCTAGACAACTTGGTTGACTCCTGCACTCTTCGAGTATCGTATATGACGAACACATTAAGTCCGTGTCGGTTATCGGGCGATCGAGGGCCGCCCTACTCCTACTGCGAGGATCTGATAATAATTTGTGAATACAACGCCGATTCGTGTCACCAGGGTCGGGTAGGACGTCGGGCACCGGGGTGCCCCCCCGACGGGAGGTCCGCGGAGCAGGCTTCGGCCAGCCGGGGCGCGCGGTCATACGGATATGTTGAACGCAGATAGAAACTCGTTCCGAAACGGGGGAATGGC??GCCCTTC?CCTATATCGTATCGACCGGACGTGGAGATGATCCCTCGGGGTCTAGTGCGCAACGAACCAGAGCTCGGCGGGACCCCGGACTTGAGGCGGACTCTGTCGGCTTGCCGATGACGTTGTTCCCTACCGCTCCCCGGGCGTGCGCTCCCGTCGGCTAATCTGGGGACAGTGTGATTTCACGCCGGCCTGGACCAT?GATAACAGATCGTCATCGAGAGGTCGCCGTCTGGGGTCCGAGCGGCGTGGAACCGGCAGGGGCTGGTGAGACCTCGCGGTCGACTCGCCGTCGCCGGAACCTTCCCGTGGACTGCTCACTGGCGGCGTTCGCGGCGTCCGCTTCGGCCGGGCGTAAGACCGATTTAACAAATGCTGCGTGGCCGGTGTGATTAAAATCCGGATCTAACGCAGAATCAGCTATCTGCCGACTCTTAGAGAATGAGAGCAC?GGAAGTTTTTTACGTTTCTTATTTATGAACGGAGGGCGGGTGCGCCCTCGACTGGAACGCCCGACTCGGCCGGGCGCGTCCCTCTAAGGTGTCAGCGGATAGAGCTTGAAGTAGGCACTACGCATCATTTTTTCGAAGTGTTAACTATTTATTAAGGATTATAACCCTTGATCGTCGTTAATCCCTCCCGTACATTCATTCTTGCTATAACAATTGGATTAGGATTGAC

**AMPHA**_*Ampharete-Auchenoplax-Isolda*

????????????????????????????????????????GGACAACTCATTTTAGGAAGAGACCATATCACAATGTTCCCCATCTAATCTCTTACGTTTTTACAATCGACACGCATCTATAGGGTCTCATAGATTCATCACTCAAATGTACCCTCAGTCTACCCGCTTAAGATCAGCAGCAGAGAAAAAAAAAGAGACCTTAGCAGGAACATTGCTTAGAACCATCAGATTTCTTTTCTCCAGCAATTTCAATCAGAGCATATTATACTTATCATACAAAATACGAACTAGAAAATATTCCTATGTAAGCTGTTTTTGAAGTAATCTCTCACCTCTCAGGTAAGACAACCGCTCTCACGAACATATATCTTTTCCTTCTAAATTATCAATCATCATATAACCTTATAGAAAATGATTGACTAGAATAATTTGTAAAACAGGCAACAGACCGTTTGTCAAAAAAAGTTTGATGTCCCAGTTGAAGCACAATATTTCTCTGTAAACTAAATGTCATCACGCCTTTACGCATCATACTCTTAACACTTTTATAAGAAAAC????????????????????????????????????????????????????????????????????????????????????????????????????????????????????????ATCACGGGTGCTGCTTCCTTTGCTCCGTGCCGGCAGTACTATGGCATCTTTAATCTTCCGCTTAATCTTGTGACTTGCTGTGTGTGTGTTCGAGAATAATCTGTTCGCGTTCACTTGGGTGACGAGAGGCGGTTCAATGAGACCTACAGCGAGGCTCTTTGGCGCCAGAGAGTCGACTCACGGATTTAACAGATGGTACATTGTTACGAGGCATACCAGGAGCCTAGCAGCAATGTCCTGTTCAAGGGATGGAAAATCGCTGGAGGCTCTCCAGATCGATCAAGAAGAACCAGACAAACCCTCTCTGCCGTGCGTGGATTAGGCTCCTGGCCGTCACGCAAACCATCACATGTAGTGGGTCGACCTGCGTGTGTTCAGGCTCTGACAAACCGAACTTCTCTCGGAACACGCGCACCGTCTCTATTGCTGGGGTAGCGAATCCACCAAGGAAACCGCCAAATCAAGGCGCTACCGTTATCAGATCAGGCTATGCGAGGACCCTGCTTTCAGTGCTGAGCGCTTGAGGATTTCTCGTAGTCCGAAGCTCGGACAATTCCCCATGTATCTTTGTGCAACATGACAATACATCCGCAAGCTGCAGCTTCTCAGAGACCCATCGA??????CATGTAAGGGCGTCGCTCTGAGTCAGCTTATACTCTGATACTCCTGCTCTCAGTCATGCCCGCCGCCAGCTCTTCTACAGCGTCCTCTCGTCTAATTCCGGTAGCGGTCCTTTCTCGCCCT????????????????????ATGCTGGTAATACGAGCTCTTAGACGGCAACGTTGATAGTTAATATTATTAATCGTACCCGCTACTTTTGTAACGAGAAGCTCCGACCCCGGGGAAAGAGCCTTAGATCAGACAACGGCGCCCCCTGCTGGCTTTGGGTTCAGCTCAGCCGGAGTATTTCAATTCTCCTGTTCGATGGTAGGCGACATCCTACCATCTTAAAGGCCAAAACTATGCCCTGGCAGAGAAACATACGAGACTCTATCGCTCGTATTAACCTTTCCTTTAAGTCTAGTTAGACTGTCTCGGGTGCAGGCGGCGTTCCGCTC?TCGGAACTGCGTCCTGACCCCTGGTCGGTCCTCTTGGGCCGACTGGTTCTGAGTGGCCGGAACGTAATTATCAAAAGAGCTGCCTGAATAATGTTAGGTGCCTCGGTTCTATTTGTTTTTCGGAATCGAGGTAAGGGAGGACGGTCGTTACGGTTTTGTCGCCTAGAATACTAATCGAATTTTAAAAACATGTGCACGACGACTACGATCCCCGGCTTGTCTCATGACTCGGCGGCAGCTTCCGACAAGTCATGGCCATAGTGATTAGAATCCCCCCGCCTTTTGGTGGGGCTCTAGCATATTCGCCGTGTCGTCGTTACAAGTGCGTTTCACGGTTTCGCCAAGGAATACGTTGTGCCTGGCCCGAGGTCGTCCGTTACCTCCTTCTATTGGGCTTGTCCCATGGACAGGGATAGTCCTTTTGATGGTTTGTGACTCTCGTCGGACCCGGCGGGGCGACCGTGGCGGTGGCGAGGACAGTCGCTGTATCATCA??????????????????GGAATTCCCCTTGTGGGCTTTCGTGGTTACGAGGGTTTAGCGCCTTTTAATACTGGATAATCTTTAGGAAGGCCTGAAAAAGTCGCAGAACATAGGCTTTGTTTTATTAATATAAAAAATTTATTTA???TAAAACTAACGCGGTTGAGGGTATCTACTCTTCTTTTTTCTCTTCTATAAAACTGTCATAGATGGAAAAGCCTTGGACTA?????????????????????????????????????????????????????CTTCACAAACTAAGCCCATGCGGATACGGCCGCCCTCGGGCTCCCGACGGGAACGCGTTGGGAGGTCGCCGGGTCGTCGTCTGCC??GTCCGGTCCTCCTGTCGGGGTTACAGACTCGTCACGTAGCG?GGCGACGGCCCCCGGCCCCCCGCGTGGAGCAGCGTAACATCTGCAGACACTCGGATTAACAGCGTTTAGAGGTGGATGGGACGCTA?GGCTCCCCGCGGGTACCCCTGTCGCGT?GC?GTCTCCGGATCCTTCGGTCGGGCGTGGGGCGCACTTCCGCGGGCGTGCCAGGAAGGCCTGGGCGGCCAAAGCCCGGAGTCAGAGCCACTCGGGCGGTGCTTAGTCTCCCC???GGGCGGGCAGCCCGTGGAGCCAGACG????ACCGCCGCCCGGGAACCGGCCGCGTCGGGTCAGACTGGTGGACTGTGCATGCTCCGACCGCCGC?GTGCTCGGGCGCTCAGGGTCTCTGGTCTTCGTCGGCACCCCATCAAGCTAAATTGCGCACTGGCGG???CAAACCCACGGCATGGGTGAAGGGCGACCTA??CGTCCCAGTGGTCCCGCCGGCGGGCCCACGCCGCTCGATCCCCGCGGATGAGGCGGACAACGCACACTTGGGTACCTATACACAAGCTGGTCTAGACAACTTGGTTAACTCCTGGACTCGTAGAGTATCGTATATGACGAACACATTAAGTCCGTGTCGGTTATCGGGCAATCGAGCCCTGCCCTACTCCTACCACGAGGATCTCATAATAATTCGTGAATACAACGCCGATCCGTGTCACTGGGGTCGGGTAGGACGTTGCACAAGGGGGAGTCCTGCCGACGGAAGGTCCGCGGAACGGGCCTAGGCTGGCCGGGGCGCGCGGTCATACGGATATGTTAAACGCAGATAGAAACTTGTTCCGAAACGGGGCATTCAT??GCCCTCC?CTCATATCGTATCGACCGGACGTGGAGATGGCCCCTCGCGGTCCAGTGTGCAACGAACCAGAGCTCGGCGGGACCCCGGACTTGAGGCGGACTCTGTCGGCTTGTCGATGACGTTGTTCCCTACCGCTCCCCGGGCGTGCGCTCTCGTCGGCTAATCTGGGGACAGTGTGATTTCACGCCGGGCTGGACCAT?GATAACAGACTATCATCGAGAGGTCGCCGTCTGGGGTCCGAGCGGCCTGGAACCGGCAGGGGCTGGTGAGGCCGCGAGGTCGACTCGCCGTCGCCGGGACCTTCCCGTGGACTGCTCACTGGCGGCGGTCGCGCCGTTCGCTTCGGCCGGGCTCAAGACCGATTTAACGAATGCTGCGCAGCCGGTGTGATTAAAATCCGGATCTAACGTAGAATCAGCTATCTGCCGACTCTTAGAGAATGAGAGCACGGGAAATTTTTTACGTTTCTTATTTATGAACGGAGGTCGGCTAGGGCCCTGCCTGGAACGCGACGCTCGGCGCCGCGCGTCCCTCTAAGGTGTCAGCGGATAGAGCTTGAGGCAGGCGCTGCGCATCATTTTTCCGAAGTGTTAACTATTTATTAAGGATTATAACCCTTGATCGTCGTCAATCCCTCCCGTGCATTTACTCTTGCTATAACAATTGGATCAGGATTGAC

**AMPHI**_*Chloeia-Eurythoe-Paramphinome*

CGAGTTAGCAGACACCGAACCTCAAAGATTTATCACAGCCGGGCGCAGCTTTTCAGGAAGAGACCACTCTACCACGTCCCCCATTCTACCTCAAATATAATAATCATTGCCACGGAACTATAGCGGCACTTAGATTTTACATTAAAATACACTCCCCTTCAACACACTGATTTTCAGCCGCAGAGAAAAAGAACACGACTATATCAGAAATCTCGCCTGCCACCCTCCGACCGCCTCGTACCGGGAGTGTCAATTGCCTCCTACTACACAAACCATTCGGGAATACAAATAGAACGGGTACACGTATTATCTATTCTAATAACTATTACGTACATCCCCGTCCCGACAATAACACTCGCGAACTAATCGCGCTCCCCGCAAAACAGGCCACCGCTACGTCACCTCATAGAAAACGATCTCTCCGATTAACTTGCCTAATAGATAGTGGAAACCACAGCAAAAGTAATACGACACCCCGATGCCAGCTTAACATTCTACCCTTCACACAACACCCTCACTCCTATATATTATACACCCCTCACACCTCCTCTAGAAGCTGATGCAGACCAGATTAACCATGGACTTAAAACACTTAGTATTACCCCACGATGATCAGCCGCAATTTCCTTACACAACCTCCCCCAACCCAACAAGC??????????CAGGCGGCCCGTCGTCACGGGCGCCGAGTCCCTCTCTCGGTAGTACCACTATTATACCATCTTCAATCTTCCGCAATATCTTGCGGATTGCCGTACATACGGCCTAGACCGGCCAGCACTCTCTCCCGTGGACGATGTCCGCCGGCAGCATCAGAAAATCTCACAAGACCCTCGAAACAAGAGAAAGCACACACGGACTCAATAGCCACTGGCTTGTACCCAGGCATACCACGAAGCTAGCGACAATGACCTGTTCAAAGGATGGGTTAGCGTTGGCGGCTCGCAGTATTAAGCAAAGAACAACAGGCAAACTCGCATTGACGCACACTCTTTCGGCGCCTGGCAACCAGACAAAACATTACACGCCACTAATGCACTTGCGCATGTCAGCACCTGACTGAGCTCTGCTCTTCCCGGAACGCCCACGGAAACTAAACATGTGGTGCAGCAGACTCTCTAAGGAGGCCGCCACCTCCACGTGACACCGCCCTGAGACCACGCTCCGCCAGGCTCTCGTCTCCAGCACCGAAATAAGGAGGAACTCTCGTTCAGCGAAGCTGAGAGAACCAAGTTTGCATCAACGCGCCAGGCATGAACCCATCCGCCATTGTGAGCTCCTCCGATATCCACCGTCG????TTGGCCAGGGCGCCGCAAAGAGTAAGCCTTGACTAGGCTCCTTCTGCTCTCGGAGCCGCGTGCCGCGAGCCCTGCTATTGCGTCCGCCCGAGCCACTCGAGCAGCAGCCCGCACTCGCCCT????????CTGTTTCCTCTTATGCTGCTAATACAAACTTTTACACAGTAACGTTAATAGTTATGGTCCTTAATCGTACATCCTACTTTTGTAACAACAAGCTCCGACCTTCGGGGAAGAGCCTTAGATCAAACAATCGGGTCCTTTGCTGGCCTTGGGTTCAGCTCAGCCGGAGTATTTCAATTCTCCTCTTCGTTGGTACGTGATATCCTACCAAGTTTAAGGCCAAAACTACGCCCTGGCAGGAAAACATACGGGACTCTTTCGCCCGTATTAACTTTTCCTTTAAGTTTAGTTAGACTGTCTCGGGTCCAGGCTGCGTCCGCCTCGCGGGTACTGCGTCCTGACCTCCTCCCGGTTCCCTTGGGCTGACTAGTTCTGGGTGGCCGGAACGTAATTATCAAGAGATTTGCCTGAATAATGGTAAGTGCCTCGGTTCTATTTGTTTTTCGGAACTGAGGTAAGGGAGGATGGTCGTTACGGTTTTGTCGCCTAGAAGACTAATCAAATTTTAAGAACATGTGCACGACAACTACGATCCCCGGATTGTTTCATGACTCGGCGGCAGCTTCCGACAAGTTTTGGCCATAGTGATTAGAATCCCCCCGCTTATTGGTGGGGCTCTAGCATATTCGCCCAGTCGGCGTAACACGTGCGTTTCACAGTTTCGCCAAGGAATGCGTCGTCACTGGTCCGAGGTTGTCCGTTACCTCCTTCTATTGGGCTTCTCCCATGGACAGGGATAGTCCTTTTAATGGTTTGTGATCCTCGTTGGTCCCGTGGGGGCAACCGCCGCGGTGGCGAGGACGATCACTTGATCATTAAAA???????????????AAAAGTTCCTCTAGAGGCTAATCCAACTACGCGAGCCCCCCGCCTTATGCCCCCAACTAACCTTTAGGAATGCCTAAAACAACTAATAAGTATCGGCTTCATTGCAAATTAGAGGCCAACCCCCCCCTTCCGCAATTTGTGCAACAAAGGCATCTAAACCTTCCTTAAAACCTTTAACAA???CGGCGATCTTATAAATGCCGTAAGTTATTTTTCTCGAGCCACTAACAGAAAAGT??????????????????????????CTCCACAAACTCAGCCTATGCGGAGACGACCGCCCTCACCGCGGTGGCGGGAACGTGTCAGGACAGCCTATGGGGCTGCGCACCGGTGTCCAGTCCTCCTGTCGGGGCTACAGGTTCGTTACGGCACTGGTCGCAGTCTCTAGAGTCCTAGCGTAGAGCAGAGTAACATCTGCTGACACTCGGATTAACAGCGTTTAGAGGCGGATGGGACGCAAAGTCGACCCGGGGACACTTGCGTGCGTGGAGTGCAAC?GGATCCGTCCCGTGTGCGGTGGGCGCACTTCCCCGGGGAGTCCACGACGGTTGGGATGGTCAAAGCCCGGAGGAAGAGCTGCGGCCGGTAGTGTTATAGCCTCCGACGCGTTGGCCCACTGCCGGACCAGACTCGTGCCCGCCCGGCTCGGTGGGCTCTTCTGCTGTTCGACTCGTGGACTTTTAATGCTTGGACTGCGGGCGGGACCCGGCACTAAGGGTCAGTGGCGTTAGTCGGTTTCCTTTCAAGCTAAATTGCGCATGGGCTCTACGAAACCTAAAGCATGGGTAAAGGCCTGGCACGAAGGCTTAGTAGTCCCTCATGGGGGCCTACGCCGCTCGTCTGCTGTCAGTGAGGCGGACAACGTACACTTGGGTACCTATACATAAGCTAGTCTAGACAACTTTGTTAACTCYTGCGCTTGTCGAGTATCGTACCTGCCGAACACATTAAGTTTGTGTCAATTATCAGAC?ATCGAGTGCTGCCCTACCCCTACCACGAGGATCTCAGAATAATTTGTGAATACAACGCCGATCCGTGTCACTAGGGTCGGGTAGGRCGTCGGGCATCGGGGTGCCTCCGCGACRGGAGGCCCGTGGAACTGGCCTAGGCCAGCCGGGGCGCACGGTCATACGGATACGTTGAACCTAGATAGAAACTCGTTCCGAAGAGGGGCACTGCCAAGCCCATTGCCTATATCGTATCGACTGGACACGGAGATGGCCCCTCGGGGTCCAGTGCACAACGAACTAGAGCTCGGTGGGATCCCGGACTTGAGGCGGACTCTGTCGGTTTGCCGATGACGTTGTTCCCTACCGGCCTTCGGGCGTGCACTCCCATCGGCTAATCTAGGGACGGTGTGATTTCGTGCCGGAATGGACCGTTG?TAACAGATTATCATCGAGAGGCTGCCGTCTGGGGTACGAGCGGCCTGGARCTGACAGGGGCTGGCGAGGCCGCAAGGYCGACTCGCCGTCGTCGGGACCTTCCCGTGGACTGCTCACTGGCGGCGGTTCGGCTGTTAGCTTCGTCCGGATCCAAAGCCAATTTAACATATGCTGCGTTGCTGGTGTGATTAAAATCCGGATCTAACGTAAAATTAGCTATCTGCCGACTTTTAGAGAATGAGAGCACGGGCAGTTTTTTACGTTTCTTATTTAGAAGCGGGGAGCGGGCAAGCCCTCGACTAGAACCGACGGCTTGGCCGCCGGCGTCCCTCTAAAGTGTCAGCGGATAGAGCTTGAAGCAGACGCTACACATCACTTTTTCGAAGTGTTAACTATTTATTAAGGATTATAACCTTTGATCGTCGTTAATCCCTCCCGT??????????????????????????????????????

**APHRO**_*Aphrodita*

?????????????????????????????????????????????????????????????????????????????????????????????????????????????????????????????????????????????????????????????????????????????????????????????????????????????????????????????????????????????????????????????????????????????????????????????????????????????????????????????????????????????????????????????????????????????????????????????????????????????????????????????????????????????????????????????????????????????????????????????????????????????????????????????????????????????????????????????????????????????????????????????????????????????????????????????????????????????????????????????????????????????????????????CAATGAAGCTAACGTCACGGGTGCTACTTCCTTTGCTCCGTGCCAACGATATTACGCTATTTTCAATCTTCCACTACCTCTCACCACATGCCGTGTAAGTGTTTCAGAACCGCCTATTCCCCTACCCTTGGGCCATATGCACCGGCAGCACGAGCCCAACAGCGATTCCCTACCAGACGTAAGGGTCTGCTCACGGATTCAACAGACAGTGCATCGTACCTCGGCATCCCTGGAGACATCCAAGAATGAGCTGTTCAAGGGATGGAACACCGCTCGAGGCCCTTTGCATCGAGCCAAAAACCACTGACAAGCACGCTCTCCCGCACGTGGTCTAGGCCCCTCGTTATTCCGCTCATGCCAACCTGTCTGCAACCGACCTGAGTGGGCCACTGCACCGTAGAGCTACCTACCTCCCGCAACGCGCGTGCCGTCTAAACCGCCGGTGCAACGAACTCCCCAAGGAGACCCACGAGTCCGTGTGACACCGCCAACAGACGGCGCCACGCTATGCCCCAGTCTTCAGCAAGGAGCGATGGAGGATCTCTCGTACCGCGAAGCTTGGACAACTCCATTCGCATCTTCGCGCCATGTGAGAGTCCCCAGGCACCTGTCGGCCTCTCACAGACCCAACCTCG??????????????????????????????????????????????????????????????????????????????????????????????????????????????????????????????????????CTGTTCCTCTTATGCTGGTAATACAAGCTATCAGACGGTAATGTTGATAGTTATGGTCCTTAATCGTACCTCCTACTCTTGCAACGACCGTGTCCAACCCCGGGGAAAGACTCTTAGATCAAACAATCGGGGCCCCTGCTGGCTTTGTGTTCAGCCGAGCCGGAGTATTTCAGTTCTCCTCGTCGATGGTACGCGACCTCCTACCATGTTAAAAGCCAAAACTACGCCCTGACAGGAAAACATACGGGACTCTTTCGCCCGTATTAACTTTTCCTTTAAGTCTAGTTAGACTGTCTCGGACTCGGGCGGCGTCCGCCTCGCGGGCACTGCCCCCGAGTCTCCTGCCGGCTCCCTTGGGCTGACTAGTTCTGGGGAGCCGGGACGTAATTATCAAGAGCGCAGCCTGAATAATGGTAGGTGCCCCGGTTCTATTTGCTTTCCGGAACCGAGGTGAGGGAGGACGGTCGTTACGGTTTTGTCATCTAGGATACTAATCGAACTTTAAAATCATCTGCCCGACGTCCACGATTGCCGGATTGTTTCATGACCCGGCTGCAGCTTCCGACAAGTCTTGGCCATAGTGATTAGGATCCCCCCGCCTTTTGGTGGGGCTCTGGCATATCCCGCCTGGTGGCGCGACAAGTTCGTATGACGGTTTCGCCAAGGAATACGTTGTCCTTGCTCCGAGGGCGTCCGTTACCTCCTTCTATTGGGCTTGTTCCATGGACAGGGTTCGACTTTTTAACGGTTTGTGATCCTCGCCGGCCCCTCGGGGGCAACCGCAGGGGTGGCGGGGACGATCACTTGATCGTTA?????????????????????????????????????????????????????????????????????????????????????????????????????????????????????????????????????????????????????????????????????????????????????????????????????????????????????????????????????????????????????????????????????????????????????????????ACAAACTCAGCTCATGCGGAGACGGCCGCCCCCGCTAGGGCGGCGGGAACGCGGAGGGACAGCCTTTGGGGCGGCGAGACTGCGTCCGGTCCTCCTGTCGGGGTTTCACACTCGTCACGGCGCGGATCGCCGCCTCTTGAGTCCTCGCGTGGAGCAGCGTAACATCTGCTGGCACACGGATTAACAGCGTTTAGAGGTGGATGGGACGCAAAGTCCGCGCGTGGATGCTAGGTCAGGTCGCGATCGAAGGGATCCTTCCTGTCGTCGTCGGGTGCCCTTCCGCGCGGAGCCCACGACGGTTCCGGCGGCGAAAGCCCGGAGCAACGGCGCAAGGGTGCGACGTTACAGGCTCCTTGGCCGAGGCCTGCCGAAGGACCAGACAGTGCCCGGCCGGGGTCGGATCCTTCTTG?????CCCGCCGTGCGGACTGTGCATGCGGTTTCGGTAAAAGGGCGGTCCGACTCAGGGTCGGTGGCGTCGATCGGCACCCCATCAAGCTAAACCGCGAATGGCGGTCACGAAACCCAGAGCATGAGTGGAGGCCCTTCTCGGGGGCCGAGTTGTCCCTGGGCGGGGCCAGCGCCGCTCGCTCGTAGTCGGCGAGGCGGACTACGCACGCTTGGGTACTCACACATAGGCCAGTCTAGACGGCTTGATTGACTCCTGCGCTCTTCGAGCATCGTACCTGCCGAACACATTGAGCCGCTGTCAATTACGCGGAAATCGAGTAGAGCCCCGACGCCACCGCGCACATCCCGGAACGACTGACGGTTACGGCGCAGCACCGCGCCGTCTGCGTGGGGCGGTGCGTCGGCCATGATGAAACGAGCGCGACGGGAGGTCGGTGGTACGGGCCGTGACCAGTCGGGGCGCACCGTCATACGGATGCGTTGAACGTAGATAGAAGCTCGTTCCGAAACCGAGCAAATAAG?CTCGGTGATCCATATCGTATCGACAGGCCACGGAGACGCGCCTTTGGGGCTCGGTGCACAACGGACCAGTGCT?GGCGGCATCCCGGACTTGAGGCGCACTATGTCGACTCGCTGATTACGCTGCACCCTACCGCTCCCCGGGCGTGCACTGCCGTCGCCGAAACTGGCGAAGGTGTGATTTCGTGCCCGACTGGAC?GC?GATAACAGACAGTCATCGAGAGGCTGCCGTCTGGGGTGCGAGCGGCCTGGAACTGGCAGGGGCTGGCGAGGCA????TGTCGACTCGCCGTCGCCGGGACCTTCCCGTGGACCGTTCACTGGCGGCTGTCGATGAATCCGCTTCGTCCGGGTGCGAAGCCGATTTAACAAATGCTGCCTCGCCGGTGTGATTAAGACCCGGATCTAACGCACAATCAGCTATCTGCCGACTCTTAGAGAATGAGAGCACCGGGTCTTTTTGACGTTTCTTATTTAGAAGCGGAGAGCGGGCAAGGCCTCCTCTGGAACCGCAGCATCGGCCGGGCGCGTGCCTCTAAAGTGTCAGCGGATAGGGCTTGAAGCAAGTGCTACACATCACTTTTTCGCAGTGTTAACTACTTGTCAAGGATTATAACCTTTGATCGTTGTTAATCCCTCCCGTACATTTAGTCTTGCTATAACAATTGAATTGGGATTGAC

**APIST**_*Apistobranchus*

?????????????????????????????????????????????????????????????????????????????????????????????????????????????????????????????????????????????????????????????????????????????????????????????????????????????????????????????????????????????????????????????????????????????????????????????????????????????????????????????????????????????????????????????????????????????????????????????????????????????????????????????????????????????????????????????????????????????????????????????????????????????????????????????????????????????????????????????????????????????????????????????????????????????????????????????????????????????????????????????????????????????????????????????????????????????????????????????????????????????????????????????????????????????????????????????????????????????????????????????????????????????????????????????????????????????????????????????????????????????????????????????????????????????????????????????????????????????????????????????????????????????????????????????????????????????????????????????????????????????????????????????????????????????????????????????????????????????????????????????????????????????????????????????????????????????????????????????????????????????????????????????????????????????????????????????????????????????????????????????????????????????????????????????????????????????????????????????????????????????????????????????????????????????????????????????????????????????????????????????TTCCTCTTATGCTGCTCATACAAGCCTAAACACGGCCACGTTAATAGTTATGGTCATTAATCGTACCAGTTACTTTTACAACCTCAAGCTCCTACCCTCGGGGACGAGCCTTAGATCAGACAATGGCGGCCATTACTGGC?ATGGGTTCAGCTCCGCCGGAGTATTTCAATTCTCCTCGTCGAATGTTGACGATATCCCACATTGTTTAGGGCCAAAACTACGCCCCGGCAGGAAAACATACGGGACTCTTTCGTCCGTATAAACTTTTCCTTTAGGTCTAGTTAGACTGTCCCAGGTCCGGGCTGCGTCCGCCTCGCGGGTACTGCATCCCGACCCACCGCCGGTTCCCTTGGGCTCGCTAGTTCTGGGTAACCGGAACGTAATTATCAAAAGTACGGCCTGAATGATGTTAGGGGCCTCGGTTCTATGCGTTTTTCGGAACCGAGGTAAGGGAAGACGGTCGTTGCGGTTTT?TCGCCCAGGAGACTAATCGAATTTTAAGAACATGTGCACGACGACTGCGATCCCCGGATTGCTTCATGACTCGGCGGCAGTCCCCGACAAGTCTTGGCCATAGTGATTAGAATCCCCCCGCTAATTGGTAGAGCTCTAGCATATTCGCCGTGTCGGCGTAACAAGTGCGTTCCACGGTTTCGCCAAGGAATGCGTTCTCTGTGGCCCAGGGTCGTCCGTTACCTCCTTCTACCGGGCTTGTCCCGCGGACGGGGATAGTCCTCTTAATGGTTTGTGATCCTCGTTGGACCTGCGG??TTTACCGCCACGGCGGCGAGGACGATCACTTGATCATTAAAA???????????????AACAATATCATTAATGAATTTTGTGATAACGAAATGTCAACTATCTTTA?AAAGAAATAAAATTTTGGATTTCCAAAAAAAATTGAAAAGTGTCGACTTCACTGAAACTTT????????A??????????TAAAGTTAGTGCGACTAGAGTACCTAAAATTTCTCTATAAATAAGAACAAATCTGTCTGGAACCAACTAGTCGCAAGCCATTCCTCTCGAGCCAATGACAGAGGAG?????????????????????????????CAACAAACTCAGCTTATGCGGAGACGACCGCCCCCGCCTGGGCGGTGGGACCGTGTAAGGACGTTTTCTGGTCGGGGCGACGTATGCCCGGTCCTCCTGTAGGGGTTGCAGTCTAGTCCCGGTGTGCGCTCCCGTCCCTTGAGTCCTCCCGTCGAGCAGCGTAACATCTGCTGGCACTCAAATT????????????????????????????????????????????????????????????????????????????????????????????????????????????????????????????????????????????????????????????????????????????????????????????????????????????????????????????????????????????????????????????????????????????????????????????????????????????????????????????????????????????????????????????????????????????????????????????????????????????????????????????????????????????????????????????????????????????????????????????????????????????????????????????????????????????????????????????????????????????????????????????????????????????????????????????????????????????????????????????????????????????????????????????????????????????????????????????????????????????????????????????????????????????????????????????????????????????????????????????????????????????????????????????????????????????????????????????????????????????????????????????????????????????????????????????????????????????????????????????????????????????????????????????????????????????????????????????????????????????????????????????????????????????????????????????????????????????????????????????????????????????????????????????????????????????????????????????????????????????????????????????????????????????????????????????????????????????????????????????????????????????????????????????????????????????????????????????????????????????????????????????????????????????????????????????????????????????????????????

**ARENI**_*Abarenicola-Arenicola*

???????????????????????????????????????????????????????????????????????????????????????????????????????????????????????????????????????????????????????????????????????????????????????????????????????????????????????????????????????????????????????????????????????????????????????????????????????????????????????????????????????????????????????????????????????????????????????????????????????????????GGCGCCACGGAAAATGACTACTCTGAATAATTTGTCAAATAACCTATGGAAACCTAATCACGAATCATTTGAAACACCTATACCAGCACACTACTTTATAGTAACTAAAATAACTTCTTTCTTTCATATTATACACCCTTAGTACCTCTATAAGAAAATGATGGAACTCAGATTAACCACGATCCTATTTCAACTCACCCTACTGCCCGATGAGCTAAAATCTCTTTATTATATTACTAACCACAATCCAGCCAGAAACAACCCCGAGGGCAGCTCGTCGTCGCGGGTGCTGCTTCCTTCGCTCGGTGCTCCCAGTACTACACCATCCTCTATCTTCCGTTATCCCTCGCGACCTGCTGCACTCGCGTTCCAGAACCGCAAGCTCCCTCACCCGTGGGCCACGTGCGCCGGCAACACGAGCCCAACAGTGGACCTCTTCGACGCAAGAGGGAGCGGCCACGGACTCAACCGACACCTCTTCGTCGCTCGGCACATCTGGAAGAGAGTGCAAATGTCCTGTACAAGGGCTGGAGCACCTATGGATGCCCTCAACATCGATCCCCAAGAGCCCGACAGGCCCGCTCTGCCGCACGCGGCTCAGGCTCCTCGTTGTTCGACCTATGGCGACTCGCGGTCAACCGACCTGGGAGGGTCCAGGCCTGGATGAGCTGTCTACCCCCCGCAACGACCGCGCCGTCTCAATCGCCGGCGCTCCGAACCCCCGCTGGAAACTGATGAGTTAAGGAGGCACCGCCTACAGACCAGGCCGCGCAGTGGCTCCGTCCCCGACAAGGAGCGCTTGAGGATCCCTCGTAGTGTGAAGCTGGGACAACCAACAGCGTGTCCCTGCTGCACGCGAGAGTGCCGGAGGCAGCGTAGGCCTCTCCAACACGCC??????CTGTGACGCCAGGGCGCCGTTATGAGCCGGCCAAGGCTCTGCTAGCGAAGAGCTCGGAGCCGCTCCCCGCGCGCTCGGCAGCCGCGCCCGCCCGAGCTCCTCGGGCAGCGGTCCGCTCGCGCCCCCCCCGCATCCTGTTCCTCTTATGCTGGTAATACGAGCTTTAAGCCAGCAACGTTAATAGTTATGGTCCTTAATCGTACACCCTACTTTTGCAACATCAAGCTCCGACCT?TCGGGAAGAGCCTTAGATCAGACAATCGGGCCGCCTGCTGGCTTTGGGTTCAGCTCCGCCGGAGTATTTCAATTCTCCTCGTCGAAGGTAAGTGACCTCTTACCTTGGTCAAGGCCAAACCTACGCCCTGGCAGGAAAACATACGGGACTCTTATGCCCGTATTAACTTTTCCTTTAAGTCTAGTTAGACTGTCTCGGGTCCAGGCGGCGTTCGCTTCGCGGGTACTGCGACCTGACCTCCAGCCATCTCCCTTGGGCTGACTAGTTCTGGGTAGTCGGCACGTAATTACCAAAAGCTCGGCCTGAATACTGTTAGGGGCCTCGGTTCTATACGTTTTTCGGAACGGAGGTAAGGGAGGACGGCCGTTACGGCTTTGTCGCCTAGAATACTAATCGAATTTTAAAAACATGTGCACACCGACTACGATCCACGGCTTGCTTTCTGACTCGCCGGCAGCTTCCGACAAGTCTTGGCCATAGTGATTAGAATCCCCCCGCCTATTGGTGGGGTTCTAGCATATTCGCCGTGTCGACCCAACAAGTTCTTTCGACGGTTTCGCCAAGGACTGCTGTGCTGCTGCTCCTAGGGTGTCCGTTACTTCCTTCTATTGGGCCTCTCCCATGGATAGGGATAGTCCTTTTGATGGTTTGTGGAACTCGTAGGTCCTTCAGCGGAGATCGGTGTGGGTGCTAGGAACTTCGCTTGATCATCAGAACAGATACGCGGGCCTA?AATATCCCTTGGGGGCTAATGCGACACCGAAAGCATAGCGCCTAAAATAAAATAATAGTCTTCAGGAGAACCTGAAAAACTTAAAAAGTGTAGGCTTCACTTATAAAAACGAAAAAACTATATTTTTCATAAGTTGACGCGGTCAGAGCATATAACCCTCTCTCATCATAAAGTATCTGCCCGTCTGATCATAAATAGCCTCAAACTATCTCTTCCGAGCACTTGAAGAAGAGGTGGCGCAGAGTATCCTAGTAACTAACTCTTTACAAACATTGCCCATGTGGAGTCGGCTGCTCCCGGCCGGTCGACGAGAACACGTTGGGCCGGATCTTGTGTCTGTCTGTTCGGGTCCGGTCCTCCTGTTGGGGTTCCGCACTCGCCGGGGCCCGGGCGGATGGCGCGT?AGGCCCTGCGTGGAGCAGCATAACATCTGCCGGCACTCGGATTAACAGCGTTTAGAGGCGAATGGGACGTACTTTGGTCTCCCGCCTACCGCACTCTCGTGCGGTGCAGTGGATCCTTCCGGTTCGCGGCGGGCGCACTGGCGGGGGACACTCGTCGCGGTCTTGGCGGTCTAAGCGCGCGGGAAGAGCTTTGGTGAAAGATGTTA?AGCCCGTAGCCGTGTAGCCCG?CTGTCGAACAGC??CG??TTAGCAGGCGCGGGGCTTCCC?TCTGGCGTTCGACTCGTGGACTGTTCATGCTCGGACCGCGGA?GTGGGCGGGAATTCAGGGTCGCGG?CGTTGGTCGGCTCCCCATCAAGCTAAACTGTGCATGAGCGACACGAAACTCAAAGTATGAGTGAAGGCAGGTT?TTGCAGCCTAGTAGCGGTCCTCGGGCAACTACGCCGCTCAATCCCCGTGGATGAGGCGGACAACACATGCTTGGGTACCTACACACAAGCTGATCTAGACGACTTTGTTAACTCTTGCACTCGATAAGTATCGTACCTGCCGAACACATTAAGTCGGTGTCGATTACCCGGCGATTGAGGGCTGACCTAATCATACCGCGAGTGTCTCAAAATAATTTGTGAATACAACGCCGACCCGTGTCACTAGGGCAAG?TATGGCGTCGGATATCGGGCCTCC?TCGCGACGAGAGGTCTGCGGATTTGGCTTAGGCCAGCCGGGGCGCGCGGTCATACGGATATGTTGAACGTAAATAGAAACTCGTTCTGAAACGGGGCGCTGACCGGCCCGTTG?CTATATCGTATCGACCGGATGCGGATACGGCCCTTTGGGGTCCCCAGTACAACGAACCAGAGCTCGGCGGGATCCCGGACTTGAGGCGGACTCTGTCGGCTTGCCGATGATGTTGTTCCCTACCGCTCCCCGGGCGTGTGCTCTCGTCGGCTAATCTGGGG?TGAC??TGATTTGCGTCGGCCTGGACCGC?ATTTACAGATTGTCATCGAGGGGCCATCAACTGGAGTCAGTACGGTCGGT??CAAGTTGGGGCTGTTTTTCTGAGTTACTGGACTCGCCGGATGTGGGCGATTAGCGTGTACATGGCACTGGCGGC?????????GCTTCCTTCGTTTGGGTTCAAGGCTAATCCAACAAATGCTGCACTGACGGTTTGATTAAAATCCGGATTTAACGCAGAATTAGCTATCTGCCGACTTTTGGAGAATGACGGAAAGTGACAGCCGTCATGTTTCTTGTTTAAAAACGGGAACATGGCGAGGTCGGTACTTGATTCAGATGATCGGTGATGTGCGCCCTTCTAAAATGTCAGCGGATAGTGCCTGAAGCAGGCGCTGTACATTACTTTTTCGAAGTGTTAACTATTGATGAAGGGTCGTCACCTTTAATAGCCGTTTATCCTTTCCGTACATATATTCCTGCCAAAGCAATTGAATTAGGATGGAC

**BRACH**_*Terebratalia-Terebratula*

??????GGCTGGTCGGGGCTAGCCCATCTTTATGGCTGTGGGGCATAAGAATGTAGGAAACGATCGTGTTGTGATGTGCTTCATAGTATTTTGCGTGTTATAATCATTGTTGTGACTTAGGGGGGTCTCGCGGGTATTGAGTTAAAATGCATGCTCTTGCTCTGCTTGCCTCGTCTGCAGCTAAGAAGCTGTTGGTGTTTGTATAGGGGGGGCTTCATAGGGCCGGCTGGTTGCTTTTTGTCGGTTGCGTCTTCTGGGGCTATTTATGGCGGTTGCTTGGGCATGAAGATGGAGCAGGTTCGTATGTGGTCTGTCTTAATTGTGGTTGTGCGTGTCTCTGCTAGGGCTATAGTGTATACGGATTTGTTTCACTTCCCGCGGCGTCGTCTTTCGTTATATGGCTCCGTAATGAATGATTGCTTCGGTGAGTTTACCGTATAGTTGGTGGATATTATGGGGAAGGATATATCCCCCCCAAATTTGGACCTTGTATTTTGTTGTACGGAAGGGGGTAGCGCTCTTTTACGTTTCTTGTTCTTCACATCTCTGCTAGGCAGCGGTGCACTTCTGGTTATTCATGGTCGCTTCTGTGATTACTTTGCCCCGCGTTGATTAAAGTCTTTGTGGTGATATTGTAGTTCCCAGTT???????????????????????????????ATCACGAGCACTACTTTACTTGCCCTATGAGAATAGTACTATGGGATTTTTTTTCTTCCGTACTTGTTTGTAATTTTGTGTACTTATAATCTAGAATCAACAGTTTACTAACCCTTCGGAGATGACCTTCGGCATGGTGAAACCTACTCTGAGGCAAATCAAAAAACAAGGACAGACATCGAGAACTAATTAAAGCATCATTGTAGTTTTCAATTTCAGAAGGAGGAGGAGGATGAAATGTTCGATGGTTGGAAGTATGTTTGAAGCACTTAGTATTCTCCCGAGCTCGTCAGACAAACACCCACTCTGGTTCGAGGATAAATACTCTTATTATATAACATACGGTAACCTGCAGTTGGCATTCGTGTGTGTGCCGAGTCGTAACAGAGCATTATACTTTTTGGAATGAGAATACAGGCTTATGAGCTGGAGCTCTGACTTCACTAAAGAAGCTGACGAATTAAAGTGGTGGTGCCTCCAGACCATGCTATGCAATTATCCTGTCTTTAATCTTGAGTGCTGGAGGATTTCACGTTCTGAGAAACTTAGATAACCAAAAGTACGTCTCTGTTCTATGAATAAAAACGTCAGCA?????????????????????????????CTATTACACAAAGGTGTCGCCCTGAGCAGGCTATCGCCAAAACAGTTCTGCTCTCGGAGCAGCGTGCTGCCCGCTTGCCTGTTGCGCCCGCCTAAGCTTGTCGGATAGCGGCCCCGTCCTACCTCTCCCACT??????????CTTATGCTGCTAATACACACTTTCACACGGTAACGTTAATAGTTATGGTCCTTAATCGTACATCCTACTTTTATAACAAAAAGCTCCGACCTCGCGGGAAGAGCCTTAGATCAGACAATCGGGGCCTTTGCTGGCTTTGGGTTCAGCTTCGCCGGAGTATTTCAATTCTCCTCTTCGATGGTACGTGACATCCTACCATGTTAAAGGCCAAAACTTTGCCCTGGCAGGAAAACATACGGGACTCTTTCGCCCGTATTAACTTTTCCTTTAAGTTTAGTTAGACTGTCTCGGGTTTAGGCGGCGTCCGCCTCGCGGGTACTGCGTCCTGACCTCCTCCCGGTTCCCTTGGGCTGACTAGTTCTGGGTGGCCGGAACGTAATTACCAAAAGGACTGCCTGAATAATGGTAGGTGCCTCGGTTCTATTTGTTTTTCGGAACTGAGGTAATGGAGGCCGGTCGTTACGGTTTTGTCGCCTAGGAAGCTAGTCGAGCTTTAAGAACATCTGCACGACAACTACGATTCCCGGATTGCTTCATGACTCGGCGACAGCTTCCGACAAGTCTTGGCCATAGTGATTAGAATCCCCCCGCTTTTTGGTGGGGCTCTAGCATATTCACCGTGTCGGTGCAACAAGTGCGTTTCACAGTTTCGCCAAGGAATACGTTGTCGCTGGTCCACGGCCGTCCGGTACCTCCTTCTATTGGGCTTCTCCCATGGACAGGGATAGTCTTTTTAATGGTTTGTGATAATCGCTGGTCCCGCG?GGGTTCGCCTCGCGGTGACGGGGACGATCACTTGATCATTA?????????????????????????????????????????????????????????????????????????????????????????????????????????????????????????????????????????????????????????????????????????????????????????????????????????????????????????????????????????????????????????????????????????????????????????CCCCACAAACTAAGCTGATTCGGAGACGACCGCCCCCGGCGCGCCGGCGTGACAGTGTTAGGACGATCTATGAGGCGTCGCTCCGTCGCCCAGTCCTCCTGTCGGGGTTACACGCTCGCTAGGGCGACGGGCAGCGTCCCTTGAGTCCTTGCGTAGAGCAGCGTAACATCTGCTTGCACTCGGATTAACACCGTTCAGAGGCGGGTGGAACGCAAAGTCTAGCCGGGGATGCCGG?????????????????GCGATCGCGCGAGCGG?CGACGGGCGCACTTCCCCGGCGAGCCCACGACGGTTGTGGCCGCCGAATCCCCGGGAAAGAGCCGTCGGGCTCGGTGTTATAGTCCCGGTGGCGTTGTTCGGCCGGACGACCAGCCACGCTCCCGCCCGGCTCGACTCGCGG?TCGTCGGTTCGACTGGTGGACTGCGCATGCTCCGACCGCCGCCGGTCCG?GCCGCTCAGGGTCTGTGGCGCCGGTCGGSCCTCCACCAAGCTAAATTGCGCACTGGTCCTGCGAAACCCAAGGCATGAGTGAAGGGCCA???GGTGGCCCGAGTGGTCCCGCGTTCGGGCCCGCGCCGCTCGTCCGCCGTCGGTGAGGCGGACAACGTACACTTGGGTACTTATATACAAGCTGGTCTAGACGACTTGATTGACTCCTGCACTCTTCGAGTATCGTACCTGACGAACACATTAAGTCCGTGTCGACTACCGGAGTTTCGAGTGCCGCCCTACCCCTATCACGGGCGTCTCATAATAATTTGTGAATACAACGCCGACCCGTGTCACTAGGGCGAGATAGGGCGTCGGGCACCGGGGTGCCGCCGCGACGGGAGGCCGGCGGGGCGGGTCTCGACCAGTCGAGGCGCGTCGCCATACGGATATGTTGAACGTTGATAGAAACTCGTTCCGAAACGGGGCGATGCCGCGCTCGTAATCTATATCGTATCGACAGGACACGGAGATGGGCCCTCGGGCTCTAGTGCGCAACGAACTCGAGCTCGGCGCGATCCCGGACTTGAGACGGACTCTGTCGGTTTGCCGATGACGTTGTTTCCTACCGGCCTTCGGGCGTGCACTCGCGTCGGCTAATCGAGGGACGGTGTAATTTCGTGCCTGTCTGGACCATCGATAACGGATTATCATCGAGAGGCTGTCGTCTGGGGTACGAGCGGCCTGGAACTGACAGGGGCTGGCGAGGCCGCAAGGTCGACTCGCCGTCGTCGGGACCTTCCCGTGGACTGCTCACTGGCGTCCG???????GGGCGCTTCGACCGGGTCCAAAGCTGATTTAACAAATGCTGCGTAACTGGTGTGATTAAAATCCGGATCTAACGCAGAATTAGCTATCTGCCGACTCTTAGAGAATGAGAGCGCAGGAAGTTTTTTACGTTTCTTATTTATAAGCGGAGAGCGGGCGAGCCCTCCTCTGGAACGTCCGGCTCGGCCGGACGCGTCCCTCTAAAGTGTCAGCGGATAGAGCTTGAAGCAGGCGCTACACATCACTTTTTCGAAGTGTTAACTATTTATTAAGGATTATAACCTTTGATCGTCGTTAATCCCTCCCGTACATTTATTCTTGCTATAACAATTGGATTAGGATTGAC

**CAPIT**_*Notomastus*

????????????????????????????????????????????????????????????????????????????????????????????????????????????????????????????????????????????????????????????????????????????????????????????????????????????????????????????????????????????????????????????????????????????????????????????????????????????????????????????????????????????????????????????????????????????????????????????????????????????????????????????????????????????????????????????????????????????????????????????????????????????????????????????????????????????????????????????????????????????????????????????????????????????????????????????????????????????????????????????????????????????????????????????????CCCGTCTCCGCGGGAGCTACTTCCTCTGCATGGTACTCCCAGTACTATGCCATCTTCCCTCTTCCGCACTGTCTCGCGATGTGCTGTGTTAGCATACCAGAACCGGCTGTCCGCTCTCCCTAGGGAGACATGAGCCGGCAACATGAGCCCAACTCTGAGGCCCTTTAAGACTGCAGAAGAGGCCCACGAATTCAACCAAGCATGCATTGTGACACGGCATATCCGGAGACCAGCACCAATGTCCTGTTCAAGGGATGGAGCACCCATGGCTGCTCACGCCATTGAGCCACAAGCTCCTGACAGGCCCTCTCTGCGGTACGTGGATTAAGTATCTGGYTATCACGCATATGGGTACCTGCTTGCAACGGACCTGCGTGGGCCACTGCCTGCCAGAGCCTTCTTCTTCCCGCAACGGACGAACCGCCTTTACGTGTGGAGCTCCGCAGTCTCCAAGGAAACTGACGAGTCAGAGTGACACCGCCTCACAACTCCGCTCTGCCATCGCTCTGACTCCAGCATGGAGCGGTAGAGGATTCCTCGTTCTGCGAAGCTGAGATGCTCCAGAATGMGTCTTTGTGCCAGGTGAGAAAGCACAGGCCAGCGTGGGCCTCTCAGACACACC??????CTATCACGCCAGGGCGCCGTCCCGAGCCCGCCACCGCCAGGCCAGCTAGGCTCTCGGAGCCGCCCCCCGCGCGCCCTGAGACCGCGTCCGAGCGAGCTCCTCTGGCAGCGGTCCGT??????????????????????TCCTCTTATGCTGCTAATACATACTTTTACACAGTAATGTTGATAGTTATGGTCCTTAATCGTACATCCTACTTTTGTAAAAAGACGCTCCGACTCTAGGGGAAGAGCTTTACAACAAACAATCGGCCTCTTTGCTGACTTTGTTCTCAGCTCAGCCGGAGTATTTCAATTCTCCTGTTCGATGTTAGTTGACATACTATCATCTTAAAGGCCAAAACTACGCCCTGACAGGAAAACATACGGGACTCGTTCGCCCGTATTAACTTTTCCTTTAAGTTTAGTTAGACTGTCTCGGGTTTAGGCGGCGTTCACCTCGCGGGCACTGCGTCCTGACCTCCTGCCGGTTCCTCTGGGCTGACTAGTTCTAGGCGGCCGGAACGTAATTATCAAGAGGTTTGCCTGAATAATAGTAGGGGCCTCGGTTCTATTTGTTTTTCGGAACGGAGGTAAGGGATGACGGTCGTTACGGTTTTGTCGTCTAGAATACTAATCGAATTTTAAAAACGTGCGCTCGACAACTACAATTCCCGGCTTGTTTCATGACTCGGCGGCGGCTTCCGACAAGTCTTGGCCATAGTGATTAGGATCCCCCCGCTTATTGGTGGGGCTCTAGCATATTCGCCGTGTCGGTGTAACAAGTTCGTATGACGGTTTTGCCAAGGACTACGTTGTCGTTGGCCCGGGGCTGTCCGTTACCTCCTTCTATTGGGCTTCTCCCATGGACAGGGATCGTCCTTTTGATGGTTTGTGATCCTCGTTGGACCCTCGGGGGTTACCGCCGCGGCGGCGAGGACGATCACTTGATCATCAAAA???????????????AGAAGTTCCCTTGGGGGCTGTTGAGATGACGTGGCCCTCGCGCCTTGATTTAATTTATAGGTTTTAAGAGCACTTGAAGTGGTTAGAGAGTGTTGGCTTTAGTTTATAGTTGTGATG?????????????TGGGCTTGGTGCGGCAGAAGCAATAG?AGCGACTTTTTT?TTTTATTTAAGCTAGATATATAGTGAAAAGCCACAGGCTATCCACTCAAAGTTATTGATGAGTGGG???????????????????????????CTCAACAAACTAAGCCTACGCGGATACGACCGCCCCTGCCCAGGTAGCGGGAACGTGTTAGGACTGCCTATGGGGTGGTGTTCGGACGTCCGGTCCTCCTGTCGGGGCTTCATACTCGTCACGGCGATCGTCGCCTCCCCTT?GGTCCTTGCGTGGAGCAGTGTAACATCTGCTGACACACGGATTAACAGCGTTCAGAGGTGGATGGGACGCAACCTCC?CCTGTGGATACC?GTCTGCCCGTCGGTTTC?CGGATCGCACGGCGGGGCCGGGGGCGCATTTTCGCAGGGA?ACCGCGACGGTTCTGTCGGTCAAAGCCCGCGGGAAGAGCTCCCTCGGAGAGTGTTATAGCCCGCGAGGTGTTGGCCCGGCGCCGGACCAGG??????CCCGCCGGCGGGGG?CCGCCT?ACGCTCGTTCGACTGGGGGACTGTTCATGCCCCGACCGCGGT??GGGTCGGGCCTTTAGGGTCTGCGGCCTCGGTCGGCTCCCTATCAAGCTAAATTGCGCATGGGCTCTACGAAACCTAAAGCATGGGTGAAGGCCCTCCTCTAGGGCCTAGTCGTCCCGCCTGCGGGCAGATGCCGCTCGAAGGCTGACCTTGAGGCGGACAACGTACACTTGGGTACCTATACACCAGCTGGTCTAGACAACCTGGTTAACTCTTGCACTCT?CGAGTATCGTACCTGGTGAACTCATTAAGTCCGTGTTGATTATTGGGCGATCGAGGGCTGACCTACGGCTATTACGATGATCTCATAATAATTTGTGAATACAACGCCGATCCGTGTCACTAGGGTTGGATAGCCCGAGAGGCATCGGGGAGCCTCCGTCTCGAGAGGTCTGCGGAATTGACCTTGGCCAGCTGGGGCGCGCGGTCATACGGATATGTTGAACGCAGATAGAAACTCGTTCTGAAACGGGGCAATGAACGGCCTGTAT?CTATATCGTATCGACCGGACACGGAGATGGCCCCTCGGGGTTCAGTGCGCAACGAACTAGAACTCGGCGGGATCCCGGACTTGAGGCATACTCTGTCGGCTTGCCGATGACGTGGTTCCCTACCGCTCCCCGGGCGTGCACTCCCGTCGGCTAATCTAGGGACGGTGTGATTTCGTGCCGGGCTGGACCAT?GATAACAGATTATCATCGAGAGGTTGTCGTCTGGAGTACGAGCGGCCTGGAACTGGCAGGGGCTGGTGAGGCCTTCGGGTCGACTCGCCGTCGCCGGGGCCTTCCCGTGGACTTCCCACTGGCGGCGGTCG?GTCGTTCGCTTCGGCCGGATTAAAAACCGATTTAACAAATGCTGCGTAGCTGGTGTGATTAAAATCCGGATCTAACGCAGAATTAGCTATCTGCCGACTTTTAGAGAATGAGAGCAC?GACAATTTTTTACGTTTTTTATTTATGAACGGAGGATGGGCAAGCCCTTTCCTGGAACGCC?GACTCGGTCGG?CGCGTCCATCTAAAGTGTCAGTTGATAGAGCTTTAAGCAGACGCTACACATAACTTTTTCGAAGTGTTAACTAGTTATTAAGGATTATAACCTTTGATCGTTGTTAATCCCTCCTGTACATTTATTCTTGCTATAACAATTGGATTAGGATTGAA

**CHAET**_*Chaetopterus*

CCAATCAGCTCGAAGAGAACTGCCCTATTATATTGCAACAGCACATATCACTATAGGGTCAGATCACGCTGTTATGTTCTCCCTCGAATTCTTAATATAGCCATAATTGACGCTCTGCAACAGCAGCCCATGGCTTCTACACTAAATTGCACCCACTTTCTACTCACTCTTCTTCTTCTTTAGAGAAACATAATAAGCCATCTGAGGAAACCTAGCCCGCCCCCCTCCGTCTACTTTTTACCAGCAATTTCCTTTAGTGCGGTTTAATCAAACTTTTTAGGTATACTGATAGAGCGAATTCTTATGCTAGCTATTTTAATAGTAATCCTACCCATCCTAGTCTTCGCTATTGCCTACTTGTATTTATATCACTTTCCGCGGATCTATCCACCACCTTAT??????????????????????????????????????????????????????????????????????????????????????????????????????????????????????????????????????????????????????????????????????????????????????????????????????????????????????????????????????????????????????????????????????????CTGGAAGCCCATCGTCATGAGCGCAACTTCTTTTGCACGATACCAAGAATACTATGTAATTTTCTCCAATCCGCTTATTTTTGTGACTCTCTGTACTTGTATTCCAGAACTACAGACTCGTGCACTCTAGGGCTACATGTGCCGGCAACAAGAGACCAACAGTGAAGCCAATCAACACAAGAAGTGAATCTCACGGACTCGACCAAGGGTTGTTCTGCACTAGGCATACCTGGAGAAATCCGAAAATGGGATGTTCAAGGGATGGTCTAACTCTTGAAGCTCTCTCGATCAATCCCTAAGCCACTGACAAGGACCCTCTGTAGTCCAAGGATTAAACCTAATATTTGCACGGTTATGGCCACCTGTAGCTAACCTACCTGAATGTGTTCAAGTTTGGAGAAACCGAATTCTAACCGTAATGCTCACACCGTCTAAACAGCTGGTGCAGCGAACTCCCAAAAGAGACTGCCAACTTGATGAGTCACCATTCTAGCACGTTAAGACACCAGTGTCCAGTCTCCAACTTTGAACGAAGGAAGATCTCTCGATCTGTGAAACTGAGACAACAGACAAGCTGAGTTCTTGCTATGTAACAGTCCCAGCGCCAGTGTTATCATTTCTGACACCAAATGTCGCCATCTAGATCGAACGCCGCACTGAGTAGGCCAACGCTCTGATCATCCTACTCTCGGAGCCGCGCCCCGCGCGCCCTCCTACTGTGCTCCCCCGAGCCTCTCGAGCAGTAGTCCGCTCCCACCCTCCCCGCT???????????????????????????????????????????????AAAAGTTATGGCCCTTAATCGTACATCCTACTTTTGTAACAATAAGCTCCGACCTCGGGGGAAGAGCCTTAGATCAAACAATCGGGCCCTTTGCTGGCTTTGAGTTCAGCTCAGCCGGAGTATTTCAATTCTCCTCTTCGATGGTACGTGATATCCTACCATGTTAAAGGCCAAAACTACGCCCTGGCAGGAAAACATACGGGACTCTATCGCCCGTATTAAATTTTCCTTTAAGTTTAGTTAGACTGTCTCGGGTCCAGGCTGTGTCCACCTCGCGGGTACTGCGTCCTGACCTCCTCCCGGTTCCCTTGAGCTAGTTAGTTCTGGGTGGCCGGAACGTAATTATCAAAAGATTTGCCTGAATAATGGTAGGTGCCTCGGTTCTATTTGTTTTTCGGAACTGAGGTAAGGGAAGACGGTCGTTACGGTTTTGTCGCCTAGAATACTAATCGAATTTTAAAGACATGTGCACGACAACTACGATCCCCGGATTGCTTCATGACTCGGCGGCAGCTTCCGACAAGTCTTGGCCATAGTGATTAGAATCCCCCCGCTTATTGGTGGGGCTCTAGCATATTCGCCGTGTCGGCGCAACAAGTGCGTTTCACAGTTTCGCCAAAGAATACGTTGTCACTGGTCCANGGTTGTCCGTTACCTCTTTCTATTGGGCTTATCCCATGGATAGGGATAGTCCTTTTAATGGTTTGTGATCCTTGTTGGTCCCGCGGAGGCAACTGCGGCGGTGGCAGAGACGATCACTTGACTATTA??????????????????GGAATTCCCTCTGCAGGCTTTCGTGACAACGAGAGAAAAGCGCCACTTAAACATAAATAGTCTTCAGGAGAACCTGAAAAAATTAAAGAGCGTAGGCTCTGTCCTAACTCCAATATTACTTAAATCTATAAAAGACTAGCGCGGCTAAGGAAACAAAATCATCCATTTAATTAAATATAAGCTTGCCAGACCAAATATAGCCTCGAATTTTCCTTCTTGAGTTACTGACAGAAGGGC??????????????????????????CTCCACAAACTAAGCCCATGCGGACACGACCACCCTTGCCGCGGCAACGGGAACGTGTTAGGACAGCCTATGCGGCGGCGTTCGGGCGTCCAGTCCTCCTGTCGGGGTTGCGTGCTCGTCACGGCGTCTGGCGCCGTCGCTAGAGTCCTCGCGTGGAGCAGCGTAACATCTGCGGACACTCGGATTAACACCGTTTAGAGGGGGATGGGACGCAAAGTCA?GCCCGGGATGCCGCGCGCCGCGCGCTGC????GGATC???TAGCGCG?CGGCCGGCGCACTTCCCGGGCGAGCCCACTACGGTCGCGGCGGTCAAAGCCCCCGCGAAGAGCCATCTCCGGACTGGCGA?AGCGCGGGCGGTGTCGGCCCGCCGGGCGACCAGAGACGCTCCAGCCCGCCTCGGCCG????????????TTCGACTCCTGGACTGTTCATGCTGGGACCGCGGGAGGACCG?GGG??GGGCGCGAAGTGGCGTCGGTCGGCTCCCCATCAAGCTAAATTGCGCACGGGTTCTACGAAACCCAAGGCATGGGTGAAGGTCCATC?GCTGGACCCAGTGGTCCCGCCTGCGGGCCCACGCCGCTCGTCCGCTGCCGGTGAGGCGGACAACGTGCACTTGGGTACCCATACACAAGCTGATCTAGACGACTTGGTTGACTCCTGCACTCCACGAGTATCGTATCTGGCGAACCCATTAAGTCCGTGTCGAATACCGGGCGATCGAGTGCAGCCCTACCCCTACCACGATGATCTCATAATAATTTGTGAATACAACGCCGACCCGTGTCACTAGGGTCGGGTAGGGCGTCGGGCACC?ACGTGCCCCCTCGACGGGAGGCCCGCGGGACAGGCCTAGGCCAGCCGGGGCGCGCGGCCATACGGATATGTTGAACGTAGATAGAAACTCGTTCCGAAGCGGGGCAATGCCGAGCCCGTATACGATATCGTATCGACCGGACGCGGAGACGGCCCCTCGGGGTCCAGTGCACAACGAACTAGCGCTCGGCCGGATCCCGGACTTGAGGCAGACTCTGTCGGCTTGCCGCTGACGTTGCACCCTACCGGCCTTCGGGCGTGCACTTCGGTCGGCTAAGCTAGGGACGGTGTGATTTCGTGCCGGAGTGGACCAT?ATTAACAGATTATCATCGAGAGGCTGTCGTCTGTGGTGCGAGCGGCCTGGAACCGGCAGGGGCTGGCGAGACCTCGCGGTCGACCCGCCGTCGCCGGGACCTTCCCGTGGACTGCTCACTGGCGGCGCCGC????GTCCGCTTCGTCCGGGTCCAAAGCTGATTTAACAAATGCTGCGTCACCGGTGTGATTAAAATCCGGATCTAAATCAGAATCAGCTATCTGCCGACTCTTAGAGAATGAGAGCACGGGCAGTTTTTTACGTTTCTTATTTATAAGCGGAGAGCGGTCGCGCCCTCCACTGGAACGCCCGGCTCGGTCGGGCGCGTCCCTCTAAAGTGTCAGCGGATAGAGCTTGAAGCAGGCGCTACACATCACTTTTTCGAAGTGTTAACTATTTATTAAGGATTATAACCCTTGATCGTCGTTAATCCCTCCCGTACATTTATCCTTGCTATAACAATTGGATTAGGATTGAC

**CHRYS**_*Dysponetus*

AGAACAGGCCGACCAAGCACCTCGAAGATACATAGCTACAGGACGCCTCCCTTCTGAAAGAGACCACATTACAATGATCCTCTTTGAATCCTAAGTGTTATAATGATTCTCATTTATCAACAGGAGTCCATAGTTTCGACATTTAAATACACCCCCAGACAACTCTCCCTACATCAGCAGCTGAGAAAGAAAAGATGCCCCTATAGATAATATCTCCTAGAGCCCTCTGTTTACATCCCATTAGAGATTAGAATTAGGGCACTTTATACAAATTATATAGAACTTCGACTAGAACGACTGCACTTATCGTCAGTAAAAATTGTAATTACTCTCTTCTCCGATATGACCATGATATACTCGTATATACAGCTCTTCCATCAAAATTATTAACCATCAT????????????????????????????????????????????????????????????????????????????????????????????????????????????????????????????????????????????????????????????????????????????????????????????????????????????????????????????????????????????????????????????????????????????????????????????????????????????????????????????????????????????????????????????????????????????????????????????????????????????????????????????????????????????????????????????????????????????????????????????????????????????????????????????????????????????????????????????????????????????????????????????????????????????????????????????????????????????????????????????????????????????????????????????????????????????????????????????????????????????????????????????????????????????????????????????????????????????????????????????????????????????????????????????????????????????????????????????????????????????????????????????????????????????????????????????????????????????????????????????????????????????????????????????????????????????????????????????????????????????????????????ATGCTGCTCATACAGACTTTTACACAGTAACGTTGATAGTTATGGTCCTTAATCGTA?TCAGTACTTTTGTAACATCGAGCTCCGAGCTCGCGCGAAGAGCCTTAGATCAAACAACCGGCGACATTGCTGGCTTTGTGTTCAGCCGTGCCGGAGGATTTCAGTTCTCCTCTTCGACGGTACGTGCCTTCCTACCGTGCTAAAGGCCAAAACTACGCCCTGGCAGGAAAACATACGGGACTCTTTCGCCCGTATTAACTTTTCCTTTAAGTTTAGTTAGACTGTCTCGGAGCCCGGCGACGTTCGCCTCGCGGGCACTGTGTTCTCACCGTCGGGCGGTCGCCCGGGGCTCGCTAGTTCCTGCGTGCCGACGCGTAATTATCAAAAGTCTCGCTCGAATAATGGTAGGTGCCTCGGTTCTATTTGTTCTTCGGAACCGAGGTAAGGGAAGACGGTCGTTGCGGTTTTGTCGCCCAGAATACTAATCGGATTTTAAAAACA?CTGCACGACGACCACGATCCCGGGATTGTTTCATGACTCGGCGGCAGCTTGCGACAAGTCTTGGCCATAGTGACCAGGATTCCATCACCTGTTGGTGGGGCTCTGACGCATCCGCCGTGGGGGTGGGACAGGTGCGTTCCACAGTTTCTCCAAGGACTGCTGTCCCCTTGGCCCACGGTCGTCCGTTACCTCCTTCTATTGGGCCTCTCCCATGGACAGGGATCCTCCTTTTAATGGTTTGTGGTCCTCGTCGGTCCCCCGCGCGCGGTCATCGTGTT?GCGAGGACGAT????????????????CAGATACGCGGACCAAAAATTTCCTTTTAAGGCTAATGCGACTCCGAAGAATTCTCGCCTCGGGCCTCTAAATAAACTTCAGGAGGACCTGAAAAGTATAAAGAATATAAGCTTCTACTGAAACTTAAAGCT??TTAATCTAA??TCAAGTTAGTGCGACAAAGGTTTATAC?CCCTCCTCTTAAAAAAGAATATATCAG??ATGGATATAAAAGCTTTAAGCTAT?TTTCTAACGTCATCAACAGAAAAG?GGCGCAGGA?ATCCATGTAACATATC??????????????????????????CGACCGCCCCGGCGACGTACGCGGGACCGTGTAGGGACAGCC?CTGGAACGGCGGCCGGTCGTCCGGTCCTCCTGTCGGGGCTGCATACTCGTCACGGCGACCGCCTCCGCTCCTGGAGTCCTCGCGTGGAGCAGCGTAACATCTGCCGACACTCGGATTAACAGCGTTTAGAGGCGGATGGGACGCAAAGTCGCCCCGAGGATACTGTAGCGGACCTCCCTCTGCCCGTTCGC??????????????????????????????????????????????????????????????????????????????????????????????????????????????????????????????????????????????????????????????????????????????????????????????????????????????????????????????????????????????????????????????????????????????????????????????????????????????????????????????????????????????????????????????????????????????????????????????????????????????????????????????????????????????????????????????????????????????????????????????????????????????????????????????????????????????????????????????????????????????????????????????????????????????????????????????????????????????????????????????????????????????????????????????????????????????????????????????????????????????????????????????????????????????????????????????????????????????????????????????????????????????????????????????????????????????????????????????????????????????????????????????????????????????????????????????????????????????????????????????????????????????????????????????????????????????????????????????????????????????????????????????????????????????????????????????????????????????????????????????????????????????????????????????????????????????????????????????????????????????????????????????????????????????????????????????????????????????????????????????????????????????????????????????????????????????????????????????????????????????

**CIRRA**_*Cirratulus-Cirriformia*

????????????????????????????????????????GCACGCCACATTCCAGGAAATGACCAATTTGTTCAGAACCCCATCCAATCCCATGCGTATTACGTCTCGTCACAATACTACATCTTGCCGCAGATTCATAATTCAATCACTCCCTCCTCCACTACTCTGATCATCAGCTGCAGAGAACGGCAACAAGGCAGCATCGGTGCGACATCCTAGACCCATCAGACCGCACCTTATTAGCGGTATCCCTCCGATCAATTTACACCAGCGAAACAGGCTGCTCAGCAGAACGAATTCCTACGTGATCTTTAGTAGATACCACCCCACTCATCACAGTCAAGTCCACCACACACTCGAACCTATCTCCTTCCCAGCGCTACTATCAATCATCATATGACCCCATCGAAAACGACATTCCTGTAAAATTCATTCCATAGCTAATAGCTACCACAACCCGAATTATTTGACACACCACTCCAAGCTTACTATTCCACCATGATGAAAGAATCTTCTTTCATTTACATTATACACCACTTACCCTCTCACAAGAAAAC???????????????????????????????????????????????????????????????????????????????????????????????????????????????????????????????GCGCTACTTCCCCTGCTCGGCGCCACAAGTACTACACCATCTCCTCTCTTCCGCTATTCCCCGTGACCTGCCGTACTAGTGTCCACCAACTGACTGCCCGCGCACCCGTGGGCGACGTGTGCCGGCAACACGAGACCAACAGCGAGGCTCTTCAACGCAACAGGGAGCGCCCGCGAGGACAACAAAAGCCGGTTCGTGACGCGGCACACCTGGAGGAGAGCTCTAATGTCCTGTACAAAGGCTGGAAGACCCTTCCAGGCCCGCTCCATCCTGCCCAGAGCCACTGACAAGCCCACTCTCCGGTACGCGGATCAAGAGACTGACTATCACGGCTATGGGGACCTGTCAACCGTGCACATGGATGGACCGAGCAGCGGAAAAGGCATGCACTCTCCACAACGCCCGGGGCGCCTCAACCTGCGGTGCAGCGAACTCTCACTGGAGACTGAGAACTCACTGTGGCGGCGCCTTGAAACCGCGAACCAGTCGCCCCCCGTCCCCAGCGTAACACCAAGGAGGATTCCTCGTAGCGCGAAGCTGAGAAGCCGAAGACTGGGTCTCCGCGCAACGCCTCTAAGCACAGGCCAGCGTGGAAGTCTCCGAGACGCTCTGCGCCTAGTTCGCAAGAGCGTCAATCCGAGCTAGTCATCGCTCTATAAGTCCTGCTCTCGGAGCGGCTCGAGGCGCGCCCGCCTGTTGCGTTCGCTCGAGCTTCCCGGGAAGCGGACTGTA???????????????????????????????????????????????????ACAGTAATGTTAATAGCTAAGGTCCTGAATCGTACATCCTACTTTTATAACAACAAGCTCCGACCTCACGGGAAGAGCCTTAGATCAAACAATCGGTTCCTTTGCTGGCTTTGTGTTCAGCTTAGCCGGAGTATTTCAATTCTCCTCTTCGATGGTACGTGATATCCTACCATGTTAAAGGCCGAAACTACGCCCTGACAGGAAAACATACGGGACTCTTTCGCCCGTATTAACTTTTCCTTTAAGTTTAGTTAGACTGTCTCGGGTACAGGCTGTGTGCAGTTTAGGCGTACTGCGTCCTGACCTCCTACCGGTTCCCTTGGGCTAAGTAGTTCTGGGTGGCCGGAACGTAATTACCAAAAG?TTTGCCTGAATATTAGTATGTGCCTCGGTTCTATTTGTTTTTCGGAACCGAGGTAAGGGAAGACGGTCGTTACGGTTTTGTCGCCTAGAATACTAATCGAATTTTAAAAACATGTGCACGACAACTACGATCCCCGGATTGTTTCATGACTCGGCGACAGCTTCCGACAAGTCTTGGCCATAGTGATTAGAATCCCCCCGCTTATTGGTGGGGCTCTAGCATATTCACCCTGTGGGTGTAACAAGTGCTTTCTACAGTTTCGCCAAGGAATGCATTGTTCTTTGTCCAAGGCTGTCCGCTACCTCCTTCTATTGGGCTTGTCCCATGGACAGGGATAGTCCTTTTGATGGTTTATGATCCTCGTCGGTCCCGCGG??CTTTATGTTGCGGAGGCGAAGAATGTCGTTTGATCATCA??????????????????GACCTTTCCTCTGGAGGATATTGTGATAACGAGAGTGTCACGCCTAAGACAAATAAATATCACTCGGGAGGACCCAGAACCATGAAAAAATGTAGGCTTAATCTTACCCACATTTATATTTAAAATTTTTTAAGATTGGTGCAACCGAGGTAATTAAATCATCCTCCTACCTAAGAATACGTCAGTCTGATCAACACAAGCCTCAAGCTATCCCTCTTAAGTTACCAACAGGAGTG?GGC???????????????????????CTCAACAAACTAAGCTTATGCGGAGACGACCGCGCCTGCCGAGGTAGCGCGAACGTGGTAGGACAGCCTTTGAGGCTGTGTACCGGCGTYCAGTCCTCCTGTCGGGGCTTCATACTCGTTACGTGCCGGTCGCAGTCCTCTTGAGTCCTCGCGTGGAGCTGCGTAACATTTGCTGACACACGGATTAACAGCGCTTAGAGGTGGATGGGACGCAAAGTCGACCCGCGGATACCGTCTGGTGCCTGATACG?TCGGATCCTCCGGGCTGGTGGCGGGTGCACTTCCGCGGGGAGCCCGCGACGGCTCCGGCCGTCAAAGGGCGGGGAAAGGACCCCGGTCGTGGGTGTTACAGTCCCCTTTCCTGTGGCCGGCTGTTGGGCCAGATT????CCCGCCGGGCGTGGCTCGCCTTCCGCCCGTTTGACTGGAGGACTGCGCATGCTCCGACTGCGG?TAGGTCCGCGCGCGTAGGGTCAGTGGCGTCAGTCGGCACCCCATCAAGCTAAATTGCGCATGGGTTCTACGAAACCTAAAGGATGAGTGAAGGCTGCTC??TCATGCCCAGTAGTCCTTCTTGGAGGCCTATGCCGCTCGATCTCTGTAGACGAGGCGGACAACGTACACTTGGGTACCTACACACAAGCTGGTCCCGGCGACTTGGTTAACTTCTGCACTCTTCGAATATCGTATATGACGAACACATTAAGCCCATGTCGATTACTGGGATATCGAGTGCCGCCCTACTCCTACAACGAAGATCTCACAATAATTTGTGAATACAACGCCGATCCGTGTCACTAGGGTCGGGTAGGACGTCGGGCATCAGGAAGGCACCT?GACGGGAGGTCTGCGGA?TTGGCCTCGGCCAGCCGGGGCGCGTGGTCATACGGATATGTTGAACGCAGATAGAAACTCGTTCCGAAACGGGGAAATTCTTCGCCCGTA?CCTATATCGTATCGACTGGACTCGGATATGGCCCTTCGGGGTCCAGTGCACTACGAACCAGAGCTTGGCGGGATCCCGGACTTGAGGCGGACTCTGTCGGCTTGCCGATGACGTTGTCCCCTACCGCTCCCCGGGCGTGCACTCCCGTCAGCTAATCTGGGGACAGTGTGATTTCGTGCCAGACTGGACCATCGATAACAGATTATCATCGAGAGGCTGTCGTCTGGGGTACGAGCGGCCTGGGACCGGCAGGGGCTGGTGGGACCGCAAGGTCTACTCGCCGTCGTCGGGACCTTCCCGTGGACTGCTCACTGGCGGCGG???GGTCGTTTGCTTCGGCCGGGTCTAAAGCCGATTTAACAAATGCTGCGTAGCTGGTGTGATTAAAATCCGGATCTAACGCAGAATCAGCTATCTGCCGACTCTTAGAGAATGAGAGCACGGGCCAGTTTTTACGTTTCTTATTTATAAGCGGAGAGTGGGCAAGCCCTTTCCTGGAACGCCCGACTCGGCCGGGCGCGTCCCTCTAAAGTGTCAGCGGATAGCGCTTGAAGTAGGCACTACACTTCACTTTTTCGAAGTGTTAACTATTTATTAAGGATTATAACCTCTGATCGTCGTTAATCCCTCCCGTACATTTATTCTTGCTATAACAATTGGATTAGGATTGAC

**CLITE**_*Capilloventer*

??????????????????????????????????????????????????????????????????????????????????????????????????????????????????????????????????????????????????????????????????????????????????????????????????????????????????????????????????????????????????????????????????????????????????????????????????????????????????????????????????????????????????????????????????????????????????????????????????????????????????????????????????????????????????????????????????????????????????????????????????????????????????????????????????????????????????????????????????????????????????????????????????????????????????????????????????????????????????????????????????????????????????????????????????????????????????????????????????????????????????????????????????????????????????????????????????????????????????????????????????????????????????????????????????????????????????????????????????????????????????????????????????????????????????????????????????????????????????????????????????????????????????????????????????????????????????????????????????????????????????????????????????????????????????????????????????????????????????????????????????????????????????????????????????????????????????????????????????????????????????????????????????????????????????????????????????????????????????????????????????????????????????????????????????????????????????????????????????????????????????????????????????????????????????????????????????????????????????????????????????????TGCTGGTAACACAAGCCTTAACACGGTAGTGTTGATACTCATGGTCACTAACCGTACAAGTTACTTTTGCAACACCAAGCTCCGACCCTTGGGGAAGAGCCGTAGACCAAACAATCGGGTTCTTTGCTGGCTTTTCGTTCAGCTCCGCCGGAGTGTTTTGGTTGTTCTCGACGAAGGTACGCTACATCCTACCTTGGTT?GGGCCAACACTACGCCCCGACAGGAAAACATACGGGACTCATTCGCCCGTATAAACTTTTCCTTTAAGTTTAGCTGGACTGTATCTGATCGAGGCTGCGTTCGCCATACGGGAACTGCGTCGTGACCACCTCGCGGTGTCCTTGGGCTCGCTAGTCCTGGGCTGCCGGAAAGTAGTTACCAAAAGTCGTGCCTGAATAGTCGTAAGTGCCGCGGTTCTATTTGTTTTTCGGAACCGCGGTAACGGAGGACGAACGTTACGGCCGTGCCGTCTAGAAAACTAATTGAATTTTAAAAACNTGAGCACGACAACTGCGATTTTAGTCTTGTTTCATGACTCGTCGACAGCCTCCGACTAGTCTTGGCCATATTGATTAGAATCCCCCCGCTTATTGGTGGGGCTCTAGCGCATTCACCGTGTCGGTGCAACAAGTGCGTTCCACAGTTTCGCCAAGGAATGCGTTGTTTTTGGTCCTAGGTCGTCCGTTACCTCCTTCTATTGGGCCTGTCCCATGGACAGGGATAGTCCTTTTGATGGTTTGCGATCCTCGTTGGGCCCGCGGGGGCAACCGCTGCGACGCCGAAGACGATCAC?????????????????????????CCTAATAATTTCTATGTAGACTGTTGTGGCACCGA?ATTAACTCGACATAAA?TTAAATATTATATTGGGGAGAGCCCCAAAATTATAAAGAGTATANGCCTAAT???????????????????????????????GATTTGCGTGGCATCGGTATTTGTAACTTCTGAGA?AAGTCGTGTTAGTCTGTCTAGATTAGAAAGGCCTTAAGCTATCTTACTTTAGCCCTTCAAAGTACGG?????????????????????????????TCACAAACTAAGCCTATGCGGAGACGACCGCCCCCGGTAGGCCGGCGGGAACGTGTTAGGACGGTC??GCGCGGAGCGGTCGGGAGTCGGGTCCTCCTGTCGGGGTA?CAGACTCGCAACGGCGCCCGGCCGTGCCGCATAGGTCCTTGCGTAGAGCAGCGTAACATCTGCAGACACTCAAATT????????????????????????????????????????????????????????????????????????????????????????????????????????????????????????????????????????????????????????????????????????????????????????????????????????????????????????????????????????????????????????????????????????????????????????????????????????????????????????????????????????????????????????????????????????????????????????????????????????????????????????????????????????????????????????????????????????????????????????????????????????????????????????????????????????????????????????????????????????????????????????????????????????????????????????????????????????????????????????????????????????????????????????????????????????????????????????????????????????????????????????????????????????????????????????????????????????????????????????????????????????????????????????????????????????????????????????????????????????????????????????????????????????????????????????????????????????????????????????????????????????????????????????????????????????????????????????????????????????????????????????????????????????????????????????????????????????????????????????????????????????????????????????????????????????????????????????????????????????????????????????????????????????????????????????????????????????????????????????????????????????????????????????????????????????????????????????????????????????????????????????????????????????????????????????????????????????????????????????

**CLITE**_*Hirudo-Lumbriculus*

TGCGTCAGCCGCAAGAGAGCAGGAAAACTACACAATTGCCACACAAATCATTTCAGGCAGAGATCACACTACCAAGTCCATCCTTATATCTAAAATATATTTATCATCGATATACAATAACAGGTGCTCACAGATTTAACATTCTATTACACACCCTTCCAATTTATAGAAGATCAGCTGCAGAGAAAAAAATAATGACATCAGAAGTAATCTAGCACGCTACCTTCTGATCACTCCCCTTTAGCAGCATCCATCAGAGCCCACTATACATGTTATCTAGGTATACGTCTAGAACGAATTCTTATGTAAGCCGTAATAATAGTAATTACCCATATCCTGGATAAGACCATTATACACATGAACCAACCTCCCTTTCAGCTGTACTGCCAATCATCATATGACTTCATAGACCTCGATTACTTAGATTAGTTCGACTCATAGATAATAGATTACACGTCAAAAATCATTTGAAACACCAATGCAAGTTCAATATTCTACCATAAACTTAATAATATCACTCCTACATGTAATACACCACTAGTAACCCCCCAAGTAAGCGTTGCCTATCACATTACACATGATCACAAAACAAATAACCTTACTGCACGTTGATCAAAAGCAACTTCCTAATATAATAATTTTCAATCCAACTAGTTAACACATCT?????????????TTCACGGGCGCAACATCCCTCTCCCCGCGCCGCCAGTACTACACCATCCCCACTCTTCCGCTRCGCTTTGCGGTCCGCTGTGTCTGCGTTCAAGAACTGCCAACTCCCTCACTCCAGGGTGACGTGAGCCGGCTCGACGAGACCAACAGCGAGGCCCATCGAGACAAGAGAGTCTGCTCACGGACACAATAGAATGCTCATCGTCACCAGGCACACCCTGAAGCCAGTCCCAATGTCGTGTTCAAGGGATGGGTCAGACATGGAGGCCCCCAACATTGACCCAAAAGAGACCGACAAGCCCCCTCTCACGTTCGAGGATTAGGCCCCATGTTGTTCCGGCAACCACTACACGCAGTCAATCGACGTACGCGCGTTCAAGCCTGGAAGAGCTCAGAACCTCCCGCAACGGCCGTACCGTCTTAACBGCTGGAGCTCCGAACTCACAAAGGAGACCGAAGAGTCAAAGCGCCACAGCCTTCAGACAGCGCTATGCCACGCTCCCTGCTCCAGCCGTGAACCCTGGAGGATCCCTCGTTCCGAGAAGATTAGACAATAGCCAACGGGGCTCTGCGCTATGCGAGAGTGCACAAGCGAGCGTAGTCTTCGTCGAAATCCAACCTCGAAAGAAAGTCAGGGCGCCACCAAAAGCTTGCCACCGGCAGGCCGCTCAGGAGAATGAAGCCGCTCCCCGCGCGCTCCCAGACCACGCCTGCCCGAGCGCCCCCGGCAGCAGCCCCCTCCCACCCCCACCGCCCCTGTTTCCTCTTATGCTGCTAACACAAACTTTTACACAGTAGTGTTGGCACCCATGATTCTTAATCGTACATCCTACTTTTGTAACAACAAGCTCCGACCCCTGGGAAAGAGCCGTAGATCAAACAATCGGGTCCGTTGCTGGCTTTGAGTTCAGCTCAGCCGGAGTATTTCAGTTGTCCTCTTCGATGGTAAGTGACTTCTTACCATGTTTAAAGCCAAAACTACGCCCTAACAAGAAAACATACGGGACTCATTCGCCCGTATTAACCTTTCCTTTAAGTCTAGTTAGACTGTCTCGGGTCTAGACCGCGTTCGCCTCGCGGG?ACTGCGTTCTAACCTCCTCCCGGTTCTCTTGGGCTCGTTAGTCTTGGGTGGCCGGATCGTAATTACCAAAAGGTTTGCCTGAATAGTAGTAAATGTCTCGGTTCTATTTGTTTTTCGGAACCGAGGTAAGGGAAGACGGTCGTTACGGTTTTGTCGCCTAGAATACTAATCGAATTTTAAAAACATGTGCACGATAACTACGATCCCAAGATTGTTTCATGACCTTGCGGCAGCCTTCGACAAGTGTTGACCATATCGATTAGAATCCCCCCGCTTATTGGTGGGGCTCTAGCGCATTCATCGTGTCGATGGAACAAGTGCGTTTCACAGTTTCGCCAAGGAATGCATTGTTTCTGATCCTAGGCCGTCCTCTACCTCCTTCTATTGGGCTTATCCCATGGACAGGGATAGTCCTTTTGATGGTTTGTGATCCTCGTTGGTCCCGCGGGGGCAACCTTCGCGGTGTCGAAGACGATCACTTGATCATCA???CTAATATGCGGGCCAAACACTTTCTATGTAGACTGATGTGGTTATGAAATTTAAGCGCCATTATTAAATAAATAGTCATTAAGAGAACTTAAAAAAATTGAAAAGTATAGGCTTTACTTTAACTTAATAAT??????????????TATAGTCGGTGCGACCAGGGAT?????ATCATCTCTAAAACATAGAATATTTCTGCCAGATCATATTAAGCCTTAAGCTATTTTCCTTCAGACTTTAACAGGAAAGAGGCGCAGGAAATCCAAGTAACTTATCCTTAACAAACTAAGCTTATGCGGAGACGACCGCCCACGTCTGGGCGGCGGGAACGTGTTAGGACGACCCTCGC?GGAACGTTCGGACGTCCGGTCCTCCTGTTGGGGTTGCAGACTCGTCACACGTCTGAGCGTTT?TGCACGGGTCCTAGCGTAGAGCAGCGTAACATCTGCTGGCACTCGAATTAACACCGTTTAGAGGCGGAGAGGACGCAAAGTCTGCCGGTCGGTACCGGGCGGTC???GGACGAT??GCATCCCTAGCCGCGCCGGCGGGCGCACTTCGATCGGGAGCCCACGACAGTCTTGGCGGTCAAAACTCCGGAGAAGGACCATCGGCGTTGGTGTTATAGATCCGTGAGCGTTGGCCCGTCGACAGATTAGAGA???CCGCGCTCATTTCGGCTCGGTT?TCGGGTGGTCGAACTGGGGACTGTTCATGCCCTAACTGCATA??CGTCGGCGTACTCGGGGTCGGTGGCGTCGGTCGGTCACCTGTCAAGCTAAATTGCACGCAGGCTTTACGAAACCCGAAGTATGAGTGAAGGCCTTCCCCG?TGGCCTAGTAGTCCCGGCAGAGGGCCTACGCCGCTCAGCCGCCGTCGGCGAGGCGGACGACGTACACTTGGGTACCTATACACAAGCTGGTCCAGACAACTTGGTTAATGTCTGCGCTCGAAGAGTATCGTACCTGACGAACACATTAAGTCCGTGTCGATTACCGGGCAGTCGAGTGCAGCCCTACTCCTACACCGTTGATCTGATAATAATTTGTGAACACAACGCCGATCCGTGTCACTAGCTTCGGGTAGGACGTCGGGCATCGGGATGCCTCCGCGACGGGCAGTCCGCGGATTAGGCCTGGGCTAGCTGGGGCGCGCGGTCATACGGATATGTTGAACATCGATAGAGACTCGTTCTGAAACGGGGCAATGC???GCCCGTA?CCTATATCGTATCGACCGAGCACGGAGATGGC?CCTTGGGGTCCAGTGTACAACGAACTAGAGCTCGGCGAAATCCCGGACTTGAGGCGGACACTGTCGGCTTGCCGATGTCGTTGTTCCCTACCGCTCCCCGGGCGTGCACTTTCGTCGGCTAATCTAGAGACGGTGTGATTTCGTGCCGGACTGGACCATTG?TAACAGATCGTCATCGAGAGGCTGCCGTCTGGAGTCTGAGCGGCCTGGATTTGGCAGGGGCTGGTGAGATCGCAAGGTCAACTCGCCATCGTCAGGTCCTTCCCGTGGACGGCTCACTGGGGGCGGT??GGCCGCTTACTACGGCCGGGTCTAAAGCCAATTTAACAAATCCTGCGTTGCTGGTGTGATTCAAAAACGGGTCTAACGCAGAATTAGCTATCTGCCGACTTTTAGAGAATGAGAGCACGGGCAATTTTTTACGTTTCTTATTTATAAGCGGAGATAGGGCAAGTCCCGTCCTGGAACGCCCGACTCGGCCGGGCGCGTCCCTCTAAAGTGTCAGCGGATAGAGCTTGAAGCAGACGCTACACATCACTTTTTCGAAGTGTTAACTATTTATTAAGGATTATAACCTTTAATCGTCGTTAATCCCTCCCGTACATTTATTCTTGCTATAACAATTGGATTAGGATTGAA

**CLITE**_*Lumbricus*

TGGGTGAGCTGCAGGGGGGCCGGGAAACTTCTCTATTGCAAGACATTGCATTCCGGGCAGTGACCATATTACAACGTTCACCGTTGAACCTCGAAAGTCTTCACCGTTGACACTCTCCAACAGGCGCCCTTAGATTCACCTTCAAAACACTCACCCCTTCCATTCCCAGTTCCTCAGCTGCCGAGAGAAAAAAGAAGACCTCTGCAGAAATCTCGCCTGCCGCCATCTGATTACTTTCCCTTAGATGCATCCTTCAGAGCTATTTATACTCGTAATCCAGGGTTACGGCTAGAACGAATCCTCGTGTCAGCTGTACTAATGGTAGTCCCCCCCATCCCTGACTCGACAACAGCCCACATGAATCTATTTCATTCCCCGCTTGGTAATTAATCACCTC????????????????????????????????????????????????????????????????????????????????????????????????????????????????????????????????????????????????????????????????????????????????????????????????????????????????????????????????????????????????????????????????????????????AGGGTGGAGCATTCTCGCAAGTACTAAATCCCCCTCCCCGCGCCGCAAGTACTACACCATCCCGTGTCTCCCACTGATTTTCCCTGGTTGCTGCGTTTGTGTTTAAGAATTGCCTGCTCGCCCTCGCCTGGGCCACGTGAGCCAGCTCCATGAGACCAACAGCGAGGCTCTTTGAGGCAAGAGGTTCGCAGCACGGACACAATCGAGGTCTCGTCGTGGCCAGGCACACCTGGAAACCAGTCCCAATGTCGTGTTCAAGGGATGGATTACACATGGAAGCTCGCAACATCGACCCACAAACGACTGACAAACTCTCTCTTCTGTCCGAGGATTAGGACTAACGCTATAACGGCTATGACGACCTGCGGTCAACCGACGCGCGCGTGCCCAGGCTCCGAGGAGCTCAGTACTCCCCGCAACGGGGGCACCGTCTTAACTGCTGGCGCTCCGAATTCTCAAAGGAGACTGAAGAATCAAAGAGTCATCGCTAACAGACAACGCTTTGCGATGTCCCCTGCCCCAATCGCGAGCGCTGGAGGATCCCTCGTAGCGAGAAGCTGAGACAACTGCCAACGCGTCCACGCGCCACGCGAGAGTTCCCAGGCGAGTGTCGGCTTCTCCGAAACGCCGCGAAG????TACGTCCTAACCTTATCCCGTCTTGGTTAAGATTCCTGACCTTCTGCTCTTAGTCGCATGCCCAGTGGACTTAACTACCGCACTTGCTCATCGGTGATGAACTCGAGTTTGCTCGCACCTC???????CCTGTTTCCTCTTATGCTGCTAACACAAACCTTTACACGGTAGTGTTGGCACCCATGATTCTTAATCGTACATCCTACTTTCGTGACCACAAGCTCCGACCCCTGGGAAAGAGCCGTAGGTCAAACAATCGGGTCCTCTACTTGCTTCGAGTTCAGCTCTGCCGGAGTATTTCAGTTGTCCTCTTCGATGGTACGTGATATCCTACCATGATAAAGGCCAAAACTACGCACCGACAGGAAAACATACGGGACTCTTTTGCCCGTATTAACTTTTCCTTTAAGTACAGTTAGACTGTCTCGGGTCGTGCCAGCGTTCGCTTCGCGGG?ACTTCGGCGCGGCCTGCCGCCGGTTCGCTTGGGCTCGTTAGTCCTGCGCGGCCGACAGATAATTACCAAAAGGCTTGCTTGTATAGTCGTAGGTGCCTCGGTTCTATTTGTTTTTCGGAACTGAGGTAAGGGAAGACGGTCGTTACGGTTCTGTCGCCTAGAATACTAATCGAATTTTAAAAACATGTGCACGATGACTGCGATCCCGGACTCGCTTCATGACCCCGCGGCAGCCTTCGACAAGTCATGACCATAGTGATTAGAATCCCCCCGCTTATTGGTGGGGCTCTAGCGCATTCGTCGTGTCGGCGCAACAAGTGCGTTCTACAGTTTCGCCAAGGAGTACGTTCTGTTTGATCCAAGGCCGCCCGATACGTCCTTCTATTGGGCTTCTCCCATGGACAGGGATAGTCCTTTTGATGGTTTGTGATCCTCGTTGGACCCGCGGGGGCAACCTTCGCGGTGTCGAAGACGATCACTTGATCATCAAAGCAGATCCGCGGACCAAACACTTCCCATGTGGGCTAATGAGATTACGAAATAAAAGCGCCTAAGAAATATAAATAATCTCTAATAGGTATTAGATTAACTGAAGAGTATAGGCTTTATCACAATGAAAAAAT??????????????TAAGATCGGTGCGACCAGGGTTTATATATCATCCCTCATAACACGAATTTATCAGCCAGATCTAAACAAGCCTTAAGCTATCCCATTAAAGTCTTCTACAATGGGGTGGCGCAGGGTATCCATGT????????CTTCACAAACTAAGCTCATGCGGATACGACCGCCCCCGGCTGGCCGGCGGGAACGTGTCGGGACGACC?TCGT?TCGTCGGCCGGTCGCCCGGTCCTCCTGTCGGGGCCACAGACTAGTCACGCGTCTGGCCGGCG?AGCCGGGGTCCTCGCGTAGTGCAGCGTAACATCTGCTGGCACTCGAATTAACACCGTCTAGAGGCGGAGAGGACGTATGGTCCGCCCGTCGGTGTCGGACGGGA???CGGCCGC??GCATCCCTAGTCAGGGCGGCGGCCGTACTTCGGCGGGGAGCCCACGACGGTTCCGGCGGCGAAAACTCCGGGCAAGGACCTCGGCGCAGGAGCATACAGGCCCGTGAGCGTCGGCCCGTCGGTGGACCAGAGA??CCGTCGCGGGCGTCGACCCTGTT?GGTTGCGTTCGACTCGGGGACTGGCCATGCCCGGACCGCGCA??CGGCGGGACGCCTGGGGTCGGTGGCGACGGCCGGCAACCTGTCAAGCTAAATTGCGCGTTGGCGCTACGAAACCCGAAGCATGAGTGAAGGCCCGGTCTC?CGGCCTGGTGGTCCGGTCTCTCGGCCCACGCCGCTCGTTAGCCGTCTAAGAGGCGGACAACGTACACTTGGGTACCCATACACAAGCTGGTCCAGACAACTTGGTTAATGTCTGCGCTCGGAGAGTATCGCGCCTGACGAACTCACTACGTCCGTGTCGATTACCGGGCAATCGAGTGCAGCCCTACTCCTACAACGCTGATCTGACAATAATTTGTGAACACAACGCCGATCCGTGTCACTAGAGTCGGGTAGGACGTCGCGCATCCGCGACCCTCGGCGACGGGCATTCCGCTC?GTGGGGCTGGGCCAGCCGCGGGGCGCGGTCATACGGATATGTTGAACATTGATAGAGACTCGTTCTGAAGCGGGGCGATGTCGTGCCCGTA?CCTATACCGTATCGGCCGAGCACGGAGATGGC?CCTTGGGGTCCAGTGCACAACGAACCAGAGCTCGGCGCAATCCCGGACTTGAGGCGTAAGCTGTCGGCTTGTCGAGGCCTTTGTTCCCTACCGCTCCCCGGGCGTGCGCTTGCGTCGGCTAATCTGGGGACGGTGTGAATTCGTGCCGGAGTGGACCATTG?TAACAGATTGTCATCGAGAGGCTGCCGTCTGGAGTCCGAGCGGCCTGGGAGCGGCACGGGCTGGTGAGGCCGCAAGGTCGACCCGCCATCGTCGGGACCTTCCCGTGGACGGCTCACTGGCGTCGCTCGGTGCGTTCGCTTCGGCCGGATTTAAAGCCGATTTAACTAATGCTGCGTAACTGGTGTGATTAAAAAACGGATCTAACGCAGAATTAGCTATCTGCCGACTCTTAGAGAATGAGAGCACGGGCAATTTTTTACGTTTCTTATTTAGAAGCGGAGAGCGAGCAAGCTCCGAACTGGAACCGCCGACTCGGCCGGCGGCGTCCATCTAAAGTGTCAGCGGATAGAGCTTGAAGCAGACGCTACACATCACTTTTTCGAAGTGTTAACTATTTATTAAGGATTATAACCCTTAATCGTCGTTTATCCCTCCCGTACATTTATTCTTGCTATAACAATTGGATTAGGATTGAC

**CLITE**_*Stylaria*

AGAGTAAGCAGAAAATGAACAGGTAAAATACATAATTATATCTCAAAGCATTTCAGGTAGAGATCACATCACTTAGAACATCATTTAATCCTAAGTGTATTTATTATCGATATACTATAACAGGGGCACACAGTTTTAACATCAGAGTACTCACTCATATAATTCATAATTCATCTGCTGCAGAGAAAAGGTTAATGATTTCATTAGAAACCTAGCATGCTACCATCAGATAGCTTTACTTTAGCTGCTTCTTTTAGAGCTGACCATACTAGTAATATAGGAATACGTCTAGAACGAGTTCATACGTAAGCTGTAACACAAGTAATCTCTCTCATCACAGACAAGACTATCACATACACGAATCAATATCACTTCCAGCAATATTATCAATCATTATACAACATTACTGATAACGTCAATTTAGAATAATTTGTTTAATAGATAACTGAAATTATAGCAAAAATTATATACTACAACAACATAAACACATTATTCCATTGTAGAATTAGATTCATCTTTCATATATATTGTTCTTTACCAGTAACCCTATAAGAAGAAGATGCAATCCACGATAACCACGATTTTAAATTCTGTTACTCTACTCAACGATGAACTAAAGCTACTACTTAATTATATGTACTACCAT????????????????????????????????CCCACGGGAACTGCTTCCTCTTCACGGCACTGCCAATACTATGCAATTTTTTATCTCCCGTACAATTCTGCGGTTTGCTGTGTTAGCGATTCAGAACAACCTGTTCGCTTTCTCTTGAGCGACGTGTGCCGGCTCCATGAGACCAACAGTGAGGCCCTACACTACCAGAGACTCTGCACACGGATTCAATATATGCTGCATCGTGACTAGGCATTCCCTGAGCCCAGCCCGAATGGGTTGTTCAAGGGCTGGACCACACATGGAAGCTCTCAACATTGAGCCAAAAACCACTGACCGACACTCTCTCACGCCCGAGGATTAACATTCTTATAACCACGGTCATGGCCACATGAAGTTAACGGACCTACGTGTATTCAGGCTCTGAGGAGCTTCTTTCCTACCGAAACGTATGCACCGTCTAAACTGCTGGTGCTCCAAACCCTCTAAAGAAAGTGAAGAATCAAGGTGTCACCGCCTTCAGACAATGCTCTGCAACGTTCCATTCTCCAGCCGTGATCTCTGGAAGATTCCTCGTAGTGTGAAGCTTAGATAATTCAGAATGCGTCTTTGTGCTACGGGAGAGTTCACAGGCA?????????????????????????????????????????????????????????????????????????????????????????????????????????????????????????????????????????????????????????????????CCTGTTTCCTCTTATGGTGCTAACACAAACTTTAACACAGTAGTGTTGGTACCCATTATTCTTGATCGTACATCCTACTTTTGTAACAAGAAGCTCCGACCCTGGGGAAAGAGCCGTAGATCAAACAATCGGGCTCTTTGCTGGCTTTGAGGTCAGGTTACCCGCAGTATTTCAGTTGTCCTCTTCGATGGTACGTGATATCCTACCATGTTAAAGGCCAAAACTACGCCCCGACAGGAAAACATACGGGACTCATTCGCCCGTATTAACTTTTCCTTTAAGTCTAGTTAGACTGTCTCGGGTCCAGGCCGCGTTCGCCTCGCGGGTACTGCGTCCTGACCTCCTCCCGGTTCCCTTGGGCTCGCTAGTCTTGGGTGGCCGGAACGTAATTACCAAAAGGGACGCCTGAATAATCGCAGGTGCCTCGGTTCTATTTGTTTTTCGGAACCGAGGTAAGGGAAGACGGTCGTTACGGTTTTGTCGCCTAGAATACTAATCGAATTTTAAAAACATGTGCACGACAACTGCGATCCCGGGCTTGATTGATGACCCCGCGGCAGCCTTCGACAAGTTTTGGCCATAGTGATTAGAATCCCCCCGCTTATTGGTGGGGCTCTAGCGCATTCACCGTGTCGGTGTAACAAGTGCGTTTCACAGTTTCGCCAAGGAATGCGTTGTTTTTGGTCCAAGGTCGTCCGTTACCTCCTTCTATTGGGCCTGTCCCATGGACAGGGATAGTCCTTTTGATGGTTTGTGATCCTCGTTGGGCCCGCGGGGGCAACCGTTGCGGTGTCGAAGACGATCACTTGATCATCAGAACAGATCCGCGGGCCAAACACTTCCCATGTGGGCTAATGTGAAAACGAAGTAATAGCGCCGTAATCAAATAAACAATATTCCAGAGAGCTGAAAAAAATCACAGAGTATAGGCTTTATTTTTAATACACTAT??????????????AAAAATCAGTGCGACTAAGGAAA????ATCATCCTAAAATTATAGAACATGTCAGTCAGATCAAAATTAGCCTTAAGCTATCCTCCTGGAATCAATTATAGGAGGGTGGCGCAGGATACCCTGGTAACCTATCCTTAACAAACTCAGCCTATGCGGATACGACCGCGCGCGCCCGGGCGCAGCGAACGTGTTAGGACTAGCCATGC?ACGGCGGTACGGCGTCCGGTCCTCCTGTCGGGGTTGCAGACTCCTCACGCGCCGCACCGTCGCTGCCGCGGTCCTCGCGTATAGCAGTGTAACATCTGCAGACACTCGAATTAACACCGTTTAGAGGCGGAGAGGACGCAAAGTCTGCCCGTCGGTATCGGTCGCTC???GGTCTGC??GCATCCGCCG?CGTGGCGACGGGTGCACTCCGGCGAGGAGCCTAGGACGGTTCCGGCGGTCAAAGCCCCGAGGAAGGGCCGTCGGGTACGGTGTTATAGACTCGTGTCGTATGGCCCGCCTTCGGACCAGAGACGAGCGCGGCCCGCGTTG?CGACCT?GCGTCCGTTCGACTGGGGGACTGTCCATGCCCCGACCGCGTG??CGTTTTCGCGCTCGGGGTCGCTAGCGTCGGTCGGTAACCTGTCAAGCTAAATTGCGCAGTGGTGTTACGAAACCTAGAGTGTGAGCAAAGGTCGGTCCTCGCGGCTTGGTAGTCCCCCGTGGGGGCCTACGCCGCTCGGCCGCCGTCGGCGAGGCGGACAACGTACACTTGGGTACCTATACACAAGCTGGTCCAGACAACTTGGTTAATGCCTGCGCTTCGTGAGTATCGTACATGACGAACACATTAAGTCCGTGTCGATTACCGGGCGATCAAGTGCAGCCCTACTCCTACAACGTTGATCTCATAATAATTTGTGAACACAACGCCGATCCGTGTCACTAGCGTCGGGTAGGACGTCGAGCATC?GTGTACGTCTTCGACGGGCAGTCCGCGGAATAGGTCTGGGCCAGCTGAGGCGCGCGGTCATACGGATATGTTGAACATCGATAGAGACTCGTTCTGAAACGGGGCAATCC???GCCCGTA?CCTAAATCGTATCGACCGAGCACGGAGATGGCACCTCCGTGTCCAGTGTACAACGAAACTGAGCTCGGCGCAGTCCCGGACTTGAGGCGGACACTGTCGGCTTGGCGATGTCGTTGTTCCCTACCGCTCCCCGGGCGTGCACCTGCGTCGGCTAATCAGTGGACGGTGTTATTTCGTGCCGGACTGGACCATCGATAACAGATTGTCATCGAGAGGCCGTCGTCTGGAGTCCGAGCGGCCTGGGATCGACAGGGGCTGGTGAGGCCGCGAGGTCGACTCGCCAGCGTCGAGACCTTCCCGTGGACGGCTCACTAGCGGCGGTCTGGCCGTTCGCTTCGGCCGGGTCTAAGGCCAATTCAACAAATGCTGCGTAGCTGGTGTGATTCCGAACCGGATCTAACGCAGAATCAGCTATCTGCCGACTCTTAGAGAATGAGAGCAAGTGCCGGTTTTTACGTTTCTTATTTAGAAGCGGAGTCGGGGCAAGCCCTCGTCTGGAACGCAATGCTCGGTCGCGCGCGTCCCTCTAAAGTGTCAGCGGATAGAGCTTGAGGCAGGCGCTGCACATCACTCTTTCGAGGTGTTAACTCGTTGTCAAGGATTATAACCCTTGATCGTCGTTAATCCCTCCCGTACATTCACTCTTGCTATAACAATTGAATTAGGATTGAC

**COSSU**_*Cossura*

????????????????????????????????????????????????????????????????????????????????????????????????????????????????????????????????????????????????????????????????????????????????????????????????????????????????????????????????????????????????????????????????????????????????????????????????????????????????????????????????????????????????????????????????????????????????????????????????????????????????????????????????????????????????????????????????????????????????????????????????????????????????????????????????????????????????????????????????????????????????????????????????????????????????????????????????????????????????????????????????????????????????????????????????????????????????????????????????????????????????????????????????????????????????????????????????????????????????????????????????????????????????????????????????????????????????????????????????????????????????????????????????????????????????????????????????????????????????????????????????????????????????????????????????????????????????????????????????????????????????????????????????????????????????????????????????????????????????????????????????????????????????????????????????????????????????????????????????????????????????????????????????????????????????????????????????????????????????????????????????????????????????????CCGCCTGGCCAGAGCGCCGTCCCGAGCCGGCCAAGGCTCTGAAATCTCTGCTAACGGAGCAGCCCCCCGCGCGCTCGCCTACCGCGCCCCCCCGAGCTCCCCGGGTAGCGGTCTGCTCCCGCCCCCCCCGCT???????????TTATGCTGCTAATACAATCTTTTACCCAGAAATGTTAATAGTTATGGTCCTTAATCGTACATCCTACTTTTATAACAACAAGCTCCGACCTTCGGGGAAGAGCCTTAGATCAAACAATCGGGTCCTTTGCTGGCTTTGAGTTCAGCACAGCCGGAGTATTTCAATTCTCCTCTTCGATGGTACGTGATATCCTACCATGTTAAAGGCCAAAACTACGCCCTGACAGGAAAACATACGGGACTCTTTCGCCCGTATTAACTTTTCCTTTAAGCATAGTTAGACTGTCTCGGGTCCAGGCCGCGTCCACCTCACGGGTACTGCGTCCTGACCTCCTCCCGGTTCTCTTGGGCTGACTAGTCCTGAGTGGCCGGAACGTAATTATCAAAAGAATTGCCTGAATAATGGTAGGTGCCTCGGTTCTATTTGTTTTTCGGAACTGAGGTAAGGGAAGACGGTCGTTACGGTTTTGTCGCCTAGAATACTAATCGAATTTTAAAAACATGTGCACGACAACTACGATCCCCGGATTGTTTTATGACTCGGCGGCAGCTTCCGACAAGTCTTGGCCATAGTGATTAGAATCCCCCCGCTTATTGGTGGGGCTCTAGCATATTCACCGTGTCGGTG?AACAAGTGCGTTTCACAGTTTCGCCAAGGAATGCGTTGTGACTGATCCGAGGTTGTCCGTTACCTCCTTCTATTGGGCTTCTCCCATGGACAGGGATAGTCCTTTTGATGGTTTGTGATCCTTGTTGGTCCCGCGGGGTCGTCTCTTGCGGTGGCGAGGACGATCGCTTGATCATCA??????????????????AAAATTTCCTTCAAAGGCCTTTGTGCTTTCGCAAGCCCTACGCCTTATAATAATTGATAACTTTTAGGATGACCTAAATCCCTTGAAAAGTGCAGACTTAACTTATAAATTATTGGTGATATCATTAATCGTATGTTTGTGCAACAAAGGTAATTCTAACTTCCTGTATGTAAAATATAAATTTGTCAAAGCAACATAAGCCTCAAACTATCTATTTTTGGCCTTCGGAAAATAGGC????????????????????????????TAACAAACTAAGCCTATGAGGATACGACCGCCCCCTCCCAGGTGGCGGGACCGTGTTAGGAGAGCCTCTGCGGCGACC?TCGGGTGTCCGGTCCTCCTGTCGGGGTTACAGACGCGTCACGGCCCCCGTGGACGTCGCTTTGCTCCTTGCGTGGAGCAGTGTAACATCTGCCGACACTCGGATT????????????????????????????????????????????????????????????????????????????????????????????????????????????????????????????????????????????????????????????????????????????????????????????????????????????????????????????????????????????????????????????????????????????????????????????????????????????????????????????????????????????????????????????????????????????????????????????????????????????????????????????????????????????????????????????????????????????????????????????????????????????????????????????????????????????????????????????????????????????????????????????????????????????????????????????????????????????????????????????????????????????????????????????????????????????????????????????????????????????????????????????????????????????????????????????????????????????????????????????????????????????????????????????????????????????????????????????????????????????????????????????????????????????????????????????????????????????????????????????????????????????????????????????????????????????????????????????????????????????????????????????????????????????????????????????????????????????????????????????????????????????????????????????????????????????????????????????????????????????????????????????????????????????????????????????????????????????????????????????????????????????????????????????????????????????????????????????????????????????????????????????????????????????????????????????????????????????????????????

**CTENO**_*Ctenodrilus*

????????????????????????????????????????????????????????????????????????????????????????????????????????????????????????????????????????????????????????????????????????????????????????????????????????????????????????????????????????????????????????????????????????????????????????????????????????????????????????????????????????????????????????????????????????????????????????????????????????????????????????????????????????????????????????????????????????????????????????????????????????????????????????????????????????????????????????????????????????????????????????????????????????????????????????????????????????????????????????????????????????????????????????????????????????????????????????????????????????????????????????????????????????????????????????????????????????????????????????????????????????????????????????????????????????????????????????????????????????????????????????????????????????????????????????????????????????????????????????????????????????????????????????????????????????????????????????????????????????????????????????????????????????????????????????????????????????????????????????????????????????????????????????????????????????????????????????????????????????????????????????????????????????????????????????????????????????????????????????????????????????????????????CTATTACACCCAGGCGCCGCTCTGAGCCAGCCTAGGTTCTGAAAGTGCAGAGAGCGGAGCCGTGCCAGGCGCGCCCGGCCGCCGCGCCCGCCCGAGCGCGCCGGGCAGTGGCCCGGTCTTGCCCCCACCGCT????TTTCCTCTTATGCTGCTAATACATACTATCTCACAGTAATGTTAATAGCTATGGTCCTTAATCGTACATCCTACTTTTATAACAACAAGCTCCGACCT?ACGGGAAGAGCCTTAGATCAGACAATCGGTTCCTTTGCTGGCTTTGTGTTCAGCTCAGCCGGAGTATTTCAATTCTCCTCTTCGAAGGTACTCGATATACTACCTTGTTTAAGGCCAAAACTACGCCCTGACAGGAAAACATACGGGACTCTTTCGCCCGTATTAACTTTTCCTTTAAGTTTAGTTAGACTGTCTCGGGTCCAGGCTGCGTGCAGCTTAGGCGTACTGCGTCCTGACCTCCTACCGGTTCCCTTGGGCTGAGTAGTTCTGGGTGGCCGGAACGTAATTACCAAAAG?TTGGCCTGAATATTAGTATGTGCCTCGGTTCTATTTGTTTTTCGGAACTGAGGTAAGGGAAGACGGTCGTTACGGTTTTGTCGCCTAGAATACTAACCGCATTTTAAGAACATGTGCACGACAACTACGATCCCCGGATTGTTTCATGACTCGGCGGCAGCTTCCGACAAGTCTTGGCCATAGTGATTAGAATCCCCCCGCCTATTGTTGGGGCTCTAGCAAATTCACCCTGTGGGTGCAACAAGTGCTTTCTACAGTTTCGCCAAGGAATGCATTGTTCTTTGTCCAGGGCTGTCCTCTACCTCCTTCTATTGGGCTTGTCCCATGGACAGGGATAGTCCTTTTGATGGTTTGTGACCCTCGTTGGTCCCGCGG??CCTTTGGTCGCGGAGGCGAAGACGGTCACTTGATCATCAAAA???????????????GATACTTCCTTTGAAGGCCTTCGCGACCCCGAAAGCCTAACGCCAACTATAAAAGGACAATCTTTAAGAAAGCTTAACCAAATAAAAAAGTGTAGGCTTAACTCATACATCTCAACC??TTACAATCACCAAGAGTTTGCGCAGCAAGAGCAACAAAAACGTCTCCTTAAACCAACCTAAGTCAGCCGGAACTTACATAGCCTCAAACTATTTGTCTTAAATCACTAACAGACAAGT??????????????????????????????????????????????????????????????????????????????????????????????????????????????????????????????????????????????????????????????????????????????????????????????????????????????????TTAACAGCGCTTAGAGGTGGATGGGACGCAAAGTCGACCCGTGGATACCA????TTTCCCCTCGGTACCGGATCCTC?AGGGGGCCGGTGGGTGCACTTCCACGGGGAGCCCACGATGGTTCTGGCCGTCAAAGTGTGAGGGAAGAACCTGGGTGGGAGGTGTTATAGCCCTGACGTCATTGGC?CGCTGGCGGACCAGATA??????CGCCGGGCGAGGCCCGCTTGTCGTTCG????ACTGGAGGACTGTACATGCTCCGACTGCGTTTAGATTGGGTTGCGTAGGGTCTGTGGCGTCAGTCGGCACCCCATCAAGCTAAATTGCGCATGGGTTCT?A?CGAAACCAAGCATGAGTGAAGGCTTGCCTCGCAGGCCCAGTAGTCCCGCCTGTGGGTCTACGCCGCTCGTTCCCTG?TGGGGAGGCGGACAACGTACACTTGGGTACCTACATACAAGCTGGTCCCGGCGACTTGGTTAACTCCTGCACTCTTCGAATATCGTATATGACGAACACATTAAGCTCATGTCGATTACTGGGCACTCGAGTGCCGCCCTACTCCTACTACAAAGATCTCACAATAATTTGTGAATACAACGCCGATCCGTGTCACTAGGGTCGGGTAGGACGTCGGGCAATGGGGAGGCTCTGCGATGGGAGGTCCGCGGAATCGGCCTCGGCTAGCCGGGGCGCGTGGCCATACGGATATGTTGAACGCAGATAGAAACTCGTTCCGAAACGGGGAAATGAACGGCCCGTA?CCTATATCGTATCGACTGGACGCGGAGATGCCCTCTCGGGGGCCAGTGCGCAACGAACCAGAGCTCGGCGGGATCCCGGACTTGAGGCGGACTCTGTCGGCTTGCCGATGATGTTGTTCCCTACCGCTCCCCGGGCGTGCACTCCCGTCGGCTAATCTGGGGACGGTGTGATTTCGTGCCAGA?????????????????????????????????????????????????????????????????????????????????????????????????????????????????????????????????????????????????????????????????????????????????????????????????????????????????????????????????????????????????????????????????????????????????????????????????????????????????????????????????????????????????????????????????????????????????????????????????????????????????????????????????????????????????????????????????????????????????????????????????????????

**DINOP**_*Dinophilus-Trilobodrilus*

???????????????????????????????????????????????????????????????????????????????????????????????????????????????????????????????????????????????????????????????????????????????????????????????????????????????????????????????????????????????????????????????????????????????????????????????????????????????????????????????????????????????????????????????????????????????????????????????????????????????GGCGYYGCGGGAAATGTTAAATCAGAATAATCCGAAATACAGATTACCGTTACCACCTCACCCATAATACGAAGCTCCCATACTAGCTATCTACTCTACAATAAACTAGGTATTAGCCTTCTTTATTATTATTCTTTTTTAGTACTTTTGCAAGAAAAAGATGCCAATCTGATTTCTCATGATTTCTTTACTGGTAATTCTTCTCCTCGATGAACTAAAGCACTTTAATTACGAAACAATCTCCAACTTATTAAATARCAACCCCC???????????????????????????????????????????????????????????????????????????????????????????????????????????????????????????????????????????????????????????????????????????????????????????????????????????????????????????????????????????????????????????????????????????????????????????????????????????????????????????????????????????????????????????????????????????????????????????????????????????????????????????????????????????????????????????????????????????????????????????????????????????????????????????????????????????????????????????????????????????????????????????????????????????????????????????????????????????????????????????????????????????????????????????????????????????????????????????????????????????????????????????????????????????????????????????????CCTGTTTCCTCTTATGCTGCTAATACAAACTTTTGCACAGTAACGTTAATACACATGATTATTCATCATAAATCCTACTTTTGTAACACCAAGCTCCGACCTGGAGGGACGAGCCACAGACCAAACAATCTGGACTCGTGCTGGTTCC??GATCAGCTTCGCCGGAAGATGTCAGTTGTCCTCA??GACTTTGAGATAGACCTCAAAGTGATTAGGGCCACAATGACGCCCTGGCTGGAAAACACGCAGGACTCCATTGCCTGCGTTAACTTTTCCTTTAAGTTTAGTTAGACTGTCTCGGTCTCGGGCTCGGTCCAGCTTCTGTGTCTCGTATCCCGAACC??AACCGGCGTTTGCGGGCTCGCCAGTTCTAAATGGTCGGAAATCAATAATCAAAAGCCAAGCCTGAATTTTTGTAAGTATTTCGGTTTTATTTGTTTTTAGAAACTGAAATAAGAGAGGACAGTAATTACTTGCGTGTCCAATATAATACCAATCGAATTTTTAAAACATGTGCACGACAACTATGATCGCGGGCTTGTTATATGACCCTGCCGCAACTTCCGACAAGTTTTGGCTGTATTAATTAGAATCCCTCCGCTTATTGGTAGGGTTCTAGCACATTTGCCAACCGGTAATAACAAGTGCATTTCACGGTTTCACCAAAGCATACGTCTCTTATGCTCGGACGGTGCCCGTTACCGCTTTCTACTGAGCCTG?CTCATGGACAGGGATTGTCCTTTTAATGGTTTGTGTCCTTTGCTGAACCCGCAGGCACGGAGGCCGCGGTGTTAGGGATAGACACTTGACCATTAGGACTATAATGCAGACTTAACATTTTCTTTAAAGACTGATGTGGTTACACAATAATCACGACCTAGACCAATAAACTTACTTTTGGAATACCAAAAACTCATAAAGAGTGTAGGCTTTAGTTA?????????????????????????TTAACTTAATGCGATTATGGCATACAAAACTTCCATCACAACAAGCACAAATCTGCCAGGTTCAAATAAGCCTCATGCTTAATTCTTTAAGTCATTGACAAGATTTTGGCGCAACAATCTSAAGTACCYTGCG??CAACAAACTAAGCCTATGAGGTTACGACCGCCTCTGGTCGGTCAGTTGGAACGTGTTGGG?TGCTTTCAGTCCTTGTAGCCGTTCGGCTTATTCTCTTGTCGGGATTACAAAATTGCGCTGTCGAACGTTAGCTGCGGGTGGCGCCTAGCGTAGTGCAGTGTAGCATCTGCTTGCATTCAGATTAACATCGTTTAGAGGTGAACGGGACGCAAAGCCTCTCGGTCATTATC?GCGTCGGGATGCTTTCGGTGGACGGAC??GCCGGTTCGATGCTG?GCGCTTGTGGCGAGGCCACAACGGTTTTTTTCTTAATTGGCTGGAGTAAGAGGCCCGGGACGTGTGGTTGTCGGTTGTAGATTCCGTTCCTGCGCACGAACCAGAAAGGCGTCCTCCTCTTTTGGCACACTGATAGTTGGGTTGACCGTTAAACTGCGCATGTAGTGACCGCAGACGTGGGCTAGC?CTCAAGGTTAGTGGCG??AATCGGCACCCCGTCAAGGCAGATCGTGCGTAGGTTCTCAACGAGACCAAGAATGGGTAAAGGGCGGTCTCTCCGTCTAAGTGTTCCGAGTCTCCGGCACACGCCGCT???TCCCTGCCGGTTAGGCGGCCAACACGTACCTGCGTACCTATATACAAGCTGATCTAGACAACTTGGTTAACTCCTGTACCGTAAGAGTATCGTAAATGACAAATACATTATGTTCGTGTCGATTATCGAAAAATCGGGTACAGCCCTACTTCTATCAGTCTGATCTGAAAATAATTTGTGAATACAACGCCGATCCGTGTCACTAGTGTTGCATAGAGCGCCGTGCATTGGTACACTGCGTCGGTGGGAGGCCTGGGGTGTTGGCGTTGGCTAGCTCGGGCGCTCGGTCATATGGATATGTTGAACGTAGATAGATATTCGTTCTGAATTGGG?CAATGTTTGCCCCGTTTCCTATATCTTATAGACTGGACACAGATATTTCTCTTCTTGGACAAGCGTACACTGAACCGAAGTTCGGCGATGTCCTGGACTTGAGGTCGATTATGTCGGCTTGCCGATTACGTTTAACCCTAGCGGCCTTCGCGCGTGCACCGTCGTCGGCTAATTCGGGG?AAGTGTGATTTTGTGCCAGACTGGACCATCGATAACAGAATATCATCGAGGGACTGTCGTCTGTGATCAGAGCGTCTTGGGAGACTTAGGGACTGTCGAGACCCGGCGGTTGAATTGCTCTGG?TCGGTCCTTCTCGTGGAAAGTGCAGTGTTCTCGGTCGCGATGTTCACTTCGGCCGAATTTAAAGTCAATCCAACGAATGTTGCGGCAACGGTGTAATCAAAATCCTGATCTAACGAATAATCAGCTATCTGCTGACTTTTTGAGAATAAGAGCACGGGACAATTTTTACTTCGCATATATATGGGCGGAAGGCGGGCCGGTGCGTTTCGGGCGCGACTTTGTCGGTTGGCCAGGTCCCTCTATAGTGTCAGCGGATAGTCCTTGAAGCAGGCGTTACACATCACTTTTTCGAAGTGTTAACTATTTACTAAGGATTACAGCCATTGACTGTCGTTAATCCCTTCCGTACATTTATCCTTCCTATAAGAATCGGATTGGGATCGGC

**DORVI**_*Microdorvillea-Ophryotrocha-Parougia-Protodorvillea*

TGATTAAGGAGGTGTAGATCTGCAAAATTTTACAATTATGGGTCAAAGCCTTCCTGGTAGAGACCATATTACAATGAACCCCCTCTAATCCAAAGTATTATGGGGCTTGATACTGCTATAACGGGGCGCACAGGTTTCACATCATAATACCCTTCCTGTCATCTCATAGTTTATCAGCAGTTGAGAAAGGTTATATGCCCACCTAAGAAACTTGTCCCAGAACCATCTGATTTCTTCCCATTAGAGATTAGGACCGGAGCTATTCCTGTAATTCGCCTATCAATAACTTTAGAAAAGGGAAACCTGTTATCAATTGAAACTGCAGATATACTTGTCACTGTCATGGGGATTATACACTCGAATCAATTGCATTTCCGAAGAATTCCTTGACCACTATACCGCCCCGTAGCACATGTTAGCCCCGTACAATTTGCTTAATAGATAATAGTAACTATAACCAATAATATTTAATGCCTCTACACTAGCCCGATATTCTATTGTGAATATAATATTAGCTCTCATACATATTATACATCTCTAACTTTTCTTCTTCAAGAAGCTGCTACCCCGATTATTCATGATCCAAAAACAATTAGGATTACTGAACGTTGGGCTAAAACAACACCATAACACTACCTTCTCCAACCCATCAAGCA??????????????????????ATTGCGGGAACTACTTCCCCTGCTCGGTGCCACCAGTACTATGGAATCTTCAATCTTCCGCAAACTCTTGCGATGTGCTGAGTTTATATTCCAGAACAAACCGTTCGTGAACTCGTGGACTATGAGGGACGGCAACAAGAGCAAGACTCCCAGCCTCTTTGAGACATCAGAGAGCAACCACGGATCCAACAGATACTACATTGTAAATAGGGTTCCCCGGAGGCTTCAAGCAATGGCATGTTCAAGGGATGGGTTATAGTTTGATGCTTGCAACATCGACCCTCAAGAGCCAGAGAAGCACGCTCTATTGTACGTGGATTAAGAGACTGATCACGTGGCTCATGGGTACATGCATCCAATTGCAATAGGTGGGTCCAAGCCTGGAGTCTCTCTTCTCTTTCCGCAACGATGGGACAGACTAAACATGTGGAGCTCCGAATTCACCAAGCAGGCATCTGCATCAAGGTGACACCGCCTCCAAACCAGAAGATGCTAGTGTCCTGCTTCCAGCCAGGAACGTTGGAGGATATCTCGTAGCGCGAAGCTGAGAAGTGACCCTTTGCATCTTTGTGCCAAGTAACAATCCCTCAGCT?????????????????????????????CTATCACGCCATAACGTCGTCCTGAGCCAGCCAACGCTCTGCAAGTCCTGCTCTCGGAGCTACCCCAGATGCGCTCGCCTACCGCGCCCCCCCGAGCATCTCCGGTTCAAGTCTGATCCCGCCCTCCCCGCT????????????TATGCTGGTAATACACGCCTCGAAAAGGTGACGTTAATGGTCATGATTCTTAGTCGTACAACCCACATTTATAACAACGAGCTCCGACCTCCGGGGAAGAGCCTTAGGACAAACGATGGGGCCCCTTGCTGGCTTTGTGTTAAGCTCCGCCGTAGGACTTCAGTACCCCTCGTAGTCGGTAGGAGCTTTCCTACCGTGGTGAAGGCCAAAACTATGCCCTGGCAGGAAAACATACGGGACTCTTTCGCCCGTATTAACTTTTCCTTTAAGTTTAGTTAGACTGTCTCGGGTCCAGGCCGCGTTCGCCTCGCGGGTACTGCGTCCTGACCTCCCACCGGCTCTCTTGGGCTGACCAGTTCCGAGCGGCCGGGACGTAGTTATCAAAGAGTCGGTTCGAATCAGTGTAGGTGCCTCGGTTCTATTTGTTTTTCGGAGCCGAGGCAATGGAAGCCGCCCGTTACGGTTTTGTCGCCTAGAATGCAAGTGGAGTCCCAAAGACGTCCGCACGACGACTGAGGCCCACGGTTTGTTTCGTGACCCTTAGGAATCCCCAGACAAGTCTTGGCCATAGTGATTAGAATCCCCCCGCCTATTGGTGGGGTTCTGGCGCATCCGCCGTGCCGGCGCGACAAGTGCTTTTCACGGTTCCGCCAAGGAATGCTGC?CTCTTGGTCCAGGGCCGTCCGTTACCTCCTTCTATTGGGCTTCTCCCATGGACAGGGATAGTCCTTTTGATGGTTTGTGATCCTCGCTGGCCCCGCGGGGGCCGCAGCCGCGGAGGCGGGGACGATCACTTGATTATCA???CAGATCCGCGGAACAAACAATCTCCTTGTGGACTAATGTGATAATAAAAATTAATCAAATTTTAACAATAAATTACCTTTAGAAAGGTCTAAAAAAAATAAAACGTATAGGCTTTTTCTTAAAACA????????T??????????TAAGATTGGTGCAACCAAGGTAACTA?????TCCTTAAAACAACGTATAAATT????TAATAAATTTAAGCCTTAAGCTATCTTATTTTATTCACACACAATAAGGTGGCGCGAGATCACTTAGTAGCTAATC???TACAAACTAAGCTGATACGGAGACGACCGCCCCTGTCCCGGCAGCGGGAACGTGATAGGAAGCGACCTCCTCGAGGTCCCCAGCGTCCGGTCCTCCTGTCGAGGCTTCATGCTCGTCACGGCGCGGGACCTCTTGG??GACTCCCTTGCGTGAGACAGCGTAACATCTGCCGCCGGATGCATCCCCAGCGCTCAGAGTCTGACGGGACGCCAAGTCGACCCGCGGAAGTCGGGGCCAGGACCGTTCGCTCGGATC?????CTTTGC?GGTCGGCGTACTTCCGCGGGGAGCCCACGACGGTTTGGGCAGTCGAAGCCTTCGGGAAGAGCCCTCCTTTGGGAACTTACAGCCCGGAACGCGTTGGCCTGTCCAGGGACCAGTCATGCGCCCGCCCAGGCGCG??????????????????????GGCGTCTCCCGCAATAC???????????????????????AGCAGCCGCGGAGGACTCGGTCGGCA?CCCGTAAAGCCGAACCATGCGCGGGCTCTACGAAACCCCAAGCATGGGTGAAGGCGCGGACCTAGAGCTGAGTGGTCCCCTTCCGGGGCCCACGCCGCTCGTCCGTCGAGGCAGAGGCGGACAACATGCGCTCGGGCACCCATACACGGGCCGGTCTAGACGACTTGGTTGACTCCTGCGCTCGACGAGTATCGTACCTGTCGAAGACATTAAGCCCGCGCCGATTCGGCGGTCTTCGAGTGCAGCCCTACCCCTACTACGG?GATCTCACCATAATTTGCGAATACAACGCCGATCCGTGTCGGCAGGGTCCGGGAGGGCGTCGAGCATGAGGGTGCCTCTCCGACGGGAGGCTCGCGGAGCTGGTCTGGC?TAGCCGAGGCGCGCGATCATACGGATATGTTGGACGTAGATAGGGACTCGTTCCGAGTGCGAGAACTTGT??CGCAGTC??CTGTATCGCATCGACCGGTCGCGGAGAAGCCGCCGCGGGGCGTCGCGCACAACTCGTCAGAGCTCGCCGGGGTCCCGGATCCTAGGCGTACTCTGTCGGTTCGCCGAAGACGTGGTCCCCAGCCGCTCCTCGGGCGAGCACTCTCGGCGGCTAATCTGACCGCAATGCATCTCCGCGCCGGCCTGGACCATCGATAACAGATTGTCACCGAGGGACCGTCGTCTTCCTCGAGAGCGGCCCGGCACCGTGCGGTGCTGGCGAGGCTCGCGGGCCGACTCGCCTTCGGCGGGACCAACCCGTGGACCGTGCCCGGCGGGCGGTCGCGTCTCCGGCTTCGGCCGGGATCAAGGCCAATCTAACGTATGCAGCGAAACCGGTGTGATTCAGGTCCGGACCTGACGAACCATCGGCTAAGCGCTGGCATTTAGGGAGCGCGAGCAGGCGAAGTTTCGCGCGTTCCTGATTCG?GATGCGGAGCAGCGCGAGCCCGT??CTCGAACGCCGTCCTCGTGACCGCGCGTCCATCCGAAGTGCCAACGCTTAGCTCTCGAAGCAGGCGCCACACATCGCCCGTCCGAGGTGCCAACTGTTTATTAAGGATTACAGCCTCTGATCGTCGTTAACCCCTCCCGTACATTCAGCCTTGCCATCGCAGTTGGATTTGGAATGAC

**DORVI**_*Parapodrilus*

????????????????????????????????????????????????????????????????????????????????????????????????????????????????????????????????????????????????????????????????????????????????????????????????????????????????????????????????????????????????????????????????????????????????????????????????????????????????????????????????????????????????????????????????????????????????????????????????????????????????????????????????????????????????????????????????????????????????????????????????????????????????????????????????????????????????????????????????????????????????????????????????????????????????????????????????????????????????????????????????????????????????????????????????????????????????????????????????????????????????????????????????????????????????????????????????????????????????????????????????????????????????????????????????????????????????????????????????????????????????????????????????????????????????????????????????????????????????????????????????????????????????????????????????????????????????????????????????????????????????????????????????????????????????????????????????????????????????????????????????????????????????????????????????????????????????????????????????????????????????????????????????????????????????????????????????????????????????????????????????????????????????????????????????????????????????????????????????????????????????????????????????????????????????????????????????????????????????????????CCTGTTTCCTCTTATGCTGCTAATGCCGAAATCGAAACGGTGACGTTAATGGTCATGACTCTTAATCGTACATCCTACTTTTATAACTGCAAGCTCCGACCTC?GGGAAGGAGCCTTAGACCTAACGACGGTGCCCCTCGCTGGCTTTGTGCTCAGCTTCGCCGGAAGATTTCGGTTCCCCTCGTCGACGGTAGGAGCTTTCCTACCGTGCTAAAGGCCAAAACTACGCCCTGGCAGGAAAACATACGGGACTCAAACGCCCGTATAGAATTTTCCTACATGCCTAGTTGGACTATCTCGAGGCCGGCCTGCGTACGCCGCGAGGGTACTGTGCGCCGGCCTCCCGTCGGATGCCCCGAGATCGCCATTTCGGGCCGGCCGACGCGTAGTTATCAAAGGGTCTCCACGAATAGTTGTAGGTGCCTTGGTCCTATTTGTTTTGCGAGACCGAGGTAAGGGAGACCGCTCGTTACGGTTTTGCCGCCTAGACGGCAAGTGGAGCTCCCGAGACGTCCGCACGACATCTGCGATCCGCGGCTTGTTTCGTGACCCGCCGGCAGCTGCCGACTAGACTTGGCCATGGTGATTAGAATCCCCCCGCCTTTCGGTGGGGTTCTAGCGCCTCCGTGCTGCGCACGCGACAAGTGCGTTCCACGGTTTCGCAGCGGAATGCTCT???CGCGGTCCAGGGCCGCAGCCTAAGTCCGTGCCCTGGGCTCGTCCCACGGCCAGGGTTCGGCCTTTTGATGGCTTGTGATCCTCACTGGCCCCGCAGGGTCGCCTACTCCGGCGGCGGGGACGATCGCTTGGTGATCAGGA????????????????????????????????????????????????????????????????????????????????????????????????????????????????????????????????????????????????????????????????????????????????????????????????????????????????????????????????????????????????????????????????????????????????????????????????????????????????????????????????????????????????????????????????????????????????????????????????????????????????????????????????????????????????????????????????????????????????????????????????????????????????????????????????????????????????????????????????????????????????????????????????????????????????????????????????????????????????????????????????????????????????????????????????????????????????????????????????????????????????????????????????????????????????????????????????????????????????????????????????????????????????????????????????????????????????????????????????????????????????????????????????????????????????????????????????????????????????????????????????????????????????????????????????????????????????????????????????????????????????????????????????????????????????????????????????????????????????????????????????????????????????????????????????????????????????????????????????????????????????????????????????????????????????????????????????????????????????????????????????????????????????????????????????????????????????????????????????????????????????????????????????????????????????????????????????????????????????????????????????????????????????????????????????????????????????????????????????????????????????????????????????????????????????????????????????????????????????????????????????????????????????????????????????????????????????????????????????????????????????????????????????????????????????????????????????????????????????????????????????????????????????????????????????????????????????????????????????????????????????????????????????????????????????????????????????????????

**ECHIU**_*Arhynchite-Bonellia-Listriolobus*

??????????????????????????????????????????????????????????????????????????????????????????????????????????????????????????????????????????????????????????????????????????????????????????????????????????????????????????????????????????????????????????????????????????????????????????????????????????????????????????????????????????????????????????????????????????????????????????????????????????????????????????????????????????????????????????????????????????????????????????????????????????????????????????????????????????????????????????????????????????????????????????????????????????????????????????????????????????????????????????????????????????????????????????????????????TCCGCGGGTGCTACTTTCCCCTGTCGGCACCGCCAATACCATGGCATCTTCTCTCTTCCGCAACTTCTCGCGATGTGCTGTACACGTGTTCTAGAACCGCAAGCGCCCGCTCCCCACAGCCACACGCACCGGCAACAGGAGCCCAACAGCGATGCCCCTCAAGACAGCAAAGGAAGCCCACGGACCCAACCAAGGCTGGTTTGTGACTAGGCATACCGGGAATTGTCCGAGAATGGGCTGTACAAGGGCTTCAAGATTGTTGGATGCCCGTACCATCGATCCCCTCCAAGCCGAGAAACCCGCTCTGACGCCTGAGGATTATGCTTAAAATAATCATGCTTATGGTGACATGCGTCTAACACACCTGGGCGTGCCACCGCTCGCCAGAGCCCAGTTCTCCCCGCAACGCCCAAACCGAAAATATGGCCCGCGCTCCGAATTGCCCAAGGTCTGCGACAGCTCAAGGCGTCACCGCCTCACCACAGTGCTCTGCCAGTGTTTCGTCCCCAGCGGGGAAATCTGGCCGATGCCTCGTTCCGCGACCCTTAGAGAACCAAGGATCGGTCTTTGCAGTAGGGAACAGTGCCCAGACC?????????????????????????????CAAACACACCAGAGCGCCGTCCCGAGCCGGCCCCCGGCAAGCACGCCCTGCTCCCGAAGCGGCCCCCCGTGCGCCCCCCTGCCGCGCCCCCCCGAGCGCCTCGGAGAGCGGTCCGCTCGCGCCCCTCCCGCA?CTGTTTCCTCTTATGCTGCTAATACATACTTTTACACAGTAACGTTAATAGTTAAGGTCCTTAATCGTACATCCTACTTTTGTAAAAAGACGCTCCGACTGTTGGGGAAGAGCTTTACACCAAACAATCGGCTCCTCTGCTGACTTTGTGTTCAGCTCAGCCGGAGTATTTCAATTCTCCTCTCCGATGGTACGTGATATCCTACCATGTTAAAGGCTAAAACTACGCCCTGGAAGGAAAACATACGGGACTCTTATGCTCGTATTAACTTTTCCTTTAAGTTTAGTTAGACTGTCTCTGGTTTAGGCTGCGTTCGCCTCGCGGGTACTGTGTCCTGACCTCCTGCCGGTTCCGCTGAGCTGACTAGTTCTCGGTGGCCGGAACGTAATTATCAAAAGCTTTGCCTGAATATTAGTAGGGGCCTCGGTTCTATTTGTTTTTCGGAGCCGAGGTAAGGGATGACGGTCGTTACGGTTTTGTCATCTAGAATACTAATCGGATTTTAAGAACGTGCGCACGACAACTACGATCCCCGGCTTGTTCTATGACCCGGCGGCAGCTTCCGACTAGTTTTGGCCATAGTGATTAGGATCCCCCCGCTTATTGGTGGGGCTCTAGCATATTCGCCTTGCAGGTG?AACAAGTTAATATAACAGTTTTGCCAAGGAATGCGTTGTTCTTGCTCTCAGGGCGTCCGTTACCTCCTTCTATTGGGCTTATCCCATGGACAGAGATCGTCTTTTTAATGGTTTGTGATTCTCGTCGGACCCGCGGGGGTTACCGCCGTGTTGGCGAGGAAACTCGCTTGATCATTAGAA??????????????????????????????????????????????????????????????????????????????????????????????????????????????????????????????????????????????????????????????????????????????????????????????????????????????????????????????????????????????????????????????????????????????????????CTTCACAAACTAAGCCTATGCGGAGACGACCGCCCCTGCCGTGGCAGCGGGAACGTGTTGGGACGGCCTCTGGGTCGTGCGCGGCGAATCCAGTTCTCTTGTAGGGATTATAGAATCGCGGGGATCGCTGC?GTCGGCTCAT?GGTCCTTACGTGGAGCATCTTAACATCTGCGGTCGCTCGGATTAACAGCGTTCAGAGGTGGATGGGACGCATGGTC??CTCGCGGATACC?GCTGGGGCGCTGTCAG??CGGATCGCACGTGCTTGGGGCGGGCGCATTTTCGCGGGGA?CCCACGACGGTTCTGTCGGTCAGAGCCTGCAGCAAGGTACGCGGAGTCGGAGCTTATAGGTTGCCGGAGCTCGGCCCGACGGGGGACCAGATT???GCCCGCCGGGCGAGGCCTGTCT?GCGTCCGTTCGACTGGGGGACTGTTCATGCCCCGACTGCGTT??GGGCGGGGCTTTTAGGGTCAGTGGCTTCGGTCGGCACCTCATCAAGCTAAATTGCGCACTGATTGTACAAAATCTAAAGCATGGGTGAAGGCCCTCCGTTCGGGCCTAGTCTTCTCT????GGAGCAGATGCCGCTCGTACCCTGTGGTTGGGGCGGACAACGTACACTTGGGTACCTATACATAAGCTAGTCTAGACAACCTGGTTAACTCTTGCACTCT?TAAGTATCGTACCTGACGAACTCATTATGTCCGTGTTGATTACCGGGCGATTGAGGGCAGTCCTACGGCTACTACGAGGATCTGATAATAATTTGTGAATACAACGCCGATCCGTGTCACTAGGGTCGGGTAGCCCGAGAGGCATCGACGAGCCACCGTCTCGAGAGGCCTGCGGTTTGGACTTTGGCCAGCCGGGGCGCGCGGTCATACGGATATGTTGAACGCAGATAGAAATTCGTTCTGAAACGGGGCAATAAAT?GCCCGAC?ATTATATCGTATCGACTGGACACGGAGATGGCCCTTCGGGGTCCAGTGTACAACGAACCAGAGCTCGGCGGGATCCCGGACTTGAGGCGTACTCTGTCGGCTTGCCGATGACGTGGTTCCCTACCGCTCCCCGGGCGTGCACTCCCGTCGGCTGATCTGGGGATGGTGTTATTTCGTGCCGGGCTGGACCAT?GATAACAGATTATCATCGAGAGGTTGTCGTCTGGCGTACGAGCGGCGTGGAGTCGGCGTGGGCTGGTGAGGCCTCGGGGTCGACTCGCCGTTGTGG?GGCCTGCCCGTGGAGTTCCCACTGGCGGCATTCA??GTGTTCGCTTCGGCCGGATGAAAAACCAATTTAACAAATGCTGCGTAGCTGGTGTGATTAAAATCCGGATCTAACGCCGAATTAGCTATCTGCTGACTTTTAGAGAATGAGAGCACGGAACAGTTTTTACGTTTTTTATTTATGAACGGAGAATGGGCTTGCCCTTTCCTGGAACGCCCGCCTCTGGCGGGCGCGTCCATCTAACGTGCCAGTTGATAGAGCTTTAAGTAGACACTACACATAACTTTTTCGAAGTGTTAACTAATTATTAAGGATTATAACCTTTGATCGTTGTTAATCCCTCCTGTACATTCATTCTTGCTATAACAATTGAATTAGGATTGAC

**ECHIU**_*Urechis*

AGAACTAGGAGCCTTAGAACTTCTAATCTAAATAGCAACAGGGCATCTCGCTCCAGGTAGAGACCACCTCACTATGAACTCCATCCTATCCTAAATGTTTTTATGATTGACATAGTCTAACTGGGGCACTCAGATTCTTCATTAAAATACTCCCTCAGACCACTCACAGATCCTCTGCCCTAATGGAGGAAGACATGATCCCAGAGGAAATATGGCTTTCTCCCCTCAGATTACATTTCACTGGCTGTATCTATCAGAGCATATTATACCAGTAATTCGGGACTACGACCAGAACGAATACTTACGTAGGCTGTAGTCATAGCAGTCGCATACATCTCTGTCTAGCCAATTACCCACATGAACCGACTGCACTTCCTCAAAAACAGCCAATCACCTCACCACTCTGTGGAAAATGACAGACTTGACTGATTCGCAAAATAGATAACAGTCCCCACATCTCCCATCATATGATACCCCAATTCAAGCTCAATATTTCATAACCAACAAAATGTCATCTTTCTTTCACATCGTTCCCTCTTAGTACTCTCCCAAGAAAAAGATGCTAATCACATTAACCATGATTCCATCACCACTAACCCTACTAATCGCTGATCAAAAGTTTCTACATAATTTGACCAACCCCAACCTAACAGCTAAAAACACCTAGGGAGGTCCATTCTCGCGGGCGCTACTTCCTTCTCCCGGCGCTCCCAGTACTATGCCATCTTCTATCTTCCGCTTGTTTTTGCCATTTGCTGTACTTGTGTTTCAGAATCGCAAGTCCTCGTTCCTGCGGGACATATGTGCCGGCAACACGAGCCCAACAGCGAGCAGCTTCAATACAAGAGGCGAGGCTCACGAATACAACCAAGGGTTCATTGTGCTTCGGCATATCCGGAAAAGAGTGACAATGAAGTGTTTACTGGCTTCGTAACCGATGAATGCCCCTGCTATTGACCCATTCCCACCTGATAGGCCCTCCCTGCCGCCCGAGGCTTAGGTTCCTGATTATCATGCTTGTTGTCACCCGCATGCAACCCACCTGCGTGTGCCACTTCCTGCCCGAGCCGTGTACTTCCCGCAACGCTCGAACCGCAAGTATTGCTGGCGTTCCGAATTCACCAAGGCCACTGAAAACTCAAGGCGGCACCGCCTCAGCACTCCAGTACGCCAGTGTCCCGTCACCAGCCAGGAGACCTTACCGATGTCTCGTACTGCGACCCTTAGAGAACCCAGGCCGCGTCTCTGCTGCAGGAAACAGTGCCCAGGCCAGTGTTGGCTTCAACGAGAC?????????CTATCACGCCAGGGCGTCGCCCTGAGCCAGCCTTGGCCAAGCATCCCCTGCTCTCGGAGCCGCCCCCCACGCGAGCGGAAACTGCACCCGCTCGAGCCCCCCTGGTAGCGGCCTGCTCCCGCCCCCCCCGCT??????????CTTATGCTGCTAATACATACTTTTACACAGTAACGTTAATAGTTAAGGTCCTTAATCGTACATCCTACTTTTGTAAAAAGACGCTCCGACTGTTGGGGAAGAGCTTTACACCAAACAATGGGCTTCTTTGCTGACTTTGTGTTCAGCTCAGCCGGAGTATTTCAATTCTCCTCTTCGATGGTACGTGATATCCTACCATGTTAAAGGCTAAAACTACGCCCTGGAAGGAAAACATACGGGACTCTTATGCTCGTATTAACTTTTCCTTTAAGTCTAGTTAGACTGTCTCTGGTTTAGGCGGCGTTCGCCTCGCGGGCACTGCGTCCTGACCTCCTGCCGGTTTCGCAGGGCTGACTAGTTCTCGGTGGCCGGAACGTAATTATCAAAAGGGCAGCCTGAATATTGGTAGGGGCCTCGGTTCTATTTGTTTTTCGGAGCCGAGGTAAGGGATGACGGTCGTTACGGTTTTGTCATCTAGAATACTAATCGAATTTTAAGAACGTGCGCACGACAACTACGATCCCCGGCTTGTTCTATGACCCGGCGGCAGCTTCCGACAAGTCTTGGCCATAGTGATTAG?ATCCCCCCGCTTATTGGTGGGGCTCTAGCATATTCGCCTTGCAGGTG?AACAAGTTAATATAACAGTTTTGCCAAGAAATGCGTTGTTTTTGCTCTTAGGGTGTCCGTTACCTTCTTCTATTGGGCTTGTCCCATGGACAGAGATCGTCTTTTTAATGGTTTGTGATTCTCGTCGGACCCGAGAGGGTTACCGTCGTGTTGGCGAGGAAACTCGCTTGATCATTA???CAAATATGCGGACCAAACATTTCCTCTAGGGGCTGCTGCGATAACGAGAAAAAAGCGCCAGAGAACACTAACCATCCTTTAGGAAGACCTAAAAAATATAGAAAGTATCGGCTTTATTTACACTTTTCAAG??????????????AAAAATTGGTGCGACCCATGACCCAAAAACGTCTATCACTATAAGTACAAGTCTGCCAGATTA?AACAAGTCGTAAGCTATCCCCTCCTAGCCAACGACGAGGGGGGGGCGCAGGGCATCTCAGTAAC?TCCGCTTCACAAACTAAGCCCATGTGGGGGCGACCGCCCTCGCCGCGGCGACGGGAACGTGTTGGGACGTGCTCTGTGTCGTGCGTGGCCAGTCCAGTTCTCTTGTAGGGATTATAGAATCGCGGGGCTGGTCGT?GTCGGCGCAT?GGTCCTTCCGTGGAGCATATTAACATCTGCGGTCGCTCGGATTAACAGCGTTGAGAGGTGGATGGGACGCATGGTC??CCCGTGGATACC?TACGGGGCGGGGCCGCTCCGGATGGTACGTGTCGGGGGCGGGCGCATTTTCGCGGGGA?CCCACGACGGTTCCGTCGGTCTGAGCCCGTGGCAAGGTACGCGGGGGCGGAGCTTATAGGCCGCCGACGTTTGGCCCGTGTGGGGACCAGAATGATACCCGCCGCCTTGGGCTTTTCG?GCGTCCGTTCGACTGAGGGACTGTTCATGCCTCGACTGCGTCTCGGGAGGAGACATTTGGGTCAGTGGCTTCGGTCGGCACCTCATCAAGCTAAATTGCGCATGGACTTTACGAAATCTAAAGCATGGGTGAAGGCTTTCCGTTCGAGCCCAGTCTTCCCTCCGGGAGGCCGATGCCGCTCGTCCCCCGCGGGTGAGGCGGACAACGTACACTTGGGTACCTATACACAAGCTGGTCTAGACAACCTGGTTAACTCTTGCACTGG?AGAGTATCGTACCTGACGAACTCATTAAGTCCGTGTCAATTACCGGACGATCAAGGGCAGTCCTACGGCTACTACGAGGATCTGATAATAATTTGTGAATACAACGCCGATTCGTGTCACTAGGGTCGGGTAGCTCGAGAGGCATCCCAAAACCTCCGTCTCGAGAGGTCTGCGGATTTGACTTTGGCTAGCCGGGGCGCGCGGTCATACGGATATGTTGAACACAGATAGAAACTCGTTCTGAAACGGGACAATTTGTGGCTCTGACACTATATCGTATCGACTGGACACGGAGATGGCCCTTCGGGGTCGAGTGCACAAAGAACCAGAGCTCGGCGGGATCCCGGACTTGAGGCGTACTCTGTCGGTTTGACGATGACGTGGTTCCCTACCGCTCCCCGGGCGTGCACTCTCGTCGGCTAATCTGGGGATGGTGTTATTTCGTGCCGGGCTGGACCAT?GATAACAGATTATCATCGAGAGGTTGTCGTCTGAGGTACGAGCGGCGTGGAGTCGGCGTGGGCTGGTGAGGCCTCGGGGTCGACTCGCCGTTGTGG?GGCCTGCCCGTGGAGTTCCCACTGGCGGCCCTCGATCGGTTCGCTTCGGCCGGATGAAAAACCGATTTAACAAATGCTGCGTAGCTGGTGTGATTAAAATCCGGATCTAACGCCCAATTAGCTATCTGCTGACTTTTAGAGAATGAGAGCACGGGAGCGTTTTTACGTTTTTTATTTATGAACGGGAAATCGCGYAGCCCTTTACTGGAACGCCTTCT?CCAGYGAGCGCGTCCATCTAACGTGTCAGTTGATAGAGCTTTAAGCAGGCGCTACACATAACTTTTTCGAAGTGTTAACTAATTATTAAGGATTATAACCTTTGATCGTTGTTAATCCCTCCTGTACATTCATTCTTGCTATAACAATTGGATTAGGATTGAC

**EUNIC**_*Eunice-Marphysa*

TGAATCAGGAGACTCAGTACCTCAAAAATTCTCGATAATAGGCCAGCTCCTTCCTGGTAGAGATCACATTACTACGCTCTTCATTCTACCTAAAGTATTATAATAATTGGCACCCTTCTGTAGGCGCTCATGGTTTTCGAACTCTAACACTCTCCCTGTCTATTCCCGGTTCTTCTGCCATCGAGAAAAATAAAATGTCCACTTCAGAAATATTGCTTGCTCCCTTCAGATAACATCCCGCTAGAGGTATCCACTAGTTCAACCTATACATGTAGCATGGGAATACGATTAGAACGAGTTCCTATGTCAGCTGTAGTTACTACCGTCTCTCTCCTCTCAGCTATGACTACTACCCTCGTGAATATACCGCCCTTCCTGCAAAACCGACATTCACTATGCCACTCTACAGCCAACGACTCATTCTCCAAACTAACCAAATAGACCACTGTCCTCACAAGCCGCGTAATACCCTACTCCTGTCCAAGTTCGCCATGCCTTTATAATTAACACAACGTCACTCCTTTATATTATGCACCCCTTGTACCTCTCCCAGAAACTATTGCCAGCCAGAATACACATGAACTCTTCACTCCTAGTCTACCCCCCCGCATAGCAAAAGCCAGGAATTAGCGAGACCA???????????????????????????CTGGCAGTCCGTCGTCATGGGTGCTACTTCCTCTGCTCGATGCCAGCGGTACTATGGCATCTTCAATCTTCMGCCTTCTTTCGTGATCTGCTGTACTAGTGTTCTAGAATCATCTGCTCGCGTACCCTAAAGCCATGAGGACCGGCAACATGAGACCAACAGCTCGAATCTATGAGATGTCAGAGAGCTCCCACGAATGCAATCGAAACTTCATTGTGATTAGGCATTCCTGGAAGGTTCCACAAATGACCTGTTCAAGGGATGGAAGACCTCTTGAGGCTTGTCAAATTGATCCATAAGCTCTTGACAAACCCGCTTTGACGTACGTGGATTAAGTGTCTTGTCAAACGGCATACGGTCAAATGTCTCTAACATACCTGGACGGGCCACCGCTCGGATGAGCTCTGTACTGTCCGAAATGGTCGTACCGTCTAAATCTGCGGGGYAGTGGATTCTCATTTGAGACCAAGGAGTCAAAGCGTTACCGCCTTCAGACCAGGCTATGCATGACCCCTGTCCCTAACAGGGAGCCCTGGAGGATTTCTCGTAGCGCGAAGCTAAGACAATTGCTCATGAGTCTGTGTGCCACGTAACAGTGCCCAGGCTAGTGTAGTCCTCAACCAGACCCTCCGTCGAAACCACGCCAGGGCGCCGATCCGAGTCAGTTAACGCTCTGCAAGCCCTGCTAGCGATCCTACTCGCCGCGAGCCCGCAAGTTGTGCTCCCACGAGCTTTCCGGGTAGTGGTCCGTTCCTGCCCTTCCTGCT????????????TATGCTGCTAATACAAGCCTGAACATGGTAACGTTCATAGTTATGGTCCTTAATCGTAGGTCCTACATTTATAACAATAAGCTCCGACCTCACGGGAAGAGCCGTAGATCAGACGGCCGGGCTCGTTGTTGGCTTTGAGTTCAGCTCAGCCGGAGTATTTCAATTCTCCTCTTCGTTGGTAAGTGCTATCTTACCATGTTAAAGGCCAAAACTTTGCCCTGGCAGGAAAACATACGGGACTCTTTCGCCCGTATTAACTTTTCCTTTAAGTTTAGTTAGACTGTCTGGGGCACAGGCGTCGTCCACCTCGCGGGTACTGGTGCCCGGCCCCCTTCCGGTTTCCTTGGGCTGACTAGTCCCGGGCGGCTGGGACGTAATTACCAAAATCTGAGACTGAATATTGTTAGGCGCCCCGGTTCTGTGTGTTTTTCGGAA?TGAGGTAAGGAAAGACGGTCGTTACGGCGGTGTCGCCTAGAATACTAATCGAATTTTAAAAACATCTGCACGACGACTGCACTCACCGAATTGTTCCATGACCCGGCTGTGGCCTCCAATCAGTCTTGGCCATAGTGATTAGAATCCCTCAGCTTATTGGCAGGGCTCTAGCACATTCGCCGTGTCGGCGTAACAAGTGCGTTTCACAGTTCCGCAGATGAATACGCT???ATTGGCCCAAGGTTGG?CGCTACCTCATCGTTTTGGGTTTATCCCATGTACAGGGATAGTCCTTTTGATGGTTTGTGATCCTCGTTGGTCACGCGGGG??CAACACTGCGGTGGCGAGGACGATCACTTGATCATCA??????????????????AAAATTTCCTTTGGAGGCTGTTGTGGAAACGAGAATAAACCGCCACACAGATATAATCTATCTTCAGGAGAGCCTGAACACTATAAAAAGCGTCGGCTTTAGTTTACTCTTATAGTTAATTATACTTCCCGTAACTTTGTGCGACAACGGTAACTC?ATCATCCGACAAACCACATTTTAATCAGTCAGACTTTAATAAGCCGCAAACTATCTCCCCCCGGCCAACTACGGGGAGGCGGCGCAAGGTTCTCTGGTAACCAACCCTCCAGAAACTAAGCCTATACGGTGACGACCGCCCCTGCCCTGGTAGCGGGAACGTGATAGGACAATCTCTGGGGTGG?CTTCGGGTGTCCGGTCCTCCTGTCGGGGCTTCATGCTCGTCAGGGCGCCTGTCGCTGCCCCTTGAGTCCTTGCGTAGAGCAGTGTAACATCTGCTGGCACTTAGATTACAGTCGTTTAGAGGTGGATGGGACGCCAAGTCAATCTGCGGGTACCGGCGACCGCAGGCGATGTCCGGATTGT?CGTGCTGGCGGAGGGTGCACTTCCGCAGAGAGCCCACGACGGTTCCGGCGGCATAAGCCCGGGAGAAGGGCTTCTTGCAATTGCTTTATAGCTCCTGTGGTCATTGCCCGCTGCTGGACCAGTCACGTGCCCGCCCGGCTCGGCTGGCCCGCTGGGCGTTCGACTGGTGGACTGATCATGCTCCGACTGCGGATGGGTTCAGGCACACAGGGTCAGTGGCGTAGGTCGGCACCCTATCGAGCTAAATTGCGCATTGGACTCACGAATCCTAAAGCATGAGTGAAGGCTGGCCTCGCCGGCCTAGTGTTCCTGCACGTGGGCACACGCCGCTCGTCCGTCGTCGGGGAGGCGGACAACGTACCTTTGGGTACCTATTCACAAGCTGGACTAGACAACTTGGTTAAGTCCTGCACTCATCGAGTATCGTACCTGACGACTACATTAAGCACGTGTTGATTATCGTGCAATCGTGTGCTGCCCTAATCCTGCTACGATGATCTGATAATAATTTGTGAATACAACGCCGATCCGTGTCACTAGGGTCGGGTAGGACGTCGGGCATGGGGGTGCCTCCTCGACGGGGCGGCCGTGGAATGGGCCTTGGCTAGCTGGGGCGCACGGTCACACGGGTGTGTTGAACGTAGATAGAAACTCGTGCTGAGTTGGGGCTTGGGTGGGCCCGCATCCTATATCGTATCGACCGGCAACGGAGATTGGTCTTCGGGGCCCAGTGTACAACAAACCAGGGCTCGGCGGGATCCCGGACTCTAGGCGGACTCTGTCGGTTTGCCGATGACATGGTACCCCACCGCTCCCCGGGCGTGCACTCCCGTCGGCTAACCTGGGGAGAGTGTGAATTCGTGCCGGACTGGACCATCGATAACAGATCATCATCGAGAGGTTGCCGTCTGGGGTACGAGCGGCCTGGAACTGGCCTGGGCTGGCGAGGCGTGGGCGCCGACTCGCCGTGGTCGGGACCTTCCCGTGGAGTGTTCACTGGCCGCCGTTCGGCGGTTGGCTTCGTCCGGATCCAAAACCGATTTAACAAATGCTGTGTCACTGATGTGATTCAAATCCGGATCTAACGCAAAATTAGCTATCTGCTGACTCTTAGAGAATGAGAGCACAGGCAATTTTTTACGTTTCTTATCTATAAGCGGAGAGCGGGCAAGCCCTCGCCTGGAACGCCCGAGTCGCTCGGGCGCGTCTCTCTAGAGTGTCAGCGGATAGTGCTTGAAGTAGACACTACACATCACTTTTTCGAAGTATTAACTGTTTATTAAGGATTATAACCCTTGATCGTCGTTAATCCCTCCCGTACATTTATTCTTCCTATAAGAATTGAAT??????????

**EUPHR**_*Euphrosine*

??????????????????????????????????????????????????????????????????????????????????????????????????????????????????????????????????????????????????????????????????????????????????????????????????????????????????????????????????????????????????????????????????????????????????????????????????????????????????????????????????????????????????????????????????????????????????????????????????????????????????????????????????????????????????????????????????????????????????????????????????????????????????????????????????????????????????????????????????????????????????????????????????????????????????????????????????????????????????????????????????????????????????????????????????????????????????????????????????????????????????????????????????????????????????????????????????????????????????????????????????????????????????????????????????????????????????????????????????????????????????????????????????????????????????????????????????????????????????????????????????????????????????????????????????????????????????????????????????????????????????????????????????????????????????????????????????????????????????????????????????????????????????????????????????????????????????????????????????????????????????????????????????????????????????????????????????????????????????????????????????????????????????????????????????CGGCACCCAGAGCAAGTCTCGGCTCTGATATCTCTGAACTCGGAGCAACGCCCCGTGGACTCGTCTACCGCGTCTGAACAAGCTCTTCTAGTAGCAGACCGCTCCCATCCTCCCCGCT????????????????????TAATACAAACTTTTACACAGTAACGTTAATAGTTATGGTCCTTAATCGTACATGTTACTTTTGTAACGAGAAGCTCCGACCTTCGGGGAAGAGCCTTAGATCAGACAATCGGGTCCTTTGCTGGCCTTGGGTTCAGCTCCGCCGGAGTATTTCAATTCTCCTCTTCGATGGTACGTGATATCGTAACATGTTTAAAATCAAAACTACGCCCTGGCAGGAAAACATACGGGACTCTTTCGCCCGTATTAACTTTTCCTTTAAGTTTAGTTAGACTGTCTCGGGTCCAGGCTGCGTCCGCCTCGCGGGTACTGCGTCCTGACCTCCTCCCGGTTCCCTTGGGCTGACTAGTTCTGGGTGGCCGGAACGTAATTATCAAGAGACTTGCCTGAATAATGGTAGGTGCCTCGGTTCTATTTGTTTTTCGGAACTGAGGTAAGGGAGGATGGTCGTTACGGTTTTGTCGCCTAGGAGACTAATCAAATTTTTAAAACATGTGCACGACAACTACGATCCCCGGATTGTTTCATGACTCGGCGGCAGCTTCCGACAAGTTTTGGCCATAGTGATTAGAATCCCCCCGCTTATTGGTGGGGCTCTAGCATATTCGCCGTGTCGGCGTAACACGTGCGTTTCACAGTTTCGCCAAGGAATGCGTCGTTACTGGTCCGAGGTTGTCCGTTACCTCCTTCTATTGGGCTTCTCCCATGGACAGGGATAGTCCTT???????????????????????????????????????????????????????????????????????????????????????????AAAGCTTCCTTCAAAGGCTTTTGTAACCACGTGAGCTTAGCGCCCTACGCACAGATCTAACCTTTATGAAGGCATTAAATAATTAAAAAGTGTCGGCTTAATTGTAACTTTGAGAAAAAATAATCTCACCCTCAATTAGTGCAACACAGGCATATAAACCTTCCTTACAATATCTTTTATCTCCAC?GAACTTATAAAAGCCGCAAGTTATTTTTCTCGAATAACTAACAGAAAAGT????????????????????????????CCACAGACTCAGCCTATGCGGAGACGACCGCCCACGCCTGGGCGTCGTGAACGTGTAGGGACAGCCTCTGGGGCTGTGTACCGGTGTCCGGTCCTCCTGTTGGGGCTACAGGCTCGTCACGGCACTAGTCGCAGTCTCTAAGGTCCTTGCGTTGCGCAGAGTAACATCTGCTGACACTCAGATT????????????????????????????????????????????????????????????????????????????????????????????????????????????????????????????????????????????????????????????????????????????????????????????????????????????????????????????????????????????????????????????????????????????????????????????????????????????????????????????????????????????????????????????????????????????????????????????????????????????????????????????????????????????????????????????????????????????????????????????????????????????????????????????????????????????????????????????????????????????????????????????????????????????????????????????????????????????????????????????????????????????????????????????????????????????????????????????????????????????????????????????????????????????????????????????????????????????????????????????????????????????????????????????????????????????????????????????????????????????????????????????????????????????????????????????????????????????????????????????????????????????????????????????????????????????????????????????????????????????????????????????????????????????????????????????????????????????????????????????????????????????????????????????????????????????????????????????????????????????????????????????????????????????????????????????????????????????????????????????????????????????????????????????????????????????????????????????????????????????????????????????????????????????????????????????????????????????????????????

**FAUVE**_*Fauveliopsis*

??????????????????????????????????????????????????????????????????????????????????????????????????????????????????????????????????????????????????????????????????????????????????????????????????????????????????????????????????????????????????????????????????????????????????????????????????????????????????????????????????????????????????????????????????????????????????????????????????????????????????????????????????????????????????????????????????????????????????????????????????????????????????????????????????????????????????????????????????????????????????????????????????????????????????????????????????????????????????????????????????????????????????????????????????????GTCATGGGTGCTGCTTCCTCTGCACGGTGCTACCGCTACTTCACCATCCTCCATCTTCCGCGACCTTTGGTGGTGTGCGGCACAAGCGGACCAGAACCGTCCGTCCGTGTTCACCTGGGAGACACGAGCCGGCTCCACGAGCCCAACAGTGAGTCTCCTCAACGTGTCAGGGAGCGCTCACGGATCCAATCAAGAACGGGTTGTGCCCTTCCAGACCAAGAGGGCTCCCCCAATGACGTGTTCAAGAAGTGGGTTATCGTTTGAGGCTCGCAGCATAGAGGAACAAGAACCAGACAAGCACCAGCTGAGGTGCGCGGATTGAGCGCCTGGTTATCCGGCCTATGGGGACCTGCGGCCGCAACACCAGCGTGGGCCACTGCTCCGAGGAGCTGCAATCCTCCCGGAATGGTGGCACCGTCTCCACCGCCGGAGCTCCGAACCGTCCAAGGAGTGCGCAGACTTACTGAGGCACCGCCAGCAGACCAGGCTCCGCGCGCCCCCCCTCCTCAGCAAGGAACGAAGGAAGTGCTCCCGTAGCGCGAAACTGAGACAACACAGAGCGGGAACCCGCGCTACGTCTCAATCCCCAGACC?????????????????????????????????TACGCCAGAGTGCCGATCTGAGTCAGCTTTGGCCAGGTATTCTCTGAGAGCGGAGCAGTGCGAAACGCGCTCGCCTGTAGCGATCGCACGAGCAAGTCGGGATCTGGTCCGTTCTCGCCCC??????????????????????????????TACACACCTTTACACGGTAACGTTAATAGTTATGGTCCTTAATCGTACATCCTACTTTTGTAAAAACAAGCTCCGACCTTCGGGGAAGAGCCTTAGATCAAACAATCGGGTCCTTTGGTGACTTTGGGTTCAGCTAAGCCAGAGTATTTCAATTCTCCTCTTCATTCGTATGTGATATCTTACGAAGTTTAAGACTAAAACTACGCCCTGGCAGGAAAACATACGGGACTC?TTCGCCCGTATTAACTTTTCCTTTAAGTCTAGTTAGACTGTCTCAGGTGCAGGCAGCGTCCACCTCGCGGGTACTGCGTCCTGACCTCCATTCGGTTCCCTTGGGCTGACTAGTTCTGGGTGGCCGAAACGTAATTATCAAAAGAATTGCCTGAATAATGGTAGGTGCCTCGGTTCTATTTGTTTTTCGGAACTGAGGTAAGGGAAGACGGTCGTTACGGTTTTGTCGCCTAGAATACTAATCGAATTTTAAAAACATGTGCACGACAACTACGATCCACGGATTGTTTCATGACTCGTCGGCAGCTTCCGACAAGTTATGGCCATAGTGATTAGAATCCCCCCGCTTATTGGTGGGGCTCTAGCATATTCACCCTGTGGGTGTAACAAGTGCTCTTCACAATTTCGCCAAGGAATGCGTTGTCACTGGTCTGAAGTTGTCCGTTACCTCCTTCTATTGGGCTCATCCCATGGACAGGGATAGTCCTTTTGATGGTTTGTGATCCTTGTTGGTCCCACGGGGGCAACTGCCGTGGTGGCGAGGACGATCACTTGAT?ATCA??????????????????AACATTTTCTCTAGAGACTTTTGTGATAACGAAGGCCCTACGGCGAGTAAAAATAAATATTATTTAGGAGAACCTAAATGATAAAAAAAGTATTGGCTTTACTCTTACTAAAATTATACA?ATAATTATACAGAGTTGGTGCGACCAAGGTTTATATTTCTTCCTTTTCTATAAGAATACACCTGCCAGATCAAAATAAGTCATAAGCTATCTTTCTCAAGCCAATGACAGAAAGGA??????????????????????????CTTCACAAACTAAGCATATGTGGACATGGCCGTCCCTGCCGAGGCAGTGTGAACGTGTTGGGAGAGCCTGTGCGGCGAACGTCTGACGTCCGGTCCTCCTGTCGGGGCTACAGACTCGTCACGCCTCAGAC?TTCGTCTCTTGACTCCTTGCGTGGAGCAGTGTAACATCTGCAGACACTCGGATTAAAAACGTTTAGAGGCGAATGGGACGCAAAGTCGACCCGTGGATACTACGCCTGCGGGGTGGAAGTCGCATCGTAGGTCCAGGCTGTCAGTGCACTTCCACGGGGAGCCCACGACGGTTCCGGCTGTCAAAGCTCCGGGGAAGAGCTCCTCGGGGGAGTGTTATAGACCCG?GTGAGTGGGCCGGCTGTGGGACCAGACT????CCCGCCGGCAGGGGTCCGCTCTCCGACCGTTCGAATTGAGGACTGTACATGCTCTGACCGCGGAAG?GACTCGGCGCTCAGGGTCAGTGGCGTCGGTCGGCACCCTATCAAGCTAAATTGCGCATTGACTCTACGAAATCTAAAGCATGAGTGAAGCCCGGTCTTAATGGGCTAGCGGTCCCGCCTGCGGGCCCGCGCCGCTCGTCTGCCGTCAGTGAGGCGGACAACGTACACTTGGGTACCTATTTACAAGCTGGACTAGACAACTTGGTTAACTCCTGCGCTTTTCAAGCATCGTACCTGCCGAACACATTGAGTCTGTGTTGATTACCAGGCGATCGAGTGCTGTCCTACTCCTACCACGAGGATCTCACAATAATTTGCGAATACAACGCCGACCCGTGTCACTAGGGCCGGGTAGGACGTTGGGCATCGGGGTGGCTCCGCAACGGGAGGTACGTGGGTCTGGCGTTGGCTAGCTCGGGCGCACGTTCATACGGATATGTTGAACGTAGATAGAAACTCGTTCCGAAACGGGGCAATGCCGCGCCCGTA?TCTATATCGTATCGACCGGACACGGAGATGGCGTTTCGGCGTCCAGTATATAACGAACTAGAGCTCGGCCAGACCCCTGACTTGAGGCGGACTCTGTCGGCTTGCCGATGACGTTGTTCCCTACCGCTCCCCGGGAGTGCGCTTTGGTCGGCTAATCTAGGGACGGTGTGATTTCGTGCCGGACTGGACCAT?GGTAACAGATTATCATCGAGAGGCTGTCGTCTGGGGTACGAGCGGCCTGGAACTGGTAGGGGCTGGTGAGACTTTTGGGTCGACTCGCCGTCACCGGGACCTTCCCGTGGACTGCTCACTGGCGGCTTTCG?GGAGTTCGCTTCGGCCGGGTCTAAAGCCGATTTAACAAATGCTGCGTAACTGGTGTGATTAAAATCCGGATCTAACGCAGAATCAGCTATCTGCCGACTTTTAGAGAATGAGAGCAC?GGAAATTTTTTACGTTTCTTATTTAAAAGCGGAGAGTGGGCAAGCCCTGTCCTGGAACGCCCGGCTCGGTCGGGTGCGTCTCTCTAAAGTGTCAGCGGATAGAGCTTGAAGCAGACGCTACACATCACTTTTTTGAAGTGTTAACTATTTATTAAGGATTATAACCTCTGATCGTCGTTAATCCCTCCCGTACATTTATTCTTGCTATAACAATTGGATTAGGATTGAC

**FLABE**_*Diplocirrus-Flabelligera*

?????????????????????????????????????????????????????????????????????????????????????????????????????????????????????????????????????????????????????????????????????????????????????????????????????????????????????????????????????????????????????????????????????????????????????????????????????????????????????????????????????????????????????????????????????????????????????????????????????????????????????????????????????????????????????????????????????????????????????????????????????????????????????????????????????????????????????????????????????????????????????????????????????????????????????????????????????????????????????????????????????????????????????????AATGCAGCCCGTCGCCACGGGAGCTACTCCCTCTGCCCGATACTGCAAGTACTACGGAGTCTTTTTTCTTCCGTTACATTTCGCGACTTGCCGTACAAATGTTTCAGAATCATCTGTTCGTGTACCCGTAGGAGATATGAGGCGGCAGCATGAACCCTACAGCGAAGACCCTCAAGACTGCAAGGGCCTCTCACGGGAACAACCAAGGCTTGCAGGTGCCTCGGGTACCCCGGAGGCTTCCCCAAATGAAGTGTTCAAGGGATTTCAGTTCGCTGGAGGCACGTGCCATCCTGCCAATCCCCCCAGACAAGGACGCTCTTATGTCCATGGATTAAGTTTCTGGTTATCAGGCTTATGGGCACCTGTCTGCAATCAACCAGGGCGTGCCGAATCTCGCCAGAGCTTTGTTCTTCCCACAACGGTGGGACAGACTATACGTGTGGTGCAGCACAGCCTCCAGAGCTGCTTCAACCTTAATGTGCCAGATCCTTGAAGGCATGCTCCGCAAACGTCTTGTCTCCAACACCGAGCGAAAGAGGATTTCCCGTAGTGCGACTCAAGGAGAACCAAGCACACGAGTTTGAGCCATGCATGCACCCATCCGCTAGCGTCGGCTTCTCAGAAACCCA???????????????????????????????????????????????????????????????????????????????????????????????????????????????????????????????????????????????????????????????????????????????????AATGCTAATAGTCATGGTCATTGATCGTACATCCTACTTTTGTAACAACAAGCTCCGACCCC??GGGAAGAGCCTCAGATCAGACAATACGGG??CTTGCTGG?CACGGGCTCAGC?CAGCCGGAGCTTTTCAATTCTCTTCGTCGATGGCAGGTGACCTCCTGCCATGATGAAGGCCAACACTACGCCCCGACAGGAAAACGTGCGGGACTCTTTGGCCCGTACT?GCTCTTCCTTGGAGTTTAGTCGTCCTGTCTCGGG?????????????????????????????????????????????????????????GCTGACTAGTCCCGGGCGGCCGGACCGTGACAGCCAAAAGAGGCAATGAACGAGTTGTAGGTGCCTCGGTTCTGTGTGT?TTCGGAATGCGAGGTAAGGGAAGACGGTCGTTGCGTCGGTATCGCCCAGAACACTAATCGAATTTTCAGAACACGTGCGCGACGACCGCGATGCCCGCGTCGA???TTGACCCGGCGTCAGCCCCCACTAGGTCTTGGACATAGTGATTAGAACTCCCCCGCCTGTTGGTGGGGCTCTAGCGCATTCGCCTT???????GAACTAATGCTTTCTATGGTTCCGCCAAAGAATGCGTT????GCGGTCCAAGGTTGCCCGTTACCTCTTTATTCTGGCATTTTGCCACGGACAGGGACGATCCTT???????????????????????????????????????????????????????????????????????????????????????????AAAATTTCCTTTAGAGGCTAATGTGACAACGAAGATATTACGTCATAAATAACTAAATTATATTTAGGAAAGCCTAAATCAATTAAAAAATGCAGGCTTGATATTTATTTTTCAG???????????????AAATATTTGTGCGACAAAGGAAATTAAATCATCCTTAAATATACGTACTTCCCAGTCTGATCAACTAAAGCCTCAAGCTTTCTTTCTCAAGTTATCAACAGAAAGTC??????????????????????????CTTCACAAACTAAGCCCATCGGGTGACGACCGCCCCCGCGCGCGTGGCGGGAGCGTGTAGGGAAGGCCGGTGCGGGCCTCAAGGGGTGCCCGGCTCTCTTGTCGGGGCGTCATTCTCGTCACGTGCCTCTGGTCGTTCGCTAGCTTCCCAGCGTAGAGCAGTGTAACATCTGCTGACGCACGGATTAACAGCGCTTAGAGGGGGATGGGACGCAAAGTCGACCCGCGGGTACT?GTTTCGTTCCGGGGCCCTCGGGTC???TCACGCG?CGACGGGTGCACTCCCGCGGGGAGCCCGCGACGGTTCCGGCCGTCAAAGCCTAGGGGAAGAGTCAGCTCGCCTGATGTTATAGCCCTTACGGTATTGGCCGGCTGGTGGACCAGACAATTGAAGGCCCTTGGTGGA????AGAGTGTCTCCTCCGCCCGCCGACTGGACTCGCACAGTAAGCTGGTGTCGCACGGTGCCCAAGGTCCGCGGCGATACTCGGCACCCCATCAAGCTAAATTGCGCATTGGTGCAACGAAACCTACAGGATGAGTGAAGCGTGCCACTTGTGCGTTAGCGTTCCGGCCTGCGGGCACGCGCCTCTCGCCGTCGGTCGACGAGGAGGACAACGTACATTTGGGTACCTACACACAGGCTGGTCAAGGCTGCTTGGTTAACTCCTGCACTCGATTGGTATCGTAGATGGCGAACCCGTTACGCCCGTGTCAATTATCGGGCCGCAGAGTGCAGCCCTACTCCTGCCACGCAGATCTCACAACAATTTGTGAATACAGCGCCGCTCCGTGTCACTAGGGTCGGGCAGGACGTCGGGCATA?ACGCACCTCCGCGATGGGAGGTCTGCGGAATTGGCTGCGGTTAACCGGGGCGCGCAGTCATACGGATATGTTAAACGTAGATAGATGCTCGTGCCAATGACGGGCGGGGCCTGCCCCCGTGACTATATCGTATCGACCGGACGCGGAGATGTCCGTCCGAGGACCAGTGTACAACGAACCAGTGCCCTGCGAGACCCCGGACTTGAAGCGCACTCTGTCGGATTGCCGATGAGGTCGGTCCTTACCGCTCCCCGGGCGTGCGCTCTCGACGGCTAAACTGGGGGCAGTGTGATTTCGTGCCGGTCTGGACCATCGATAACAGACTCTCATCGCGAGGTCGTCGTCTGGCCCACGAGCGTGAGTTAGCCGTCACGGGTTGAAGTGGGC??????????????CCACCGTCTGGACCTCTATGTGGAGTGCCGCGGGGGTCTTGTCGCCGCGCTGACGACGGCCGAGCTCAAGGCCAATTCAACAAATGCTGCGGCAACGGTGCGMTTAAAATCCGGATCTAATACCGAATCAGCTATCTGTTGATTC?TAGAGGACGTGAGCACGGGCCAATTCGCGCGCTTCTTATATAGAGGCGGGTGCGCGGCACGTCTCCGTCTCCCGGGGGCGGGTCTCGCGCGTGCGCCTCTCTATATTCATAGCGGATAGCGCTCCAAGCAGGCGCTACACTTGGCTTTTTCGAAGCGATAACTATTCATGAAGGATTACAGCCTCTGATCGTCGTTAATCCCTCCCGTACATTTATTCTTGCTATAACAATTCACTTAGGATCGGC

**FRENU**_*Galathealinum-Siboglinum*

AGAATTAGCAGAAAATCAACTAGAAAAATTATCTCTCACAGGACAAATCTTTTCAGGTGATGATCAATTTTGTCTATACTTGACTATAATTGAAATATTTTATATTTTGATACTCTATAACAGGAGCTCATAGTTTCAAGTTTTAGATACACTCTCTCTTAGTAAATAGAATATCTGCCTTCATGAAAAATATAAAGATTTTAGATCTAATATTGCTTGCAACCATATATTTACTTCTCTCAATACTTTTCTTTCAGATCTATTCATACATGTAATATAGGACTTCGTTTAGAACGTGTCCTTATGTAAAGGGTCAAATAAGCAATCTCTCTCTTCTGTATTATGAGTCTTATATACATGAATTAATATCACTTTCTAGAATTTTGTCATTCATCATATAACTTTATTATAAATGTTATATTTGATTAATTAGTTATATAATCAATGGTTACCATATCAAAAATTATATTCTACTCCAATAAAAGTTAAATATTTTATCGTAATTAAATAGTCTGGTTTGTTATATATTATAAATTTTCAGTATTTTTTTAAGATATAGATTCTATTGAGTTAATTCATGATCTTATTACAAACTTCTATTCTCCTCGTTGAAGAAAAATTTCTTTATTATATATTAAACCACAATTTATTAAGATAAAGTTTTA?????????????GTCGCGGAAGCTACTTCCCCTGCTCCGTGGTGCAGGTACTACACCATCTCGGATCTTCCGCCTCGTCCCGCGACTGGCAGTGTGCGCGTTCCAGAACCGGCCGCACGCCGTCGCCCCAGAGATGCGCGCCGGCAACACGAGGCCCACAGCGAGGTGCTACAACGCAAGAGGGAGCACGCACGGACCCAGCGGACACTGCGTCATGGCCCGGCACACCCCGAGGGAAGCAGCAATGACATGTTCAAGGGATGCATGACCGCTCGAGTCGCCCTACATCGACCCGGAGCCCCCCAACAAACGCGCTCTGCTGCATGTGGATTAGGCGACTCGTCATCCGACCCATGGCCACACTTTACCAACCCACCCGGACGTGTCACCGCGCGGATGAACTTTGCTCCCACCACAACGGCCGCACCGTCTATTTCTGCGGCGCTCGGAACTCGCACGCGAATGTGGCGACTCACCATGGCATGGCCTTCAGACCAGAACATGCGACCCTCCTGTCTGCAGCCAGGAGACAAGGTGGATTTCTCGTACCGCGGAGCTCGCAGAACGTCCTCGACATCCATGCGCCATGCGTGAGCTCGCAGGTG?????????????????????????????CCGACCAACACAGGCGGGATGCCGAGTGGGCCTCGGCTCCGATCGTTCTGCTCTTGATCCGGCGCCCCGCGGGCTCGGCCACAGCGGCCGCCTGAGCTCATCGGACAGCGGGCCGGTCCCACCCCCGCCGCT??????????CTTATGCTGCTAATACACACTTTCACACAGCAACGTTAATGGTTACTCTCATTGATCGTTCCACCTACTTTTATAACAACCAGCTCCGACCGGGCGGGAAGGGCCTTAGATCAAACAATCGGGCTCCTTGCTGGCTTTGGGTTCAGCACCGCCGGAGTATTACAATTCCCCTCTTCGATGGTACGTGCTATCCTACCATGCTAAGGGCCAAAACTACGCCCCGGCAGGAAAACATACGGGTCTCTATCGTCCGTATTAACTTTTCCTTTAAGAATAGTTAGACTGTCTGGGGTGCAGGCCGCGTCCGCCTCGCGGG?ACTGCGTCCTGACCCCCTGCCGGTTCCCCCGAGCTCATCAGTTCTGGGTGGCCGGAACGTAATTATCAAAAGTCCAGCCTGTATAATGCCAGGTGCCTCGGTTCTAGTTGTTTTACGGAACCGAGATGAGGGAAGACGGTCGCTACGGCTTTGTCGCCTAGGATACTAATCGAATTTTAAAAACATGTGCACGATGATGACGATCCCCGGTTTGTACAATGACCCGSCGGCAGCGTCCGACAAATGGCGACCATATTGATTAGAACTCCTCCGCTTATTGGCGGAGCTCTAGCATATACGCGCTTGCGCGGTGACAAGTGCGTATCACAGTTTCGCAAAGGAATGCGTCGCAATTGCTCCCGGGCTGTCCGCTACCTCCTTCTACTGGGCTTCTCTCACGGACAGGGACCATCCTTTTAATGGATTATGATCCTCGCTGGCCCCGCGGGGGACCCCTCCCCGGTGGGGGGGATGCTCGTTTATCTGCTA???CAAATATGCGGGCCGAACATTTCTTTTAAAGGCTAATGTAATAACGAAAATTTAGCGCCTTAAATTTATAAATAATCTTAAAGAAAGCTTTAATCTATTATAGAATATTGGCTTTATATAATTTTTACAAAGTATTAAACTTTTATAATATTAGTGCGACTAAAGGATTAAAATCTTCTTTTTATTAAAGGCCATTTCGGTTTAACTAAAAATAGCCATAATCTATTTTTCTTTAATTAATAACAGAAAAGAAGCGCAAGATTTCCTAGTAACTAATCCTCCACAAACTGAGCGCATGTGGAGACGACCGCCCCTGCTAGGGCAGCGGGAACGTGATAGGACAGCCTCTGCGACGTCGACCGGTCGTCTAGTCCCCCTGTCGGGGTTACATACTTGTGACGCCGCCGGTC?GCGTCGCTTGTATCCTGGCGTTGAGCAGCGTAACATCTGCCGACACTCGAATTAACACCGTTTAGAGGTGGATGGGACGCAAAGTCGAACCGCGAATGCTGGGCGCCGCG?CGTGTTCGGTGACCTTCCGAGGGGGCGGCGGGTGCACTTTCGCGGCGAGCCCACGACGGTTCCGGCGGCCGATACTCCGTCGAAGAGCCGCGCTGCGTGGTGGTACAGCTTCGGGCGTTCTGGCCCGCGCGCGGACCAGAGCTGTGCCCGCGGGCGCACGCCCTCCT?TCGGCCGTTCCACAGGTGCACTGTTCATGCTTCGACCGCGTCCGGGTTCGGACGCCAAGGGTCAGTGGCGTCGGCCGGTCGCCTATCAAGCTAGATTGCGCACTGGACGCACTAAACCTACGGCATGAGTGAAGGCGCGTCCTCGGCGCCCAGTAGTCGCGCGCCCGCGCCTACGCCGCTCGTCCGCCCGCGGTGAGGCGGACGACGTACACCTGGGTACTTATTTATAAGCTAGACTAGACAACTTTGTTAACTCCTGCGCTCGCAGAGTTTCGCACCTGTCGAACACATTAAGTCCGTGTCGATTATCGGACGTTCGAGTGCAGCCCTACTTCTACCACGAGGATCTCATAATAATTTGTGAATACAACGCCGATCCGTGTCACTAGGGTCGGGTAGGACGTCGGGCACTGCCGCAACCCCGCGACGGGAGGCCCGCAGAACAGGCCTTGGCCAGCCGGGGCGCGCGGTCATACGGATATGTTGAACGCAGATAGCGACGCGTTCCGAAACGGGGGAATGA???GCCTGTA?TCTATATCGTATCGACCGGACACGGAGATGAC?TCGCCGAGTCTAGTGTACAACGAACCAGAGCTTGGCGGAATCCCGGACTTGAGACGGACTCTGTCGGTTTGACGCTGACGTTGCCTCCTACCGCCCCCCGGGCGTGCACTTCCGTCGGCTAATCTGGGGACGGTGTAATTTCGTGCCGGGCTGGACCATTACTGACAGATCATCATCGAGAAGCTGTCGTCTGGAGTACGAGCGGCCTGGAACCGGCAGGGACTGGCGAGGCGGTGTGGCCGATTCGCCGTCGTCGCGACCTTCCCGTGGAGTGCTCACTGGCGTCGGTCGCGTCGTTCGCTTCGGCCGGATCCAAAGTTGATTTAACAAATGCTGCGCAGCTGGTGTGATTAAAATCCGGATCTAACGCAGAATTAGCTATCTGCCGACTTTTAGAGAATGAAAACGCGGGCAGTTTTTTACGTTTCTTATTTAGAAGCGGAGAGCGGCAGCGCCCACGCCTGGAACGAGCGCGTGC??CGCGAGTGTCCCTCTAAATTGTCAGCGGATAGAGCTTGAAGCAGACGCTACACATCACTTTTTCGAAGTGTTAACTATTTATTAAGGATTATAACCTTTAATCGTCGTTAATCCCTCCCGTACATTTATTCTTGCTATAACAATTGGATTGGGATCGAC

**GLYCE**_*Glycera*

????????????????????????????????????????????????????????????????????????????????????????????????????????????????????????????????????????????????????????????????????????????????????????????????????????????????????????????????????????????????????????????????????????????????????????????????????????????????????????????????????????????????????????????????????????????????????????????????????????????TATTACCCCACCGCAAATGTTTACCCCGCCTAATTCGGAAAATGGCCTACAGAAACCACGACAAAAATTATACACAGCTCCTATATCAGCATAATATTTCACAGTAAATAAAATATCATCTTTCCTTGACATCATTCTCCCTCAGTATTCTTCTAAGAAGATATTGCCACCGGGGTCTGTCACGATCTTATTACGACTAACCTTAACCAACGTTGAGCAAAAGCCCTTTCGCCGTATATTATTCCCCAACCCA?????????????????????????????TTCGCGGACGCTACTTCTTTCGCTCCGCGCATCCAGTATTACGCCATCCCCAATCTTCCGCTATTCTTTACCATTTGCTGTGTTAGTGTTCTAAAATTGTCTGTTCTCCCACCCTTAGGCCATGAGAACCAGCTCCACGAGACCAACAGCGAATCTCTCCAATACAAGAGAGTCTGCCCACGGACTCAACTGATACTACATCGTGACTCGGCATACCCGGAGGTCTCTCCTAATGGCCTGTGGAAGAATTGGCCCACCCCTCGCTGCTTGCAACATTGACCCAAAAGCCTCTGAGAAGCACCCTCTCATGTCCATGGATTAACATTCTTGTTGTACGGCAATGTGCTACCTGTAGTCAATGTACCTGGGAGCGTCACCGCCCGGAACAGCACAGTTCTTCCTGCAACGTTTGCACCGTCACAATTGCCTCAGCAGTGAATTCCGCAAGGAGACCGCTGAATCAAAGTGTTACCGCCTAGAGACCGTGCTCCTCCTCACTCCAGTCTCCAGCAGGGAACCCTTGAGGATTCCTCGTTCTGCGAAGCTTGGATAACCGAGAAGGCGTCTATGTGCCATGTGAGAGTCCCAGCGCT?????????????????????????????????CACGCGAGGGCTCCGTCCCGAGCAAGCCCAGGCTCTGTTAGTCCTGCTCCCGGAGCTACGCCCCGCGCGCTCTGCTGTCGCGCTCCCCCGAGCTCCCCGAGTAGCGGTCCGCACCCGCCCC?????????????ACCTCTTATGCTGCTAATACATACTTTTACATAGTAACGTTAATAGTTATGGTCCTTAATCGTACATCCTACTTTTGCAACAACAAGCTCCGACCTTCGGGGAAGAGCCTTAGATCAGACAATACGGGCCTTTGCTGGCTTTGGGTTCAGCTCAGCCGGAGTATTTCAATTCTCCTCTTCGATGGTACGTGATATCCTACCATGTTAAAGGTCAAAACTACGCCCTGGCAGGAAAACATACGGGACTCATTCGCCCGTATTAACTTTTCCTTTAAGTTTAGTTAGACTGTCTCGGGTCCCGGCTGCGTTCGCCACACGGGCACTGCGTCCGGACCTCCTGCCGGTTCCCCTGGTCTCGTTAGTCCTGGGTGGCCGGAACGTAATTATCAAAAGGATTGCCTGAATAATGGTAGGTGCCTCGGTTCTATTTGTTTTTCGGAAGCGAGGTAAGGGAAGACGGTCGTTACGGTTTTGTCGCCTAGAATACTAATCGAATTTTAAGAACATCTGCACGACGACTACGATCCCCGGA?TGTTTCCTGACCCGGCGGCAGCTTCCGACAAGTCTTGGCCATAGCGATTAGAATCCCCCCGCCTATTGGTGGGGCTCTAGCATATTCGCCGTGTCGGTGCAACAAGTGCGTTTCACGGTTTCGCCAAGGAATGCGTT?TTGATTGTCCGGGGCCGTCCGTTACCTCCTTCTATTGGGCTTATCCCATGGACAGGGATAGTCCTTTTAATGGTTTGTGATCCTCATTGGTCCCGCGGGGGCAACTGCCGCGTGGGCGAAGACGATCACTTGATCATTA???????CCCGCAGGCTAAACATTTCCTTTGAAGGCTTTTGTGATCACGCAAATAAAACGCCCAATAACTGTAACCAGACTTTGAGAATACTCAAAAATAATAAAGAATATAGGCTGCATTCTTGATAAAAGCCCCT??GACTACATACGGAATTTGTGCAACAAAGGAACCCAAAACTTCCTTCTCATACAGTATTAGTCTGTCAGATCTAAAAAAGCCTTAAGCTATCCTTTTAGAGTTACTGACAAAAGGGAGGCGCAAGATATCCTTGTAACAAATCCTCCACAAACTAAGCCTATGCGGAGACGACCGCCCCTGCCGAGGCAGCGGGACCGTGTTAGGACAGCCTCTGGGGCAGCGTTCTGGCGTCCGGTCCTCCTGTCGGGGCTTCATACTCGTCACGGCGCCGGTCGCCGTCCCTTGAGTCCTTGCGTGGAGCTGTGTAACATCTGCTGACACTCGGATTAACAGCGTTTAGAGGCGGATGGGACGCTAAGTCGGCTCGTGGATGCTGGCGCA???G?GTGGACCACGGATCTTCCGCTTGGGCGGTGGGCGCACTTCCACGAGGATACCACGACGGTTTCGGCGGTCAAAGTCCGGGGAAAGAGCTCTCGCGCGGAGTGTTATAGGCCCCGAGACGTTGGCCCGCTGGGAGACCAGACACGCGCCCGCCCGGCTCGGCCCGCCTTGTGTCCGTTCGACTGGAGGACTGTACATGCTCCGACTGCGCGAGGTCCGGGTCGCTCAGGGTCAGTGGCCTCGGTCGGCACCCCATCAAGCTAAATTGTGCACGGGTTCTACGAAACCTAGAGCATGAGTGAAGGCCTACC?TCCTGGCCCAGTAGTTCCTCCTCGGGACCTACGCCGCTCGTCCTCCGTAGGTGAGGCGGACAACGTACACTTGGGTACCTGTACACAAGCTGGTCTAGACAACTTGGTTGACTCCTGCACTCTTCGAGTTTCGTATATGACGAACACATTAAGTCCGTGTCAATTACCGGGCGATCGAGTGCCGCCCTACTTCTATTACGATGATCTCATAATAATTTGTGAATACAACGCCGATCCGTGTCACTAGGGTCGGATAGGACGTCGGGCACCGGGGCGGCTCTGCGACGGGAGGCCTGTGGGACAGGCCTTGGCCAGCTGGGGCGCACGGTCATACGGATGCGTTGAACATAGATAGAAACTCGTTCCGAAACGGGGCAATGC?CGGCCCGTACTCTATATCGTATCGACCGGATGCGGAGAGGGCCCCTCGGGGTCTAGTGTGCAACGAACCAGAGCTCGGCGGGACCCCGGACTTGAGGCGGACTCTGTCGGCTTGCCGATGACGTTGTCGCCTACCGCTCCCCGGGCGTGCACTCCCGTCGGCTAATCTGGGGGTGGTGTTATTTCGTGCCGGACTGGACCGC?GATAACAGATTATCATCGAGAGGCTGCCGTCTGGGGTACGAGCGGCGTGGAACTGGCAGGGGCTGGCGAGGCCTTAAGGTCGACTCGCCGTCGCCGGGACCTTCCCGTGGAATGCTCACTGGCGGCGGTCGAGTCGTTCGCTTCGGCCGGATCTAAAGCCGATTTAACAAATGCTGCGTAGCTGGTGTGATTAAAATCCGGATCTAACGCAGAATTAGCTATCTGCCGACTTTTAGAGAATGAGGGCGCGGGCAATTTTTTACGTTTCTTATTTATAAGCGGAGAGCGGGCAAGCCCTGGACTGGAACGACCGACTCGGCCGGGCGCGTCCCTCTAAAGTGTCAGCGGATAGAGCTTGAAGTAGGCACTACACATCACTTTTTCGAAGTGTTAACTATTTATTAAGGATTATAACCTTTGATCGTCGTTAATCCCTCCCGTACATTTATTCTTGCTATAACAATTGGATTACGATTGAC

**GONIA**_*Goniada*

??????????????????????????????????????????????????????????????????????????????????????????????????????????????????????????????????????????????????????????????????????????????????????????????????????????????????????????????????????????????????????????????????????????????????????????????????????????????????????????????????????????????????????????????????????????????????????????????????????????????????????????????????????????????????????????????????????????????????????????????????????????????????????????????????????????????????????????????????????????????????????????????????????????????????????????????????????????????????????????????????????????????????????????????????????ATCGCGGGCGCTACTTCCTTTGCCCCATGCCGGCAGTATTACGTCATCTTCCCTCTTCCGCTATTTTTTACCGTTTGCTGTGTTCGTGTTCCAGAATCGTCTGCTCCCTCACCCTTGGGCCACGTGAACCGGCTCCAAGAAACCGACAGTGAATCTCTTCAATGCAAGAGAGTCCGCCTACGGATACAATCGATGGTACTTTGTTACTCGGCATACCTAGAGGCTTCACCAAATGCCCTGTGGGTCAAGTGGGGCACCCCTAAGTGCTTATAACATTGACTCGCAAACCACTGAGAAGCCCCCTCTCACGTCCGTGGATTAATAATCTTGTTGTCCAGCATTGCGTTGTCTGTTGTCAATAAACCTACGTGTGCCACTGCCCTGAACAGCTCTGTTCTTCCTGAAACGTTCACACCGTCTAAATTGCTTCAGCAGCGAATTCTGCAAGGAGACTGCTGAATCAAAGTGTCACCGCCTTGAGACCGTGCTCCTCCATATTCCAGACTCCAGCAGGGAACGCTCGAGGATCCCTCGTTCTGCGAAGATTGGATAACCGAGAAGGCGTCCATGTGCCATGCGAGAGTCCCAGCGCC???????????????????????????????????????????????????????????????????????????????????????????????????????????????????????????????????????????????????????????????????TGTTTCCTCTTATGCTGCTGATACACGCCTAAGCACGGCAACGTTAATAGTTATGGTCACTAATCGTAAACCCTACTTTTGCAATCG?CCGCCCGGAACCTCGGTGACGGGCCAGAGAACAGACAACACGGGCCTTTGCTGGCCTT??GCTCAGCTCCGCCGGAGTATTTCGATTCTCCTCGAAGATGGTAGGTGACTTCCTACCATGTTTAGGGCCAAAACTACGCCCCGGCAGGAAAAGATACGGGACTCATTGGCCCGTATTAACTTTTCCTTTAAGTACAATTAGACTGTCTCGGGTGCCGGCGGCGTTGGCCGCAAGGCTACTGCGCTCCGGCCCCCTCCTGGTTGCCCTGGTCTAACCAGTCCTGGGTGACCGGAACGTTATTATCAAGAGTGTCGCCTGGATGGTGGTAGGTGCCTCGGTTCCGTCTGTACTTAGGAAGGGAGGTAAGGGAGGCCGGTCGTTACGGTTTGTTCATCTAGACGAA??GTCGAACT???AA?ACA?GTGCACGACGACTACGATCTTCGGATTGCATCGCGACCCGGCAGCTGCCCCCGACAAGTCTTGGCCGTAGTGATTGGGACTCCACCGCTTAACGGTAGGGCTCTAGCACAAAGCCAGTCTGGCTGCCACTTGTGCTTTTCAAGGTTCCGCCAAGGAATGCGTT?TTGCCTGTCCTGGGCAGTTGGTTACCTCCTTGTATTGGGCTTGTGCCATGTGCAGGGAGCGTCCTTTGAATGGTTTGTGACTGTCGTTGGCCCCGC?GGTTCGCCCATCGCGTGGGCAAGGACATTCACTGGATCATTAAAA???????????????AAAATTTCCTCTAGAGGCTATTGTGACTTTGAAAATATATCGCCTTATATTTATAAATGATCTACAGGAAATCCTGTAATTTTTAAAGAATATTGGCTTTATTTTTATAAAATGAATAA?TCACTTAATAATAAATCTGTGCGACACAGGCATCTTTAACTTCCTTTATCAAAAGCACAAATCAGTCAGATCAAAAAAAGCCATAAGCTATCATTTCAGAGTCAATGACGAAATGGG??????????????????????????CTTGACAAGCTAAGCCTATGCGGAGACGACTGCCCCTGCCCTGGCAGCGGGACCGTGTTGGGACGACCCCTGGGTAGGCGCTGCTCTGCTCGGTCCTCCTGTCGGGGCTGGATGCTCGTCAGGGCGGGCGGTGCCTTCCC?CGGGTCCTCGCGTGGAGCAGCGTAACATCTGCTGACACACGGATTAACAGCGCTTAGAGGCGGATGGGACGCAAGGTTGGCCGGCGGGTACCCCCGGGAGCCGAATCTCTCGGGATC?????GCCCGGCGG??GGGCCACTTCTGCGCG?TGTCCACGACGGTTTCGGCGGCTCAGGCTCCGGGGAAGAGGGCGGGCGTGGTCTGTTACAGACCCGGGATGCAGGGCCCGCTGCGAGACCAGACTGGCGCCCGCCCGGCACGG?CTGCCGAGGGGGTGTTCGACTGGATGACTGTGCATGCTCCGACCGCTTGACGGATG???CGCCAAGGGTCTGTGGCCTTGGTCGGCACCCCATCAAGCTAAATTGCACACGGGTCCTACGAAACCCAGAGCGAGATCGAAGGCCGGTCTAGG?GGTCGAGCGGTCCCTCCTCGGGGCCCGCGCCGCTCG???TC??TCGGCGAGGCGGACAACGTACACTTGGGTACCTACACACAAGCTGGTCTAGACGGATTGGTTAACTCCTGCGCTCTATGAGTATCCTACCTGACGAAGTCATGAAGCCCGTGCGGCCCATCGGACGATCGAGTGCAGCCCTACTGCTACTACGGTGATCGCATAGCTATTTGTGAATACAACGCCGATCTGTGTCACCAGGCTCCGGTAGCACGTCGAACAACGGGCCGGTGTTGCGACGGGAGGCCCGCGGACTGGGCTTTGGCTAGCCGGGGCGCGCGGTCATACGGATGCCCCGAGCGTAGATGGAAACTCGTTCTGAAACGGGGCAATGTC??GCCCGTC??CCATATCGTATCGACCGGACACGGAGATGTCCCTTCGGGGGCGAGCGCACAAGGAGCCAGAGCCCGGCGGGGCCCCGGAGTTGAGGCGGACTCTGTCGGCTCGGCGATGACGTTGTCGCCTACCGCTCCCCGGGCGTGCACTCCCGTCGGCTAATCTGGTGGTGGTGTTATTTCGTGCTGGACTGGAGCGC?GATAACAGATTGTCATCGAGAGGCTGCCGTCTGGGGCAGGAGCGGCGCGGAACCGGCACGGGCTGGCGAGGTCGTCGGATCGACTCGCCG?CGTGGGCACCTTCCCGCCGAGTGCTCACTGGCGGCAGTCGGGCGGTCGGCTTCGGCCGGACGTAAAGTCGATTTAAATGACGCTGCGCTGGCGGTGTGGTTAAAAGCCGGATCTAACGCAGAATCGGCTATCTGTCGACTCTTAGAAGATGAGGGCACAGGCAATTCTTCACGTTTCTTATTTGGAGGCGGAGATCGGGCAAGCACCGGCCTGGAACGCGCGGCCCGGTCGCGCGCGTCCCTCCAACGTGTCGGCGGATAGAGCTCGAAGCAGGCGCTACACATCGCCTTTTCGGGGCGTTAACTACTTGTCAAGGACTATAACCTTTGATCGTCGTCAATCGCTCCCGTGCATTGGTTCTTGCCACAGCACGTGGATCACGACTGAC

**HESIO**_*Hesione-Ophiodromus*

CGTATCATCCGCCATAGCACATCAAAACTTCGCAGCAACCGGACATGGCTTTACCGGAAGAGACCACCTTACTATGCTCTCCATCTAATCCCTAGAGTAATAAACATTGTCATAGCATAGCTGGGAGACGTACCTATTAAATCAAAACACTTGCCCCTTTAACACTCACCGCGTCTGCAATCGAGAAAAGGATTACGGCGTCTTAAGGAACATBKCGCGCCGCCCTCTGACCACTTTTCCTACGCGGTATCTATCTCGTCCGATCACTCTAGTCATTCAGCCTACCGCCTAGAAATAGTCCGCTTGTGATCAATTAAAATAGCCATTATACATATCCTAGACCTGGCTACCATATACGTGTATCAACTGCTCTTCCTGCAGCCCTGCCGACCATCAC????????????????????????????????????????????????????????????????????????????????????????????????????????????????????????????????????????????????????????????????????????????????????????????????????????????????????????????????????????????????????????????????????????????AGGGCAGTCCGTCATCACGGGTGCTGAGTCCCTCGCTCGGCGCCTCCAGTACTACGCCATCTTCTATCTTCCGCAACCTCTCGCGACTCGCTGTGTAAGTGTTCAAGAATTGCCTGTTCGCCCACACTTGGGCGACGTGAGCCGGTTCCACGAGACCCACAGTGAGGCTCTTCACTGCGAGAGGGAGCCAGCACGGATTCAACTGACTTTTCATTGTGACTAGGCATACCTGGAGAAGTCATCAAATGACCTGCACAAGGGATGGCACACCCCTTGAGTCCCTCAACATTGATGAACAAGAACCAGACCGTCCCGCTCTTCTGTACGAGGATTAACATTCTTGTCAAGATATCTATGGTCACATGCATGCATGGTACATGTGCGAGTCACCGCCCTGATGCCCTTTTTACTTCCCGAAACGCCCGTACCGTCTAAACGTGTGGAGCTCCGAATTCCCAGCTGGATGTGAGAACTCTTGGAGCTATCGCTCAGAGACCGTCAGACTCCTGTGTCCAGCTCCCAGCTCAGAGCGACCGAGGATCTCTCGTTCTGAGAAGCTGGGAGAACCGAGAAGGGGTCTATGTGCTAGGAGTGAATCCATCCGCCAGTGTAGGCTTCTCAGAAATCAGCCTA??CTGTTACGCCAAGGCGCCATCAAGTCTAAGCCTAGGCTCTGTACGTCCTGCTAACGATCTTGCCCCAAACCAGCTCCCCTGCCGCATCCGCCCGAGCTCCTCTGGCTCTGGTCCGCTCCTGCCCTCCCTGCT????????GAGTTATCATCCT??TACAAACTTTGACACAGTAACGTTAATAGTTATGGTCCTTAATCGTACCTCCTACTTTTGCAACAACAAGCTCCGCCCTTCGGGGAAGAGCCTTAGATCAAACAATCGCGTGTTTTGCTGGCTTTGTGTTCAGCTTAGCCGGAGTATTTCAATTCTCCTCTTCGATGGTACTTGATATACTACCATGTTAAAGGCCAAAACTACGCCCTGGCAGGAAAACATACGGGACTCTTT?GCCCGTATTAACTTTTCCTTTAAGTTTAGTTAGACTGTCTCGGATCCCGGCCGCGTCCACCTCACGGGTACTGCGTCCGGATCTCCTCCCGGTTCCCTTGGGCTGACTAGTTCTGGGTGGCCGGAACGTAATTATCAAAAGGCTTGCCTGAATAATGGTAGGTGCCTCGGTTCTATTTGTTTTTCGGAACTGAGGTAAGGGAAGACGGTCGTTACGGTTTTGTCGCCTAGAATACTAATCGAATTTTAAAAACATCTGCACGACAACTACGATCCCCGGATTGTTTCATGACCCGGCGGCAGCTTCCGACAAGTCTTGGCCATAGTGATTAGAATCCCCCCGCCTATTGGTGGGGCTCTAGCATATTCGCCGTGTCGGTGAAACAAGTGCGTTTCACAGTTTCGCCAAGGAATGCGTCGCTACTGGTCCGAGGTTGTCCGTTACCTCCTTCTATTGGGCTTGTCCCATGGACAGGGATAGTCCTTTTAATGGTTTGTGATCCTCGTTGGACCCGTGGGGGCAACCTTCGCGGTGGCGAGGACGATCACTTGATCATTA???CCGATTCGTGGGCCAAACCATCCCTTTGAAGGCTCTTGTGATAACGAGAGGTTAATGCCCACTACACATAAATAGTCTCCATGAGAACCTGGAATAACTAAAAAGTGTTGGTTTAACTTAACCTAAATATGCT?ATAATATAATGATAAGTTGATGCAATTGATGTAATCCCACCGTCATCCGAGAAAAG??TAAATCCTTTAAAAGAAAATCTACCACAAACCATCTCCTCAAAGACTACGCAGAGGGGGAGGCGCAGGGAATCCATATAATAACCCCTCCACAAACTAAGCCTATGCGGATACGACCGCCCCTGTCCAGGCAGTGGGAACGTGTTAGGACAGCCTATGGGGCGATGTACAGACGTCCGGTCCTCCTGTCGGGGCTTCATACTCGTCACGGCGACTGTCGTCTCCCCTTGAGTCCTTGCGTGGAGCAGTGTAACATCTGCCGACACTTGGATTAACAGCGTTTAGAGGCGGATGGGACGCAAAGTCTACTCGCGGATACTGGGTGC???TGGGGATA?CCGGATCCGTCGTGTCGGTCGCGAGCGCACTTCCGCGAGGAGCCTACGACGCCTCTGGCCGTCAAAGCCCAGTTCAAGAGCTTTCGGGAGGAGTGTTATAGGTACTGTGGCATTGGCCGGCTGGAGGACCCCC??????CCCGCCCGGCTCGGTCTGCGTGCGGGCCGTTCGACTGGAGGACTGTACATGCTCCGACTGCGGTTAGGT?GGGTCGCCGAGGGTCAGTAGCGTCGGTCGGCACCCCATCAAGCTAAATTGCGCATGGGTTCTACGAAACCTAAAGCATGAGTGAAGTTCTTCCTCTAGGAACTAGTGGTCCCTCCCAGGGGCCCACGCCGCTCGTTCTCCGTAGATGAGGCGGACAACGTACACTTGGGTACCTATTTACAAGCTGGACTAGACAACTTGGTTAACTCCTGCACTCTTCGAGTATCGTATATGACGAACACATTAAGTCTGTGTCGATTACCAGGCAATCGAGTGCCGCCCTACTTCTATTACAAGGATCTCAAAATAATTTGTGAATACAACGCCGATCCGTGTCACTAGGGTCGGATAGGACGTCAGACATCGGGGTGACTTCTTGACGGGAGGCCTGCGGAACAGGCCTTGGCCAGCTGGGGCGCGCAGTCATACGGATGCGTTGAACGTAGATAGAAACTCGTTCCGAAACGGGGCACTGACTCGCCCGTATCTTATATCGTAACGACTGGCTACGGAGATGATCCTTCGGGGTCCAGTGCACAACGAACCAGAGCTCGACAGGACCCCGGACTTGAGGCGCACTCTGTCGGCTTGCCGATGACATTGTCCCCTACCGCTCCCCGGGCGTGCACTCCTGCCGGCTAATCTGGGGATGGTGTGATTTCGTGCCAGACTGGACCGC?GATAACAGATTATCATCGAGAGGCTGCCGTCTGGGGTACGAGCGGCCTGGAACTGGCAGGGGCTGGCGAGACTGCAAGGTCGATTCGCCGTCGCCGGGGCCTTCCCGTGGACTGCTCACTGGCGGTGGTCGGGTCATTTGCTTCGGCCGGATCTAAAGCCGATTTAACAAATGCTGCGTAGCTGGTGTGATTAAAATCCGGATTTAACGCAGAATTAGCTATCTGCCGACTTTTAGAGAATGAGAGCACCGGCAATTTTTTACGTTTCTTATTTATAAGCGGAAAGCGGGCAAGCCCTCAACTGGAACGCCCGGCTCGGTCGGGCGCGTCCCTCTAAAGTGTCAGCGGATAGAGCTTGAAGCAGACGCTACACATCACTTTTTCGAAGTGTTAACTATTTATTAAGGATTATAACCCTTGATCGTCGTTAATCCCTCCCGTACATTTATTCTTGCTATAACAATTGGATTACGATTGAA

**HISTR**_*Histriobdella*

????????????????????????????????????????????????????????????????????????????????????????????????????????????????????????????????????????????????????????????????????????????????????????????????????????????????????????????????????????????????????????????????????????????????????????????????????????????????????????????????????????????????????????????????????????????????????????????????????????????????????????????????????????????????????????????????????????????????????????????????????????????????????????????????????????????????????????????????????????????????????????????????????????????????????????????????????????????????????????????????????????????????????????????????????????????????????????????????????????????????????????????????????????????????????????????????????????????????????????????????????????????????????????????????????????????????????????????????????????????????????????????????????????????????????????????????????????????????????????????????????????????????????????????????????????????????????????????????????????????????????????????????????????????????????????????????????????????????????????????????????????????????????????????????????????????????????????????????????????????????????????????????????????????????????????????????????????????????????????????????????????????????????????????????????????????????????????????????????????????????????????????????????????????????????????????????????????????????????????????????????TATGCCGCTAATACAATCTGTG??AAAGAAATGCTAATAGTCGACATTACTGATCTTG?ACGTTACATTTGCAACAACAAGCTTCGATCCCGTGATCCGAGCCTCAGATCGAGCAACGGGGTCCCTTGCTGGTGCTA?GATCAGCTCCGCCGGAAGATCACAATTCTCTTCGTCGACGGCACTTTAAGCACTGCCGTGTTGAAGGCCAACACTACGCCGAAACATAGAAACATACGGGACTCGTACGCCCGTATTGACTTTGCCTTCAAG?ACAATTAGACTGTCTCGGGAGTAGCCGGTGTCGGCCTCGCGGTTACTGCGCACCGAGCTCTTCTCGGTGGCCGACGGCCGACTGGTCGCGCCGGTCCGGCGAGTGATTGCCAGAAGGCAAGCCTGAACAATGATAGGCGACGAGTTCCCGTCCGTTTT?CTGAACCGTTCTAAAGGATGACGGACGTTGCGGGCGTGCCCTCCAGACGACAAATCGGATTTTAAAAACATCTGCCCGATGTCCACGGCTCCTGTCTTACTACATGACGCGGCGAAAGACCCCAACTGGATTTGACCATATTGATTAGAATCCCCTCGGCTATTGGTGGGGTTCTGCTGCCACGCCGCTACCT???AGTCAAGTGCATATCACAGTTCCGCCAAGGAAGACGTCGGCTGTGGCCAAGTGCCGATATTGAATTCCTTCTTCTGGCCTCGCGCCACGGATGGGGGTCGCCCCCTTGATGGTTTGTGCTCCTTGTCGGCCCCGC?GCCTCGCGCGGTGCGGAGTTGAGGACGAACGCTTGGCCATCA???????????????????????????????????????????????????????????????????????????????????????????????????????????????????????????????????????????????????????????????????????????????????????????????????????????????????????????????????????????????????????????????????????????????????????????????????????????????????????????????????????????????????????????????????????????????????????????????????????????????????????????????????????????????????????????????????????????????????????????????????????????????????????????????????????????????????????????????????????????????????????????????????????????????????????????????????????????????????????????????????????????????????????????????????????????????????????????????????????????????????????????????????????????????????????????????????????????????????????????????????????????????????????????????????????????????????????????????????????????????????????????????????????????????????????????????????????????????????????????????????????????????????????????????????????????????????????????????????????????????????????????????????????????????????????????????????????????????????????????????????????????????????????????????????????????????????????????????????????????????????????????????????????????????????????????????????????????????????????????????????????????????????????????????????????????????????????????????????????????????????????????????????????????????????????????????????????????????????????????????????????????????????????????????????????????????????????????????????????????????????????????????????????????????????????????????????????????????????????????????????????????????????????????????????????????????????????????????????????????????????????????????????????????????????????????????????????????????????????????????????????????????????????????????????????????????????????????????????????????????????????????????????????????????????????????????????????????

**HRABE**_*Hrabeiella*

???????????????????????????????????????????????????????????????????????????????????????????????????????????????????????????????????????????????????????????????????????????????????????????????????????????????????????????????????????????????????????????????????????????????????????????????????????????????????????????????????????????????????????????????????????????????????????????????????????????????GGCGTCGCGGAAAACGACTACTCAGAAAAATTTACATAATAACTAATAGATAATACTGCAAAAGTAATATGATACCCCTATATAAGCTTTTTATTTCATACTACATAACATATTATCTTTCATATATGTAATTCTTTATCAACACCTCTTTATCAAAAAATTGCTATTCTCATTTATCACGATTCTATTTCATTATGCTGTAATCCCCGTCTTGCCAAAGCTTTATAATTATATAATTAACCCCAATTCAATAAGACACAACCCCC??????????????????????????????????????????????????????????????????????????????????????????????????????????????????????????????????????????????????????????????????????????????????????????????????????????????????????????????????????????????????????????????????????????????????????????????????????????????????????????????????????????????????????????????????????????????????????????????????????????????????????????????????????????????????????????????????????????????????????????????????????????????????????????????????????????????????????????????????????????????????????????????????????????????????????????????????????????????????????????????????????????????????????????????????????????????????????????????????????????????????????????????????????????????????????????????????GTTTCCTCTTATGCTGCTAATACAAGCCTTAACACGGTAATGTTAATAGTCATTATTATTGATTGTAC?CCACACTTTTGCAACAACAAGCTCCGACCTAACGGGAAGAGCCTTAGATCAAACAATGGGGTTCTTTGCTGGCTTTGGGTTCAGTTTAACCGGAATATTTCAGTTCTACTCGTCGATGGTACGTGATATCCTACCATGTTAAAGGCCAAAACBRCGCCCCGAAAGGAAGACGTGCGCGACTCTATTGCGCGTATTAACTTTTCCTTTAAGTTTAGTTAGACTGTCTCGGATCCAGGCTGCGTTCA?CTCGCGGGTACTGCGTCCTGATCTCCATCCGGTTCCCTAGGGCTCACTAGTTCTGGGTGGCCGGAACGTAATTATCAAAAGAATTGCCTGAATAATGGTAGGTGCCTCGGTTCTATTTGTTTTTCGGAACTGAGGTAAGGGAAGACGGTAGCTACGGTTTTGTCGCCTATAAAACTAATCGAATTTTAAAAACATCTGCACGACAACTACGATCCGCCACTGGTTTAATGACTGGCCGGCAGCTTCCGACAAGTCTTGGCCATAGTGATTAGAATCCCCCCGCCTATTGGTGGGGCTCTAGCATATTCACCGTGTCGGTGTAACAGTTGCGTTT?ATAGTTTCGCCAAGGAATGCATTTATATTGGTCCGA?GTTGTCCGCTACCTCCTTCTATTGGGCTTATCCCATGGACAGGGATAGTCCTTTTGATGGGTTGTGG?GCTCGTTGGTTCTGCGGGGGCAACCACCGCGGGTACGAGGACGCCCACTTGCCTATCA???????????????????????????????????????????????????????????????????????????????????????????????????????????????????????????????????????????????????????????????????????????????????????????????????????????????????????????????????????????????????????????????????????????????????????????????????????????????????????????????????????????????????????????????????????????????????????????????????????????????????????????????????????????????????????????????????????????????AATTAACAGCGTTCAGAAGCGGATAGGGCGCAAAGTCCGCTCGTGG?TACCG????CTCGTCGGGTAAGTCCGATCCTC??GCCAGGCCGATGGTGCACTCCCGGGGTGAGCCCACGACGGTTGTGGACGTCAAGTCTCCGAGAACGAACCGTCGCGAGCGGCGTT?TAGTCTCG??GGTGATGGATCGCCGGCCGACCAGACC????GTTGCCGGACGAGGTCCGATCGGCGTTCG????ACCGGAGGACTGCGCATGCTCCAACCGCGTTTCGGGTCGGGC?TTCAGGGTCGGTGGCGACGGTCGGTAGCCTATCAAGCTAAATTGCGCATGGGTTCTACGAAGCCTAAAGAATGAGTGAAGGTCTTCCTCTCTGACTTAGTAGTCCCGCTCGCGGGCCTACGCCGCTCGAACTCCG?CAGTTAGGCGGACTACGTACACTTGGGTACCTATATACAAGCTGGTCTAGACAACTTGGTTAACTCCTGCACTCTTCGAGTATCGTACATGACGAACACATTAAGTCCTTGTCGATTACAGGGCACTCGAGTGCCGCCCTACTCCTACCACGATGATCTCATAATAATTTGTGAATACAACGCCGATCCGTGTCACTAGTTTCGGGTAGGACGACAGGCACCGGGGTGCCACCGTGTCGGGAGGTCCGTGGAACAGGCTCTGGCCAGCCAGCGCGCACGGTCATACGGATATGTTGAACGTAGATAGAAACTCGTTCTGAAACGGG?CAATGCCGTGCCTGTA?TGTATATCGTATCGACCGTGCACGGAGATACC?TTTCGGGGTCAAGTGTACAACGAACTAGAGCTCGGCGAGATCCCTGACTTGAGACTAACTCTGTCGGTTTGCCGATGACGTTGCTTCCTACTGCCCCTTAGGAGTGCACTCTCGTCGACTAATCTAGGGATGGTGTTATTTCGTGCCGGGCTGGACCAT?CCTAACAGATTATCATCGAGAGGCTGTCGTCTGGGGTACGAGCGGTCTGGAACTGGTATGGGCTGGTGAGGCCGCAAGGTCGACTCGCCACCGCCGGGACCTTCCCGTGGACTGCTCACTAGTGGCGTTCG?GGCGTTCACGTCGGCCGGATTTAAAGCCAATTTAACGTATGCTGCGTTGCTGGTGTGATTAAAATCCGGATCTAACGAAGAATCAGCTATCTGCCGACTTTTAGAGAATGAGAGCACGGAAAATTTTTTACGGTTCTTATTTATAAGCGGAGAGTGG??????????????????????????????????????????????????????????????????????????????????????????????????????????????????????????????????????????????????????????????????????????????????????????????

**LUMBR**_*Lumbrineris-Ninoe*

TGAATCATCCGATACAGCACCTCAAATATACTCAGCAATAGGACAAAGCACTGCAGGCAGAGACCGTATTACAATGTACCTCATCCTACCCTAAACGTACTAATAATCGACACTCATCTACTGGCGCACTTGGATTCCATACCTAAACACACTCACCTCTAATTCTCAGTATATCAGCTGCAGAGAGAGAATAAAAGTCCATAGCAGAAACATAGCCTGCAACCATCAGACCCCTTTACTCTAGTGACTTCTACCGGATCTACCTATACTTATTATTTAGGCCTACGACTAGAACGAGTACATATGTTATCAATTAAAGATACCACCTCTCTCATCCCAGCCATGGCAACAATATACACGAACCAACCTCACTTCCAGCTGAACAGATATCCATTACAYGGGGCTGCGGCTAACGATAAACCGGAACAATACGCTCAATAGATAATAGAAACCATTGCACCTATTATATGACCCACCAATACAAGCTCAATACTCCATAATACACATAACATTAGCCTTCATCCTTATTAGCTACCATTAGTACTCTTTCAAGAAGATGACGCCATTCTGATTACGCACGATCTCCTCACACCTAACAATACTATCCGCCTTCTAAAAGTCCTTTCCTCATATGTCAAACCCCAACTTACCAAGAAACAACCCCCAGGGTGGCCCGTCATCGCCGGAGCTACCTCCCTTGCTCCGTGCTGGCCATACTACACCATCTTCACTCTTCCACTCAATTTTGCGATTCGCTGTACCAATATTTCAGAATTGCCAATTCCCTCACCTGTTGGCTATGAGAACCGGTTACATGAGACCAACAGTGAGAGCCTTCCAAACGTGAGAAAGTGCCCACAGACTCAACAGACTGTACATTGTCCCTGGGCATACCCTGAGCTATCTGACAATGAAGTGTTCAAGGGATGGTCAATCGTTGCAGGCCCTCAACATTGAGGAATAAGCCACCGACAGGCACGCTCTGATGTGCGTGGATTAGGAGACTAATTGTTACGCATATGGAAACCTGCAGTTAACACACCTATGTGGGTCGAAGCCTGACTGAAGTGTACACTAACCACAACGGCGGCATCGTCTCAATGGCCAGCGCTCCAAATCCTGCAAAGAGACTGAGGAATCAAGGTGCCACCGCCTACAGACAATGCTGTGCATTACTCCAGCTTCTAGCAGGGAACCATCGAGGATCTCTCGTAGTGCGAAGCTTGGATAACTGCCCACGAATCTCTGCGCTATGGGAGAGCCCACAGGGCAGCGTTATCCTCGCCGATATGCAATATCG?????????????GCGCCGATCTGAGTCAGTTAAGGCTCTGTTAGTCCTGCTCTCGGAGCAGCCCGAGACGAGCTCGACTATCGTGCCCGCTCGAGCTACTCTGGCAGTGGTCTGTTCGTGCCCCTCCTGCT????????????TATGCTGCTAATACATACTTTTACACAGTAATGTTGATAGTTATGGTCCTTAATCGTACATCCTACTTTTATAACAAGAAGCTCCGACCTTCGGGGAAGAGCCTTAGATCAAACAATCGGGCTCTTTGCTGGCTTTGGGTTCAGCTTAGCCGGAGTATTTCAATTCTCCTCTTCGATGGTACGTGATATCCTACCATGTTAAAGGCCAAAACTACGCCCTGACAGGAAAACATACGGGACTCTTTCGCCCGTATTAACTTTTCCTTTAAGTCTAGTTAGACTGTCTCGGGTCCAGGCGGCGTCCTCCTCGCGGGTACTGCGTCCTGACCTCCTCCCGGTTCCCTTGGGCTGACTAGTTCTGGGTGGCCGGAACGTAATTATCAAAAGAATTGCCTGAATAATGGTAGGTGCCTCGGTTCTATTTGTTTTTCGGAACCGAGGTAAGGGAAGACGGTCGTTACGGTTTTGTCGCCTAGAATACTAATCGAATTTTAAAAACATCTGCACGACAACTACGATGCCCGGCTTGTTCAGTGACCCGGCGGCAGCTTCCGACAAGTTTTGGCCATAGTGATTAGAATCCCTCCGCCTATTGGTGGGGCTCTAGCATATTCACGGTGTCCGTGTAACAAGGGCGTTTCCCAGTTTCGCCAAGGAATGCATTGTTTCTGGCCCAAGGCTGTCCTCTACCTCCTTCTATTGGGCTCATCCCATGGACAGGGACAGTCCTTTTGATGGTTTGTGATCCTCGTTGGTCCCGCGGGGGCAACCGCCACGGTGGCGAGGACGATCACTTGATCATCA???????TACGCAGGCTAAAAATTTCCCTCAGGGGCTGTTGTGAAAACGAAATTATAACGCCTTTTAAATATAAACAAACTACAAGAGTGCCTGTATATACTAAAGAATGTTGGCTTTACCTACAATAAAATA???????????????TAAGGTTAGTGCGACTAAGGAAC?TAAAGCTTCCATTTAATAAAGTATATGTCTGTTACAACCAGAAAAGCCACAAACTATCCTCCTCAAGTTAATGACAGGAGGGCGGCGCAAGGTATCCTAGTAACTAATCCTCCACAAACTAAGCCTATGCGGAGACGACCGCCCCTGCCTGGGTAGTGGGAACGTGTTAGGACAGCCTTTGGGGTGG?GATCTGGTGCCCGGTCCTCCTGTCGGGGCTTCATACTTGTCACGGCATTGGTTTCCGCCTCTTGAGTCCTTGCGTGGAGCAGTGTAACATCTGCTGACACACGGATTAACAGCGTTTATAGGTGGATGGGACGCAAGGTCGCCCCGCGGATACTGGTGGG???TCGGAGTGTCCGGATCCGTCGATCTGGCGGTGCGTGCATTTCCGTGGGGAGCTCACGACGGCTCCGGCAGTCGAAGGTCTGGGGAAGGGCTAGGTCGGTTAGAGTTATAGCCCCGGATACGTTGGCCTGCTGGAGGGCCAGACACGTGCCTGCCCGGCTTGGCGCGCCTGTCGTCTGTTCGACTTGTGGACTGTACATGCACTGACTGCGGGGGGATTCGGGCACTCTGGGTCGGTGGCGTCGGTCGGCACCCCATCAAGCTAAATTGCGCATGGGTCTTACGAAACCTAAAGAATGAGTGAAGGCCTGCCTCTCGAGCCTAGTAGTGCGGCCTGCCGTCCTACGCCGCTCGTCCGCCGTCGGTGAGGCGGACAACGTGCACTTGGGTACCTATTCACAAGCTGGACTAGACAACTTGGTTAACTCCTGCGCTCTTCGAGTATCGTACCTGACGAACACATTAAGTCAGTGTCGATTACCTGGCGATCGAGTGCTGCCCTACTCTTATCACGATGATCTGATAATAATTTGTGAATACAACGCCGATCCGTGTCACTAGGGTCGGGTAGGACGTCGGGCCTGGGGCTTACGCCGCGACGGGAGGCCTGCGGAACTGGCCTTGGCCAGCCGGGGCGCGCAGTCATACGGATATGTTGAACGTAGATAGAAACTCGTTCCGAAACGGGGCAATGTCGCGCCCGTATCTTATGTCGTATCGACCGGCCACGGAGATGGCCCTTCGGGGTCCAGTATATAACGAACTAGAGCTCGGCGGGATCCCGGACTTGAGGCGGACTCTGTCGGCTTGCCGATGATGTTGTTCCCTACCGCTCCCCGGGCGTGCACTCCCGTCGGCTAATCTAGGGACAGTGTGATTTCGTGCCGGACTGGACCATCGATAACAGATTATCATCGAGAGGCTGCCGTCTGGGGTACGAGCGGCCTGGAACTGGCAGGGGCTGGCGAGGCCGCAAGGTCGACTCGCCGTCGCCGGGACCTTCCCGTGGACTGCTCACTGGCGGCGTTCGGGTCGTTCGCTTCGTCCGGATCCAAAGCCAATTTAACAAATGCTGTGTAGCTGATGTGATTAAAAGCCGGATCTAACGACACATTAGCTATCTGCCGACTCTTAGAGAACGAGAGCACGGGCAATTTTTTGCGTTTCTTATTTAGAAGCGGAGAGCGGGCAAGCCCTCGACTGGAACACCGGACTCGGCCTGGTGCGTCCCTCTAAAGTGTCAGCGGATAGAGCTTGAAGCAGACGCTACACATCACTTTTTCGAAGTGTTAGCCATTTATTAAGGATTATAACCCTTGATCGTCGTTAATCCCTCCCGTACATTTATTCTTGCTATAACAATTGGATTTGGATTACC

**MAGEL**_*Magelona*

????????????????????????????????????????????????????????????????????????????????????????????????????????????????????????????????????????????????????????????????????????????????????????????????????????????????????????????????????????????????????????????????????????????????????????????????????????????????????????????????????????????????????????????????????????????????????????????????????????????????????????????????????????????????????????????????????????????????????????????????????????????????????????????????????????????????????????????????????????????????????????????????????????????????????????????????????????????????????????????????????????????????????????????????????????????????????????????????????????????????????????????????????????????????????????????????????????????????????????????????????????????????????????????????????????????????????????????????????????????????????????????????????????????????????????????????????????????????????????????????????????????????????????????????????????????????????????????????????????????????????????????????????????????????????????????????????????????????????????????????????????????????????????????????????????????????????????????????????????????????????????????????????????????????????????????????????????????????????????????????????????????????????CAATTACGTAAAGGCGACATCCTAAGTAGGAATCGGATCTGGAACTTCTGCTCTTGGAGCTACGCCAGGCGAGCTCCCCTACAATGGTCCCCCGAGCTTCTCAGATAGCGGCCCGCTCCTGCCCCCTTCACT????????????????????TAATACAAATTCTAACACGGTAATGTTAATAGTTATGGTCCTTAATCGTAAAACCTACTTTTATAACAAACAACTCTGACCGCAAGGGAAGAGCCTTAGATCAAATAATCGGTGCCTTTACTGGCTTTGGGTTCAGCTTCGCCGGAGTATTTCAATTCTCCTCGTCGATGGTACGTGATATCCTACCATGTTAAAGGCCAAAACTACGCCCTGACAGGAAAACACATGGGACTCTTTCGCCCATGTTAACTTTTCCTTTAAGTCTAGTTAGACTGTCTCAGGTCTAGGCTCCGTGAACCTCACGGTCACTGGGTCCTGACCTCCATTCGGTCCCCTTGGGCTGATTAGTTCTGGTGTGCCGAAACGTAATTATCAAAAGTCTTGCCTGAATAATGGTAGGTGCCTCGGTTCTATTTGTTTTTCGGAACAGAGGTAAGGGAAGGCGGTCGTTACGGTTTTGCCGCCTAGAATACTAATCGGATTTTAAAAACATGTGCACGACAACTACGATCCCCGGATTGTTTCATGACTCGGCGGCAGCTTCCGACAAGTCTTGGCCATAGTGATTAGAATCCCCCCGCTTATTGGTGGGGCTCTAGCATAATGCGGGCAGTGCCGGAACAAGTGCGTTTCACAGTTTCGCCAAGGAATGCGTTGTCCCTGGTTCAGGATCGTCCGTTACCTCCTTCTATTGGGCTTCTCCCATGGACAGGGATCGTCCTTTTAATGGTTTGTGATCCTCGTCGACCCCTC??GAGCTTCTGCTTAGCGGGTGAGGACGATCACTTGATCATTA??????????????????AACATTTCCCTAGAGGGCTATTGAGCAAGCGAGGGCCCAACGCCGAATG?ACTAAAATATGCTTTAAGAGCACTTAAAATTTCCACCGAGTATAGGCTTTACTAAAAATTTTTTCGTATTCAGGCCTATTAATAGTTGGTGCGACCCAGGTAA????AGCTTCCTTTTCCAAAAGCACAAGCCTGTCTGAACAAATCAAGCCTTAACGGATCTTTTCCGAGTTATCTACGAAAAGG?????????????????????????????CCACAAACTAAGCCTATGGGA?GACTGCCGCCTTCGGTTGGCCGACGGA?CTGCGATAGGATTTACTCTGCTGCAACGTTCTGGCTTCCGGTCCTCCTGTCGGGGCTACATGCTCGTCACGGAGCCGGACGTTGTCGCTATGCTCCTCGCGTAGAGCAGCGTAACATCTGCTGACACTCGGATT????????????????????????????????????????????????????????????????????????????????????????????????????????????????????????????????????????????????????????????????????????????????????????????????????????????????????????????????????????????????????????????????????????????????????????????????????????????????????????????????????????????????????????????????????????????????????????????????????????????????????????????????????????????????????????????????????????????????????????????????????????????????????????????????????????????????????????????????????????????????????????????????????????????????????????????????????????????????????????????????????????????????????????????????????????????????????????????????????????????????????????????????????????????????????????????????????????????????????????????????????????????????????????????????????????????????????????????????????????????????????????????????????????????????????????????????????????????????????????????????????????????????????????????????????????????????????????????????????????????????????????????????????????????????????????????????????????????????????????????????????????????????????????????????????????????????????????????????????????????????????????????????????????????????????????????????????????????????????????????????????????????????????????????????????????????????????????????????????????????????????????????????????????????????????????????????????????????????????????

**MALDA**_*Axiothella-Clymenella-Clymenura*

AGGACAAGGAGTCATAGCACATCTAAACTATACTGTTACAGGACAACCCATTCTAGGCAGAGACCGTGTCACTATGTACCTCTTTCTATTTAAAATATTTTTATGATTGGCATAGAACTACGGGGGCACGTGGTTTCAAAATTAAAATACCTACACATTCCACACTCATAGCCAGAGCTGCTGAGAAAAAAAAAAAGACATCATTAGAAATTTAGCTTGCCCCCATCTGATTACCCTTTATTAGAAGTATCAATTGGGGCAATTTATACAAGCTATTTAGGCCTTCAACTAGAACGAATACTTATGTCATCTGTAAAAATTGCTATCATACTCCTCACAGGTAAGCCAATTACTTACACGAATCAACAGCACTTCCAGCAACCCTATCTACCACTACACAGCTTTATAGAATATGCCAAATTTGATTAATTTGATAGATAGATCGTAGTTACTCAAAGACCTATTAACAGAAACACCAATATAAGCCTAGTACTTTATGGTTGTCTCGATGTCATCACTCATTTATGTAATTCATTTCTTACTCCCCTCCATCTAAGTGATGCTTATCAGCTCAACTATGATTTCTTTACATGTTACTTTACTGACCGATGAACTAAAATTTTCTAGCTATGCAACTACCTCCAACTTACCAGCATGAATCACCT???GCAGCCCGTCGTCACGGGTGAGACTTCCTCTGCCCGGTGCTGCAAGTACTACGGAATCTTCAATCTTCCGCTAACCCTCGCCATGTGCCGTACTAGTGGTTCAGAATCGCAGATCCGCGTACTCGAGAGCCATGGGCGCCGGCAACATGAGACCTACAGTGGGCCTCTTTGATGCAAGAAGCGGTGGCCACGGAATTAACCGACTGTTCCTCGTGACTCGGCACGCTTGGAGAAGAGTGCAAATGACCTGTACGATGGCTGGGTCACTGATGGATGCCCCCAACATCGACCCACAAGAAACTGACAGGCTCGCTCTCCGGTCCGTGGATTAAGTGCCTGGTCATCAGGCTTATGGGGACATGAACCTATGACACCAGGGCGGGTCCAAGCTTGGAGGAGCTATGTCTTGACCACAACGCCCGTGGCGTCTAAATGTGTGGCGTTCCGAATTCCCTGCTGGAACTGATGAATCAAGTCGTCACCGCCTTCAAACCAGGCTATGCCATGGCCCCGTCTCCAGCAGGGAGCCCTGGAGGATCTCTCGCAGCGCGAAACTGGGACAACTGCTCACGGGTCGATGCTGCACGGGAGAGTTCTGGCATCAGTGTGAGCTTCTCC??????????????CCATTTGACCAGAACGCCGTCCTGAGCCGGCCAATATTCTGCCAGTCCTAAACTCGGAGCCGCTCCCCGCGCGCTCGCCTACCGCGCCCCCACGAGCTTCTCTGGCAGCATCCCGTCCGCGCCCTCCCCGAA??GGTTTCCTCTTATGCTGCTAATACAAGCTTTTACCCAGTAACGTTAATAGTTATGGTCCTTAATCGTACATCCTACTTTTGCAACAAGAAGCTCCGACCTTCGGGGAAGAGCCTTAGATCAAACAATCGCCCGTTTTGCTGGCTTTGGGTTCAGCTTTGCCAGAGTATTTCAATTCTCCTCTTCGTAGGTAGGTGCTATCCTACCTAGTTTAGGGCCAAACCTACGCCCTGGCAGGAAAACATACGGGACTCTATCGCCCGTATTAACTTTTCCTTTAGGTTTAGTTAGACTGTCTCGGGTCCAGGCTGTGTTCCTTTAACCAGAACTGCGCCCTGACCTCCTGCCGGTTCCCTTGGGCTGACTAGTTCTGGGTAGCCGGAACGTAATTATCAAAAGAATTGCCTGAATACTGGTAGGTGCCTCGGTTCTATTTGTTTTTCGGAACTGAGGTAAGGGAAGACGGTCGTTACGGTTTTGTCGCCTAGAATAATAATCGAATTTTAAAAACATGTGCACCCCAACTAAGATCCGCGGCTTGTTTTATGACTCGTCGGCATCTTCCGACAAGTCGTGGCCATAGTGATTAGAATCCCCCCGCCTATTGGTGGGGCTCTAGCACATTCGCCGTGTCGGTGTAACAAGTGCGTTTCACAGTTTCGCCAAGGAATACGTTGCCGTTGGTCCGAGGTCGTCCGCTACCTCCTTCTATTGGGCTTGTCCCATGGACAGGGATAGTCCTTTTGATGGTTTGTGATCCTCGTTGGTCCCGCGGGGGCAACCATCGCGGTGGCGAGGACGATCGCTTGAGCATCAAAGCAAATTTGCGGGCCTA?AATATCCCTTGGGGGCTAATGTGATAACGAAAATAAATCGCCTAATATATCTAAATTACCTTTAAGAAGGCTTAAATAAATTAAAAAACATAGGCTTGATTATTTAAATAAAAATAAT??ATTTTATTTTTAATTAACGCGGTTAAGGCAATTATAACCTCCTTTTTCTTTTTCTCTAGCCTGTCAGATTAAAAAAAGCCTTGAGCTATATCTTTTAAGTAATCGAAAAAGAAGTGGCGCAGGGGAACCCGGTAACCACCCCTTCACAAACCTTGCCCATGAGGAGACTACCGCCCCTGCTGGGGTAGCGGGAACGTGTTAGGACGCTGACTGTGG?CGACCGGGGACGTCCGGTCCTCCTGTTGGGGTTACAGACTCGTCACGGCTTCCGCGGTCGTGGCTT?AGTCCTTGCGTGGAGCAGTGTAACATCTGCTGGCACTCGGATTAACAGCGTTTAGAGGCGGATGGGACGCAAGGTTGTCCCCCGCATACC?GGTCGCGCCGCGTCGTC?CAGATCTCATGGGCGGGCGGCGGGCGCACTTGCGGGGGGAGCCCACGACGGTTCAGGCGGTCGAAGCCCGCAGGAAGGGCTCCTCGGGGGAGTGTTACAGACTGTGAGGTGTTGGCCCGCCAGGGGACTAGA??????CCCGCCGGTTGGGGCCCGCTG?TCGAACGTCCAACTTGTAGACTGTTCATGTTCAGACCGCGGA?CGGATCGGGCACTCAGGGTCGGTGGCGTCGGTCGGAACCCTATCAAGCTAAATTGCGCATGAGCTTTACGAAACTCGCAGCATGAGTGAAGGCTTGTCTTTAGAGCCCAGTAGTCCCC???AGGGGCCTACGCCGCTCGATCTTCGTAGATGAGGCGGACAACGTACACTTGGGTACCTATATACAAGCTGATCTAGACAACTTTGTTAACTCTTGCACTCA?CGAGTATCGTATATGCCGAACGCATTAAGTCAGTGTCGATTACCTGGCAATCGAGGGCTGCCCTAATCCTACAACGAGGATCTCAAAATAATTTGTGAATACAACGCCGACCCGTGTCACCAGGGTCGGGTAGGACGTCAGGCATCGGGTCGCCTCCGTGACGAGAGGTCTGTGGGCTTGGCCTTGGCTAGCTGGGGCGCACGGTCATACGGATATGTTGAACGTAAATAGAAACTCGTTCCGAAACGGGGCTATGCCGTGCCCGTAT?CTATATCGTATCGACTGGACACGGAGATGGCCCCTCGGGGTCGAGTGCACAACGAACCAGAGCTCGGCGGGATCCCGGACTTGAGGCGGACTCTGTCGGCTTGCCGCTGACGTTGTTCCCTACCGCTCCCCGGGCGTGCGCTCCCGTCGGCTAATCTGGGGA??GTGTGATTTCGTGCCAGACTGGACCAT?GATAACAGATTATCATCGAGAGGCTGTCGTCTGGGGTGCGAGCGGCGTGGAACTGGTAGGGGCTGGTGAGACCTCGCGGTCGACCCGCCATCACCGGGACCTGCCCGTGGACTGTTCACTGGTGGCGGTCG?GTCGTTCACTTCGGCCGGATTCAAAGCCAGTTTAACAAATGCTGCGTAGCTGGTGTGATTAAAATCCGGATCTAACGCAGAATTAGCTATCTGCCGACTCTTAGAGAATGAGAGCAC?GAGAATTTTTTACGTTTCTTATTTAAAAGCGGAGAGTGGGCAAGCCCTTTGCTGGAACGCCCGACCTGGCCGGGCGCGTCCATCTAAAGTGTCAGCGGATAGAGCTTGAAGTAGACGCTACACATCACTTTTCCGAAGTGTTAACTATTTATTAAGGATTATAACCCTTGATCGTCGTTAATCCCTCCCGTACATTTATTCTTGCTATAACAATTGGATTAGGATTGGC

**MOLLU**_*Chaetopleura-Katharina*

TGTATTAGCTGGTAGAGGACTGCTTAATTACATTGCAGCAGGTCAAGGCTTTATGGGGGATGACCACGTTGTTATGTACTTCTTTGTATTTCGTGTATAATAATGGTTGTCGCAGGTTAGTAGGGGTGCGTGGTTTTCGTATTAAATTACTTGCTCGGATATGTCTTGTAGCTTCAGGGGCGGTGAGAGGGGTGATGATTTTGGGGGGAATGTGGGGTGCTTGGATCTGTCTACTTTTTATTAGCAGTATCCTTTAGGGCTGTTTATACTAATTGTATAGGGATACAATTAGAGCGATTATTTGTGTTGTCTGTAAAAATTGCTATTGTATGCTTCCTTGTTATGCGAATAATGTACTTGTATTTATTTCTTTTTCTGCTAGACTATTAACCAT?????????????????????????????????????????????????????????????????????????????????????????????????????????????????????????????????????????????????????????????????????????????????????????????????????????????????????????????????????????????????????????????????????????????????????????????TCACGGGTGCGAAATCCTTTGCCTGGTACCACTAATACTATGGTGTTTCTATCAGTCCGCACTATCTTGTGACGTGCCGTGTTAACAGTTTAGCAGAATAGATTTGTGTACGCTTAAGCNACATGAGATAGCAGCAAACTACCCTCAGTCAGCCACCTTGAAACTCAAGAGGGTACTTACAGGTTCAACCAAGGCATGTTCGTAACTAGGAATACCCGGAGGTATCCTCAAACTGCGTGTTTAAGGGATGGAAAACCCTTTGAGGCTTATTCTATCCTGCCCAAAACCCCAGACAAACTCCAACTGGTATGCGTGGACCAAATACAAGGGTATTATGCTTGTGACCAAGCTGCTGTAACACACTTGGATGAGTCGAAAGTTATCAGAACTCTTTTCTTTCCGTAACGCTGGGCGAAAAAATACTGCTGGTGTAGCGAACTCTCGATGAGTGCAGCTTCATCAATGCGCCATCGCCTTGAGATCATGCTTCTCGAGTATTTTGTCCTTAATGCCAGCCCCTGGAAGGTGCAACGTACGGTGAAATTGAGACAACCAAAATGGGATCTTTGTGCCATGCCAGCATACATCCGCTAGCGTGAACCTCACAGAAACCCCACTACGCTATCAAGTCCTAGCGTAGATAAGAGCTAGCCAAGATCAAGAAACTCCAGCTCTCGATCCTGCCCCCTACGCGAGTACCTACCGTGCTCTCTCGAGCTCCTCGGGCAGCATTCTGCTCGTGCCCTCCCCGAA?CTGTTTCCTCTTATGCTGCTAATACAGACTTTCACACAGTAACATTAATAGTTATGATTCTTAATCGTACATCCTACTTTTGTAACCCGCCGCTCCGACCTGAAGGGAAGAGCCTTAGACCAAATAATCGGGTCCTGTGTTGACTTTGTGTTCAGCTTCGCCGGAGTATTTCTGTTCTCCTCTTCGATGGTAGGCGAGTTCCTACCATGCTAAAAGCCAAAACTACGCCCTGGCAGGAAAACATACGGGATCTCTTCGCCCGTATAAACTTTTCCTTTAAGTTTAGTTAGACTGTCTCGGGTTTAGGCGGAGTCCGCCTCGCGGGTACTTCGTCCTGACCTCCCTCCGGTTCCCTTGGGCTGACTAGTTCTGGGTGGCCGGGACGTAATTATCAAAAGGCGTGCCTGAATAGCTGTAGGCGCCTCAGTTCTATTTGTTTTTCGGAAGCGAGGTAAGGGAAGACGGTCGTTACGGTTTTGTCGCCTAGAATACTAATCGAATTTTAAAAACATGTGCACGACAACTACGATCCCCGGATTGTTTCATGACTCGGCGGCAGCTTCCGACAAGTTGTGGCCATAGTGATTAGAATCCCCCCGCTTATTGGTGGGGCTCTAGCATATTCGCCGTGTCGGCGTAACAAGTGCTTTTCACAATTTCGCCAAGGAATGCGTTGTCGCTGGCCCGAGGCTGTCCGTTACCTCCTTCTATTGGGCTTCTCCCATGGACAGGGATAGTCCTTTTAATGGTTTGTGATCCTCGTTGGTCCCGCCGTGGCAACAACGTCGGTGGCGAGGTTGCTCGCTTGATCATTAGAACAGATACGCAGGCTTAAAACTTTTTTTGAAAGCTTTTGTGCTTGCGTGGGCAAGACGCCTTACATAAATAAATAATTTTTAAGAGAGCTTAAAAATCTCAAAAGATATCGGCTTTAAATTTATGTAAAAATCT?TAAAAACAAAACCATTTTAGTGCGACAAAAGCAAATAAAGCTTCTTTTTAATGTAATATAAATTAGGCTGATTAAAAAAAGCCGTAACGTTTTTTTTCAGAGTCTACGAAGAAAAAGTGCGGAAAAGTAACCTGGCGACCACCTCTCCACAAACTTAGCGCATGCGGAAATGGCCGCCCACGGGCACTCGTTGGGAAAGTGATAGGACAGCCTTTGCTGCCGTGTCCGGAAGCCAGGTCCTCCTGTCGGGGCTTCACACTTGTCACGGCGATCGGCTCGACAGCTCGAGTCCTCACGTAGGGCAGCATAACATCTACTGACACTCAGATTAACACCGCCTAGAGCCGGGTGGAACGCACATTCGGCCCGCGGATACCATGCGACGCGAGTGTTTT?CCGATC???TCCTTGG?CGGTGGGCGCATTTCCGCGGGCACCCCAAGACGGTAGCGGCAGTCAAAAGTCGGCAGTATTGCGTAACCGTCGGACGTTAAAGCTGTCTTCATGCAGGCCTGTGTGGCGACCAGACGCGCGCCCGCCTGGCTCGGCACTCAG?CAATGCGTTCAAATGGGTGACTGCGCATGATCAAGCCGCGTGTCGGTGCGGTTGCAATGGGTCGTTGGCGTGGGTCGGACTCCCACCAAGCTAAGCTGCGCATTGGTGTTTCGAAACCTAAAGCATGAGTGAAGGCAGC?C?TCCT?GCCTAGTAGCCCCCCACCGGGGCCTATGCCGCTCTTCCTCCGAGGCGA?GGCGAACAACGTGGGCTTGGGTACCTACACACAAGCTGATCTAGACGACTTGGTTAACTCCTGCGCTCCACGAGTATCGTACCTGACGAACACATTAAGTCCGTGTTAACTACCGGGCGTTCGAGTGCAGCCCTACTGCTACTAATCTGATCTCACAATAATTTGTGAATACAACGCCGACCCGTGTCACTAAAGTCGTGTAGCACGTCGCGCAACGGGAAAGCTCGGCGACGGGAGGCCTGCGGAACCGGCTCGGGCCAGCTAGGGCGCGCAGTCATACGGATATGTTGAACGTAAACAGAAGGTCGTTCTGAAGCGGGGCAATTGTTAGCCCGCATTTGATATCGTCTCGACCGGACACGGAGATGACCTTCGCGGGTCACGTGCGCAACGAACTCGAGCCCGACGGCATCCCGGACTTGAGGCGCACTCTGTCGGTTTGCCGATGAGCATGTTCCCTACCGGCCTTCGGGCGTGCACTTCCGTCGGCTAATCGAGCAACGGTGTGATTTCGTGCCGGACTGGACCAT?GATAACAGATTATCATTGTGAGACTGCCGTCTGCGGTACGAGCGTTACGGGACGGGTGCGGGCTGGCGAGGCCTCCAGGTCGACTCGCCGTGGCCTCAACCTGACCGTGGACTTCTCACTGGCGGCCGGG??GCGGTGCGCTTCGTCCGGATTCAAAGTCGATTTAACGTATGCTGCGTCACTGGTGTGATTAAAACCCGGATTTAATAGATTAACTGCTATCTGCTGACTCTTAGAGAATGAAGGCACGGGTCAATTTTCACGTTTCTTATTTGTAAGCGGAGAGCGGGCAAGCCCTCGTCTGGAACGGCCGGCTCGGTCGGCCGTGTCTCGCCATAGTGTCAGCGGATAGCGCTTGAAGCAGGCGCTATACATCATTTTTCCGAAGTGTTAACTATTTATTAAGGATTATAACCTTAGTTCGTTGTTAATCCCTCCCGTACATTCATTCTTGCTATAACAATTGAATTAGGATTGAC

**MOLLU**_*Ilyanassa-Nassarius*

TGAATAATCAGGTGGTGAACAGCTTAACTTCTTTGCTACTGGACATAGCACTTCTGGTGACGACCACTTCGTGATGGACTCCTTCGAATTCTAAAAATAATGATAGTTGTTATAGTTTAACAGGAGCTCTTGGTTTTTATATTAAAACATGCTCTCTGTTACTTCTTATGTCTTCAGCCGCAGTGAAAGAAATGATGTTATTATTGGTAATTTAGCTTGCTTGGTTCAGTCTACTTTTCGTCTGATGTATCTATCAGAGCTGATTATACATATTATTTAGGGATGCAATTTGAGCGTCTTCTCATGTAATCTGTAAAAATTGCAATTATGCTTATCTTTGATATGACTATAGCTCACATGAATTTATTGCTTTTTCAGCAATATTATCCATCACTACATAACTTTATAGAAAGTGTTTAATTAGATAAACTTGATAAATAGATTACTGTAATCATTGCTAAAATTATACGAAACTCCAATACGAGCATAATATTTCTCAGTGAGCTAGATGTTATCATTCTTCTACATCATGCCTCATTAGTATCTTTTCTTCAAGATGATGCTCTTGACTTTAATTACGGTCTTATAAGTTGTAACACTAATAGTCGATGAACTAAAGCTTCTATGTTGTATAATGTTCTTCAGTCTAATGGCATAAGTTACTT???AGAAGCCATCTTCGCGAGTGCTACTTCCTCCGTCTGGCGCCGAAGATACTACGCCATCCTCTTTCTTCCGCTATTTTCTGTGACGTGCTGTACCAACGTTCCAAGAGTGCCTGCTCGCGCACCCGTGGGCCACATGTGCCGGCAACACGCCCCCCACAGTGCAGCTCTTCGAGACAGAAGTGAGCGGCCACGTACTCAACCAAGACTGGCTCGTGCCTGGGGCTTCCACGAAGAGAGTACCAATGGGTTGTTCAAGGGCTGGATGACCGTTGTCTGCTCGTTCTATCCTGCCGCAAGCCCCCGACAAACTCGCTCTGCGGTCCGCGGATTAAGCGACTGGCTGTTCGGCTCATCGGCACTCGCTCCCCTTACTCCTGGGTGGGCCGAATCTCTGTCGAGCTGTTTTCCTACCGCAACACTCGANCCGTCTTTACGTGTGGTGCAGCGAATCCACTCTGGAGTCCAAGACTCCTATGTGTCACCCCCTAGAGATCAGGAACCCCCTTGTTCCTGTCTCCAATTCCAGACTCAGGAGGATTC??????????????????????????????????????????????????????????????????????????????????????????????????AGTTCCGGCGCCGCTCCGNNNCCGCCAGCGCCCTGCTCGTCCTGCTCTCGGAGCCGCGCGCCGCGCGCCCGCCCGCCGCGCCCGCCCGAGCCTCTCGGGCAGCGGCCCGGGTCT?????????????CTGTTTCCTCTTATGCTGCTAATTCACACCCTCGTACGGTAACGTTAATAGTCGAGGTCCTTAATGATCCAATTTACTTTTGTAACCGACAGCTCCGACCCTCGGGAAAGAGCCTTAGTTCAAACAGTCGGGTCCGTTGCTGGCTTTGTGCTCAGCTCAGCCGGAGCATTTCAATTCTCCTAGACGATGGTACGTGATCTCCTACCATTACAAGGGCCAAAACTACGCCCTGGCAGGAAAACATACGGAACTCTTTTGTCCGTATTAACTTTCCCTTTAAGTTCAGTTGAACTGTCTCAGGCATGGGCCGCGTCCGCCTCGCGGGTACTGCGTTTTGGCTTCCGGTTGTTGCCCATGGGCTCACTAGCCTTGGGTGGCCGGAACGTAGTTATCAAAAGGCGTGCCTGAATAATGGTAGGTGCCTCGGTTCTATTTGCTTTTCGGAACCGAGGTAAGGGAAGACGGCCGTTGCGGTTTTGTCATCCAGAAAACTAATCGCATTTTAAGAACATGTGCACGACAACTACGATTCCTGGTTTGCTTCTCGACTCTGCGGCAGCTTCCGACAAGTTTCGGCCATAGTGATTAGAATCCCCTCGCTTATTGTTGGGGCTCTAGCACATTCGCCGTGTCGGCGCAACAAGTGCGTTTCACAGTTCCGCCAAGGAATGCGTGCTTTCTGACCCAGGGTTGACCGTTATCTCCTTATATTGGGCATCTCCCATGGACAGGGATAGTCCTTTTAACGGTTTGTGGGCCTCGTTGGTCTCGCGCCTTCACCGGGCGCCGTTGTGAGGACGCTCGCTTGATCGTTAGAA??????????????????????????????????????????????????????????????????????????????????????????????????????????????????????????????????????????????????????????????????????????????????????????????????????????????????????????????????????????????????????????????????????????????????????CTCCACAAACTAAGCTCATGCGGTCACGACCGCCCCCAGCTCGCTGGCGGGAACGTGATGGGACGCCAACTGTCGAATGCGCCGGTGTCCGAGTCCTCCTGTCGGGGCTTCAGATTCGTTACGGCCGCTGGCGCGTCGGCTCGAGTCTCCGCGTGGAGCAGCGTAACATCTGCTGGCACTCGGATTAACACCGCCTAGAGGTGGGTGGATCGCAAAGTCGGCCCGCGGATGCTGTCGGGCGG???????????GGATCGCA?????GCCCGGCCTGTGCACTTCCGCGGGGAGCCCACGACGGTTCGGGCGGTCAAAGGCGCGAGGATGAGGTGCGGCGTGCACTGGTATAGCCTCG?CCTGTCCCGATCCCTCGGGGACCAGACC????GCCGTCGGTGTGGGCCCGCCC?CGGGATGTTCGACTGGGAGACTGGACCTGTCCCGCTTCCTGGGGTGGGCCCG????CAGGGTCGGTGGCGTCGGTCGGCCCTCCACCAAGCTAAATCGCGCGTTGGTCGTACGAAACCCGAAGAGTGAGCGAGGGCCGT?C?TCTT?GCTCAGTGGTCCCGCCCGTGGGCCCACGCCGCTCGTCCGCTGTCGGTGAGGCGGACATCGTGCACTTGGGTACCTATACACAAGCTGGTCCAGACAACTTGGTTAACTCCTGCACTCAACGAGTATCGTACCTGACGAACACATTAAGTCCGTGTCGATTACCGGGCTTTCGAGTGCCGTCCTACTCCTATTACGCTGATCTCACAATAATTTGCGAATACAACGCCGATCCGTGTCACCAGGGTCGGACAGGACGCCTGGCATCCGGGAGCCGCCCAGGCGGGAGGCCCGCGGAGCCGGCCTGGACCAGTTGGGGCGCGCGGCCATACGGATATGTTGAACGTAGATAGAGAGTCGTTCTGAATCGAAGCACTGTTGTGCTCTCGTTGGATATCGTATCGACTGGACACGGAGATGTCCTTT?GGGGCCATGTGCACAACGAAGTGGAGCTCGGCCGAGCCCCGGACTTGAGGCTCGATCTGTCGGCTTGCCGATGACGGGGTTCCCTACCGGCCTTCGGGCGTGCGCTC?GGTCGGCTAATCCACGGACGGTGTGATTCCGTGCCAAGCTGGCCCATCGATAACAGATTGTCATCGAGGGACTGTCATCTGGAGTGTGAGCGTTTTGGGACGGCCGCGGGCTGGCGAGGCCCGTGGGCCGATTCGTCGCCGGTTCAACCTTCCCGTGGACCGCCCACTAGTGGCGGCGG????GTTCACGTCGGCTGGATTTAAAGTCAATCTAACAAATGCTGCATCACCGGTGTGATTAAAATCCGGATCTAACGCGGAATCAGCTATCTGCCGACTTTTAGAGAATGAGAGCGCCGGACCTTTTTTACGTTTCTTATTTACGAGCGGAAAGCGGGCAAGCCCTGGCCTGGAACTCTCGACTCGGCCGAGGGCGTCCCTCTAACGTGTCAGCGGATAGAGCTCGAAGCAGGCGCTACACATCGCTTTTTCGAAGTGTTAACTATTAGTTAAGGATTACAGCCTTTGATCGTCGTTTATTCCTCCCATACATTTACTCTTGCTATAACAATTGGATTAGGATTGAC

**MOLLU**_*Nuculana-Yoldia*

TGATTAAGCAGAAAAAGAACCACTTAGTTGAACTTTGTTACGGGTTAAGAAAGTATTCGATCCGCTAATTGTGATGTGCACCATAATGTTTAGTGCGTAATATAGTTTGATACCGCTTAATAGGTGTGCTTGTTTATATCACTTAAGTGTGGTCTTTGTTATTATTTATAATATCAGTGATGTTGATAAAGAATATAGCTTCTTTAGCTATAACTATTACGGACCAGGGTTCGCATTATACTGTATTTATCCTATGGGTCTAATTAGGCCTATTGTGAGGAAGAAACTTGAGATAAAATTGTTAGCCATGCTATTTGTGCGAGATTTGTACGACTCTTTGATGGGGGTGGAACGACCATGGCTTTATAACTTTTCCTAAATTGCGGGTATTCGTTATAT??????????????????????????????????????????????????????????????????????????????????????????????????????????????????????????????????????????????????????????????????????????????????????????????????????????????????????????????????????????????????????????????????????????????TGGGCCATCGTCACGGGCGCGACTTCCTCTGCTCCGTACCAATGATACTACACCACTCTTCCCAATCCGCCGGGCTTTGCGACGTTCTGCACCCGTGTCTCAGAACAACAAACCCGCGCACCCCTGGGCGATGTCCAGTGGCTCCAAGAGACCCACAGTGAGAAACATCAATACAAGCAGTGGCAATCACAGACTTAACTAAAGCTTGCTCCTCCCCCTCCATTCCACGAAGCAAGTGATAATGACCTGTATAAGGGATGGAAGACCGTTCGAGGCCCGCTCCATCCTGCCCATCACTCCGGACAAGGTCACTCTTCCGTCCGTGGATTAAGCTCAGGGTTATCAGGCTGATGGGGACCTGCCTCTGGTCTACCTGAGCGGACCGAGTCTCTCCGGAACTCTGCACTTTCTGGAACGCCCGAATCGCCAAAACGGCTGGAGCAGCGAACCCCCTAAGGGAGCCAAGTCTTCCACGCGCCACCACCCGGAAACAAGAATACGCCAATGTCCTGCCCCCAGCCAGGAGACAAGGAGATGCTCCCGTAGTGAGAGCCTGAGAGTACCAAGGTTACGTCCATGCGCCAGGAGACTTNACCAGCACCAGTGTGAGCGTGTGTGCAACCCCTG????AAATTTGGCCAGGGCGCCGCTCTGAGCTGGTCTTGAACAAGAACCTCCTACTCTCGGAGCCGCTCCAGACGCGCTTGCCTACAGCGCTCGCTCGAGCCCCTCCGGTAGCAGTCTGCTTGCGCCCTCCCCGCT??????????CTTACGCTGCTAATACAGACTCCAGTACGGTAACGATGATGGTCAATGTTACTGATCGTACATCCTACTTTTGCAACAACAAGCTCCGACCTTCGGGGAAGAGCCTTAGATCAGACAATCGGGGCCTTTGCTGGCTTTGGGTTCAGCTCAGCCGGAGTATTTCAATTCCCCTCTTCGACGGTACGTGATATCCTACCGTGTTTAAGGCCAAAACTACGCCCTGGCTAGAAAACATACGGGACTCTTTCGCCCGTATTAACTTTCCCTTTAAGTTTAGTTAGACTGTCTCGGGTCCAGGCCGCGTCCGCCTCGCGGGTACTGCGTCCTGACCTCCTCCCGGTTCCCTTGGGCTGACTAGTTCTGGGTGGCCGGAACGTAATTATCAAAAGAGCTGCCTGAATAATGGTAGGTGCCTCGGTTCTATTTGTCCTTCGGAACTGAGGTAAGGGAGGACGGCCGTTACGGTTTTGTCGCCTAGAAAACTAATCGAATNTTAAAAACATGTGCACGACAACTACAATCGCCGGATTGCTTCATGACTCGGCCACAGCTTCCGACAAGTTTTGGCCATAGTGATTAGAATCCCCCCTCTTATTGGTGGGGCTCTAGCACATTCACCGTGTCGGCG?AACAAGTGCGTTTCACAGTTTCGCCAAGGAATGCGTTTTTGTTTGCCCAAGGTTGTCCGTTACCTCCTTCTATTGGGCTTTTCCCATGGACAGGGATAGTCCTTTTAGTGGTTAGTGACCCTGGCTTGCCCAGTGGAGGCAACTGCCGCTGTGGCGGGCTCGGTCACTCCATTACTA???TAAATCTAAGGGCCAGATTCTACCCTTGTGGGCC?ATGTGATTGCG?GGTTGCAGTGCCTTGTGTTGTTTGTTAACTTTTAGGAGGGCCTGAATATATAGGGGGATGTCAACTTTAATTTAACTAC????CTGCTTGTGCTTGT?TAAGTTTAGTGCAACTGGGAGATAAATACTTTTAAGGAAATTTTAGCTTTGTTAATCCTGAATAAATAAGTTGGAACATATTCTTTTGGAGTATTTGATAAAAGAGTGTGAAGAAGCTATTTGGCGCCTTGTT?CTGCCAAACTAAGCCTATGCGGTGACGACCGCCCGCAGCGCGCTGCAGGGAACGTGTTGAGACGTCAGCTGTCGCCTCGTTCGGGCGCCCAGTCTTCCTGTCGGGGCTTCAGACTCGTTACGGCGCCTGACGGGGTGACTTGAGTCTTCGCGTGGAGCAGTGTAACATCTGCCGACACTCGGATTAACAGCGCTCAGAGGTGGGTGGATCGCAAAGTCGACCCGGGGGTACTGGGA??GGCGGAGGTG?G?CGGATC???CATTCTG?CGATGGGTGCACTTCCCCGGGGAGCCCACGACGGTTTTGGCGGTCAAAGCCGTCGAGAAGAGCCGCCGAGGACGGTGTTATAGATCGAGCGTAGTCGGCTCGCCGGGAGACCAGGGTTGCCGTGCTCAACTCCGG????CTC?GTGACCGTTCGACTGGCGGACTGCGCATGCGCCGACCGCGGAGCGGACCGGTCACTTAAGGTCAGTGGCGTCGGTCGGCCCTCCACCAAGCTAAATTGCGCATGGGTTCTACGAAACCTAAAACATGAGTGAAGGCCGC?C?TCCG?GCCTAGTAGTCCGTATCGGCGGCCTACGCCGTCCGTCAGCCGTCTGTGGAGCGGACAACGTACACTTGGGTACCTATATACAAGCTGGTCTAGACAACTTGGTTAACTCCTGCACTTTACGAGTATCGTACCTGACGAACACATTAAGTCCGTGTCGAATATCGGACATTCGAGTGCTGCCCTACTCCTATTACGCTGATCTCATAATAATTTGCGAACACAACGCCGATCCGTGTCACTAGAGTCGGATAGGACGTTGGGCATCGGGGTGCCTCGGCAACGGGAGGCCCGCGGAACCGGCCTGGGCTAGCTGGGGCGCGCGGTCATACGGATATGTTGAACGTAGATAGAGACTCGTTCCGAAACGGGGCAATGCCGAGCCCGTCTATCATATCGTATCGACCGGACACGGAGATGGCCCTCGCGGGTCATGTGTACAACGAACTGGAGCTCGGCGGGATCCCGGACTTGAGACGGACTCTGTCGGCTTGCCGATGACGTTGTTTCCTACCGGCCTTCGGGCGTGCACTCCCGTCGGCTAATCCAGGGACAGTGTGATTTCGTGCCGGACTGGCCCATCGATAACAGATTATCATCGAGAGGCTGCCGTCTGGAGTTCGAGCGGACTGGGACGGGCACGGGCTGGCGAGGCCTCGCGGTCGACTCGCCGTCGCTTCAACCTTCCCGTGGACAGCTCACTGGCGGCGGCGG???CGTTCGCTTCGTCCGGAACTAAAGCCGATTTAACAAATGCTGCGTCACTGGTGTGATTAAAATCCGGATCTAACGCAGAATCAGCTATCTGCCGACTTTTAGAGAATGAGAGCACGGGCAATTTTTTACGTTTCTTATTTATAAGCGGAGAGCGGGCAATCCCTCGACTGGAACCACCGACTCGGCCGGTGGTGTCCCTCTAAAGTGTCAGCGGATAGAGCTTGAAGCAGGCGCTATACATCACTTTTTCGAAGTGTTAACTATTTATTAAGGATTATAACCTTTGATCGTCGTTAATCCCTCCCTTACATTTATTCTTGCTATAACAATTGGATTAGGATTGAC

**MYZOS**_*Myzostoma*

?CTGTTATCTGATTATGTTCCTCATAGATACATAACTATAGGTCAGTTCCTTGGTTTAAGTGATCATATTACAATGTTCTTCTTTGTATTCTAAAAATAATAGTAATTGGTCTATAACTATAAGTACCCTTAGATTCATAATCCAAATAAATACACAGAAAATTTACATATCATCATCAATAGTGAAATATTATAAGCCTTCATTAGGAATATTGCTTAGAGCCTTCTGTTTGTATTCCTTTAGAAGTCAGACTTAGAGCTATTTATTCAAATTTTTTAACAATAAAGTTTGAACGATTATTTATACAAGCAATACTTTAAGTTACCTTATGTATCATAGATGAGACTATTACATACTTGTACTTATAGCATTTTCAGCTAAGTTATTAACCAC???????????????????????????????????????????????????????????????????????????????????????????????????????????????????????????????????????????????????????????????????????????????????????????????????????????????????????????????????????????????????????????????????????????????AAGGAGGTTCATCCTTACGAGTGCAAAGTCATTTGCATGATGCCGTGAGTATTATGGCATTTTTCTTAATCCGTAATATTTTATGGTTTGCTGAACTAATATTCAAGGATGGACGATCCTTGTATATGTGGAAGATGTCTGGTAGCACCAAGAGACCATCAGTGAGGCTCATCAATGCAAGAAGGCAAGGATTGGGATTTAACGAAATCTTGATTGTCTCAGTCCATATTATGATGCAGAGACCCCTTCCTTGTACAAGGGATATAAAAATGCTGTCTGCTTATAACATTCTTCCATACTAGCCCGATAAGCGCCAACTTTGATGCGTGGATTAGGCTAAGTGTTATTACGCATATGGTGTCTCGATGCTGGGCTTCGTACGTGTGTTGAGAGTTAACTGAGCTCTTTTCTTCTTGCAACAATGGTATAAGAAGAATAGCTGGTGTAGCGAATTCTCCAAGGAAGCTAAGAATTCACAGAGCTATCCTTCGCAGATAGAGCTTTGCTTTCCTCTCGGTTCCAATGTAGAATGAAAGAGGTGTTCTCGTACTGCGAAACTCGGATGCTTCAAACAGAATCACCTTGCTGCGCATTAGAACATCAACCAGTGTGGTCTTCAGTACAATCCATTG???CAATCAAGCCCAGGCCCGGCTCCGAGTCTTATTACGACAAGCGATTTCTGCTCTCGGAGCCGCGTGCCGCGAGCTCTGCTGTTATGATTGAACGAGCCACCCTGGTAGCGGATCCATCGCGCCCTCTTCGCA?CTGTTTCCTCTTATGCTGCTGATACACATTCTCGCAAAATAATATTTAAGGTTATGGTTCTTGGAAATAGATCCTACACTTATAGCTACATCATCCGATTCA???TCGGGTGACAGTGATCCAACAATTGCGTCTTGGGCTGGTCTA??GATTGGCTTCGCTGAATCATTTCAATACCACCCTTCGAAGGTAGGACATTGCCTACCTCGTTAAAAGCTAAAACTACGCACCGACAGAGAAACAAACGGG??GGCATCATTCGTATAAGCTTTCTCGCTATTTATGGTTAGACTGTCTCAGTGTGACAGGCCGTCCACTTAACGGG?ACTGAATGTCAGCCTTTTCACGGTGGAGTAGAACAGATTCGTTCTTTCCATCCGCGACGTACTTGCTCAAAGTTTTGCCAGAACATTTATAGATTCCTGATCATATTTCGTTTTGGTGCGATCAGGTGAGGGAATACGATCGTTGTGGCGGACTCGCTCAGCATAATGACTGCGTTTCAAAGACATCTGTGCCATGGCTGCGATCTCTGATATGATTAACAGACCAGTGGCAGCCCTCGACAAGCTTTGGCCATAGTGATTAGGTTCCCATCGTATATTGGCTAAGCACTAGCGCCAGACCCCCGAGAGGGTCACAATCGCATTTTGAAGGATCTCCAAAGATAACGGTATCCATTGCTCGAGATTGTCC?TGGCGTCTTTCCATCGGGACTATTCCGTGGACAGGGATCGTCCTTTTGATTAGTAGTGGAGTTTGCCTAGTCGAC?GGATCGGCTTTTGCGAC???GGAGAACGCCATGTATTTGTCAGAATAATAATATAT?ATTGAAATTTCCTTTGAAGGATTTTGGAATAACAAGAAATTATCGCCTTTTATAAATAAATAATTTACAAGAAAGCTTGTTAAAATAAATAAGTGTTGGCTTTTTCTAATAAA????????????????????TTAGATTTGTGCAACATATATTTAAAAACTATTTTTTTA?TTTAGAACAAATTCTTTATAAAATAATAAGCCACAAGCTATTTTCTTTTAGTTAATAACAAGAAAGTGGTGCAAAGTATCTTTGT??????????TCACAAGTTAAACTCACCAGCAGACAAGTGCCGGCGTTACGAACGTGTGAATGTCTATGG?CGATGTCGAAGGCGGTTTGTCGGATCTGTATCGTTTGGACAATGAC??ATAAACTCGGTGGATTCCGCGACCGCGAACGAGGCCAGAAAATGATGCGTCGAAACTCGTGCGAATACACAAATTAAAACGATATGGTGG?GGATAATGAGCTGAGATGG?CTGGCGAGACG?????????????????????????????????????????????CGTTACGAC?????GTTCGACGCGGTCG????????????????????????????CTTGCGCGTACGCTACCGCCGTA????CACGGACGCG???????ATCAAA????CGGCTACCC??TTCGG????????????????????????GAAAGCATGTCCGCGTCGTCTATG????????????????TTGGGTGGGTAGCGCGCGCAAGCGATCTATCAAGCTAAATTACGTGCGGACAAG???TGAAGCCTTAGATGAGTGA????????????????ATGACTGTTCCCG?TGGCGGGCGCACG?CGTC????????????TACAAGAACACGATGCACATTTGGTTACCTACGTATAGGCTAGCTAAGAAAGTTTGGTTAACTCCTGCGCTTTAAGAGTATTGTATCCGACGAATTCATTAAGTCTCTATTGATTATGTGACGATAGAGTGCAGTCACGCTACCATAATGA?GATCTGATCACAATTTGTGAATACAACGCTGATCCGTGTCACTAGAATCTAAGAGTACGTCTTGTACTTGTTGCTTACAAAGACGGGAGG?CAATTCGGTGAGCTCTGCCCAGGAAGGGGGGATCGTCATGCGGACGCGTTGGATGTGGAATCCTAACGTTAATGTGGC?GGACGATCC???GCACGT???TGCCATGATATTCACTGGTATCTTCACGAACT????GATGTTCAGAGTACAGTGAACCGGGGCCTATCGGTACTCCACAATGTTGGTGCTGACTATCGTGTCATTGCTGTTCTGGT?CCCTGCTACCTTCCAGGTGTGCGCTATCGATGGCTAATTCGGGG??AGTGTGAA???GTGCTAGGCATGCACGCCGATAATAGATTCTTATCTTAGGACTGTCCACATGATTGGAATGTGCCCCG??CAGTCAAG???CCGCGTTTTTGGGGGTTTAACGCAGTGTTTTGGGGAGT??CC?ACGACCGGCTTTTTG??GGTTG?????TCGCAACCCTCGAAAGAGCCTAAAGTTAATCCAACAATTGCCGCAGCAATGGTGAGAATAAAACCTGGATCTAACGCAAAAACAGCTATCTGCTGACGTCTAGGAAATCAGAGCGAGTGACTTTTCTGGCGTTCCTCATTAGAAAAAGAAAGTCGCGCGCG??ACATTCTAGACTGCTCTCG????GAGTGCGTGTCTAC?CGATCCATCAGCGGATAGGACTTGAATGAAATCTTACATATTACCTTTTCGAAGTGCTAACTG?TTATTAAGGATTATAACCCTTGTTGGTTGTTAATCCTCCACGTACATTCATTCTTGCTACTACAATTGCATTCGAATTGAC

**NEPHT**_*Nephtys*

??????????????????????????????????????????????????????????????????????????????????????????????????????????????????????????????????????????????????????????????????????????????????????????????????????????????????????????????????????????????????????????????????????????????????????????????????????????????????????????????????????????????????????????????????????????????????????????????????????????????????????????????????????????????????????????????????????????????????????????????????????????????????????????????????????????????????????????????????????????????????????????????????????????????????????????????????????????????????????????????????????????????????????????????????????GTCACGGGAGCTACTTCCTTTGCTCGGCGCCAACGATACTATGTCATCTTCTCTCTTCCGCCAAACTCTGCGATCAGCTGTACTAGTGCTCAAGAATCGTCCGTCCACTCACTCAAGGGCTACACGTGTCGGCTCAACGAACCCAACTCAGGCCCTCATTGAAGCAAGAGAGGCTACCTGCAGATACAACAGACACCTCATCGTCATCAGGCATATCTGGAGGCATCACAAAATGAGTTGTTCAGTGGATGGGTAACCCCTCCAGGCTCTCAACATCGATCCACCAACTTCTGGCCGCCCCGCTCTATCGTACATGGATTAGCTTCCTTGCTATCCGGCTCATGGTGACCCGCCGCTAATGAACATGGGCGGGCCACTGCCCTGAAGAGCTCTTATCTACCCGGAATGGTCGTATCGCCTAAATTGTCTCCGCTCAAAGTTCTGCAAGGAAACAGAAGAGACAAAGCGCCACCGCCTCCAGACTCCGCTACTCCAAGGTCCAGTCCCCAGCAGGGTTCCCTGGAAGATTTCTCGTTCTGCGAAGCTTAGATAACACAGCACGCATCTTTGTGCCAGGTGAGAGTCCTCAGGCCAGTGTAGGCCTCAGTGACATGCCTCCGCG????AAAGTTCCGGCCCCGATCCATCTCTGCCTACGCTCTGAAATTCCTGCTCTCGGTCCCGCCCGCCGCGCGCCCGGCTACCGCGATCGCACGAGCTCCCCTGGCAGCGGTCTGCTCCCGCCCT???????CTTGTTTCCTCTTATGCTGCTAATACAAACTTTTACACAGTAACGTTAATAGTCAGGATCCTTAATCGTACTTCCTACTTTTATAACACGAAGCTCCGACCTTCGGGGAAGAGCCTTAGATCAAACAATCGGTCCCTTTGCTGGCTTTGGGTTCAGATTATCCGGAGTATTTCAATTCTCCTCTTCGTTGGTACGTGATATCCTACCAAGTTAAAGGCCAAAACTACGCCCTGGCAGGAAAACATACGGGACTCATTCGCCCGTATTAACTTTTCCTTTAAGTTTAGTTAGACTGTCTCGGGTCCGGGCCGCGTCCACCTCGCGGGTACTGCGTCCCGACCTCCTACCGGTTCCCTTGGGCTAACTAGTTCTGGGTGGCCGGAACGTAATTATCAAAAGGCTTGCCTGAATAATGTTAGGTGCCTCGGTTCTATTTGTTTTTCGGAACTGAGGTAAGGGAAGACGGTCGTGATGGTTTTGTCGCCTCGAATACTAATCGAATTTTAAAAACATCTGCACGACGACTACGATCCCTGGATTGTTTCATGACCCAGCGGCAGCTTCCGACAAGTCTTGGCCATAGTGATTAGAGTTTCCCCTCCTATTGGTGGGGCTCTAGCATATTCGCCGTGTCGGCGTAACAAGTGCTTTTCACGGTTTCGCCAAGGAATGCGTTTCCG?TGCTCCGGGGGTGTCCGTTACCTCCTTCTATTGGGCTTGTCCCATGGACAGGGATAGTCCTTTTAATGGTTTGTGATCCTCGTTGGACCCGCGTGGGCAACCGATGCGGTGCAGAGGACGATCACTTGATCATTAAAA??????????????????????????????????????????????????????????????????????????????????????????????????????????????????????????????????????????????????????????????????????????????????????????????????????????????????????????????????????????????????????????????????????????????????????CTCAACAAACTAAGCCTACGCGGATACGACCGCCCCTGCCCAGGTAGCGGGAACGTGTTAGGACAGCCTATGGGGTGGTGTTCGGACGTCCGGTCCTCCTGTCGGGGCTTCATACTCGTCACGGCGACCGTCGCCTCCCCTTGTGTCCTTGCGTGGAGCAGTGTAACATCTGCTGACACACGGATTAACATCGTTTAGAGGCGGATAGGACGCAAAGTCTACTCGTGGATACTTGCGCG???GGCAGGTGTCCAGATCCGTTGTCCGGGCGGGGGGCGCACTTCCGCGAGGAGCCCACGACGGTTTCGGCAGTCAAAGCCCCGAGTATGAGCTTCGGGCAGAAGCAGTATAGACTCGGTGGCCATGGCCTGCTGGGGGACCACCCTCGCTCCCGCCCGGCTCGGCGCTGTGAGAGCTCGACCAACTGGAGGACTGTGCATGCTTCGACCGCGCTAGGCACGGGTCGCTCAGGGTCAGTGGCGTCGGTCGGCACCCTATCAAGCTAAATTGCGCATGGGTTCTACGAAACCTAAAACATGAGTGAAGCCGCTCCTCTAGTGGCTAGCAGTCCTCCCTGGGGGCCTGCGCCGCTCGTCTGCCGTCAGTGAGGCGGACAACGTACACTTGGGTACCTATATACAAGCTGGTCTAGACAACTTGGTTAACTCCTGCGCTCTTCGAGTATCGTACCTGCCGAACACATTAAGTCCGTGTCGATTACCGGGCGATCGTGTGCTGCCCTACTTCTATTACGAGGATCTCATAATAATTTGTGAATACAACGCCGATCCGTGTCACTAGGGTTGGATAGGACGTCAGGCATCGGGGAGCCTCCGTGATGGGAGGCCCGCGGAACAGGTCTTGGCTAGCTGAGGCGCGCGGTCATACGGATATGTTGAACATAGATAGAAATTCGTTCCGAAACGGGGCAATAATCGGCCCGTATTTTATATCGTATCGACTGGCTACGGAGATGATCCCTCGGGGTCCAGTGCACAACGAACCAGAGCCCGGCGGGATCCCGGACTTGAGGCGGACTCTGTCGGTTTGCCGATGACGTTGTTCCCTACCGCTCCCCGGGCGTGCACTCCCGTCGGTTAATCTGGGGATGGTGTGATTTCGTGCCAGACTGGACCAT?GATAACAGATTATCATCGAGAGGCTGCCGTCTGGGGTACGAGCGGCCTGGAACTGGCAGGGGCTGGCGAGGCCAAGGGGTCGATTCGCCGTCGCCGGGACCTTCCCGTGGACTGCTCACTGGCGGCGGTCGGGTCGTTCGCTTCGTCCGGATCTAAAGCCGATTTAACAAATGCTGCGTAGCTGGTGTGATTAAAATCCGGATCTAAATCAGAATTAGCTATCTGCCGACTTTTAGAGAATGAGAGCACAGAAAATTTTTTACGTTTCTTATTTATAAGCGGAGAGCGGGCAAGCCCTCCTCTGGAACGTCCGACTCGGCCGGGCGCGTCCCTCTAAAGTGTCAGCGGATAGAGCTCGAAGTAGGCACTACACATCGCTTTTTCGAAGTGTTAACTATTTATTAAGGATTATAACCCTTGATCGTTGTTAATCCCTCCCGTACATTTATTCTTGCTATAACAATTGGATTAGGATTGAC

**NEREI**_*Ceratonereis-Nereis*

????????????????????????????????????????GGACAACGCATTKCAGGAAGAGATCAATTTGTTATGTCCCTCATTTGACCTTAAGTGTATTTATTGTTATCACTAATTATTAGGTGCTCTTAGATTCAAAATTAAAATACTTACGCCTTCTATTCTCTGATCTTCTGCAGCAGTGAACAAAGAAATGACCACTGAGCCAATATTTCACAGATCCCTCTGACCTCTTCTTATCTGTAGTTTCTTTTAGATCAATTTATACAAGTACGATA????????????????????????????????????????????????????????????????????????????????????????????????????????????????????????????????????????????????????????????????????????????????????????????????????????????????????????????????????????????????????????????????????????????????????????????????????????????????????????????????????????????????????????????????????????????????????????????????????????????????TCACGGGTGCTACTTCCCTCGCTCCGTGCTGCCRGTACTACGACATCYTCTCTCTTCCGCTATCTTTGGTGACCTGCCGTACTTACATTCCAGAAYCGCCTGCTCGCTCACCCTAGGGCCACGTGAGGCGGCTCTATGARACCAACAGCAAGRCTCATTGAGACCAAAGACAGCACCCACGGAYTCAACTGACACCTGCTCGTGCCTTGGAATATCTGGAGACATCTAGTAATGACCTGTTTAATGGCTGGAAGACCTCTCGAAGCCCGCAACATTGATCCYATCTCTTCCGATAAAGCCGCTCTTCGGCACAAGGATTAAGYTACTRATTATYCCGCCTATGGTGCAGCGCTNGKKGKMYGCCTGCRTGGGTCACAGCTCTGAGCAKCTRTGTTCMTTCCGGAACACTCGTACCGTCTYAATTGCTTCTGCAGCGAATTCWGYMGKGAAACCGATAGCTCAAGGTGCYACCCCCTAGAGACCGTGCTCCGCGMCATTCCTGTCTTCAGTGCTGAGCCAAGGAGGATTCCTCGTTCCGTGAAGATGAGACAACCGAGAAGGGGTCTTTGTGCCACGCCAGAGTCCCCAGGGCAGCGTCGGCCTCAGCGACATGCTCCT???????CACGCCAGGGCGCCGTCCTGAGCCGGCCATGGCTCTGCACCTCCTGCTCTCGGTCCCGCGCCCCGCCCGCTCGCCTGCCGCGCTCGCCCGTCCTCCTCGGGTTCTGGTCCGTTCGCGCCCC???????CCTGTTTCCTCTTATGCTGCTAATACATACTTTTACACAGTAATGTTAATAGTCCAGGTCCTTAATCGTATATCCTACTTTTGTAACATTAAGCTCCGACCTTTCGGGAAGAGCCGTAGATCAAACAATCGGGTCCGTTGCTGGCCTTGAGTTCAGCTCGGCCGGAGGATTTCAGTTCTCCTCTTCGATGGTACGTGACCTCCTACCATGTTAAAGGCCAAAACTACGCCCTGTCAGGAAAACATACGGGACTCAATCGCCCGTATTAACTTTTCCTTTAAGTCTAGTTAGACTGTCTCTGGTCCAGGCCGCGTT?GCCTCGCGGGTACTGCGTCCTGACCTCCAACCGGTTCCCTTGGGCTTACTAGTCTTGGGTGGCCGGAACGTAATTACCAAAAGATTTGCCTGAATAGTGGTAGGTGCCTCGGTTCTATTTGTTTTCCGGAACCGAGGTAATGGAGGCCGGTCGTTACGGT?TTGTCGCCTAGAATACTAATCGAATTTTAAAAACATCTGCACGACGACTACGATCCCGGGATTGTTTTATGACCCCGCGGCAGCCTTCGACAAGTCTTGGCCATAGAGATTAGAATCCCCCCGCTCATTGGTGGGGCTCTAGCATATTCGCCGTGTCGGCG?AACAAGTGCGTTTCACGGTTTCGCCAAGGAATGCGTTGTTGTTGATCCCAGGCCGTCCGTTACCTCCTTCTATTGGGCCTATCCCATGGACAGGGATAGTCCTTTTAATGGTTTGTGATCCTCGTGGGTCCCGCGGGGGCAACCATCGCGGAGCCGAGGACGATCACTTGATCATCAGAACAGATCCGCGGGCCAAACAGTTCCTTCAAAGGCTATTGTGACACCAAGAATAAAGCGCCTTAAATTTTTATTCGTATGACAGGAATTCCTGTAATCAATAAAAAGTATAGGCTTTATTTACAGCAGATAC???????????????GTAAAATAGTACAACTAAAACACGTACACCTTTTTACTA????TACACAAAAC????ATGTAGATCACAGCCTTAAGCTATTTTGCTAGAGACACTGACAGCAAAGAGGCGCAGTGTAACCTTGTAACAATCA????????????????????????????????????????????????????????????????????????????????????????????????????????????????????????????????????????????????????????????????????????????????????????TTAACAGCGTTTAGAGGCGGATGGGACGCTAAGTCGGCTCGGGGATGCTGGCGCG???G?GCGGAACGCGGATCTTCCGTCTGGGCGGTGGGCGCACTTCCACCAGGATACCACGACGGTTTCGGCGGTCAAAGTCCGTGGCAAGGGCTCTTCGGAGGAGTGTTATAGGCCGCGTGACATTGGCCCGCTGGGAGACCAGACACGCGCCCGCCCGGCTCGGCCCGCCTTGTTGCCGTTCGACTGGAGGACTGTACATGCTCCGACCGCGCGAGGACTCGGTCGCTCAGGGTCAGTGGCCTCGGTCGGCACCCCATCAAGCTAAATTGTGCACGGGTTCTACGAAACCTAGAGCATGAGTGAAGGCCTTCC?TCCTGGCCCAGTAGTCCCCCTTTGGGGCCTACGCCGCTCGTCCTCCGTAGGTGAGGCGGACAACGTACACTTGGGTACCTGTACACAAGCTGGTCTAGACAACTTGGTTGACTCCTGCACTCTTCGAGTTTCGTATATGACGAACACATTAAGTCCGTGTCAATTACCGGGCGATCGAGTGCCGCCCTACTCCTATTACGATGATCTCATAATAATTTGTGAATACAACGCCGATCCGTGTCACTAGGGTCGGATAGGACGTCGGGCACCGGGGCGGCTCTGCGACGGGAGGCCTGTGGGACAGGCCTTGGCCAGCTGGGGCGCACGGTCATACGGATGCGTTGAACATAGATAGAAACTCGTTCCGAAACGGGGCAATGCCGTGCCCGTACTCTATATCGTATCGACCGGATGCGGAGAGGGCCCTTCGGGGTCTAGTGTGCAACGAACCAGAGCTCGGCGGGACCCCGGACTTGAGGCGGACTCTGTCGGCTTGCCGATGACGTTGTCGCCTACCGCTCCCCGGGCGTGCACTCCCGTCGGCTAATCTGGGGGTGGTGTTATTTCGTGCCGGACTGGACCGC?GATAACAGATTATCATCGAGAGGTTGCCGTCTGGGGTACGAGCTGCGTGGAACTGGCAGGGGCTGGCGAGGCC?TAAGGTCGACCCGCCGTCGCTGGGACCTTCCCGTGGAATGCTCACTGGCGGCGGTCGGGTCGTTCGCTTCGGCCGGATCTAAAACCGATTTAACAAATGCTGCGTAGCTGGTGTGATTAAAATCCGGATCTAACGCAGAATTAGCTATCTGCCGACTCTTAGAGAATGAGAGCGCGGGAAATTTCTCACGTTTCTTATTTAAAGGCGGAGAGGCGGCGGCCCCTCGAGTGGAACCGGCGACTCTGCCGCCGGCGTCCCTCTAAAGTGTCGGCGGATAGAGCTCGGAGCAGGCGCTACACACCGCCCATTGGAGGTGGTAACTGATCGTCAAGGATTATAACCCTTGATCGTCTTCCACCCCTCCCGTGCATACATTCTCGCTACAACGATTGGATCACGATTGAA

**NERIL**_*Paranerilla*

??????????????????????????????????????????????????????????????????????????????????????????????????????????????????????????????????????????????????????????????????????????????????????????????????????????????????????????????????????????????????????????????????????????????????????????????????????????????????????????????????????????????????????????????????????????????????????????????????????????????????????????????????????????????????????????????????????????????????????????????????????????????????????????????????????????????????????????????????????????????????????????????????????????????????????????????????????????????????????????????????????????????????????????????????????????????????????????????????????????????????????????????????????????????????????????????????????????????????????????????????????????????????????????????????????????????????????????????????????????????????????????????????????????????????????????????????????????????????????????????????????????????????????????????????????????????????????????????????????????????????????????????????????????????????????????????????????????????????????????????????????????????????????????????????????????????????????????????????????????????????????????????????????????????????????????????????????????????????????????????????????????????????????????????????????????????????????????????????????????????????????????????????????????????????????????????????????????????????????????????????CTTATGCTGCTAATACAAACTTTAACACAGTAACGTTAATAGTTATGGTCCTTAATCGTACATCCTACTTTTATAACAA?AAGCTCTGACCTCTGGGAAAGGGCCTTAGATCAGACAATACCGTGGTTTGCTGGCTTTGTGTTTAGCTTTGCCGAAGTATTTCAATTCTCCTGTTCATTCGTCGGTGCTATCCTACGAACTTAAAAGCCAACACTACGCCCTAGTTGGAAAACATACGGGACTCTTTCGCTCGTATTAACTTTTCCTTTAAGTTTAGTTAGACTGTCTCAGGTTCGGGCTGCGTGCTCCTCGTAGGTTCTGCGACCCGGCCTGCTTTCGGTGCACAGGGGCTAGTTAGTCTTGTGTGTCCGTAACGTAATTATCAAAATTACTGACTGAATAATGGTAGGTGCCTCGGTTCTATTTGTTTTTCGGAACTGAGGTAAGGGAAGACGGTCGTTGCGCCTTTGTCGGCCAGAAAAGTAATCGAATTTTAAAAACAAGTGCACGACAGCTACGATCCCCACATTCATTTATGACTCGGCGGCAGCTTCCGACAAGCCTTGGCCATAGTGATTAGAATCCCCCCGCTTATTGATGGGGCTCTAGCACATTCACTGTGTCAGTGCAACAAGTGCTTTTTACAGTTTCGCCAAGGAATGCGTTTTGCTTGGTCCGAGGCTGTTCGTTACCTCCTTCTATTGGGCCTGTCCCATGGACAGGGATAGTTCTTTTAATGGTTTGTGATCCTCGTTGGACTCGCGGAGGCAACTCTCCCGCTGTCGAGGACGATCACTTGATCATTA???CAAGTATGTCTGGGAGAAATTTTCGCTAGTGACCTTTCCAA?GGTTTGAGTAATATTTTTTTTA?AGAAGGATAGAGTGAAAGAATCCTTTTAAGTTTATAAAGATATAGGTTTTATTTTTAAAAAGCAGGTAGTTGTGAGGATGAAAGATTAGAGCATCTCCAGCTT????AGTTTCTGGCAGCGGGGGTATATGTTAGA?TGTAAATAATAAATCTTAAGTTATAGTTTTTTAGATAATGACAAAACTGTAATGCAGGAAAAGCTCGGACCGATTT??????????TAATCCTATGCGGAAACGACCGCGCCTGCCTGGGTAGCGCGAACGTGTTAGGACAGCGATTGCGGTGACGGTTGGAAGCCTAGTTCATCTGTCATGGCTTCATATTTGTGAGGGCATCTGCCGCTGCCGCTTGAGTCCTCGCGTAGAGCAGTGTAACACCTGCTGAGACACAAATTAACATTGTTTAGAGGTGGATGGGACACTGGGCCAG?TGTGGGATGCT??ACTGCTGGGGCTGGGTGCGGAGCTTTTGGACGGACGGCGGGTGCACTTCCTGCAG??GCTCACGGCGGTTTTGGCGGTCAACGCTTGGGAGAAGAGGTGCGGTGTTTACTGTTATAGCTCCT?GGTTGCTGGCCTGCTGGCGGACCAGATCCGTACCTGCCC?TCGCGG?CTACCGGTCTTTCGGCTACCTGGTGGACTGCGCATGCGCTAACCGTCGTCATGTTCGGCTACTTCAGGTCAGTGGCGACGGTTGGCACCCCATTAAGCTAAATTGCGCATGGGACCT??ACGAATCCAAGCATGAGTGAAGGCTTCCCATTAGAGCCTAGTAGTCCTG?CTGTGGGCCTACGCCGCTTATTCGC??GTGGAAAGGCGGACAACGTACATTTGGGTACCTATTTACAAGCTGGACTAGACAACTTGGTTAACTCCTGCGCTCT?CGAGTATCGTGCCTGACGAACACACTAAGCCTTTGTTAATTACTTGGCGATCGAGTGCCGCCCTACTCTTATGACGATGATCTCATAATAACTTGTGAATACAACGCCGATCCGTGTCACTATGGTTGGATAGGACGTCGGGCAATGGGGTGCCTCCTCGATGGGAGGCCTATGGGACAGGCGTTGGCTAGCTCGGGCGCATGGCCATACGGATATGTTGAACGTAGATAGCGATGCGTTCCGAAACGGGGCAATGCCGTGCCCGTCACCTATATCGTATCGACAGGACACGGAGATGGCCCTTCGGGGTCCAGTATATAACGAACTAGAGCTCGGCGGAACCCCGGACTTGAGACGGACTCTGTCGGTTTGCCGATGACGTTGTTTCCTACCGCTCTCCGGGCGTGCGCTTCCGTCGGCTAATCTAGGGACAGCGTAATTTCGTGCCAGGCTGGACCRTCGATAACAGATTGTCATCGCGAGGCTGCCGTCTGGGGTTCGAGCGGCCTGGGACTGGCTAGGGCTAGTGGTCGCGTGTGATCTTCTCGCCGTCGCCGGGACCTTCTCGTGGACTGCTCACTGGCGGCGG???TGTCGTCCGCTTCGTCCGGATCTAAAGCCAATTTAACAAATGCTGCGTAGCTGGTGTGATTAAAATCCGGATCTAACGCAGAATTAGCTATCTGCCGACTCTTAGAGAATGAGAGCACGGGCAATTTTTCACGTTTCTTATTTATAAGCGGAGAGCGGTTGTGCCCACGCCTAGAACGCCTGGCTTGGGTCGGCGCGTCCCTCTAAAGTGTCGGC?????????????????????????????????????????????????????????????????????????????????????????????????????????????????????????????????????

**OENON**_*Drilonereis*

TGCACTAGGAGCCTCAGAACCTCAAAACTACCCAGCAACAGGCCAAAGCATTCCAGGAAGCGACCACACTACTATGACCCCCATCCAACCCCACACATCCTAACAGTTGACACAGTTCCACTGGCGCACACGGGTTCAATACTAAAACATATACGCCGTCGACCCTTAGCAGATCCGCTGCAGAGAAAAAAAAAATGACCACAGCTCTAACATTGCCTGCACCCCTCAGACCTCCCTACACTAGCTGCCTCCTTTAGCGCGCACTACACCAGTAATCTAGGACTTCGCCTTGAACGAATCCGTATGTGAGCAGCCTTCACAGTAACCTTACACATCCCAGACTCGCCCACAACCCGCACGAACCTATAGCTCTTCCTGCCCTTCAATCAATCACCCCATAACCCCATTGTAAACGACCTCCCAGATAAACTCGAACAATGGCTAACCGCCACCACATCACGAATTATACGAAACACCCATACAAGCACACTAYTCTATGGTAAGCAACGAATCTGCCTTCATTCATATTGTACTTCACCAGTACTCCTATAAGAAAATGCCGCCCACCAGATCACGCATGATTTCTCATCAAATAACGCTCCTCAACGATGAGCAAAAGTACCTTCCTAACATTTCCGCCCSCAACCCATCAAGC??????????AGGGAAGCACATCGTCGTAGGCGCTACTTCCTCTGCCTGGTACTGCCAGTACTACGTCATCTTCTTTCTTCCACTCCCTCTTGCGACTTGCTGTACTCGTGTATAAGAACCGCCCGTCCGTGCTCCCTGGGGCAATGGGTGGCGGCTCGAAGAATGGCACAGTAGTACCCTTTGATACAAGAAGTGGAGCCCATGGATTCAATTGACGCTTGTTTGTGTCTTGGCATTCCAGGAGGCGTCAAGCAATGACCTGTACAAAGGCTGGACTAACGCTGGAGGCCCCCAGCATTGACCCCCTCTCGACTGACAAGCACGCTCTTCTGTCCGTGGATTCGGCTACTTGCAGTTCGGCTTATGGGGACCTGCTGCTGCACCAGCTACGTGTACTACAGCTTGGAGAAGCTGAAACTTGACCGCAACACCTACACCGTCTAAACCGCTGGCGTAACGAATCCTCTAAAGAGGCTGGTGACTTGTTGTACCACTACCTACAGACCAGACTGTGCCATACCCCGGCCTTCGGCAGTGAACCAAGGAGGATCTCCCGTTCTGCGAAGCTTAGACAACAACTATGCCGTCTCTGCGCTAGGCCTCAGTCCTCAGGCAAGTGTCGGCATCTCTGAGACCAATTGTC?????????????????????????????????????????????????????????????????????????????????????????????????????????????????????????????????????TCTGTTTCCTCTTATGCTGCTAATTCAGACCCCTCACCGGTAACGTTAATAGTTATGGTCCTTAATCGTACATCCTACTCTTATAGTCACAAGCTCCGACCCGAGGATACGAGCCATAGATCAGACGACCGGGGCGATTGCTGGCTGTT?GATCAGCCAAGCCGGAGCATTTCAGTTCTCCTCTTCGATGGTAGGTTCAACCCTACCATGGTAAAGGCCAACACTACGCCCCGGCAGGAAAACATACGGGGTTCTCACGCTCGTATTGACTCTTCCTTCACTCCTAGTTAGACTGTCTGGGGGACGGGGGGCGCCCTCCTCGGTGGGGCTGCGCCCCGACCCGCGGCCGGTCCCTAGGGTCTGGTTAGTCCCGGGTGACCGGCCCGTGATTATCAAAAGCGACGCCTGCATAGTGGTAGGGGCCCCGATCCTATTCTTTCTGAAGGAACGGGGCAAGGGAGGCCGGCCGTTGCGGCGGTGTCGCCCAGAGGACAAATCGAATTTTAGAAACATCTGCCCGTCGTCTACGATGGACGAAGTATTCGAGGACCCCTTCTCAGCTTCCGACAAGCGCTGGCCATAGTGATTAGRASYCCYYCGCYYATTGGTGGGGCTCTAGCATATTCACGGTGTCCGTGTAACAAGGGCGTTTCCCAGTTTCGCCAAGGAATGCATTGTTTCTGGCCCAAGGCTGTCCGTTACCTCCTTCTATTGGGCTCATCCCATGGACAGGGATAGTCCTTTTGATGGTTTGTGATCCTCGTTGGTCCCGCGGGGGCAACCGCCGCGGTGGCGAGGAC???????????????????CAGATCCGCGGGCCAAACACTCCCTCTAGAGGCTAATGAGACAACGAAAGCCAAGCGCCTCACACCAATGCACAACCTTCGAGAGGGCTCGAAACCTAAAAAGAGTGTCGGCTTCAATTTGAAACCAAGCTCTAATAAGCTAAT?TCAAATAAGTGCGACTTGGGCACTATAAACGTCCCTACCTACAAGTCTCAGCCTGTCCGACCAAAACCAGCCGCAAACTATCCTCCTAAAGCCAATGTCAGGAGGGCGGCGCAAGGCAACCTAGTAACTAGCC????ACAAACTAAGCTCATGCGGCGACGACCGCCCCCATTAGGGCGGCGGGAAAGTGTGGGGACGGCCACTCGACGCCGGCGGGCCCGTCCAGTCCTCTTGCCGAGGCGTCACACTCGCTGGGGCGGGCGACCCGTCGGCGGCGGTCCCAGCGTGGAGCAGCGTAACATCTGCCGGCACACGGATTCACAGCGTTCAGAGGGGGATGGGACGCAAAGTCCACCCGAGGATGCTGGGGAGGGCGGCCCGCGCGCGGATC???CCGGGCCT?GGCGGGTGCACTTCCTCGGGGAGCCCACGACGGTTCCGGCGGCGAAAGCCGGGGGAGAGGGCCGCCTCTCCGGAGTTTACAATCCCCTCGGTGGAGGCCCGCCGGTAAACCAGTAAAAGGACCGCGCGGGGGGGGGACAGGTCCTTCGGTACGACGGGCGGACTGCGCATGCGCCGCCCGCGTCGCTTCTGTCTC????AGGGTCGGTGGCGTCGGTCGGCACCCCATCAAGCTAAATTGCGCACGGGTTCTACGAAACCCAGAGCATGAGTGAAAGCTCGGCGGGGGGGAAGCGTCGCCCGCCTTACCGCCCCGCGCCTCTCGACCGCCGTCGGTGAGGAGGATCCCGCACACTTGGGTACCCATTCACAAGCTGGACTAGACGACTTGGTTAACTCCTGCACTCGACGAGTATCGCAACAGCCGAACACGTTAGGCCCGTGCTGAACACCGCATTATCGAGTGCTGCCCTACTCCTACTCCGGGGATCTCATAATGATTTGCGAATACAACGCCGTCCCGTGTCGCTAGGATCGGGTAGGACGTCGGGCATGGGCAGCGGACCCCGACGGGAGTGTCCCGGAATGGGCCTCGCCCAGGCGGGGCGCGGGGTCATACGGATATGTTCAACGTCGACAGGTACCCGTTGTGAAGGTCGAGAAAGACATCCCCCGCC?CGAGATCGTATCGACCGGCGACGGAGAGGTCGCCTCGGGGAGCAGTGTGCAACGAACCGAAGCTCGGCCAGATCCCGCAGTGTGGGCGTACCCTGTCCGTTCGGCGATGGCGTTGTGCCCCACCGCTCCTCGGGCGTGCGCTCTGGCCGGCTAATTCGGGGACGGTGCGAATCCGTCCCGGGCTGGACCATCCATAACAGATTGTCATCGCGAGGCCGTCGTCTGGATCGAGAGCGGTCGGGGGCCGGCCAGAGATGGCGAGGGTTGCGGCCCGATTCGCCTCCGCCGGGATCTGTCCGTGGACCGTTCACTTTGTCCCGGGTCGTCTCGGGCTACGGCCGGGACTAAGGCCGATTCAACGAATGCTACGTCGGCGGTTCGATTGAAAGCCGGATCTAACGCGAGGTCGGCTGTACGCTGGTACGCRAGCAATGGGAGTGGTCGACTCTTCGCWCGCKCTCCGCTTGGAGACGGAGTGCAGGCCCGCGAAAGTATGGWACGCACGCCCCGTCCGGGCGCGTCCCTCCGAAGTTCCAGCGTACAGAGCCCGACACGGGCTGTGCACATCGCTTTTTCGAAGTGCTAACTATTTATTAAGGATTACAGCCCTTGACCGTTGTTCATCCCTCCCGTACAAACATTCTTGCTAGAACAGTTGAATTGGGTAAGGC

**ONUPH**_*Diopatra-Hyalinoecia*

TGCACAAGGCGTCTCTGAACCTCTAAAATTTATAGTAACAGGACACGTCATTCCAGGAAGTGACCAATCTACAATGATCATCATTCTACCTCATAAGTAATAACTGTTGTCAAAGATCTACAGGTGCCCCTAGATTCATAATTAAAATATACCCCCATTCCCTCCCCAATTCCTCCGCAGCTGAGAAAAGGTAAAAGTTTCCATAAGTAATATTGCACTCAACCATCAGATAACATTCCTTTAGATGTGTCACCCTGGTCAATCTACACATGTTGCCCAGGCCTACGGTTTGAACGAGTCCATATGTCATCTGTAAAAACTACCATCCCCCTCATCCTAGCCAAGACAACAACTTACACGTATCCACATCATTTTCAGTAAGGTAACCAACCACCCCACAACTCTATAGAAAACGATAACTTTGAACAATTAGAAACATAGATAACAGCAAACATATCACGCTTAATATGATACACCCATCCAAGCTCCATACTTCATCACAAATACAATGTCATCATTCCTCCATATCGTTCCCCACCAGTTTTTTTTTATCTAAAAGATGCAAACCACATTTCCCACGATCTCGATTCATATAACACTTCCTCACGATGATCAAAAATCTTGCCTTAGTATAACACACTCCAACCCATCAAGC???????????????????CGTCGTCGCGGGAGCTACTTCCTCTGCGCGGCGCCTCCAATACTACGGCATCTTCTATCTTCCGCTTCCTCCTTCGACTCGCTGTGTTCGTGTTCAAGAACCGCCTATCCGCGCTCCCGTGGGCGATGCGAACCGGCAGCATGAGCAAGACAGCGAGAAACTATTCTACGTGAGGGAGCTCCCACGGACACAACTGATTCCGCATTGTGACCAGGCATACCTGGAGCCTTCCACCAATGTCTTGTACAAGGGATGGACAACCGTTGGAGGCCCTCCAGATCGATCCACAAGCTCGTGATAAGCCCGCTCTGAGGCACGTGGATTATGACCCTGGCTATACGGCTTATGGTGAAGCGCCGCTAACACACCTGGGTGGGTCACTGCCTGGAATCTCCCAGGACTAACCGCAACGGTCGTACCGTCTAAACGGCTGGCGCACCGGACTCTCCTTCGAGACCAAGGAATCAAGGTGTCACCGCTTCCAGACCAGGCTACGCAACATCCCTGTCTCCAGCAGGGAACCCTGGAGGATTCCTCGTTCTGCGAAGCTGAGACAACTGCCCACGCGTCCCCGCGCCATGCAACAGTACCCAAACTAGCGTAGTCCTCAACAACACCCCTCCTCG?????????????????????????????????????????????????????????????????????????????????????????????????????????????????????????????????????CTGTTTCCTCTTATGCTGCTAATACAAGCCTGAACATGGTAATGTTAATAGTTATGGTCCTTAATCGTAGGTCCTACATTTATAACCTAAAGCTCCGACCTAACGGGAAGAGCCATAGATCAGACGGCCGGGTCCGTTGTTGGCTTTGAGTTCAGCTCAGCCGGAGTATTTCAATTCTCCTCTTCGTTGGTAAGTGCTATCTTACCATGTTAAAGGCCAAAACTTCGCCCTGGCAGGAAAACATACGGGACTCTTTCGCCCGTATTAACTTTTCCTTTAAGTTTAGTTAGACTGTCTGGGGCTCCGGCGTCGTCCGCCTCGCGGGCACTGGTGCCCGGCCCCCTTCCGGTTTCCTTGGGCTGACTAGTCCCGGGCGGCTGGGACGTAATTACCCAAATCTCGGACTGAATATTGTTAGGCGCCTTGGTTCTGTGTGTTTTTCGGAA?CGAGGTAAGGAAAGACGGTCGTTGCGGCGGTGTCGCCCAGAATACTAATCGAATTTTAAAAACATCTGCACGACGACTGCACTCGCCGAATTGTTCCATGACCCGGCCGTGGCCTCCAATCAGTTTTGGCCATAGTGATTAG?ATCCCCCAGCTTATTGGCAGGGCTCTGGCACATTCGCCGTGTCGGCGTAACAAGTGCGTTTCACAGTTCCGCAGATGAATACGCT???GTTGGCCCAAGGTTGG?CGCTACCTCATCGTTTTGGGTTTATCCCATGTACAGGGATAGTCCTTTTGATGGTTTGTGATCCTCGTTGGTCACGCGGGG??CAACACTGCGGTGGCGAGGACGATCACTTGATCATCA???CAGATCCGCGGGCCAAACATTTYCYCTAGKGRCTWTTGTRRTWACGAGWWTCWWACGCCKTATWAMAATAWAYARYCTTYAGGAGRRCCTRAAYWWWTTAAAAAGTGTCGGCTTTAWTWTMYWWWAAAAATAA??TAAAATTMYYWAWAWTTWGTGCGACAAAGGYAWATAAAWCTTCCTWAAWAWAWWATTCAAATCAGYCTGATCAAAWAWAGCCGCAAWCTWTTCTCCTYAAGCTAWYTAMAGGAGAGAGGCGCARGAAMTCSRAGKAACTATTCCTCCAGAAACTCAGCTGATACGGCCACGGCCACCCCTGCTCGGGCAGCGGGAACGTGGTAGGACAATCTCTGGGG?TGACTTCGAGCGTCCGGTCCTCCTGTCGGGGCTTCATGCTCGTCAGGGCGCTTGTCGTCGCTCCTTGAGTCCTTGCGTAGAGCAGTGTAACATCTGCTGGCACTTCATTTAACATCGTTTAGAGGCGGATGGGACGCCAAGTCGATCCGCGGGTACCGGTGGCTGCGCGGGGTGTCTGGATCTT?CGCGCTGGCGGAGGGTGCACTTCCGCGGAGAGCCCACGACGGTTCTGGCGGCGAAAGCCCGGGAGAACGGCCTTCGGCGGTTGCTTTATAGCTCCCGCGGTCATCGCCCGCTGCCGGACCAGATTTGTGCCCGCCTGGCTCGGGTCGCCCGCTGGGTGTTCGACTGGTGGACTGATCATGCTCCGACTGCGGACGGGATCGGGCACATGAGGTCGGTGGCGTAGGTCGGCACCCTATCGAGCTAAATTGCGCATTGGACCTACGAATCCTAAAGCATGAGTGAAGGCCTGCCTCGCGGGCCCAGTGTTCCCGCGCGTGGGCACACGCCGCTCGTCCGTGGCCGGGGAGGCGGACAACGTACATTTGGGTACCTATTCACAAGCTGGACTAGACAACTTGGTTAAGCCCTGCACTTGTCGAGTATCGTACCTGACGACTACATTAAGCCCGTGTTGATTATCGTGCAATCGAGTGCCGCCCTAATCCTATTACGATGATCTGATAATAATTTGTGAATACAACGCCGATCCGTGTCACTAGGGTCGGATAGGACGTCGGGCATGGGGGTGCCACCTCGACGGGGCGGCTGTGGAGCGGGCCTTGGCTAGCTGGGGCGCACAGTCACACGGATATGTTGAACGTAGATAGAGACTCGTGCTGAGTCGGGGTTTTGTCGAGCCCTAGTCCTATATCGTATCGACCGGCAACGGAGACGGTCCTTCGGGGCCCGGTGCGCAACGAACCAGGGCTCGGCGGGATCCCGGACTCTAGGCGGACTCTGTCGGTTTGCCGATGACGTTGTGCCCCACCGCTCCCCGGGCGTGCACTTCCGTCGGCTAACCTGGGGAGAGTGTGAATTCGTGCCGGACTGGACCATCGATAACAGATCATCATCGAGAGGTTGCCGTCTGGGGTACGAGCGGCCTGGAACTGGCCTGGGCTGGCGAGGCGTGGGCGCCGACTCGCCGTGGTCGGGACCTTCCCGTGGAGTGTTCACTGGCCGCCGTTCTGCGGTTGGCTTCGTCCGGATCCAAAACCGATTTAACAAATGCTGTGTCACTGATGTGATTCAAATCCGGATCTAACGCAAAATTAGCTATCTGCTGACTCTTAGAGAATGAGAGCACAGGCAATTTTTTACGTTTCTTATCTAAAAGCGGAGAGCGGGCAAGCCCTCGCCTGGAACGCCCGAGTCGCTCGGGCGCGTCTCTCTAGAGTGTCAGCGGATAGTGCTTGAAGTAGACACTACACATCACTTTTTCGAAGTATTAACTGTTTATTAAGGATTATAACCCTTGATCGTCGTTAATCCCTCCCGTACATTTATTCTTCCTATAAGAATTGAATTGGGATTGAC

**OPHEL**_*Ophelia-Ophelina*

???????????????????????????????????????????????????????????????????????????????????????????????????????????????????????????????????????????????????????????????????????????????????????????????????????????????????????????????????????????????????????????????????????????????????????????????????????????????????????????????????????????????????????????????????????????????????????????????????????????????GGCGTCGCGGATCGTGATTTTTTGGTTAAATTGGTAAAATAGATAAATGTAATTTTGTCTCGTGCAATTTGAGGCGCCAATATTAGCACTTTATGTCTTTACTTGCAATATGTCAGCTTGCGTTCTTGTTTCTCTTCTCTGGTATTTTTTCAAGAAAATGTTGGTATTGACATTACTCACGATTTCATTACAGCTAGTTCTTCCCGACGATGACTAAAAGCTTCATTTCTATATGTTCATTTACAATTCTTCGAGAAACAACCCCCATGGCAGCACGCAATCACGGGCGCTGAGTCCTTTGCTCCATGCTGGCAGTACTACGCCATCTTCCCTCTTCCGCTCGATCTTGCGACATGCTGTGTGCATGTTCGAGAACCGCCTGTTTGTGTACTCGAGGAAGACATGAGCCGGCTCCATGAAACCAACAGTGAGAATCTTCTTAGCAAAAGGGTCCCAGCACGGACTCAACTGATGCTGCATTGTGCCTCGGGTTTCCGTGCGCCCTCTGACAACCCAATGTTTAAAGGATGGAAGATCGTTAGACGCCCTCAACATCGAGCAGCAAACACCTGACAGGCCCGCTCTCACGTGCATGGATTAGGAAACTARCTATCAGGCCTATGACGACCCGTTGCCCACACACCTGAGTGGGCCTGTTCCCTGAGGAGCTCTGAACTTCCCACAATGTTGGCACCGTCTAAATCTGCTCAGGAGCGAATCCAGCAAGGAAGCTGAGGAGTCAAAGTGGAACCGCCTACAGACCAGGCTATGCCCGGGCCCCTCCCCCAACAGGGAGCGTTGGAAGATCTCTCGTTCTGCGAAAATGGGACAACCCAGCACGGATCCTTGTGCTACGCGAGAGTACACAAACAAGTGTGGGCTTCTCTGACACGCTT?????????????????????????CCTGAGCCAGCTTATGCTCTGCTCCTCCTGCTCTCGGAGCCGCTCCCCGCGCGCTCTGCTGCCGTGCTCGCCCGAGCTCCCCGGGTAGCGGTCCGTTCGCGCCCCCCCCGCT??TGTTTCCTCTTATGCTGCTAATACAAACTTTTACACAGTAATGTTAATAGTTATGGTCCTTAATCGTACA?GTTACTTTTGTAACAACAAGCTCCGACCCTCGGGGAAGAGCCTTAGATCAAACAATCGGTCCCTTTGCTGGCTTTGGGTTCAGCTTAGCCGGAGTATTTCAATTCTCCTCTTCGATGGTACGTGATATCCTACCATGTTAAAGGCCAAAACTACGCCCTGACAGGAAAACATACGGGACTCTTTCGCCCGTATTAACTTTTCCTTTAAGTCTAGTTAGACTGTCTCAGGTGCAGGCGGCGTCCACTTTGCGGG?ACTGCGTCCTGACCTCCTCCCGGTTCCCTTGGGCTGACTAGTCCTGGGTGGCCGGAACGTAATTATCAAAAGGTTGGCCTGAATATTCGTAGGTGCCTCGGTTCTATTTGTTTTTCGGAACTGAGGTAAGGGAAGACGGTCGTTACGGTTTTGTCGCCTAGAATACTAATCGAATTTTAAAAACATGTGCACGACGACTACGATCCCCGGATTGTTTCATGACTCGGCGGCAGCTTCCGACAAGTCTTGGCCATAGTGATTAGAATCCCCCCGCTTATTGGTGGGGCTCTAGCATATTCACCGTGTCGGTGTAACAAGTGCTTTCCACAGTTTCGCCAAGGAATGCGTTGTTGCTGGCCCGAGGTCGTCCGTTACCTCTTTCTATTGGGCTTGTCCCATGGACAGGGATAGTCCTTTTGATGGTTTGTGATCCTCGTTGGTCTCGCGGGGGCAACCTCCGCGATGGCGAGGACGATCACTTGATCATCAAAA???????????????AACACTCCCCTTGGGGGCTGATGCGACACCGAAAGATACACGCCTTTTACCCTTAAACAACCTTCAGGAGGGCCTGAATCTATAAAAGAGTATTGGCTTTACTTTTAAACTATAAAC??CCAAATTAACCTAAAGTTGGTGCGACCGAGGCACACTAAACTTCCTTTTATACCTAAACTAGCTTGTCCGATTAAAAAAAGCCATAAGCTATCCTCCTAGAGCTCCTGATAGGAGGGG??????????????????????????TTTCACAAACTAAGCCTATGCGGAGACGACCGCCCCCGGCGTGTCGGTGGGAACGTGTTAGGCCGGCTATTGTCGTCGGTGCGGAGCGTCCGGTCCTCCTGTCGGGGTTACAGACTCGTCACGGCCCTC?CGCCCGTCGGCATCGTCCTTGCGTGGAGCTGTGTAACATCTGCTGACACTCGGATTAACAGCGTTTAGAGGCGGATGGGACGCAAAGTCGACCCGCGGATACTTGCTCGCGGCTTGGC?????GGACCGTACGGTCGTGCGGGGGGCGCACTTCCGTGGGGAGCCCACGACGGTTCCGGCAGTCAAAGCTTGTGGGAAGAGCTCTCGGGGTGAGTGTTATAGCCCATGACGTGTTGGCCTGCTGGGGGACTAGA??????CCCGCCGGCGTCTGGCCGTGT?CGGTTCGTTCGACTGGGGGACTGTTCATGCCCCGACTGCGG??CAGACCGGGCGCCTAGGGTCTGTGGCGTCGGTCGGCCCCCTATCAAGCTAAATTGCGCATGGGTTCTACGAAACCTAAAGCATGAGTGAAGGCTTGCTGTTCTGGCCTAGTAGCGCCGTTCGCGGCCCTACGCCGCTCGATATCTGTATATGAGGCGGACAACGTACACTTGGGTACCTATATATAAGCTAGTCTAGACAACTTTGTTAACGTCTGCGCTTT?CGAGCATCGTACCTGACGAACACATTGAGTCTGTGTTAATTACCAGGCAATCGAGCGCCGCCCTACTCCTACCACGAGGATCTCATAAGAATTTGTGAATACAACGCCGATCCGTGTCACTAGGGTCGGGTAGGACGTCAGGCATCGGGGCGTCTCCGTGACGGGCGGTCTGCGGAACGGGCCTTGGCCAGCTGGGGCGCGCGGTCATACGGATATGTTGAACGTAGATAGGAACTCGTTCTGAAACGGGGCAATGCCGTGCCCGTAT?CTATATCGTATCGATCGGACACGGAGATGGCCTCTCGAGGCTCAGTGCACAGCGAACTAGAGCTCGGCGGGATCCCGGACTTGAGGCGGACTCTGTCGGCTTGCCGATGACGTTGTTCCCTACCGCCCCCCGGGCGTGCACTCCCGTCGGCTAATCTAAGGACGGTGTGAGTTCGTGCCGAACTGGACCATCGATAACAGATTATCATCGAGAGGCTGTCGTCTGTGACATGAGCGGCCTGGAACTGGCAGGGGCTGGTGAGGCCGCGAGGTCGACTCGCCATCGCCGGGACCTTCCCGTGGACTGCGCACTGGCGGCGGTCG?GTCGTTCGCTTCGGCCGGATTTAAAGCCAATTTAACAAATGCTGCGTAGCTGGTTTGATTAAAATCCGGATCTAACGCAAAATTAGCTATCTGCCGACTTTTAGAGAATGAGAGCAC?GAAAATTTTTTACGTTTCTTATTTATAAGCGGAGAGTGGGCACGCCCTATCCTGGAACGCCCGACTCGGCCGGGCGCGTCCCTCTAAAGTGTCAGCGGATAGAGCTTGAAGTAGACACTACACATCACTTTTTCGAAGTGTTAACTATTTATTAAGGATTATAACCCTTGATCGTTGTTAATCCCTCCCGTACATTTATTCTTGCTATAACAATTGGATTAGGATTGAC

**ORBIN**_*Orbinia-Phylo*

TGAATCATCAGGCTGAGAACCTCTAATCTTCCCAGCAACAGGCCAACTCCCTACTGGAAGAGATCACATTACTATGACCCCCCTCCTACCCTAAGTGTTCTAATCCTTGAGATAACTCAACAGCTGCCCACGGTTTCAAAATTAAATCACTCCCTCCTTCTATTCCCTGTATATCTGCTGCTGTGAAAAATTACATGACCTCATCAGCAATATTGCCTTCCCCCCTCGGACAACATCCCCTCTGAAGCAAGATCCTCATCAATCTATACAGGCCAAACAGGTATAACATTAGACCGAATGCCCGTGTCAGCAGTAAAAAGTACTATCATACCCCTCTCCGTCAAGACTATTACTCACACGTATCAACCTCCTTCTCATCCAAACAGGCATCCACCACACAACTCCATAGAAAACGACTTTCCAGCCTAATTAGCATAATAATCAACAGTAAATACCTCTAAAGTTGTATTCCCCTGCTATAGAAGCTCTATGATCTACATGTACCACTATGTCTTCTTTCATACATATTGTCCCTCTTCAGTACCCTCACTAGAAAAAGACTCTACTCCGTTCAACCACGATCTCAAAACTCGTAATAATACACCCCAATGATCTAAAGCAATGGGATGATGAAATAACTCTCAACTCATTAGCCACTCACACTC?????????????GTCGTGGGAGCTACTTCCTTTGCTCGGTACTGCCGGTACTACGCCATCTTTACTCTTCAGCTAAACTTAGTGACAAGCTGTGTTAGCGCTCCAAAATTGTCTGTTCGTGTACCCGTAAGAGACATGAGCCGGCTCCATGAGACCTACAGCGAGACTCTTCAAGATCAGAGATAGTGCACACGAATTCAACTGACAGTGCTTTGTGACTCGGCATACCTGGAAGCGTCTGCCAATGAGTTGTTCAAGAATTGGACCACCGTTGGAGGCTCTTAACATTGATCCATAAGCTTCTGACAAGCTCGCTCTATTGTACATGGATTAATAGCCTGATTTCTCGACAGATGGTTACCCGTTGCTAATCGACCTGCGTGAGCCCAGACCTGGAAGAGCTATGATCTGCCTGTAATGGTGGCACCGTCTCAACGGCTGGCGCTCGGAACTCTCCAAAGAGTGTGGAAACTCAAAGAGTCACTGCCAACAGACCAGGCTATGCAATGCTCTCGTTTTCAGCAAAGAGTGATGGAGGATCTCTCGTTCTGTGAAGCTGAGATAACCTTATTTGGGTCTTTGTGCCATGAGAGAGTTCACAGGCA?????????????????????????????????TGCGCAAGAGCGCCGATCTGAGCCAGCCAAGGCTAGGATCGTCCTGCTCTCGGAGCAGCGCCAGGCGCGCTCGACTACCGTGCCCGCCCGAGCAACTCTGGCAGCGGACCCATCGTGCCCT???????CTGGTTTCCTCTAATGCTGGCAATGCAAACTTTTACACGGTGACGTTGATAGTCTTTGTCCTTAACGGAA?GTGCTACTCTTGCAGCGAGAAGCTCCGACCTTCGGGGAAGAGCCAAAGATCAGACAACCGAGTTCACTGCTGGCCTCGGGTCCAGCTCAGCCGGGGCATTTCAGTTCTCCTCTTCGATGGTATGCGACCTCTTACCATGGTTAAGGCCAAAACTCCGCCCTGGCAAGGCAACGCCCGGGACTCTTTCGCTCGGGTTAAATCTCACTTTCGGAATAGCTAGACCATCTCGGGTCCAGGCGGCGTCCGCCTCGCGGGGACTGCGTCCTGACCCACTGCCGGTTCCCGGGGGCTGATTAGTTCTGGGTGGCCGGTGCTTAATTACCAAGAGTTGCGCCTGGATACTATAAGGTGCCTCGGTTCTATCTGTTCTCCGGAACCGAGGTAAGGGAGGACGGTCGTTGCGGGCGTGCCCTCCAGAAGACAAATCGAATTTTAAGAACATCTGCACGACGACTACGATGCCGGGCTTG?GTACTGACCTCGCGCCAGCTCCCGACAAGTCTTGGCCATAGTGATTAGAATCCCCCCGCCTATTGGTGGGGTTCTAGCGCATTCGCCGTGTCGGCGCAACAAGTGCTTTCCACGGTTTCGCCAAGGAATGCGTTGCCGTTGGTCCAAGGTCGTTCGCTACCTCCTTGTATTGGGCCTCTCCCATGCACAGGAGTAGCTCTTTTAGCGGTTCGTGACCCTCGCTTGCCCAGCGGCGGCAACGGCTCCTGCGGCGGGGAATGTCGCTGTATTGCTAAAACGGATACGCGGGCCTAACATTTCCTCTGGTGGCTATTGTGACACTGAGAGCTAAGCGCCCTTCACAAATAAACAACCTTCAGGAAGGCCTGAAAACTTTATAGAGTGTCAGCTTTACTAATTCAATCCTAAATATTAAACTAACCTTTAGTTAGTGCGACTGGGGCAAAACTAGCGTCCCCCCTAAAAAGAATATCCCTGTCAAGACCAGAAAAGCTGCAAGCTATTTTCCCTAAGCCAACGAAGGGAAAGTGGCGCAAGACAACCTAGT?ACTAGTCCCCGACAAACTTAGCTCGTGCGGAGACGACCGCCGCTGCCGAGGCAGCCGGAACGTGTTGGGACGACTGTTGCGGGGTTGCTTCGGCGTCCAGTCCTCCTGATGGGGCCTCACGCTCGTTGGGACGCCGGGTTTCCTCGCTCAGGTCCTTGCGCAGAGCAGCGTAATATCTGCTGGCATTCGGATTAACAGCGTCTAGAGGTGGATGGGACGCAATGTCTGCCTGCGGGTACTGGGCGATTGCCTTTTAACCAGGACTCGCGGCACGGTCGGGGGGTGCACTTCCGCAGGGAGCCTGCGACGGTTTCGGAGGTCAAAGCCTGATGGAAGGGCTCCGGTGGCGAGTGTTACAGCCTCC?GATGGTGGGCCCTCTGGAAGATCAGACGCGCGCACGCCCGGCTGGGCTCG?CCGTCGGGGATTCGATCTGCGGACTGCGCAGCCGCTAACTGTCGTTTGGTCCCGACGCTTTGGGTCGGCGGCGTTG?TCGGCACCCCATCAAGCTAAATTGCGCATTGGCTCTACGAAACCCACGGGATGAGTGAAGGCAAGCCTCCGA?GCCGAGTGGTCCCGCCGGAGGGCCCACGCCGCTCGTCCGCCGTCGGTGAGGCGGACAGCGTACACTTGGGTACCTATGCACAGGCTGGCCTTGACAGCTTGGTTAACTCCTGCACTCTACGAGTATCGTATATGCCGAACACATTAAGCCCGTGTTTAATACCGGGGCATCGAGTGCCGCCCTACCCCTATTACGATGATCTCACCATAATTTGTGAATACAACGCCGATCCGTGTCACTAGGGTCGGATAGGGCGCCGGGCACAGGGGTGTCACCCCGGCGGGAGGCTCGTGGAGCGGGCTTCGGCCAGCCGGGGCGCACGATCATACGGATATGTTAAACGCCCATACGGACTTGTTCTGAAGCAAGGCGGTGCTA?ACCGACCAGACGGATCGTATCGACCGGACACGGAGATGACCCTTCGGGGTCGTGCGTACAACGAACCAGAGCCCGGCGGGATCCCGGACTTGAGGCGAACTCTGTCGGCTTGTCGAGGACGTTGCTCCCTACCGCTCCCCGGGCGTGCACTCTCGTCGGCGAATCTGGGGGCAGTGTTATTTCGTGCCGGTCTGGACCATGG?TAATAGACTGTCACTGCGAGGCTGTCGTCTGGGGTGCGAGCGGCCTGGAACCGGCAGGGACTGGCGAGCTCCAAGGGGCGATTCGCCGCCGCCGGGGCCGTCCCGTGGAGTGCTCACTGGCGGCGGTCGTTCCTTTCGCTTCGTCCGGGCTCAAAGCCAATTCATCGAATGCCGTGCAAGTGATGTGATTAAAATCCGGATCTAACGCCAAATCTGCTATCTGTCGACTCTTAGAGAATGAGAGCACAGGCAGTTTTTTACGTTTCTTATTTGGAAGCGGGGCTCAGTCTCGTCCTGGCCTGGAACGCCCGACTCGGCCGGGCGCGCCCCACCGAAGTGTCAACGGATAGCGCTCGAAGCAGGCGCTACACATCGCCTACTCGAAGCGGTAACTGTTTATTAAGGATTATAACCCTAGATCGTCGTTAATCCCTCCCGTGCATTCATTCTTGCTATAACAATTGAATCAGGATTGAA

**OSEDA**_*Osedax*

AGAATTAGCCGTTAGTCAACTAGAAAAACTATTTATTATAGGTCAAAGCTTTTCTAATGACGATCAACTTTGCTAATACCCGGCACTAACTGAAGTATTTTAAACATTGGCACAGTTTAATAGGTGCCCTTAGATTTTTGATTTAGATACTCACTCTGTGTATTCTTAGAATATCAGCTTTTATGGAAAAACAAATGTCTTCATTTCTAACCTAGCCCGCATCCTTCAATCCACTTCCCTTCTTAGGTATCTATTAGTTCTATCTATACCTATAATATAGGATTACGACTTGAACGTGTTCATACGTAAAGAGTAAAACATGCAGTCTCACACATCTATATCTCGAGTATTACTCTCTCGTATCTATCTCACTCCCAGCAATTCTGTTACCACCT???????????????????????????????????????????????????????????????????????????????????????????????????????????????????????????????????????????????????????????????????????????????????????????????????????????????????????????????????????????????????????????????????????????????????????????????????????????????????????????????????????????????????????????????????????????????????????????????????????????????????????????????????????????????????????????????????????????????????????????????????????????????????????????????????????????????????????????????????????????????????????????????????????????????????????????????????????????????????????????????????????????????????????????????????????????????????????????????????????????????????????????????????????????????????????????????????????????????????????????????????????????????????????????????????????????????????????????????????????????????????????????????????????????????????????????????????????????????????????????????????????????????????????????????????????????????????????????????????????????????????????CTTATGCTGCTAATACAAACTTATACAAAGTAACGTTAATGGTCACTATTACTGATCATA?GTCCTACTCTTATAGTTTCTGACTGGAACACTTGGTAAAGGTCCATAGATCAATCGTCATATTATTTTGCTGGCCTTCGGTTCAGCTTTGCCGGAATATTTCAGTTCCCCTCGTCGATGGTAAGTGATATCTTACCATGCTAAGGGCCAAAACTACGTCCTAGTAGGAAAACATGCGGGCTTCTAACGTCCGTATTAATTTTACTTTTAATCTTAGTTAGACTGTCTGGACTTCGAAGGGTGTCCGGCTCACGTGTCTTTCCTTCTGGTCTCCTGCCGGTGTCTGTGAGCTTATCAGTTTAGGATGGCCGGAATGTAATTATCAAAAGGTTCGCTTGTATAATGTCAGGTGTCTCGGTTCTATTTGCTTTTCTGAGCCGAGGTAAGGGAAGTCGGTCGTTACGCGTTTGTCCCCTAGTATAAAAATCGAATTTTAAAAACATGTGCACGATAATCACGATCCATGGTTTGCTACATGACCTGTCGGCAGCCTCCGACAAATTTTGACCATATTGATTAGAATCCCCTCGACGTTTGTCGTAGCTCTGGCACAAATGCGATCTTTGATTGACAAGTGCGTTTTACAGTGCCGCCAAGGATAACGTTGTAATTGGTCCAGGGTTGTCCGTTACGTCTTTCTATTGAGCTCATCTCATGGACAGGGATAGTCCTTTTAATGGTTTGTAGTATTCATCGGTGTCAT?ATGGGAAACCACGTGGA?AAGAGGAACTCTGCTTGGCCATTA???TTAATATGCGGGCCAAACATTTCCTCCAGAGGCTATTGTGACACCGAAATCTAACCGCCAGACTGTAATAAATTATCTTCAAGAGAACTTGAAAAATTTGAAAAGTAATGGCTTTAATTTTATTTTTCTTTGAAATTAATTAAA?AAAATTTTGTGCGACAAAAGACTAAATTACCTCTTTACAACCTAGAATTTCTCTGCTTAATCAACAATAGCCATAAACTATTTTTCTCCAGCTAATTACAGAAAAGAGGCGCAGAGGCCCCTGG???????????????????????????????????????????????????????????????????????????????????????????????????????????????????????????????????????????????????????????????????????????????????????????????????????????????????????????????????????????????????????????????????????????????????????????????????????????????????????????????????????????????????????????????????????????????????????????????????????????????????????????????????????????????????????????????????????????????????????????????????????????????????????????????????????????????????????????????????????????????????????????????????????????????????????????????????????????????????????????????????????????????????????????????????????????????????????????????????????????????????????????????????????????????????????????????????????????????????????????????????????????????????????????????????????????????????????????????????????????????????????????????????????????????????????????????????????????????????????????????????????????????????????????????????????????????????????????????????????????????????????????????????????????????????????????????????????????????????????????????????????????????????????????????????????????????????????????????????????????????????????????????????????????????????????????????????????????????????????????????????????????????????????????????????????????????????????????????????????????????????????????????????????????????????????????????????????????????????????????????????????????????????????????????????????????????????????????????????????????????????????????????????????????????????????????????????????????????????????????????????????????????????????????

**OWENI**_*Myriochele-Owenia*

????????????????????????????????????????GGACGGAAGATTATGGGGGATGATCATATTACTATGTACTTCTTTGTATCTTTTGGATTATAATTTTTGTTATGTGTTAATAGGTGCTCTTGGTTTTATAGTTAGAGTGTGTACGCTGTTGTTGTGCTCTGGTTCAGCAGGTGAGAAGGAAGATAAGTTTATGTTTCTAATGTGGCTTGCGGGGGTCTGTTAGCATTTCTTTGGTGGCGTCTTTTAGAGCGGGTTATACCAGTTATCTAGGTATATTATTTGAACGTCTTCTTGTGTTATCTATTTTTATCGTTATTATACTCTTCTCAGGTGCGAGGATTGTGTGCATRNNTTTANCTGCT???????????????????????????????????????????????????????????????????????????????????????????????????????????????????????????????????????????????????????????????????????????????????????????????????????????????????????????????????????????????????????????????????????????????????????????????????????????GCCCGTCATCACGGGCGCTACTTCCTTCGCTCGGTGCTCCCAATACCACGCTGTTTTCAATCTTCCGCAAATTTTTACTACTTAGCACCCTTGTATTCCAGAATTGCAAGTTCCCCTACCCTTGAGCGACATGAGCCGCCAACATGAGACCAACAGCCAGGCCCTTTGAGACCAGAGATGCTCAGCGCGGACCCAACAGAGACTACATTGTCACTAGGCATATCCCGTCAAGAGTGACAACCACCTGTTCAAGGGATGGGCAACCTGTCGATGCTCTCAACATTGAACCCCAAGCTCCAGACAARCTCCCTCTGCCGTCCGAGGATTAGAAGTCTGGTTATCCCGCTCATGGCCACATGCCAATGTYGAACCTGCGTGAGTTGAAGCTCCACTGAACTATTAACTTACTACAACGGTCGAACCGACTTAATGTGCGGTGCTCCGAATCGACAAAAGAGGCCAGAACTTCGTTGTGYCACCGCCYACAGACAGCAATCCGCATGTGTCCMGATTTTAACACTGAGACCAGAAGGATCTCTCGTTCTGAGGAGCTCGGATGCACAAATTTGCATCTATGTGCCATGCGTCAACCCCCAGGCCAGYGTAGGYT???????????????????CTATTTAAAAAGGGTGTCGTCCTGAGCCAGCCAAAATCAGGAAACTCCTGCTCTCGGTCCCGCTCCAGATGAGCTTACCTGCCGCGCCCGAACGAGCCCCCCTGGTTCAGGTCTGTTCTCGCCCCCCCCACT?????TTCCTCTTATGCTGCTAATACAGACTTTCACACAGTAACGTTGATGGTTATTGTCCTTAATCGTACCAGTTACTTTTATAACAACAAGCTCCGCCCTTACGGGAAGAGCCTTAGAACAAACAATCGGGCCCTTTGATGACTTTGGGTTCAGCTCAGCCGGAGTATTTCAGTTCCCCTCGTCGACGGTCGGTGATATCCTACCGTGATTAAGGTCAAAACTACGCCCTGGCAGGAAAACATACGGGACTCTTTCGCTCGTATTAACTTTTCCTTTAAGTTTACTTAGATTGTCTCAGGTCCAGGCAGCGTACGCCTCGCGGGTACTGCGTCCTGACCTCCAGCCGATTCCGTGGGGCTCATTAGTTCTCGGCGGTCGGAACGTAATTATCAAAAGGAGTGCCTGAATAATGGTAGGTGCCTCGGTTCTATTTGTTTTTCGGAACCGAGGTAAGGGAGGAGGGTCGTGGCGCCTTAGTCGGCCCGGATACTAATCTGATTTTAAGGACATCTGCACGACAACTGCGATCCCGGGATTGCTCAATGACTCTGCGGCAGCCTCCAACAAGTTTTGGCCATAGTGATTAGAATCCCCCCGCCCGTTGGTGGGGCTCTAGCGCACTCGGCCTGCGGTCGTAGCAAGTGCGTTTCACAGTTTCGCCAAGGAATGCGTT??CCTTGGCCCGTGGTCGCTCGCTACCTCCTTATATCGGGCCCATCCCGTGGACGGGGATAGTCCTCTTAACGGTTTGTGATCCTTGTCGGTCCCGCGGGGGCAACCCTCGCGGTGGCGAGGACGATCACTTGATCGTTAAAACGGATACGCAGGCTTGAAATTTCCTTTGAAGGCTATTGTGGATACTTGAAATCTATTCTAATATTCTTTTGGTGCATATAAATAATGATTTAATTTTTTGATAGGTGTCGTCTTTATTGTTTATTTATTAGTTTTTTCAATTATAAATAAGTAGTGCGACTAAGGGTAATGTATCTTCCTTTAAATATGATTTTTTCTTGGCTGATTAAATTAAGTCGCAAAGTATAATTATGAAGTTTTCGATATAATTGTACTGAAAGAAAACTTAACGTTTTTTC????ACAAACTAAGCTCATGCGGAGACGACCGCGCCCGGCCGGTCGGCGCGAACGTGTTAGGACAGCCTCTGGGCGGCGTACGGGT?GTTCGGTCCTTTAGACGGGGTCACACGCATGTCACGACCCCCGTCACCGTCC??TTGGTCCTCGCGTGAAGCAGTGTAACATCTGCGGACACGCGGATTAACATCGCTTAGAGGTGGATGGAACGCAAAGTCTCCGCTCGGGTACTGGATCGGGTGGGGTGCTCGCGGATCTTC??GCCCG??GCCGGACTCACTTCCGAGCGGAGCCCACGACGGTTGTGGAGGTCTAAGCCCCGGGGAAGAGCTTCTTGTTGGAGCCTTATAGACCCGATGGCGTAGGCCTC?TGAACGACCTGATGCACCGGGTATGGCTCGGAGGACAGTGTTATCTCTTCGAGTTTAGGACCGGTCATTCTCGACGAGCAGCAGTCTGTGTTC??CGTCTGTCAGTGGCGT?GGTCGGCCCTCCACCAAGCTAAATTGCACAAGGGTTCTACGAAACCTAAGGCATGAGTGAAGGCTCT????CCGTGCCGAGTGGAGCCGCTCGGTGGCCCACGCCGCCCGATCGCCGTCGATGGGGTGGACAACGTACACTTGGGCGCGTACACATCGGCTAATCTAGACAACTTGCTTGACTCCTGCGCTCTGAGAGTATCATACCTGACGAACTCGTAATGTTCTTGTCGATTATCGAA?CATCGAGTGCAGCCCTACTCCTACCGCGAGGATCTCACAATAGTTTGTGAATACAACGCCGTTCCGTGTCACTAGCGTCGGGCAGGACGTCGGGCATCAGCGCTCCCCCCCGACGGGAGGCATCCGGAACCGGCCTCGACTGGTCGGGGCGCGGGTTCATCCGGAGGCGTTGAACGTAGATAGCAACGCGTTCGGAAGCGGGGCTCTAGCGCGCCCGCCATTCATATCGTATCGACCGGACACGGAGACGGC?CCCTGGGGTCCAGTGCACAACGAACCCGAGCCCGGCGGAATCCCGGAGTTGAGGCGGACTCTGTCGGTTTGCCGATGACGTCGTGCCCTACCAGCCTCCGGGCGTGCACTTCCGTCGGCG???CCGAGGACGGCGTGA?TTCGTGCCGGACTGTACTGCCGATAACAGATTATCATCGAGAGGCTATCGACTGGGATGCGAGTGGCTTGGAACTAGCCGG?CCTGCTGAGACCGCGAGGTCGAGTCGCTGTCGCTGGGTCCTTCCCATGGAATGTTCGCTAGCAGCA?TCG?????TGCTTAGCGACCGGAATCGAAGTCAATTTAACAAATGCTGTGTCGCCGATGTGATTAAAAAGCGGATCTAAATCAGAATTAGCTATCTGCTGACTCTTAGGGGATGAGAGCACGGGCAGTTTCCCATGTTCCTTATCGGTATGCGGGAAGCGGTTGCGCCCTCCTCTAGAACGCCCTTCTCG?TCCGGCGTGCCCTTCCCGACAGTCTGCGGATAGAGCTTGAAGTTGGCACTACACATCACTTTTTTGAAGTGTTAACTATGTATGCTGGATTATAACCTCTGATCGTCGCT??????????????????????????????????????????????????

**PARAL**_*Paralacydonia*

??????????????????????????????????????????????????????????????????????????????????????????????????????????????????????????????????????????????????????????????????????????????????????????????????????????????????????????????????????????????????????????????????????????????????????????????????????????????????????????????????????????????????????????????????????????????????????????????????????????????????????????????????????????????????????????????????????????????????????????????????????????????????????????????????????????????????????????????????????????????????????????????????????????????????????????????????????????????????????????????????????????????????????????????????????ATCATGGGAACTACTTCCCTTGCTCGATACTCCTAATACTACGCAGTCCCCCATCTTCTGCATCTTTTCTCTATTTGCCGCCAATATGGTCAAGAACTATCTGTCCGCCCACATGTCGGCTACATGCGCCGGCAGCGCGGC????ACAAACAGGACCATCGACACTGTAGACGGCGCATACGGGGCCAATAAAGACCAGCAGGTAACTCTTGAGTTCTGGAACCCAGCTCAAATGTCGTGTTCAAGGGGTGGATTAACGTTGGAAGCCCCCTCCATCACAGAGATCGCGACTGACAAACTCTCTCTCACGCCCATGGCTTAGGCGCCATGCAAAACTACACGTTAAGACCTGGACCTATGACACCTGGGTGAACCGAATCCCCAAAGTGCCTTACTCTCCTCGCAACACGAGCGCGAGCTTAACAGCCGGAACTCAGCAGTCACAAAACCAGCGGCCAACTCACAGCATTATAGCCTTGAGACCGTGCGCCAGTTACCCTCAGCTTCCAATTCTGAATGAAAGAAGATCTAGCGAAGTGTAAAGATGAGACAACCGAGAAGGGGAGTTCGCGCTACATAAGCATCCATCCGCT?????????????????????????????????????????????????????????????????????????????????????????????????????????????????????????????????????????????????????????????????????GGATCCCTTATGCTGCTAATACAAACTTTTACACAGTAACGTTAATAGTTATGGTCCTTAATCGTACTTCCTACTTTTGTAACAACAAGCTCCGACCTTCGGGGAAGAGCCTTAGATCAGACAATCGGGTCCTTTGCTGGCTTCGGGTTCAGCTCAGCCGGAGTATTTCAATTCTCCTCTTCGATGGTACGTGATATCCTACCATGTTTAAGGCCAAAACTACGCCCTGGCAGGAAAACATACGGGACTCTTTCGCCCGTATTAACTTTTCCTTTAAGTTTAGTTAGACTGTCTCGGGTCCCGGCTGCGTGCACC?AATGGGTACTGCGTCCGGACCTCCTCCCGGTTCCCTTGGGCGGACTCGTCCTGGGTGGCCGGAACGTAATTATCAAAAGGCTTGCCTGAATAATGGTAGGTGCCTCGGTTCTATTTGTTTTTCGGAACTGAGGTAAGGGAAGACGGTCGTTACGGTTTTGTCGCCTAGAATACTAATCGAATTTTAAAAACATCTGCACGACGACTACGATCCCCGGATTGTTTCATGACCCGGCGGCAGCTTCCGACAAGTGATGGCCATAGTGATTAGAATCCCCCCGCCTATTGGTGGGGCTCTAGCATATTCACCGTGTCGGTGTAACAAGTGCGTTTCACAGTTTCGCCAAGGAATGCGTTGTCTCTAGTCCGAGGCTGTCCGTTACCTCCTTCTATTGGGCTTATCCCATGGACAGGGATAGTCCTTTTAATGGTTTGTGATCCTCGTTGGCCCCTCGGGGGCAACCTCCGCGGTGGCGAGGACGATCACTTGATCATTA??????????????????AACATTTCCTTTGAAGGCTGATGTGATAACAAAAATATATCGCTATTAATTATTAAATAATTTTTAGGAGAGCCTAAAAAAGCTAAAAAATATTGGCTTTATTTAAATTTAAAAGTTAGT?????TTATAAAAAATTGGTGCGACATAGGTAAATAAATCTTCCTAAA?TCATATAATAAAT?AATCTAAGAAAAATTAGTCATAAGCTATTTTTATTAAGTTATTGACATAAAAGA??????????????????????????CTCCACAAACTAAGCCTATGCGGAGACGACCGCTCCTGCCTGGGTAGCGAGAACGTGTTAGGACAGCCTCTGGGGTGACGATCAGACTTCCGGTCCTCCTGTCGGGGCTTCATACTCGTCACGTCGTCTGTCGTCCTCCCTTGAGTCCTTGCGTGGAGCAGTGTAACATCTGCTGACACTCGGATTAACACCGTTTAGAGGCGGATGGGACGCAAAGTCGGCCCGCGGATACCGGTGGG???GCGGGATA?CCGGATCCGTCGTTTCGGCGACGGGCGCACTTCTGCGGGGAGCCAACGACGGTTTCGGCAGTCAAAGCCCGGTGGAAGAGCTTTCGGGGTGAGTGTTATAGACTCCGTGGCATTGGCCTGCTGGGAGACCAGACTCGTGCCCGCCCGGCTCGGCCCCTCCTCCGTGCGTTCGACTGGGGGACTGTACATGCTCCGACTGCGGAAGGCG?GGGTCACCCAGGGTCAGTTGCGTCGGTCGGCACCCTATCAAGCTAAATTGCGCATGGGTTCTACGAAACCTAAAGCATGAGTGAAGGCCCGCTTCTCGAGCCTAGTAGTCTCGCCTGCGAGCCTACGCCGCTCGTGCTCCGCAGCAGAGGCGGACAACGTACACTTGGGTACCTATATACAAGCTGGTCTAGACAACTTGGTTAACTCCTGCACTCTTCGAGTATCGTATATGTCGCACACATTAAGTCCGTGTCGATTACCGGACATTCGAGTGCTGCCCTACTCCTATTACGATGATCTCATAATAATTTGTGAATACAACGCCGATCCGTGTCACTAGGGTCGGATAGGACGTCTGGCATCGGGGTGGCTCTTAGACGGGAGGCCTGTGGAACAGGCCTTGGCTAGCTGGGGCGCACAGTCATACGGATGCGTTGAACGTAGATAGAAACTCGTTCCGAATCGGGGCAATGCCGTGCCCGTAACCTATATCGTATCGACTGGCCACGGAGATGGCCCCTCGGGGTCCAGTGCACAACGAACCAGAGCTCGGCGGGACCCCGGACTTGAGGCACACTCTGTCGGCTTGCCGATGACGTTGTTTCCTACCGCTCCCCGGGCATGCACTCCCGTCGGCTAATCTGGGGACAGTGTGATTTCGTGCCAGACTGGACCGC?GTCAACAGATTATCATCGAGAGGCTGCCGTCTGGGGTAAGAGCGGCCTGGAACTGGCAGGGGCTGGCGAGGCCGCAAGGTCGATTCGCCGTCGCCGGAACCTTCCCGTGGACTGCTCACTGGCGGTGGTGG??TCGTTTGCTTCGGCCGGATCTAAAGCCGATTTAACAAATGCTGCGTAGCTGGTGTGATTAAAATCCGGATCTAACGCAGAATTAGCTATCTGCCGACTTTTAGAGAATGAGAGCACCGGCAATTTTTTACGTTTCTTATTTATAAGCGGAAAGCGGGCAAGCCCTCAACTGGAACGCCCGACTCGGCCGGGCGCGTCCCTCTAAAGTGTCAGCGGATAGAGCTTGAAGCAGACGCTACACATCACTTTTTCGAAGTGTTAACTATTTATTAAGGATTATAACCTTTGATCGTCGMTAATCCCTCCCGT??????????????????????????????????????

**PARAO**_*Aricidea-Cirrophorus-Paraonis*

??????????????????????????????????????????????????????????????????????????????????????????????????????????????????????????????????????????????????????????????????????????????????????????????????????????????????????????????????????????????????????????????????????????????????????????????????????????????????????????????????????????????????????????????????????????????????????????????????????????????????????????????????????????????????????????????????????????????????????????????????????????????????????????????????????????????????????????????????????????????????????????????????????????????????????????????????????????????????????????????????????????????????????????????????????GTTACGGGGACTACTTCCTTTGCTTGATGCTACCAGCTGTACACTATACTTTCTCTTCCGCCCAATTTTGTGGTTTGCTGTGTTAGCGTCTTAGAATCGTCTGTCCGTGTACCCTTCGGAGACGGGAGCCGGCACTATGAAACCCATAGCCAGAGTCTTTGACACAAGAGAGAGTGCCCACGGACATAATAGATACTGCCTCTTATCCCGGCACACTAAGAGAAATCTGAGAATGGGTTGTACAATGGCTGGTTGACAGTTTGAAGCCTGTGCGATTGATGCACAGTCCCCAGACAAGCACCCTCTAATGTGCGTGGACTAAGCTTCTGGTCATCACGCTCATGAAGACTTGCAGCCGCCATACAAGGGTGCGTTCAAGCCAGGACGAACTCAATTCTGTCTGAAACGGCGGCACCGTCTCGACTTGTGGAGCTCCGAATTCTCTAAGGAGACAGACAGCTCAAGGTGGCACTGTCTACAGACCAGGCTCCGCGTGCGCCTTTCCCGCAGCAGGGAGCGCTCGAGGTGCTCTCGTTCTGTAACCCTGGGAGAACTCCCCATGGATCCTTGTGCCACGCAACAGTGCGCAGGCT??????????????????????????????????????????????????????????????????????????????????????????????????????????????????????????????????????????????????????????????????CTGTTTCCTCTTATGCTGCTAATACAATCTTTTACCCAGAAATGTTAATAGTTATGGTCCTTAATCGTACATCCTACTTCTATAACAACAAGCTCCGACCCTCGGGGAAGAGCCTTAGATCAAACAATCGGGTCCTTTGCTGGCTTTGGGTTCAGCGTAGCCGGAGTATTTCAATTCTCCTCTTCGATGGTACGTGATATCCTACCATGTTAAAGGCCAAAACTACGCCCTGACAGGAAAACATACGGGACTCTTTCGCCCGTATTAACTTTTCCTTTAAGCATAGTTAGACTGTCTCGGGTCCAGGCCGCGTCCGCCTCGCGGGCACTGCGTCCTGACCTCCTCCCGGTTCCCCCGGGCTGACTAGTCCTGGGTGGCCGGAACGTAATTATCAAAAGACGTGCCTGAATAATGGTAGGTGCCTCGGTTCTATTTGTTTTTCGGAACCGAGGTAAGGGAAGACGGTCGTTACGGTTTTGTCGCCTAGAATACTAATCGAATTTTAAAAACATGTGCACGACGACTACGATCCCCGGATTGTTTTATGACTCGGCGGCAGCTTCCGACAAGTCTTGGCCATAGTGATTAG?ATCCCCCCGCCTATCGGTGGGGCTCTGGCATATTCGCCGTGTCGGCGCAACAAGTGCGTTTCACGGTTTCGCCAAGGAATGCGTTGCTGCTGGCCCTGGGTCGTCCGCTACCTCCTTCTATTGGGCCTCTCCCATCGACAGGGATAGTCCTTTTGATGGTTTGTGATCCTTGTGGGCCCCGCGGGGCCCCCGCCTGCGGTGGCGAGGACGCTCAC?TGATCATCA???CTGATTCGCGGGCCTAACATTTCCTTTGGTGGCTAATGTGACACCGAAACTCTACCGCCAATTTCCTATAAATAGCCCCCAAGAGGACTTGGAAGTATTAAAAAGTGAAGGCCTTATCACCACTTAATCAGCAATTAAACCTACGCGTGATTAGTAGAACTAAGGCATCTAAAGATTCCTCAACACCCAGCACAAGACTGCCTGATCATACCTGGCCTCAAACTATTTCCTTCGAGCTGATGACAAGGAAGTAGCGCAGGGTACCCTAGTAACT????CTCCACAAACTCAGCCCATGCGGAGACGGCCGCCCCCGCCCGGGCGGCGGGAACGCGTTGGGAGAGCCTCTGCGGCGACC?TCGGGGGTCCGGTCCTCCTGTCGGGGTTGCACACTCGTCACGCCCCCGTC?GTCGTCGCTTGACTCCTCGCGTGGAGCAGTGTAACATCTGCGGACACTCGGATTAACAGCGTTTAGAGGCGGATGGGACGCAAAGTCGACCCGCGGATACTGCGTCGGCCGGGGGAAGGGCGGATCCTTCCCTCGGCCGGCGATCGCACTTCCGCGGGGAGCCCGCGACGGTTCCGGCCGTCGAAGCCCGGGGGGAGGGCCCAAGGCCGGGGTGTTAAACCCCCG??AGGGCGGGCCGGCCGTCGGACCAGACT???TCCCGCCGTCGGGCTCCCGCCCTCCGCCCGTTCGACCTGAGGACTGTACATGCTCTGACCGCGGGAGTGACCGGGCGCTCAGGGTCTGCGGCGTCGGTCG????CCCATMAAGCTAAATTGCGCACGGGCCGTACGAAACCTAAAGCATGAGTGAAGGCGCTCCTCTCGCGCTCGGCGGTCCCGCCCGCGGGCCCGCGCCGCTCGACCTCCGTCGGCGAGGCGGACGACGTACACTTGGGTACCTATATACAAGCTGGTCTAGACGACTTGGTCAACTCCTGCACTTTTCGAGTATCGTACCTGCCGAACACATTAAGTCCGTGTCGATTACCGGGCAATCGAGTGCTGCCATGCTCCTACCATGAGGATCTCATAATAATTTGTGAATACAACGCCGATCCGTGTCAGTAGGGTCGGGTAGAACCTCGGGCATCGGGTGC?CCCCGCGAGGGGAGGTCCGCGGAACGGGCCTCGGCCAGCTGGGGCGCGCGGTCATACGGATATGTTGAACGTTGATAGAAACTCGTTCCGAAACGGGGCGATGACGAGCCCGTAT?CTATATCGTATCGACTGGACACGGAGATGGCCTTTCGGGGTCCGGTGCGCAACCAACCGGAGCTCGGCGGGACCCCGGACTTGAGGCGGACTCTGTCGGCTTGCCGATGACGTTGTTCCCTACCGCCCCCCGGGCGTGCGCTCCCGTCGACTAATCTGGGGACGGTGTGATTTCGTGCCAGACTGGACCAT?GATAACAGATTATCATCGAGAGGCTGTCGTCTGGGGTACGAGCGGCCTGGAACCGGCAAGGGCTGGTGAGGCC????GGTCGACTYGCCATTGCCGGGACCTGCCTGTGGACTGCTCACTGGCGGCGGTCG?GCCGTCCGCTTCGGCCGGATTCAAAGCCGAGTTAACGAATGCTGCGGAGCCGGTGTGATTAAAATCCGGATCTAACGCAGAATCAGCTATCTGCCGACTTTTAGAGAATGAGAGCACGGGCAATTTTTTACGTTTCTTATTTATGAGCGGAGAGCGGGCAAGCCCTTTCCTGGAACGGCCGACTCG?CCGGCCGCGTCCCTCTAAAGTGTCAGCGGATAGAGCTTGAAGCAGGCGCTATATATCACTTTTTTGAAGTGTTAACTATTTATTAAGGATTATAACCTCTGATCGTCGTCAATCCCTCCCGTACATTTATTCTTGCTATAACAATTGAATTAGGATCGGC

**PARER**_*Stygocapitella*

???????????????????????????????????????????????????????????????????????????????????????????????????????????????????????????????????????????????????????????????????????????????????????????????????????????????????????????????????????????????????????????????????????????????????????????????????????????????????????????????????????????????????????????????????????????????????????????????????????????????GGCGTTGCGGTAAATGACAATCTAGATAAATTCGGTTAATAGTCCACCAAATGAGTAGCAAACTTAATTAGAAACACCATTACCAGCCAAATATTTTATTATAAATAAAACATCTGCATTCATTATCATTTCCCTTTACTCGTACCTCTTCAAGAAAAAATTGCAATCCAGATTAATTTTGATCATGCATCGAATAACTTTAATCCAAAATGAACTCAAGCCTTGGATTGATATAATTAATCTCAATTCTATAGCTAGCAACCCCC?????????????????????????????????????????????????????????????????????????????????????????????????????????????????????????????????????????????????????????????????????????????????????????????????????????????????????????????????????????????????????????????????????????????????????????????????????????????????????????????????????????????????????????????????????????????????????????????????????????????????????????????????????????????????????????????????????????????????????????????????????????????????????????????????????????????????????????????????????????????????????????????????????????????????????????????????????????????????????????????????????????????????????????????????????????????????????????????????????????????????????????????????????????????????????????????????????????CTTATGCTGCTAATACGAACTTTAACAAGGTGACGTTAATAGACATGGTCATTAATCGAAAAATCCACTCTTGTAGCACTAAGCTCCGACCCTCGGGGAAGAGCCGTAGATCAAACAATGGGTCCCTTTGCTGGCTTTTCGTTTAGCTTTGCCGGAGTATTTCAATTCTCCTTGTCGATGGTATCTGATATGTTACCATATTG?GGGCCAAAATTACGCCCNGGAA?GAAAACACCCGAGACTCTTTCGCTCGGGTTAACCTTTCCTTTAAGTATAGTTAGACTGTCTCGGGCTCAGGCGGCGTTCGCTCGG?GGANACTGTGTCCTGACCTCCTGTCGGTTAGCGCGGGCTAAGTAGTTCGGCTTGGCCGGTACGTAATTGCTAAAAGCTGTGCCTAGACAGTGCAAGGTGCCTCGGTTCTATTTGTTTTTCGGAACCGAGGTAAGGGAAGACGGTCGTTGCGGTTTTGTCGCCCAGAAAACTAATCGAATTTTAAGAACATCTGCACGACAACTGAAATCTCCGGATTGCTACATGACTCGGCGGCAGC?CCCGACAAGTCTTGGCCATAGTGATTAGAATCCCCCCGCTTATTGGTAGGGCTCTAGCATATCAGCCCTGCGGGCGCGACAAGTGCGCTTCACGGTTCCGCCAAGGAATGCATTGTAACTGGTCTGAGGTTGTCCTTTATCTCCTTCTATTGGGCTTGTCCCATGGACAGAGATAGTCTTTTTAACGGTTTGTAGTCCTCGTTGGTCGCTGGGGGGCAACCGTCCCGGTGGCGAGGACGACTACTTGATCGTTAA????????????????????????????????????????????????????????????????????????????????????????????????????????????????????????????????????????????????????????????????????????????????????????????????????????????????????????????????????????????????????????????????????????????????????????????????????????????????????????????????????????????????????????????????????????????????????????????????????????????????????????????????????????????????????????????????????????????????TTAACAGCGCTTAGAGGCGGATGGGACGCAATGTCCGGCTG?GGATGCCCGAGGGGGCCCCGTCGATCTGTATCGCCCGTTGTGGCGGTGATTTCCCGTCCCG???GAGCCCGCGACGGTTTCTGGGGCCTAAGCCGGCGGGAAGTGCTCGGCGGCTAGCGCTTATAGACCGCTTGGCGGCGGTGCCCCGGCGGACCAGGAACGTGCCCGGCCGGGAGGGGCAGCGCGAAACCGACGGGAATGGCGGACTGGGCATGCGCCGAGTAGCCTGGCGGCTCGTCACTAAGGGTCCGCGGCGCCTGTCGGCACCCTATCAAGCTAAATTGCGCAATGGCAAGTTTGTTGCGAGAGTATGGGTAAGGGGCGGCATTTGGCCCCGAGTGGTCCCCGATGGGGGCCCACGCCGCTCGACTTGCGTCGGTGAGGCGGACTACGTACACTTGGGTACCTATACACAAATTGGTCTAGACAACTTGGTTAACTCTTGCGCTCAATAAGTATCGTACCTGCCGAACACATTAAGCCCGTGTCGGTTATCGGGCAGTCGAGTGCTGTCCTACTGCTACAACGATGATCTGATAATAATTTGTGAATACAACGCCGATCCGTGTCACCAGGGTCGGGTAGCACGTTGGCAGCAGCGATGCTTCGGCAACGAGAGGCCCGTGGAGTAGGCTGCGGCCGGCCGGGGCGCACGGTCATACGGATACATTAAACGCAGATCGAAGCTCGTTCGGAAGGGAGGCAAAGATTAGCCTGCGGTTGATATCGTATCGACTGGACACGGGGATATC?CTTCGGGGTCGAGTGTACAACGAACCAGAGCGTGGCGAAATCCCGGACTTGAGGGCGTCTCTGTCGGCTTGCCGATGATTTTGCGCCCTACCGCCCCCCGGGCGTGCACTTTCGTTGCCTAATCTGGGGACAGTGTTAT????????????????????????????????????????????????????????????????????????????????????????????????????????????????????????????????????????????????????????????????????????????????????????????????????????????????????????????????????????????????????????????????????????????????????????????????????????????????????????????????????????????????????????????????????????????????????????????????????????????????????????????????????????????????????????????????????????????????????????????????????????????????????

**PECTI**_*Pectinaria*

TGGCTTCATGTTTTGTGTGGAGCTAGTATGGTATGCCGCCATGGGCAATGCAATCGTAGATCCATGTTTCTCAAGAGGTATCGCGGTGACCGTTGGGCCGGGGTGNCTCACGAGACGTGGAAGGCGCGCTCGGTATGGCAGTTAGACTGATTGCATTGGTTACTATTGTGTCAACCTTTTTTAGAGTGGTTCTCGTAGTGGCTGC?????????TTGGGCAGGACGCTTTATTTACTTGGTCGCCGGTGTCTTCAGGGGCCACTTATGCATATATTTCGGGCATGACCTTCATGCGCCTGCGCTTGTCGACTTGGATTGCGGCGTTTGCGAAGGTCGACGATGCGTGGGCTGTGTACATAATTTTGCTAGCCTTTGTGCATTCTAGGTGTTCGTTACA???????????????????????????????????????????????????????????????????????????????????????????????????????????????????????????????????????????????????????????????????????????????????????????????????????????????????????????????????????????????????????????????????????????????????????GATCGCGGGTGCTGAGTCCCTCGCTCGGCGCCCCCAGTACTACGGCATCTTCTCTCTTCCGCAAACTCTCACGACTTGCTGTACCAGCGTTCCAGAACCGCCTGTTCGCCCTCCCTAGAGCGACGTGTGYCGGCTCCACGAGCCCAACAGCGAGAGTCCTCAAGACCAGAGGGTCCTCRCACGGACTCAACCGACTGTCGCTCGTGCCTTGGCATACCATGAGAAGTCCTCCAATGAGCTGCATAAGGGATGGTCAACCGCTGGAGGCTCTCAACATTGACCCCCAAGCCACCGACAAGCCCGCTCTCCCGCCCGTGGATTAAGCGCCTTGCTATCACGCCTATGGGGGTCCGCATGCAACGGACCTGGGTGGGCCACCGCCCCCTTGAGCTGGATTCTTCCCGGAACGGTGGCACCGTCTCAACTGCCGGAGCAGCGAACTCCCCAAGGAGGCTGGCACGTCACCGTGCCACCGCCTACAGACTGTGCTGTGCCCGCCCCCTGCCTTCAGCGCCGTGCGTGCAAGGATCCCTCGTTCCGAGGAGCTTGGACAACTCAGCATGCGTCTATGTGCCATGCGACCGTCCCTCCGCCAGCGTGGTCTTCTCCGACATCCTCCTGTCCTGAAAAGTTCTGGCGCCGATAGAAGCAATCTTTGGCTCTGGCTCCGCTGCTCTCGATCCGGCGCCAGACGAGCTCTCAGACAGCGATCGAGCGAGTATTATGGACAGTGGCCCCGTCCTGCCCTCTCCGCA??TGTTTCCTCTTATGCTGCTAATACA?GCTCTCGTATAGTAACGTTAATAGTTACTATTCTTAATCTTACTTGTTACTTTTGTAACACTCAACCCTGACTTC??GGAAAGGGTCTCAGACTAAACAATCAGTCTGTTTGCTGGAGTG??GGTCAGCTCCGCGGGAGTATTTCAATTCTCCTCGTCGACGGTAGTGTATTGACTACTGTGTCAAAGGTCAACACTACGCCCTAGCAAAGAAACATACGGGACTCTTTCGCCCGTATAAAGTGTGCACTTAAGTTTAGTTAGACTGTCTCGGGAGCGGGCGGCGTCGGGCGTTCAGCTACTTC??CCCATCCTCTGTCCGGTGTCCCCTGGCTAACCAGTTAGGAGCGTCTGGAGCGTAATTACCAAAAAGAGTGTCTGAATAGCCGTAGGTGCCTCGGTTCTATTTGTTTTTCGGAAGCGAGGTAAGGGAAGACGGTCGCTGGGCCCCTTCTGGCCAGAAAACTAATCGAATTTTAAAAACAGGTGTGCCACAACTGCGATCCCAGGTTTT?CTCATGACCCTGCGGCAGCATCAGACAAGTTTTGGCCATAGTGATTAGGCTCCCCCCGCATTTTGGTTGGGCTCAATCGCCTGCGCCGC?CAGGCGAGACAGATTTATATGATAACTTCGCCAATGGATACGTC?CTTCAAGGCTAAAGCGATTCGTTACCCCATTACGTAGAGCCTATCTCTTGGACAGGGATCGTCCTTTCGATTGTCCGTATTGGTAGGAGTTGAAGCGG??GCTTCGGT??CGGCTATTCGTTCCATTGCTGGACTCTCA???CGGATTCGCGGGCCTGGAATTCCCCTTGTGGGCTTTTGTGATAACGAGGGCCAAGCGCCTAGTAAAACTAAATGGCCTTTAGGAAGACCTAAAAAAATCAAAGAGCGTAGGCTCTGTTCTCATATAGGGGGAAATTATCTTTTTTGAGAACCAGCGCGGCTGAGGAATTAA?ATCTTCCATTTAAATCAGTTCAAGCCTGCCGGATCAAAAATAGCCTCGAATTATCCTTCTTGAGTTCCTGACAGAAGGGCGACGCAGGATATCCTGGTAACCAATCCYTAACAAAATCATCTCACGCGGAAACGCCAACTGCAGGTTGGCCTGTCAGAGAGTCTTGCGCCGGGGTATGCCAGCGGTGCGGACCTTCTCAGTTTCTTGAAGGGGCT?CAGAACTGTGAGTGGGGGGT??TCGTCCGTTTTCGGCCTTACGTGGAGCAGTCTTGCATCTGCCAGCACTCGAATTATAAGCTGTAAGAAGCGGATGAGGC?ACATTAGGCGCCACGGTTGGT?CGGGGCTTGGCGCGGTCTTGCATCGT???????GTCTGCGGCTGCACTGCCGTGGCGCGTGGG?GACGGTTGTGTCGACGAAAGCTCAGGTGTAGAGCTCTCGGATGTAGTATTATAGGCCGC???GAGTGGGTGTCATGCCCGACCAG??????????????????????????????????????????????????????????????????????????????????ACGGAAGGTCTC?????TTGTCTGGTATCTCTACCAGCTAAATTACGCGAAGGTGG???TAAACCCCACGCACGAGTAAAGGCGGCCTCGCGGAGGTGGGTCTTCCCT??CGGGAGCCCACGCCAACCGTTCCCGGGGGTTGGTGTGGACAACGTCCACTTGGGTACCTGTGTACAAGCTGGCTTAGGCAACCTGGTTAACTCCTGCGCTTTTCAAGTATTGTAAATGACTAGTTCATTAACT?CGTGTTGACTACCCGAGATTCGAACGCTGTCCTACTGCTACGGCGAGAATCTGAAAACAATTTGTGAATACAACGCTGACCCGTGTCACCAGGTTTGGGTAGCACGTCGGGCATC???AAAACTCCCCGACGGGAGGTGCGCGGAGACGGCTTTCGCCAGCGAGGGCGCGCGGTCATATGGCTATGTTGAAAATAGATAGAAACTTGTTCCGAA??GGGGCAATCGC??GCCCCGT??CTATATCACATTGACTGAACCCGGGCTA?????????????????GGCACAACGAACCAGATCCCTGCAGGATTCTGGAAT?GTAACGGACACTGTGGTCTTACACATGTACCCTTGCTCCACCACGCTTTGGGCG?ACGCTCCTGAAGGCTACTCTGGGGAGTTAATGGCTGCGGGTCAGGCTGGACCATCGGTAACAGATTATCACCGAGAGGCTGCCTTACTGAACCGGGACTGCCCC?GGTCGCCCAGGGCTGGCGAGACCGGTTGGTCGACCTGCTCTTCTAGGGTGGTTGGTCAAGAA?GCGCTCGCGTCGGGGTCA???GCGTGGCTTCGACAGGATTCAAAGTCAGTTCAACAAATGCTGCGCTGTTGGTGTGATTAAAACCCGGATCTAACGCAGAATCAGCTATCTGCTGACTCTTGAAAAACGAGAGAGCTGGACGGTTCTTGCGTTTTTTATTTGACGAGTTGGCGCACTACC?TGCATGTCTTGAACGTTGACTTCG??TCGGCGCGAACAGTTAAACTGTCAGTTGATAGAGCTTGAAGTAGGCACTACGCATCGCCTTTTCGAAGCGTTAACTATTTACTAAGGATTCTAAATTTTGATCGTCGTTTACCCCTCCCGTACATTCATCCTTGCTAAAACAATCGAATTAAGATTGAC

**PHOLO**_*Pholoe*

TGAACCATCTGCTATAGCACATCTAAGATGCTTTGCTGTAGGCCATATCTTTATAGGCAGAGACCGCACCACAATGAGCATCTTTCGATCCTGCATATTCTTGTAATTGTCACTACTCCACTGGTGCTCTTGGATTCTCCACCAAAATATACACTCATATAATCCTTACAAGATCAAGTGCAGTGAAAAGGAAAAAGACCTCAGAGCAAATATCGCCTGCTGCCCTCAGTCCTCTTTTCGTATGATGTTTCAACTAGCGCATACCACACCAGTTCTTCAGGGCTACGCTTAGAACGAGTGCTTATGTAGGCTGCCAAAGATGCTACCACACTTAAGACAGATATGTCAATCGTACACATGTACTAACTGCTTTTTCTGCATGGCA???????????????????????????????????????????????????????????????????????????????????????????????????????????????????????????????????????????????????????????????????????????????????????????????????????????????????????????????????????????????????????????????????????????????????????????????????????????????????????????????????????????????????????????????????????????????????????????????????????????????????????????????????????????????????????????????????????????????????????????????????????????????????????????????????????????????????????????????????????????????????????????????????????????????????????????????????????????????????????????????????????????????????????????????????????????????????????????????????????????????????????????????????????????????????????????????????????????????????????????????????????????????????????????????????????????????????????????????????????????????????????????????????????????????????????????????????????????????????????????????????????????????????????????????????????????????????????????????????????????????????????????TTTCCTCTTATGCTGGTAATACAGACTGTGCTACGGTAATGTTGATAGTTATGGTCCTTAATCGTACATCCTACTTTTGCAAACGAAATGTCCGACTCTCGGGGAGGACTCTTAGATCAGACAATCGGGTCTTTTGTTGGCTTTGTGCTCAGCTCAGCCGGAGTATTTCAATTCTACTTCTCGATGGTACGCGACCTCCTACCATACTAAAGGCCAAAACTACGCCCCGACAGGAAAACATACGGGACTCGATTGCCCGTATTAACTTTTCCTTTAAGTCTAGTTAGACTGTCACAGGTCCGGGCGGCGTCCGCCTTGTGGGTACTGCTTCCCGGTATCCCTCCGGACCTGTCGGGCCCGCTGGTCCGCAGCGGCCGGGACGTAATTATCCAAAGGTGTGCCCGAATAATGGTAGGTGCCTCGGTTCTATTTGTTTTTCGGAACCGAGGTAAGGGAAGACGGTCGCTGCGGTTTTGTCGCCCAGAATACTAATCGCATTTTAAAAACATCTGCACGACGACTACTATCGCCGGATTGTTTCATGACTCGGCCGCAGCTTGCGACAAGTCTTGGCCATAGTGATCAAAATTCCACCGCTTTTTGGCAGGGCTCTAGCGCCTCCGTCGTGTCGACGCGATAAGTGTGTTCCACGGTTTCTCCAAGGACTGCGTTTTTGTTGGCCCGAGGTCGCCCGTTACCTCCTTCTATTGGGCTTCTCCCATGGACAGGGTTCGACCTTTTAATGGTTTGTGATCCTTGCCGGCCCCGCGGAG?TCTCTGTCGCGTGGGCGGGGACGATCGCTTGATCATTA???????????????????????????????????????????????????????????????????????????????????????????????????????????????????????????????????????????????????????????????????????????????????????????????????????????????????????????????????????????????????????????????????????????????????????????????????????????????????????????????????????????????????????????????????????????????????????????????????????????????????????????????????????????????????????????????????????????????????????????????????????????????????????????????????????????????????????????????????????????????????????????????????????????????????????????????????????????????????????????????????????????????????????????????????????????????????????????????????????????????????????????????????????????????????????????????????????????????????????????????????????????????????????????????????????????????????????????????????????????????????????????????????????????????????????????????????????????????????????????????????????????????????????????????????????????????????????????????????????????????????????????????????????????????????????????????????????????????????????????????????????????????????????????????????????????????????????????????????????????????????????????????????????????????????????????????????????????????????????????????????????????????????????????????????????????????????????????????????????????????????????????????????????????????????????????????????????????????????????????????????????????????????????????????????????????????????????????????????????????????????????????????????????????????????????????????????????????????????????????????????????????????????????????????????????????????????????????????????????????????????????????????????????????????????????????????????????????????????????????????????????????????????????????????????????????????????????????????????????????????????????????????????????????????????????????????????????????

**PHYLL**_*Eteone-Phyllodoce*

TGAATTGTCTGGCTCTGGACCTCTATAATATGTGGCTATAGGTCGATTCTTTATGGGAAGAGACCGTATTACAATGCTCATCTTTTAATTTTACAAGTAATGATTGTTGATGTAGTTTAGTAGGGGCTCATAGTTTTTCTATTAGAATGTGTACCCCTTCTATTAACTTAGGGTCTGCAGCAGTGAGCAAGAATGGGTTTACTTTAGAAATGTTGCTTTCATCCTTCAGATTACTTTTTGTTGGTAGTGTCTATCCCTTCAATTTATACAAGCAATATAGGCTTGCGTTTAGAACGTGTACTTGCGTCATCTGTAGCTATTGCTTACTCCTATGTCTTTGTTAAGACTATAATATACTTGTATTAATATCTTTTTCTGCT??????????????????????????????????????????????????????????????????????????????????????????????????????????????????????????????????????????????????????????????????????????????????????????????????????????????????????????????????????????????????????????????????????????????????????????????????????????TTCACGGGAGCTACTTCCCTCGCCCGGCGCCGATAATACTACACCATCCTCTCTCTTCCGCAAAACTTTGCGACCTGCTGTGTGAGTGTTCCAGAACCGTCTGTCCGCCCACCCCTGGGCCACGGCTGCCGGCTCCACGAGCCCAACAGCCAGGCCCATCGAGACAAGAGAGTCTACCCACGGACTCAACCGATACCTGCTCGTGACCCGGCATTCCCCGTTGAGAGTGACAATGGAATGTTCAAGGGATGGAAAATTGCTTGCTGCGCCCACCATCGAGGCCCAAGCTCCAGAGAAGCCCGCTCTGCGGCACGCGGATTAAGCTCCTTGCTATTTGGCCTATGGTCAACTGCTGTCAACGGACCTGTGCGAGCCCAGACCCTGAGCAGCTGGTAACCTCCCGCAACGGCCGAACAAGCTCAATTTGCTCAGCACCGAACCCCGAAAGGAGGCTGATGAGTCAAGGTGCCACCGCCTTGAGACCGCAAGTCAGCCTGGTCCTGCTTCCGCCGACACCCGCTCGAGGATCTCTCGTTCCGTGAAGCTGAGAGAACCGAGAAGGGGTCTTCTCGCCCTGGGAGAAAGCCACCGCC?????????????????????????????????AACATTAGGGTGCCGCTCCATCGAAGTATTAGACAGGTTCATCCTGCTCCCGGTCTTGCGTGCCGTGCGCCCTCCTGCCGCGATCTCCCGAGCTCCCCTGGTAGCGGACCTTTCCTGCCCC???????????TTTTGGCTTATGCTGCTAATACAA??CTTTACATAGTAACGTTAATAGTTATGGTCCTTAATCGTACATCCTACTTTTGCAACAAGCAGCTCCGACCTTCGGGGAAGAGCCTTAGATCAGACAATACGAGTCTTTGCTGGCTTTGTGTTCAGCTACGCTGGAGTATTTCAATTCTCCTCGTCGATGGTACGTGATATCCTACCATGTTTAAAGCCAAAACTACGCCCTGGCAGGAAAACATACGGGACTCTTTCGCCCGTATTAACTTTTCCTTTAAGTTTAGTTAGACTGTCTCAGGTCCCGGCCGCGTTCACTTCGCGGGTACTGCGTCCGGACCTCCCGCCGGTTCCCTTGGGCTCGTTAGTTCTGGGTGGCCGGAACGTAATTATCAAAAGGCTTGCCTGAATAATGGTAGGTGCCTCGGTTCTATTTGTTTTTCGGAACCGAGGTAAGGGAAGACGGTCGTTACGGTTTTGTCGCCTAGAATACTAATCGAATTTTAAGAACATCTGCACGACGACTGAGATCCCCGGATTGTTTCCTGACCCGGCGGCATCCTCTGACGAGTCATGGCCATAGTGATTAGGGTCCCTCCGCCTATTGTTGGGGCTCTAGCATCTTCACCGTGTCGGTGCAACAAGTGCGTTTCACGGTTTCGCCAAGGAATTCGTT?CTGATAGTCCACGGCCGTCCGTTACCTCCTTCTATTGGGCTTTTCCCATGGACAGGGATCGTCCTTTTAATGGTTTGTGACCTTCGTTGGTCCCGCGGGGGCAACTGCCTCGTGGGCGAGGATGTTCACTTGATCATTA??????????????????AAAATTTCCTCTAGGGGCTGATGTGATATCGAGGTTTTAGCGCCCTTTAGTATTAAATGGTTTTCAGGAGAACCTGAATTTAGTAAAGAATATTGGCTTTATTTAAACTAGTTTAAGATATTACTTTATTTTAAATTGGTGCAACAAAGATATATAAATTTTTCTTTATTATTAGTATGTTTCTGTTTGATATGAAAGAGTCATAAGCTTTTCTTTTGTAGTTAATGATAAAAGAGT??????????????????????????CTCCACAAACTAAGCCTACGCGGAGACGACCGCCCCTGCCGAGGTAGCGGGAACGTGTTAGGACAGCCTTGGG?GCTCAGCTGTCACGTCCGGTCCTCCTGTCGGGGCTTCATACTCGTCACGGCGTGCGGCTGTGTCCCTGGGGTCCTTGCGTGGAGCAGTGTAACATCTGCTGACACTCGGATTAACAGCGTTTAGAGGTGGATGGGACGCAA?GTCTCCCCATTGATACCCGTGTCTGA?????GAAGTTCGATCCTTCGGCGAGGCGGTGGGCGCCCTTCTTTGGGATGTCCAGGACGGTTCGGGTGGCCAAAACCGTGTACCAGAGCTTTCGTGAGGTAGCGTTTAGGTGCTGCGGAGGCGGCCGCTTCTCGGACCAGACA????CCCGCCGGATGCGGGTCGAACGTCGTATGTTCGACCTGTGGACTGTACATGCTTTAACTGCGGT?GCGTGTTGGCACTCAGGGTCTCTGGCTTCGGTCGGCACCCCATCAAGCTAAATTGCGCATTGGTGG???CAAACCTACAGCATGAGTGAAGGCAGGCTT???CTGCCCAGTAGTCCCTCCTTGGGGCCTATGCCGCTCGATCTCCGTAGATGAGGCGGACAACGTACACTTGGGTATCTATACACAAGCTGGTCTAGACAACTTGGTTAACTCCTGCACTCTTCGAGTTTCGTATATGACGAACACATTAAGTCCGTGTCGGTTATCGGGCGATCGAGGGCCGCCCCACTCCTACTACGAGGATCTCATAATAATTTGTGAATACAACGCCGATTCGTGTCACCAGGGTCGGATAGGACGTCAGACATCGGGGTGACTTCTTGACGGGAGGCCTGCGGAACAGGCCTTGGCCAGCTGGGGCGCGCAGTCATACGGATGCGTTGAACGTAGATAGAAACTCGTTCCGAAACGGGGCACTGACTCGCCCGTATCTTATATCGTAACGACTGGCTACGGAGATGATCCTTCGGGGTCCAGTGCACAACGAACCAGAGCGCGACAGGACCCCGGGCTTGAGGCGCACTCTGTCGGCTTGCCGATGACATTGTCCCCTACCGCTCCCCGGGCGTGCACTCCTGCCGGCTAATCTGGGGATGGTGTGATTTCGTGCCAGACTGGACCGC?GATAACAGATTATCATCGAGAGGCTGCCGTCTGGGGTACGAGCGGCCTGGAACTGGCAGGGGCTGGCGAGACTGCAAGGTCGATTCGCCGTCGCCGGGGCCTTCCCGTGGACTGCTCACTGGCGGTGGTCGGGTCATTTGCTTCGGCCGGATCTAAAGCCGATTTAACAAATGCTGCGTAGCTGGTGTGATTAAAATCCGGATCTAACGCAAAATTAGCTATCTGCCGACTTTTAGAGAATGAGGGCACGGGCAATTTTTTACGTTTCTTATTTAGAAGCGGAGAGCGGGCAAGCCCTGACCTGGAACGCCCGACCCGGCCGGGCGCGTCCCTCTAAAGTGTCAGCGGATAGAGCTTGAAGTAGGCACTACACATCACTTTTTCGAAGTGTTAACTATTTATTAAGGATTATAACCCTTGATCGTCGTTAATCCCTCCCGTACATTTATTCTTGCTATAACAATTGGATTACGATTGA?

**PILAR**_*Ancistrosyllis-Sigambra*

TGAACCAGCAGGCTTGGGACTTCAAAATTACACGGCAATAGGTCGCAAGTTTACAGGGAGGGACCGCATTACTATGTACTCCCTTTAATTCTATACGTAATAATCATTGATACTGAACTACTGGTGCGCATAGTTTTCACATTAAAATACTCCCCCTTCTAACCCACAATAGATCCGCAGCAGAGAAAAAACTAAAGATTTCATAAGAAATATTGCTCGCAGCCCTCTGATTACATCCTACTGGAAGTCTCTGTCGGCGCCGACTATACTCGTAATTTGGGATTACGACTAGAACGAGTTCATATGTAGGGCGTAAAGATCGCCGTCACACCTATCCCTGCCGAGACTATAACCTACTCGAACTGATATCTTTCTCTGCAAATCAGATAACCACCTC?????????????????????????????????????????????????????????????????????????????????????????????????????????????????????????????????????????????????????????????????????????????????????????????????????????????????????????????????????????????????????????????????????????????????????????ATCGCGGGCGCTACATCCCTTGCTCGGCGCTCCCAATATTACATCATCTTCAATAGTCCGCCAATTCCTACGATGCGCCGAACAAGCGTTCCAGAACCGTCTACCCGCTCTCCCCCGGGAGACACCTGCCAGCTCCACACTACCCACTCTGAGACCCTTCAACGCAAGAAGGAGCTCTCACGGACCCAACAGACTGTTCGTCCTCCCCCGGAATACCCGGAGGCGAGCGACAATGATGTGTGGCAAAAGGCCGTCTCAGATGCAGGCACCCAACATCCAGCCAAAGCCCCCAGACAAGGTCGCTCTTTGGTCCATGGAATAAGAACCTTGTACTCCGGCAAATGGCGACCTGAAGCTAACACACCTGCACGCGCCACTGCTCGGACGAGCGACGCTTCTACCACAACGGCCGTACCGTCTCAACGTGCGGCGCAGCGAACCCACNNAGGAGGCTGGCAGTTCAATGTGGCACCGCCCCCAAACAATGCGATGCCAGTGTCCTGTCCCCAACCTAGAACTATGGAGGATCTCTCGTAGCGAGAAGCTTGGACAACTCAGCATGCGTCTTCGCGCCACGCAAGAGTTCCCAGGCA?????????????????????????????????????????????????????????????????????????????????????????????????????????????????????????????????????????????????????????????????TCTGTTTCCTCTTATGCTGCTCATACATACTTTGACACAGTAACGTTGATGGTTATGGTCCTTAATCGTACCGTCTACTTTTATAACCAGCAGCTCCGACCTTCGGGGAAGAGCCGTAGATCAGACAATCGGTGCCTTTGATGGCTTTGGGCTCAGCTCAGCCGGAGTATTTCAATTCCCCTCTTCGATGGTACGTGCTATCCTACCATGTTAAAGGCCAAAACTACGCCCTGGCAGGAAAACATACGGGACTCTATCGCCCGTATTAACTTTTCCTTTAAGTCTAGTTAGACTGTCTCGGGTGCCGGCGGCGTTCGCCTCGCGGG?????????????????????????????????????????????????GGGCCGGAACGTAATTATCAAGAGCCGTGCCTGAATAATGGTAGGTGCCTCGGTTCTATTTGTTTTTCGGAACGGAGGTACTGGAGGACGGTCGTTGCGGTTTTGTCGCCCAGAATACTAATCGAATTTTAAAAACATCTGCACGACGACTCCGATCCCCGGATTGTTTCATGACCCGGCAGCAGCGTGCGACAAGTCTTGGCCATAGTGATTAGAATCCTCCCGCCTATTGGTGGGGCTCTAGCATATTCGCCGTGTCGGCGTAACAAGTGCGTTTCACAGTTTCGCCAAGGAATGCATTCTTGGTGCTCCAGGGGTGTCCGTTACCTCTTTCTATTGGGCTTGTCCCATGGACAGGGATAGTCCTTTTAATGGTTTGTGATCCTTGTTGGTCCCGCGTAGGCAACTGATGCGGTGGCGAGAACCGA???????????????????????????????AACATTTCCTTTGAAGGCTTTTGCGATAACGCAAATCAAGCGCCTTACAAACATGAACAACTCTCAAGAAGGCTTGAATATAGTAAAAAGTATCGGCTTTATTCTTACAATTAAAT???TCAAAAGTAAATAGAATTAGTGCAACTAGGGAATAATAAACGTCCGTACAATATTCATTAAATCAGCCCGACTAAATAAAGCCGTAAGCTTACTCCCCCAAATTACTAAAGGGGGCA???????????????????????????CTCCACAAACTGAGCCTATGCGGAGACGGCCGCCCCGACCGCGGTCGTGGGAACGCGTTGGGACGACCTGTGCGGCGACGTGCGGACGTCCGGTCCTCCCTTCGGGGCT?CATACTCGTCACGGCGTCTGTCGTTGTCTCTTTGGTCCCTGCGTGGAGCATTGTAACATCTGCTGACACTCGGATTAACAGCGTTTAGAGGCGGATGGGACGCAGAGTCGGCCGATTGATACTGGCGGC?CGTGCATGTGTGTGGAGCCTTCT?GCGGGCGGCGGGTGCACTTCTTTCGGGAGCCCACGACGGTTCCGGCAGTCAAAGGCCGTGGAAAGAGCTCTCGGTGCGAGTGTTATAGATTCGGTGCTGCAGGCCTGCTGGGGGACCAGACCCGTGCCCGCCCGGCTCGGCTC??TCCTGGGTTCGTCGACTGGAGGACTGTGCATGCTCTGACCGCGTTCTGG??CGAGCACTCAGGGTCGGTGGCGTCGGTCGGCACCCTATCAAGCTAAATTGCGCATGGGTTGTACGAAACCTAAAGTATGAGTGAAGG?AGGTTCTTGC?TCTTGGTGCTCTCG?TC?GCCCCCTATGCCGCTCGACCGCCGTCGGTGAGGCGGACGACGTGCACTTGGGTACCTATACACAAGCTGGTCTAGACGACTTGGTTGACTCCTGCGCTCTTCGAGTTTCGTATACGCCGAACACGTTAAGTCCGTGTCGATTACCGGGCAATCGTGTGCTGTCCTACTCCTATTGCGATGATCTCATAATAATTTGTGAATACAACGCCGATCCGTGTTACTAGGGTCGGACAGGACGCCGTGCAGCGGGGTGGCGCAGCGGCGGGAGGCCAGCGGAGTGGGCCTCAGCCAGCCGGGGCGCGCTGCCATACAGATATGTTGAACGCAGATAGAAAGTTGTTCGGAAAGGGGCCTTCAG???CTCTGCT??CTGTATTTTATCGACCGGCTAAGGAGATGGCCCCCTGGGGTCCAGTGCGCAACGAACCAGAGCT?GTCTGGATCCCGGACTTGAGGTGGACTCTGTCGGCTTGCCGATGACGTTGTTCCCTACCGCTCCCCGGGCGTGCACTCCCGTCGGCTAATCTGGCGATGGTGTGATTTCTTGCCGGACTGGACGATTG?TAACAGATTATCATCGAGAGGTTGCCGTCTGGGGTGCGAGTGGACCGGAACTGATGCGGGCTGACGAGACTCGGTGGTCGATTCGACATCGGCAGGACCTGCCCGTTGACCGCTCACTGGCAGCGTTTCCTGCGTTCGCTTGGTCCGGATCTAAAACCAATTTAACAAATGCTGCGCAGCTGGTGTGATTAAAATCTGAATCTAACGTAGAATTAGCTATCTGCCGACTTTTAGAGAATGAAGGCACCGGCAATTTTTTACGTTTCTTATTTATACGCGGAGAGCGGGCAAGCCCTCCGCTGGAACGCGCCGCTCGGTGGCGCGCGTCCCTCTAAAGTGTCAGCGGATAGAGCTTGAAGCAGGCGCTACACATCACTTTTTCGAAGTGTTAACTATTTATTAAGGATTATAACCTTTGATCGTCGTTAATCCCTCCCGTACATTTATTCTTGCTATAACAATTGGATTAGGATTGAA

**PISIO**_*Pisione*

CGGACAGTCAGTCTCTGCACTTCGAACCTTTATTGCTATAGGTCAACTCATTGTAGGAAGAGACCACATCACTATGAACCCCATCCTATCTTATAAATTTTAGAGATTGTCACAACATAACAGGAGCTCTCGGTTTCTTCTCCAAAATACACACACCTCCTATCCTTACTTCATCTAGAGCCGTGAAAAAAACAACGTCTTCAGCTCTAACATTGCTTGCTACCATCAGTTTACTTTTCTCATGATGTTTCTATCAGTGCCCATTACACCTGTTATTTAGGTTTACGCTTAGAACGTGTACATATGTCATCAGCCAAAACTGCAATCTTACTCTTCGCAGTCATGGCTACCGCTCTCTTGTATCAACAGCATTTTCTGCTAAACAATCTACCACCTT???????????????????????????????????????????????????????????????????????????????????????????????????????????????????????????????????????????????????????????????????????????????????????????????????????????????????????????????????????????????????????????????????????????????????????????????????????????????????????????????????????????????????????????????????????????????????????????????????????????????????????????????????????????????????????????????????????????????????????????????????????????????????????????????????????????????????????????????????????????????????????????????????????????????????????????????????????????????????????????????????????????????????????????????????????????????????????????????????????????????????????????????????????????????????????????????????????????????????????????????????????????????????????????????????????????????????????????????????????????????????????????????????????????????????????????????????????????????????????????????????????????????????????????????????????????????????????????????????????????????????????TATGCTGGTAATACAGACTGTACAACGGTAATGTTGATAGTTATGGTCCTTAATCGTACATCCTACTTTTGCAACTGAAATGTCCGACTTTCGGGGAGGACTCTTAGACTAGACAATCGGGTCCTTTGCTGGCTTTGTGCTCAGCTCAGCCGGAGTATTTCAATTCTACTTGTCGATGGTACGTGACCTCCTACCATACTAAAGGCCAAAACTACGCCCCGACAGGAAAACATATGGGACTCTTTCGCCCATATTAACTTTTCCTTTAAGTTTAGTTAGACTGTCTCGGATCCGGGCAATGTCCGTCTCACGAGTACTGTTTCCCGGTCTCCCTCTGGACTCGTCGGGCTCACTAGTCCGCGGTGGCCAGGACGTAATTATCCAAAGGTTTGCCCGAATAATGGTAGGTGCCTCGGTTCTATTTGTTTTTCGGAACTGAGGTAAGGGAAGACGGTCGTTGCGGTTTTGTCGCCCAGAATACTAATCGCATTTTAAAAACATCTGCACGACGACTACGATCCCCGGATTGTTTCATGACCCGGCGGCAGCTTGCGACAAGTCTTGGCCATAGTGATTAGAATCCCGCCGCTTTTTGGCAGGGCTCTAGCGCCTCCGTCGTGTCGATTGGACAAGTGCTTTTCACGGTTTTCACAAGGAATACGTTTATTCTGGTTCGAGGTCGTCCGTTACCTCCTTCTATTGGGCTTTTCCCATGGACAGGACTAGGTCTTTTAATGGTTTGTGATCCTCGCTGACCCCGCGGGG?TCTCCGTCGCGTGGGCAGGGACGATCGCTTGATCATTA???????????????????????????????????????????????????????????????????????????????????????????????????????????????????????????????????????????????????????????????????????????????????????????????????????????????????????????????????????????????????????????????????????????????????????????????????????????????????????????????????????????????????????????????????????????????????????????????????????????????????????????????????????????????????????????????????????????????????????????????????????????????????????????????????????????????????????????????????????????????????????????????????????????????????????????????????????????????????????????????????????????????????????????????????????????????????????????????????????????????????????????????????????????????????????????????????????????????????????????????????????????????????????????????????????????????????????????????????????????????????????????????????????????????????????????????????????????????????????????????????????????????????????????????????????????????????????????????????????????????????????????????????????????????????????????????????????????????????????????????????????????????????????????????????????????????????????????????????????????????????????????????????????????????????????????????????????????????????????????????????????????????????????????????????????????????????????????????????????????????????????????????????????????????????????????????????????????????????????????????????????????????????????????????????????????????????????????????????????????????????????????????????????????????????????????????????????????????????????????????????????????????????????????????????????????????????????????????????????????????????????????????????????????????????????????????????????????????????????????????????????????????????????????????????????????????????????????????????????????????????????????????????????????????????????????????????????????

**POECI**_*Poecilochaetus*

????????????????????????????????????????????????????????????????????????????????????????????????????????????????????????????????????????????????????????????????????????????????????????????????????????????????????????????????????????????????????????????????????????????????????????????????????????????????????????????????????????????????????????????????????????????????????????????????????????????????????????????????????????????????????????????????????????????????????????????????????????????????????????????????????????????????????????????????????????????????????????????????????????????????????????????????????????????????????????????????????????????????????????????????????????????????????????????????????????????????????????????????????????????????????????????????????????????????????????????????????????????????????????????????????????????????????????????????????????????????????????????????????????????????????????????????????????????????????????????????????????????????????????????????????????????????????????????????????????????????????????????????????????????????????????????????????????????????????????????????????????????????????????????????????????????????????????????????????????????????????????????????????????????????????????????????????????????????????????????????????????????????????CCGCCACGCCCTGGCGCCGCTCCGAGCCGGCCATCGCCAGGCACCCGCTGCCCTCGGTCCCGCTCGCCGCGCGCTCGGAGGTCGCGCCCGCCCGTCGCCGCCGGGCAGCGGCCCTGTCCCGCCCCCCCCGCT???GTTTCCTCTTACGCTGCTAATACAAGCCTTAACAAGGTAACGTTAATAGTTATGGTCCTTAATCGTTCATCCTACTTTTATAACCAGAAGCTCCGACCTTCAGGGAAGAGCCTTAGACCAAACAACGCGGGGCATTGCTGGCTTTGTGCTCAGCTTCGCCGGAGTATTTCAATTCTCCTCTTCGATGGTACGTGCTATCCTACCATGTTAAAGGCCAAAACTACGCCCTGGCAGGAAAACATACGGGACTCTTTCGCCCGTATTAACTTTTCCTTTAAGTTTAGTTAGACTGTCTCGGGAGCAGGCTGCGTCCACCTCGCGGGTACTGCGTTCCTGCCTTTTGCCGGATCCCCCGGGCCCGCTGGTCCGGGGTGGCCGGAACGTAATTATCAAAAGATTTCCCTGAATAATGGTAGGTGCCTCGGTTCTATTTGTTTTCCGGAGCCGAGGTAAGGGAGGCCGGTTGTTGCGGCTTTGTCGTCC?AAACACAAATCGAATTTTAAAAACATGTGCACGACAGCTAGGATCCCCGGCTTGTTTCATGACCCGGCGACACCTTCCGAC?AGCTTTGGCCATAGTGATTAGAATCCCCCCGCTTATTGGTGGGGCTCTAGCGCATCCGCCGTGCCGGTGAGACAAGTGCGTTTCACGGTTTCGCCAAAGAATACGTTGTCCCTGGCCTAAAGTCGCCCGCTACCTCTTTCTATTGGGCCTGTCCCATGGACAGGGATCGTCCTTTTGATGGTTTGTGACCCTCGTTGGTCCCGCGGGGGCAACCGCCGCGTGGGCGAAGACGGTCACTTGACTATCAAAA???????????????AACATTTCCTCTAGAGGCTTTTGTGACACCGAGAGTCATACGTCTTTTATAAATAAATAATCTTTAGGAAAGCCTAAACAAATAAAAAAATATTGGTTTTATTGAAACCATTTCTCTAAATAAATCAATGATCAATTAGTGCGACTAAGGGAAATTAAATCTTCCTCTACATAAATATTAGTCTGTCTGATTAAATCTAACCATAAGCTATCTTCTTTGAGCCTTTGACAAGAAGGA????????????????????????????CCACAAACTAAGCCTATGCGGAGACGACCGCCCCCGCGACCGTGGCGGGAACGTGGTGGGAGAGCCTCTGGGCCGGTCGGTGGACGTCCGGTCCTCCTGTCGGGGAAATATACTCGTCACGGCCTCCGCCGCCGGCCCTTGCCTCCTCGCGTTGAGCAGTGTAACATCTGCTGGCACACGGATT????????????????????????????????????????????????????????????????????????????????????????????????????????????????????????????????????????????????????????????????????????????????????????????????????????????????????????????????????????????????????????????????????????????????????????????????????????????????????????????????????????????????????????????????????????????????????????????????????????????????????????????????????????????????????????????????????????????????????????????????????????????????????????????????????????????????????????????????????????????????????????????????????????????????????????????????????????????????????????????????????????????????????????????????????????????????????????????????????????????????????????????????????????????????????????????????????????????????????????????????????????????????????????????????????????????????????????????????????????????????????????????????????????????????????????????????????????????????????????????????????????????????????????????????????????????????????????????????????????????????????????????????????????????????????????????????????????????????????????????????????????????????????????????????????????????????????????????????????????????????????????????????????????????????????????????????????????????????????????????????????????????????????????????????????????????????????????????????????????????????????????????????????????????????????????????????????????????????????????

**POEOB**_*Poeobius*

??????????????????????????????????????????????????????????????????????????????????????????????????????????????????????????????????????????????????????????????????????????????????????????????????????????????????????????????????????????????????????????????????????????????????????????????????????????????????????????????????????????????????????????????????????????????????????????????????????????????????????????????????????????????????????????????????????????????????????????????????????????????????????????????????????????????????????????????????????????????????????????????????????????????????????????????????????????????????????????????????????????????????????????????????????TTCACGGGTGCTACTTCCCTTGCCCGGTACTGAGAATACTATGATGTCTTCTCTCTTCCGCAACATTTCGTGACGTGCTGTACTTGTGTTCCAGAACTGCAAGTCCGCCTACCCGTCGGAGACGTCGGCCGGCAACACGAGACCAACAGTGAAGCTCTTCCAGACATCAAGCGGTGCCCGCGGGGACAACCAAGGCCTGTTTGTTCCCCGGCATATCAGGAAGAATCCCCCAATGACGTGTTCAAGGGTTGGAAAACCACTGAATGCACACGCCATCCTGCCATCAACCACTGACAAGCCCGCTCTGACGCCCGAGGATTAAGATCCTAAATAAAATGCATACGGGGACCTGTACCCAACACACCTGGGTGTGCCGAATCTCCTTAGAGCTTTGCACTTCCCGGAACGACGGGACCGTCTAAACTGCTGGTGCAGCGAACTCACCAAGGCAGCAGAAAGCTCCTTGCGCCAGATCCCTGAGGGCATGCGCTGCCAGCCCCCAGCCTCCAGCGCTGAGCCAAAGAGGATCTCTCGTAGCGCGTCTATGAGACAACCAAGATGACGTCTATGCGCCACGTAAGAGTCCCAGCGCCAGGGTAAGCTTCTCTGAAATGCCACGT??CTAATACACCAGGGCGTCGCTCTGAGTCAGCTAAGACTCTGCAACTTCTGCTAACGGAGCTGCGCCCCGCGAGCTCGCCTGTTGCGATTGCCCGAGCTTCCCGGACAGCGGCCCGCTCGCGCCCCCCCCGCT???TT????????????????????CAGGCCCTCGATGGTCAATGCGAATAGTCATGGTCATTGAAGGTACATCCTACATTTGTAACAACAAGTTCCGACCTCAGGGGAAGAACCAGAGACCAGACAATCGGGC??TTTGCTGGCA?CGGGTTCAGCGCAGCCGGAACTTTTCAATTCTACTCCAAGATGGCAGGGTAACGCCTGCCATGGTGAAGGCCAACACTACGTCCCGACAGGAAAACGTGCGGGACTCTTTGGCTCGTACTGACTTTTCCTTGGAGTTTAGTCGTCCTGTCTTTGGTGTCTGGAACGCT????????GGGGACTGCGGTTCGACCCTCCGTCGGCTCTCCGTGGCTAACCGGTCACGGGTGGCTAGACCTTGACAGCCAAAAGATTTACTGAACCAGTTGTAGGTGCCTCGGTTCTGTGTGTTCTCAGGAAGCGAGGTAAGAGAAGACGGTCGTTGCGTCGGTATCGACCAGAAAAACAATCGAATTTTCAGAACATGTGCGCGACTACTGCGATATCCACGTCGTATCATAAATGGGCGTCAGCCCCCACTCAGTTTTGGACTTAGTGATTGGAATTCCCTCGCTCATTGCTGGGCCTTTGGC?CATCCGTCTT????GTGTAACTAATGCTTTCCATGGATCCGCCGAGGAATACGCTTTAGACGGTCCAGGGCTAG?CGTTACCTCCTCGTTCTGGCATTATGCCACGGACAGGGACAGTCCGTTGA?CGTTCGGTGGAGACTGTCGGCCATTTCGCCGCGGCCTCCATGGTGAGGAGGATTTATGCTTGGACGTCA??????????????????AAAATTTCCTTTGAAGGCTAATGTGATAACGAAAATCTCCCGTCATCAACCTCTAAACTATTTTTAGGAAAGCCTAAAACATTTAAAAAATGCAGGCTTTATACAAAATTT????????????????ACTTAGTATTTGTGCGACATAGGTAAGTAAAACATCCTCTATATAAAGTACTATTCAGTCTGATCAATTAAAGCCTCAAGCTTTCTTTCTTGAGTCCTCGACAGAAAACT??????????????????????????CTTGACAAACTAAGCCTATGAGGAGACGACCGCCCCCGCGCGCGTGGCGGGAACGTGAAAGGAGGGCCGTGCTGGATCACAGGGGGCACCCGGTCTCACTGTGTGGCGTTCATACTCGTCACGTGCCTCTGGTCGTCGGCTGAGCTCCTAACGCGGAGTAGCGTAACATCCGCTGACGCACGGACTAACAGCGTTTAGAGGGGGATGGGACGCAAGGTCGAACCGTGGGTACC?GGTTTGTCAT?????????GGATCGTGCAGACGGAGCCAAGGGTCACTTCCACGGAGACACCGCGACGGTTCCGGCCGTCAAAGCCGCGGGGAAGAGTTGCGCTTGCCAATGTTATAGCCCCGATGGCGTTGGCCGGCTGGTGGACCAGAAAGCTTGCCGGGGGTTGCGCGCCC??CTGG?????TTTGACT????GACTCTCCATCCGCTGTGGGTGCC?GCGCCACCCCGAGGAAGGTCAGCGGCGACACTCGGCACCCCATCA?GCTAAGTTGC?CACTGGCG?CATG?AACCCCAAGCATGAGCAAATCTCAGCTCGTCTGAGAGAGCGGTCCTGCTATCGGGCCCGCGCCCCTTGAGTCTGGTCGACGAGGGGGACTACGTACACTTAATTTCTCGCATGCAAGCTGATCTTAGCTGCCTGACCAACTCCTGCGCTCATCTGGTATCGTACCTGACGAACTCGTTATGCCCGTTTCAGTTATCGGGTCTCAGAGTGCCGCCCTACTCTTGCAACGC?GATCTCACAACAATTTGTGAATACAACGCCGCTCCGTGTCACTAGCGTTGGGCAAGACGTCGGGTATGTCAAATCCTCCTCGACGGGAGGGCTGCGGAGTCGGCCTTGGCTAGCTGGGGCGCGCAGTACTACAAGTATGTTGGGCGTGAGTGGAAAACCGTTCGACAGGCGGGCAACGACACTCCCTAC??TCGCAACGGAGCGTCTGGACGAGGATATGAGGCATCAGGGCCACGAGCACAACGAACCAGTGCTCCACGTGAGACCGGAGTTGTAGCTGACTCTGTTATCCTTAAGAAGAGGACTGGCCTTACCGACCTGCGGGCGTAACCTCACGGGGACTAAGCTGGGGATGGTGTTATTACTCGTCAGATATTACCATTGAGTATAGGTTTTTCTCAGGGGGCCGTCGTTTAAGTCACGAGCATTGAGTACCCGTCACAGGAGGCTGTG????????????????????????CCTGACC??TAAGTGGAGTGCCTACTTGCGGGTTTCACCGCGTGGACGACGACCGTCTG?AGGGTTGATCCAAAAAATGCGATGGTGACAATGCGATTAAGACCCGGATCTGACGCGTGATCAACTATACGCTACGATTTCGAGGGTGGGAGCGTTAGCCAATTCTCATGCTTCGCAGTCAGAGGCGGGCAAAACGCGAGTTGCCGCCTCCCGGGCTGTGGTCG??TGCTGGTGCCTCTCTGAAGTCGTAGTGTATAGCGCTCCAAGCTGACGCTACACTTGGCCTTTTCGAAGCGTTAACTATTCATGAAGGATTACAGCCAATGATCGTCGTTAATCCCTTCCGGATCCAA?CTCCTAATCTAACAATCAAGTTAGGATTGAC

**POLYG**_*Polygordius*

?????????????????????????????????????????????????????????????????????????????????????????????????????????????????????????????????????????????????????????????????????????????????????????????????????????????????????????????????????????????????????????????????????????????????????????????????????????????????????????????????????????????????????????????????????????????????????????????????????????????????????????????????????????????????????????????????????????????????????????????????????????????????????????????????????????????????????????????????????????????????????????????????????????????????????????????????????????????????????????????????????????????????????????????????????????????????????????????????????????????????????????????????????????????????????????????????????????????????????????????????????????????????????????????????????????????????????????????????????????????????????????????????????????????????????????????????????????????????????????????????????????????????????????????????????????????????????????????????????????????????????????????????????????????????????????????????????????????????????????????????????????????????????????????????????????????????????????????????????????????????????????????????????????????????????????????????????????????????????????????????????????????????????????????????GTGCCGATCTGAGTCAGATAAGGCCAAAAAAGTCCTGCTAGCGGAGCTGCTCGCCGCGCGCTCGCCTGTCGCGCCCGCTCGAGCTTCTCGGGCAGCGGTCCGGACCCGCCCCCCCTGCTCCTGTTTCCTCTTATGCTGCTAATACAAACTTTTACACGGTAACGTTAATAGTTATGGTCCTTAATCGTACCTCCTACTTTTATAAGATGAAGCTCCGACCTTCGGGGAAGAGCCTTAGATCAAGCAATCGGGTCCTTTGCTGGCTTTGGGTTCAGCTCAGCCGGAGTATTTCAATTCTCCTCTTCGATGGTACGTGATATCCTACCATGTTAAAGGCCAAAACTACGCCCTGGCAGGAAAACATACGGGACTCTTTCGCCCGTATTAACTTTTCCTTTAAGTTTAGTTAGACTGTCTCGGGTCCAGGCTGCGTTCACCTCGCGGGTACTGCGTCCTGACCTCCCTCCGGTTCCCTTGGGCTAATTAATTCTGGGCGGCCGGAACGTAA?TAGCAAAAGTTTGGCCTGAATAATGGTAGGTACCTCGGTTCTATTTG?GTTTCGGAACTGAGGTAAGGGAAGACGGTCGTTACGGTTTTGTCGCCTAGAATAGTAATCGAATTTTAAAAACATGTGCCCGACACCCACGATCCCCGGCTTGTTGCATGACTCGGCGGCAGCTTACGACAAGTCTTGGCCATAGTGATTAGAATCCCCCCGCTTATTGGTGGGGCTCTAGCATATTCGCCGTGTCGGCGTAACAAGTGCGTTTCACAGTTTCGCCAAGGAATGCTGTGTTCTTCGTCCGAGGTTGTCCGTTACCTCCTTCTATTGGGCTTGTCCCATGGACAGGAATAGTTCTTTTAATGGTTTGTGATCCTCGTTGGTCCCGCGGGGGCAACCTCCGCGTGGGCGAGGACGATCACTTGATCATTAAAA???????????????AACACTCCCTTTGGAGGCTAGTGTGATAACGAGGGCAAAGCGCCTTACCCCCTTAAATAGCCCTCAGGAGTACCTGGAAAAAATGAAAAGTGTTGGCTTTACTCTACCTGCATTAATAAGTAAAATTAATCCGAGTCTGTGCGACAAAGGTATACCAATCTTCCTTCAATAATAG?ACCAATCTGCCAGATCAAAATCAGCCACAAGCTATTCTTCTTGAGCCACTAACAGAAGAGT????????????????????????????CCACAAACTAAGTTTATGCAGAGACGACCGCCCCTACCTGGGTAGTGGGAACGTGTTAGGACAGCCTCTGGGGCAGTGTATGGACGTCCAGTCCTCCTGTCGGGGCTTCATACTCGTCACGGCGTCCGTCGCTGTCTCTTGAGTCCTTGCGTGGAGCAGTGTAACATCTGTTGACACACGGATT????????????????????????????????????????????????????????????????????????????????????????????????????????????????????????????????????????????????????????????????????????????????????????????????????????????????????????????????????????????????????????????????????????????????????????????????????????????????????????????????????????????????????????????????????????????????????????????????????????????????????????????????????????????????????????????????????????????????????????????????????????????????????????????????????????????????????????????????????????????????????????????????????????????????????????????????????????????????????????????????????????????????????????????????????????????????????????????????????????????????????????????????????????????????????????????????????????????????????????????????????????????????????????????????????????????????????????????????????????????????????????????????????????????????????????????????????????????????????????????????????????????????????????????????????????????????????????????????????????????????????????????????????????????????????????????????????????????????????????????????????????????????????????????????????????????????????????????????????????????????????????????????????????????????????????????????????????????????????????????????????????????????????????????????????????????????????????????????????????????????????????????????????????????????????????????????????????????????????????

**POLYN**_*Lepidonotus*

CGTACTATCGGTCTTAGCACTTCTAAACTCCTTTGCTGTAGGACAAATCCTTATAGGAAGAGATCACATCACCATGTTCCTCATTCTATTTCGAATGTTCTAGAAATTGTCATGATATAGCAGGGGCTCTTAGTTTCCTTACCAAAATATACACCCTTACTATTTATACTTCTTCTAGAGCAGGGAAAAAGAAAACGTCATTATCTCTAATATTGCTTGCAGCCTTCAGATTACTTTTTATATGTGGTTTCTTTCAGAGCCCTCTATACCAGTAGTTCAGGTTTACGACTTGAACGTGTTCTTATGTGATCGGCTAAAATTGCTATCCTATATATCTTTGCTAAGGCTATCACGCACTCGAACTAACTGCTCTTTCTGCAAGGC??????????????????????????????????????????????????????????????????????????????????????????????????????????????????????????????????????????????????????????????????????????????????????????????????????????????????????????????????????????????????????????????????????????????????????????????????????GTCACGGGCGCTACTTCCCTTGCCCGGCGCCACCGGTATTACGCCATCCTCAATCTTCCGCTATCTCCTGCGATATGCCGTGTTTGCGCTCCAGAACCGTCTGCCCGCCCACCCTTGGGCCACATGTGCCGGCAACACGAGCCCCACAGCGAGGCCCTTCGGTGCGTGAGGGACCAGCCACGGACTCAACCGACAACGCCTCTGCCCCAGGCACCCCTGGAGGAGTCCAAGAATGTCCTGTTCAAGGGATGGAAAATCCCTCGAGTGCCGCTGCATCGAGCCCCAAGCTCCCGACAAGCCCCCTCTCCCGTCCGAGGATTAGGCGCCTTGCTGTCCGGCCTATGGGGACCCGCATCCAACACACCTGCATGGGCTCAATCCCCACCGAGCCAACATCCCACCGCTCAGCCGGCACCGTCTAAACTGCTGGCGCAGCGAACTGCCCAAAGAGACCAAGGAGTCGTCGTGCCACCGCCTTCAGACAACGCCCCGCCCGGCCCCAGCCTCCAGCGCTGAGCCATCGAGGATCTCTCGTTCCGCGAAGCTCGGACAATCCAGCACGCGTCTTTGCGCCAGGAAAGAGTGCCCAGGCC?????????????????????????????CTGCGAAGTCCCAGTGCCGTTCTGAGCCGGCTTAGGCTCTGCTAGTCCTGCTCTCGGTCCCGCCCGCCGCGCGCCCGCCTGCCGCGTCCGCTCGTCCCCCTCTGGCAGCGGTCCGCACCCGCCCCCCCCGCT?CTGTTTCCTCTTATGCTGGTAATACAGACTGTACAACGGTAATGTTGATAGTTATGGTCCTTAATCGTACATCCTACTTTTGCAACTGAAATGTCCGACCTTCAGGGAGGACTCTTAGATCAAACAATCGGGTCCTTTGCTGGCTTTGTGCTCAGCTCAGCCGGAGAGTTTCAATTCTACTAGTCGATGGTAAGTGACCTCTTACCATTATAAAGGCCAAAACTACGCCCCGACAGGAAAACATACGGGACTCTTTCGCCCGTATTAACTTTTCCTTTAAGTTTAGTTAGACTGTCTCGGATCCGGGCAATGTCCGCCTCGCGGGTACTGTGTCCCGGTCTCCCTCTGGACCCGTCGGGCTGACTAGTCCGCGGCGGCCAGGACGTAATTATCCAAAGGCGTGCCCGAATAATGGTAAGTGCCTCGGTTCTATTTGTTTTTCGGAACAGAGGTAAGGGAAGACGGTCGTTGCGGTTTTGTCGCCCAGAATACTAATCGAATTTTAAAAACATCTGCACGACAACTACGATTCCCGGATTGTTTCATGACCCGGCAGCAGCTTCCGACAAGTCTTGGCCATAGTGATTAGAATCCCTCCGCCTTTTGGCGGGGCTCTGACGCCTCCGTCGTGTCGACGCGACAAGTGCGTACCACGGTTTCGCCAAGGAATACGTTTATGTTGGTCCGAGGTCGTCCGTTACCTCCTTCTATTGGGCTTCTCCCATGGACAGGGTTAGACCTTTTAATGGTTTGTGATCCTCGCCGGCCCCGCGGGGGCAACCGTCGCGTGGGCGGGGACGATCGCTTGATCATTAAAA???????????????AACATTTCCTTTGAAGGCT?TTGTGATAACGAAACTAAAGCGCCTAAAACATATATATAGCCTTTAGGAGTTCCTAAATTATCTAAAAAGCATAGGTTTTAGCTTAATAACTTTA???????????????TAACCTTGGTGCGACCAAGGTAAATAAAACATCCTTAACAAATAGTATTAATCAGCCTGATCTTAAAAAACCTTGAGCTTTCTTCCTAGAGCTATTGATAGGAAGGT??????????????????????????CCCCACAAACTCAGCCTATGCGGAGACGACCGCCCCTGTCGCGGCAGTGGGAACGTGTTAGGACGATCACTGGCGCGTCGTGCTCCCGTCCGGTCCTCCTGTCGGGGCTTCATACTCGTCAGGGCGAGGGCCGGCGTGCCTTAAGTCCTTGCGTGGAGCAGTGTAACATCTGCTGACATACGGATTAACAGCGCTCAGAGGCTGATGGGACGCAAAGTCGCCTCGTGGATACT?GCTCGGGC?GTTCAGACGTAGATCC??GGGGCGGTCGGAGGGCGCCCTTCCACGAGGAGCCCACGACGGTCCTGGCGGCCAAAGTCCAGAGAAAGAGCGGTCGCCTTCCGTGTTATAGTCTCTGGTTCGGAGGCCCGCCGGCGAACCAGACATGCCGCCGCTTCTTCGGGGCGGCCCGTCGGTTTGTCGTCGAGTGGACTGTACATGTGCCGGCTGCCA?CGCTGTGGGTCGCCTAGGGTCCGTGGCGTCGATCGGCACCCCATAAAGCTAAATTGCACATTGGCTCTACGAAACCCACAGCATGAGTGAAGGGCGTTC????CGTCCGTGCAGTCCGCCCCGGTGGCCTGTGCCGCTCGGTCGCCGTCGACGAGGCGGACAACGTACACTTGGGTACCTATACATAGGCCAGTCTAGACGACTTGGTTAACTCCTGCGCTCATTGAGCATCGTACCTGACGAACTCATTGAGTCCGTGTTAATTATCGGACCATCGAGTGCCGCCCTACCTGTACCGCGATGCTCTGACCCGGAACATTCGGATTCAATTCAATCATGACGGCTTGCGGAGGGCACAGCGTTGGGCATCGCCGATCCGCCGCAACGGGAGGCTCGTGGAACGGGCCTCGGCTAGCCGGGGCGCACGATCATACGGATGCGTTGAACGTAGATAGAAACTCGTTCCGAAACGGGGCAAAGA???TCCCGTAATCTATATCATATTGACGGGACACGGAGATGGCTCTCCGGGAGCCAGTGTACAGCGAACCAGTGCTCGGCGGGACCCCAGACTTGAGGCGGACTATGTCGGCTTGCCGATTACGTTGTACCCTACCGCTCCCCGGGTGTGCGCTCCTGTCGGCTAAACTGGGGACGGTGCGATTTCGTGCCCGACTGGACCGC?GATAACAGATTCTCATCGCGAGGCTGCCGTCTGGGGTGCGAGCGGCCTGGAACCGGCAGGGGCTGGCGAGATGGGCGCGTCGACTCGCCGTCGCYGGGACCTTCCCGTGGACCGCTCACTGGCGGCGTCAAGCGCATCCGCTTCGTCCGGGTCCAAAGCCGATTTAACGAATGCTGCGTAGCTGGTGTGATTAAAATCCGGATCTAACGCAGAATCAGCTATCTGCCGACTTTTAGAGAATAGGAGCGCGGGCAATTTTTTACGTTTCTTATTTAGAAGCGGAGAGCGGGCAAGGCCTCCTCTGGAACGCCCGGCTCGGTCGGACGCGTCCCTCTAAAGTGTCAGCGGATAGAGCTTGAAGTAAGTACCACACATCACCTCTTCGAGGTGTTAAATATTTATTAATGATTATAACCTTTGATCGTCGTTAATCCCTCCCGTACATTCATTCTTGCTACGACAATTGAATCGGGATTGGC

**POTAM**_*Potamodrilus*

???????????????????????????????????????????????????????????????????????????????????????????????????????????????????????????????????????????????????????????????????????????????????????????????????????????????????????????????????????????????????????????????????????????????????????????????????????????????????????????????????????????????????????????????????????????????????????????????????????????????GGCGTYGCGGAAAATATTTATTTTTATAAATTTGTTATGAAATTTATAAGTTAAGATGGAAAGTGTAATAAATATTAGTTTAATAGTGTATTATTTTGTGATAGATAAGATATCTTCTCTCTGTTATATTTCTCTTTTTTCACTTTTTTACGTCTAGCAGATACTATTTTCTTGAGGCATATCTTATTTTATGGTTACTTTTATTCTATACTGAGAAAAGCTATTGAGTGTTATTATAATTTTATATTTAGAGATAAACAACCCCC???????????????????????????????????????????????????????????????????????????????????????????????????????????????????????????????????????????????????????????????????????????????????????????????????????????????????????????????????????????????????????????????????????????????????????????????????????????????????????????????????????????????????????????????????????????????????????????????????????????????????????????????????????????????????????????????????????????????????????????????????????????????????????????????????????????????????????????????????????????????????????????????????????????????????????????????????????????????????????????????????????????????????????????????????????????????????????????????????????????????????????????????????????????????????????????????CCTGTTTCCTCTTATGCTGCTAATACAAGCCTCGACATGGTAACGTTAATAGTTATGGTCATTAATCGTACATCCTACTTTTGTAACGAGAAGCTGCGACTTTTTGGGAGCAGCCGTAGACCAAACAATCAGGTCTCTTGCTGGCTTTGTGTTCAGCTCAGCCGGAGTATTTCAGTTCTCCTCGTCGATGGTACGCGACATCCTACCATGCTAAAGGCCAAAACTACGCCCTGGAAGGAAAACATACGGGACTCTTTTGCCCGTATTAACTTTTCCTTTAAGTTTAGTTAGACTGTCTCGGGTTTAGGCTGCGTTCGCCTCGCGGGTACTGCGTCCTGACCTCCATCTGGTAGGCGCGGGCTAATTAGTCCGGTCTGGCCGGAACGTAATTACCAAAAGG?CTGCCTGAATAATGGTAGGTGCCTCGGTTCTATTTGTTTTTCGGAGCCGAGGTAAGGGAAGACGGTCGCTACGGCTTTGTCGCCTAGAACACTAATCGTATTTTAAAAACATCTGCACGACGACTACGATCCCCGGCTGGTTTGATGACCTGGCGGCAGCTCCCGACAAGTTTTGGCCGTAGTGATTAGAATCCCCCCGCCTATTGGTGGGGCTCTAGCATATTCGCCGTGTCGGCGCAACAAGTGCGTTTCACAGTTTCGCCAAGGAATGCGTTCTTCTTGGTCTGAGGTCGTCCGTTACCTCCTTCTATTGGGCTTATCCCATGGACAGGGATTGTCCTTCTGATCGTCTCTAAACTTTGTTGGG?ACGCGGGGGCAACCGCCTCGCG?TCGAGGAAGCTTAGCGAACGATTGGGA????????????????????????????????????????????????????????????????????????????????????????????????????????????????????????????????????????????????????????????????????????????????????????????????????????????????????????????????????????????????????????????????????????????????????????????????????????????????????????????????????????????????????????????????????????????????????????????????????????????????????????????????????????????????????????????????????????????????????????????????????????????????????????????????????????????????????????????????????????????????????????????????????????????????????????????????????????????????????????????????????????????????????????????????????????????????????????????????????????????????????????????????????????????????????????????????????????????????????????????????????????????????????????????????????????????????????????????????????????????????????????????????????????????????????????????????????????????????????????????????????????????????????????????????????????????????????????????????????????????????????????????????????????????????????????????????????????????????????????????????????????????????????????????????????????????????????????????????????????????????????????????????????????????????????????????????????????????????????????????????????????????????????????????????????????????????????????????????????????????????????????????????????????????????????????????????????????????????????????????????????????????????????????????????????????????????????????????????????????????????????????????????????????????????????????????????????????????????????????????????????????????????????????????????????????????????????????????????????????????????????????????????????????????????????????????????????????????????????????????????????????????????????????????????????????????????????????????????????????????????????????????????????????????????????????????????????????

**PROTD**_*Protodriloides*

???????????????????????????????????????????????????????????????????????????????????????????????????????????????????????????????????????????????????????????????????????????????????????????????????????????????????????????????????????????????????????????????????????????????????????????????????????????????????????????????????????????????????????????????????????????????????????????????????????????????GGCGTCGCGGACAATGACAACTTAGATTAATCAGTATAATAGATAACTGCAACCACGAGAAAAATATATAACTACACCCATACTAGCATGATATTTTACGGTACACTCTATGCCTGCTCTCTTACACATTGTGCTTCTCTTGTACTCCTGTAAGAAAAAGTTGCCTGTCACTTTACACACGGTTCTGTTACCGTTAACGCTCCTCAGACTTGGTCCAAGATTCTTTTTTAACATGTCACATCCCAACTCCCTAAGGAATAACCCCC???????????????????????????????????????????????????????????????????????????????????????????????????????????????????????????????????????????????????????????????????????????????????????????????????????????????????????????????????????????????????????????????????????????????????????????????????????????????????????????????????????????????????????????????????????????????????????????????????????????????????????????????????????????????????????????????????????????????????????????????????????????????????????????????????????????????????????????????????????????????????????????????????????????????????????????????????????????????????????????????????????????????????????????????????????????????????????????????????????????????????????????????????????????????????????????????????TTTCCTCTTATGCTGTTAATACAAGCTTTTATACAGCGACGTTAATAGTTATGGTCCTTAATCGTATCTTCTACTTTTGTAACTATAAACCCCGACCTCGCGGGAGGGGCCATAGAACAGACAATGCCGCCCTTTGCATGCTTCCAGATCAGCTTCGCCGGAGTATTTCAATTCTCCTCTTCGAAGGTCGGTGCTATCCCACCTTGTTAAAGGCCAAAACTACGCCCTGGCAGGAAAACATACGGGACTCTTTCGCCCGTATTAACTTTTCCTTTAAGTCTAGTTGTACTGTCTT?GGCCGAGGCAGCGTCTGTCCCTTGAGGACTGCGTCCTCGGCTCCTGTCGGCGTGGCCTGGCCCACCGGTCGGTCGCAGACGACGCCTAATTATCAAAAGCTCGGCCTGGATAGTGTTAGGTGCCGGGCTTCTATGCGTTTTTCGGAACTCTGGTAAGGGAAGACGGTCGTTACGGTTTTGTCGCCTAGAATACTAATCGAATTTTAAAAACATGTGCACGACGACTACGATCCCCGTTTTGCCTAACGACTCGGCGGCAGCTTCCGACAAGTCTTGGCCATAGTGATTAGAATCCCCCCGCTTATTGGTGGGGCTCTAGCGCAAGCGCT??GTCGGCGTAGCAAGTGCTTTTCACAGTTTCGCCAAGGAATGCGTTCCTGCTGGGCCAAGGCTGTCATGTACCTCCTTCTATTGGGCGTGTCCCATGGACAGGGATAGTCCTTCTGATGGTTTGTGGTGGACGATGGCGCGGTGCTGGCGACAGGCTCTGATGCGTGGTCTACCGCTTGTTCATCGAAA??????????????????????????????????????????????????????????????????????????????????????????????????????????????????????????????????????????????????????????????????????????????????????????????????????????????????????????????????????????????????????????????????????????????????????????????????????????????????????????????????????????????????????????????????????????????????????????????????????????????????????????????????????????????????????????????????????????????TTACCATCGTTGAGAGGTGGTGAAGGCTCAAAATTGCCCAATGTTTGCCCGTCTGGGCTATTAGTCTTGGGCTTCTC??????ATTGATTGGTGCACTGACATGGG????TCACGACGATTGCGGGGGTGTATACTACTGGTGGGGCTTCTCGGGTAGTGCTTTCAAGATTCT??GGCTGCGGC?CTCTGGGCGAGTCGA???ATGCTTGCTC?TTTTGGCTTGCTCGTCGGGTA???CATTGGTAGACTGCGCATGTTCTGGTGTTAGTCGCGTTCGTTT?TTCCCCGTCCGTGGCGTGGATC?????CTTTACAAGCTAAATTGTGTCAAGGAAGT?????AAACCCGGCATGAGTGA?????????????????TTAGTGGAGCCT???ATGGTCCCGCTCCGCCTGTC???CGTATGCAGGGCGGAAAGCATACACTTGGGTACCTACATACAGGCCGGTCTAGACTGTTTGGTTAACTCCTGCGCTCGAAGAGTATCGTATCTGTCGAATACATTAAGTTCGTTTTAATTACCGGA?CATTAGGTGCAGCTCTACTTGTACCGTGC?GATCTCACAATGATTTGTGACCTCAACGCCGATCCGTGTCACTAGGTTTGAGTACGATGTCGAACATCGGGTGGTCGTTCCGACAGGAGG?TGGGGCGTTAGGGGTACGCTAGCGTTGGGGCTTCATCATACGGATACGTTGAACGTCCGAGGAGAGTTTCTCGGAGGT?????GACCCCTTGTCCGT????CGGATGATATTCAGCAGATACGGGGAGTTGCCTTCGTGCAAATGTGTACAACGACCTGGAGCTTTGCGGGTTCCCGGACTTGAGCGCTAAAGTGTTGGATTATCAAGCTTGTAA???CCCGCCGGCTCTCGGGCGTGCACACTCGTGGGCTAATCAGCGA??ATCGTAAGCTCGTACTGCTAATGACCGT?GTTAATAGAATATCATCGAGAGGCTGCTTTCTGGGGGCAGAGCAGATTTATGGCATCGAGAACTGACTTGCCTTCCGGCTCGTCTGA?TCTCG?TGTGTCT?TCCGGTGGATGACTCACTAGTCGCTGTGGCGGTGATTTTTTCGGCTTGAACAAAAGTCGATTTAAAAAATGTTGCTTAAA?GGTGTAATTAAAACCCGG??????????????????????????????????????????????????????????????????????????????????????????????????????????????????????????????????????????????????????????????????????????????????????????????????????????????????????????????????????????????????????????????????????????????????????

**PROTO**_*Protodrilus*

????????????????????????????????????????????????????????????????????????????????????????????????????????????????????????????????????????????????????????????????????????????????????????????????????????????????????????????????????????????????????????????????????????????????????????????????????????????????????????????????????????????????????????????????????????????????????????????????????????????????????????????????????????????????????????????????????????????????????????????????????????????????????????????????????????????????????????????????????????????????????????????????????????????????????????????????????????????????????????????????????????????????????????????????????????????????????????????????????????????????????????????????????????????????????????????????????????????????????????????????????????????????????????????????????????????????????????????????????????????????????????????????????????????????????????????????????????????????????????????????????????????????????????????????????????????????????????????????????????????????????????????????????????????????????????????????????????????????????????????????????????????????????????????????????????????????????????????????????????????????????????????????????????????????????????????????????????????????????????????????????????????????????CCACAGCGCACAGGCCCCGTTAGGAGCTTGCTGTAGCCAAGCTCCTCCTACTCTCGGAGCCGCTCCAGGCTCGAACCGCTGTTGTGTCCCCACGAGCTTCACCGACTCCGGTCTGCTCCTGCCCCCCCCGCCCCTGTTTCCTCTTATGCTGCTAATACATACTTTTATACAGTAACGTTAATAGTTATTGTTATTTATTGTAACTCCTACTTTTGTAACTAAAAGCGTCGAGCTTGTGCGAGACGTCTTAGATCAGACAATTCGGGCTTTTGCTGGCTCT??GTTCAGCTTCGCCGGAATATATCAATTCTCTTCTTCGATGGTAAGTTATGTCTTACCATGTTAAAGGTCAACACTACGCCCCGGCAGGAAAACATACAGGACTCTTTCGCCTGTATAAAATTTTCCTTTATGTTTAGTTAGACTGTCTCGGGGTGTCACGCCGTCCGCTCACAAGGTACTGGGCGATGTCCTTCCTCCGACCCCACCCGGCTGACTAGTCAGGGGAGAAGAGGACGTAGTTATCAAAAGCTGTGCCTGAATAATGGTAGGTGCCTTGGTCTTATCTGTTCTCCGAGACTGAGGTAAGGGAAGCCGGTCGTTGCGCCTGTGTCGGCCAGCATACAAATCGAATTTTAAAAATGTGCATGCGACAACTACGATCAGGAGCTTA?TTAATGACTTCCCTGCAGCTTCCGACAAGCTTTGGCCATAGTGATTAGAATCCCCCCGCTGATTGGTGGGGCTCTGGTGCATGGCGCGTATGGCGATCGTTAGTGTTTCTCACAGTTTCGCCAGGGGGTACAAT?GCCATGGGCCATGGCTGTCTTTTAACCTTCTCTATAGGAATTACTCCTTGGATAGGAATAGTTCTTTCAACGATTTGTGAGCATCGCTGGCCATTCGAGGGCAACCCTCTAACG?GCAGAGACGTTCACTTGATCGTTAGGA???????????????AACATTTCCCTTGGGGGCTTTTGTGATTACTTTA?CCTAATGCCGCGTATCTACGACTTATATTCAAGAGAACTTGAATCAAATGAAGCATATAGGCTTCACAATAAATGTAGCCC??????????????TCTTGTTCATGCGATGATAGTTTC???CTCCTCTACTAATTACCTTCCAAATTTACCCTAGACCGAAAAGCCTTAATCTATCCTCCTTGAATTACAGACAGGAGGGTGGC?????????????????????????TCACAAACTAAGCCTATGCGGAAACGGTTGCCCGTCGTGGAGCGGCGGGGAAACGTGTAGACGCGT?CTGTCACGTGGCGCGCCGGCCCCCTCCAGCTGTTCCGGCTGCATAATCGCGTCGGTCGGTGTGTCCGTGACGGAAAATCTTACGTAGAGCAGTGTAACATCTGTCCGGACACGAATT????????????????????????????????????????????????????????????????????????????????????????????????????????????????????????????????????????????????????????????????????????????????????????????????????????????????????????????????????????????????????????????????????????????????????????????????????????????????????????????????????????????????????????????????????????????????????????????????????????????????????????????????????????????????????????????????????????????????????????????????????????????????????????????????????????????????????????????????????????????????????????????????????????????????????????????????????????????????????????????????????????????????????????????????????????????????????????????????????????????????????????????????????????????????????????????????????????????????????????????????????????????????????????????????????????????????????????????????????????????????????????????????????????????????????????????????????????????????????????????????????????????????????????????????????????????????????????????????????????????????????????????????????????????????????????????????????????????????????????????????????????????????????????????????????????????????????????????????????????????????????????????????????????????????????????????????????????????????????????????????????????????????????????????????????????????????????????????????????????????????????????????????????????????????????????????????????????????????????????

**QUEST**_*Questa*

????????????????????????????????????????????????????????????????????????????????????????????????????????????????????????????????????????????????????????????????????????????????????????????????????????????????????????????????????????????????????????????????????????????????????????????????????????????????????????????????????????????????????????????????????????????????????????????????????????????????????????????????????????????????????????????????????????????????????????????????????????????????????????????????????????????????????????????????????????????????????????????????????????????????????????????????????????????????????????????????????????????????????????????????????????????????????????????????????????????????????????????????????????????????????????????????????????????????????????????????????????????????????????????????????????????????????????????????????????????????????????????????????????????????????????????????????????????????????????????????????????????????????????????????????????????????????????????????????????????????????????????????????????????????????????????????????????????????????????????????????????????????????????????????????????????????????????????????????????????????????????????????????????????????????????????????????????????????????????????????????????????????????????????????????????????????????????????????????????????????????????????????????????????????????????????????????????????????????????????TTTCCTCTAATGCTGGCAATGCAAACTTTTACACGGTGACGTTGATAGTCGTAGTCCTTAACGGAA?GTGCTACTTTTGCAACGAGAAGCTCCGACCTTCGGGGAAGAGCCAGAGACCAGACAACCGGGCCCCCTGCTGGCCTCGGGTCCAGCCCAGCCGGGGCATTTCAGTTCTCCTCTTCGATGGTATGCGATCTCTTACCATGGTTAAGGCCAAAACTACGCCCTGGCAAGGCAACGCCCGGGACTCTTTCGCCCGGGCTAAATCTCACTTTCATAATAGCTAGACCATCTCGGGTCCAGGCGACGTTCCCCTTACGGGGACTGGGACACGACCTACTGCCGGTTCCCGGGGGCTGACTAGTTCTGGGTGGCCGGCGCCTAATTACCAGAAGCTTCGCCCGAATACTCTAAGGTGCCTCGGTTCTATCTGTTCTCCGGAGCCGAGGTAAGGGAAGACGGTCGTTGCGGGCGTGCCCTCCAGAAAACAAATCGAATTTTAAGAACATCTGCACGACGACTACGATGCCGGGCTTG?GTACTGACCCCGCACCAGCTCCCGACAAGTCTTGGCCATAGTGATTAGAATCCCCCCGCCCATTGGCGGGGTTCTGGCACATTCGCCCTGTGAGCGAAACAAGTGCTTTCCACGGTTCCGCCAAGGAATGCGTTTGCGTTGGCCCAAGGCCGTTCGCTACCTCCTTGTACTGGGACTGTCCCACGCACAGGAGTAGCTCTTTTAGCGGTTCGTGACCCTCGCTTGCCCGGCG??????ACGGTTCTTGCGGCGGGGATTGTCGCTGTATTGCTA??????????????????AACATTTCCTTCAAAGGCCCTTGCGACACCGAAAAATAGACGCCTCTTACCAATAAATAACCTTTAGGCAGGCCTAAAAAATTTAAAGAGTATCAGCTTAATTACGCCCATACAAACAAT??????????CGTTTTTAGTGCGACTGAGGCAAAATCAACTTCCCCGTTAACACGAAAAATTCTGCCCGATCAT?AAAAGCTGTAAGCTATTTTCTCCCAGCCAACGAAGAGAAAGT????????????????????????????CGACAAACTGAGCTCGTGCGGAGACGACCGCCCGCGTCGGGGCGCCGGGAACGTGTCGGGACGACCGGCCTTGTCTCTGTCAGGCGTCCAGTCCTCCTGACGGGGCCTCACGCTCGCTGGGACGCCGGGCCGCCCCTCTGGGGTCCCAGCGCAGAGCAGCGTAACATCTGCGGGCACTCGGATT????????????????????????????????????????????????????????????????????????????????????????????????????????????????????????????????????????????????????????????????????????????????????????????????????????????????????????????????????????????????????????????????????????????????????????????????????????????????????????????????????????????????????????????????????????????????????????????????????????????????????????????????????????????????????????????????????????????????????????????????????????????????????????????????????????????????????????????????????????????????????????????????????????????????????????????????????????????????????????????????????????????????????????????????????????????????????????????????????????????????????????????????????????????????????????????????????????????????????????????????????????????????????????????????????????????????????????????????????????????????????????????????????????????????????????????????????????????????????????????????????????????????????????????????????????????????????????????????????????????????????????????????????????????????????????????????????????????????????????????????????????????????????????????????????????????????????????????????????????????????????????????????????????????????????????????????????????????????????????????????????????????????????????????????????????????????????????????????????????????????????????????????????????????????????????????????????????????????????????

**SABEL**_*Sabella-Schizobranchia*

TGTGTAAGCAGACTCAGAACATCCAAAATTCATTACCACCGGACAAAACATTCCAGGAAGAGACCACCTCACAATGAGCCTCCTCCAATCTCAAAAATAATAATAGTCGACACTGTCCAACCGGAGCGCACGGATTTTCCATCAAAGTGCACGCTCCTCCTACACGCAGTTTCTCAGCCGCAGAGAAAAAACAAAAGACCACATAAGGAATCTCGCCTGCCACCGTCAGATCACATTTCCCCAGATGTTTCACTTACATCTTACTATACGAACAATCTAGGCATACGAATAGAACGGGTTCCCATGTCATCTGTTATAATAGCTACCTCTCTCTTCCCGGTTAGGTCCACCATACACTCGTACCAACCGCACTTTGGGCAGAGCCGGCAATCACCCCAT??????????????????????????????????????????????????????????????????????????????????????????????????????????????????????????????????????????????????????????????????????????????????????????????????????????????????????????????????????????????????????????????????????????AGGGAAGTCCGCCGTCACGGGCGCTAAGTCCTTCGCACCGTGCCACCAGTTCTACGGCATCTTCCCCAGTCCGCTAAATTCTGTGATCTGCTGTACTTGTGCTCAAGAACAGCAAGCCCGCCCACTCCAGGGAGACATGAGGCGCCAGCATGAACCCAACAGCGAACAGAGTTGAGACAAGAGACCAGGCTCACGGATTCAACTAAGCTGCCCTTGTCCCTCGGCACACCGGGAACCATCCTCCAATGGGCTGTACAAGGGCTGGAAGACCGTTCGAGGCCCGCGCCATTGAGCCCCAAGAGCCAGACAAGGCCCAGCTCACGTCCACGGATTAAGCGTCTGGTTATCACGCCTATGGGCACCCGCCAGCGGAACACCTGCGCGCGTCACAGCCAGGACGAGCTCTCATCTTTCCGAAATGCCGGAACCGTCTAAACGTGTGGTGCAGCGAACCTTCCAAGGAAACCGCCAGCACAACGAGTCACTGCCCTGAGACCGTAAGCCGCCCGGGCCCTGCTCCTAGTGACGAGCCCTGGAGGTTACAACCATCTGTGAAGCTGAGACAATAGAGAAGGCTTCCACGCGCCACGTAAGAATCCCCACGCTAGCGTTGGCTTCTCT??????????????CCATCATATCAGAACGCCGGCAGGAGTCGGTCATGGCCAGGATTGCTCTGAGAGCGGAGCTGCGCGAGATGCGCTTGTCTACCGCGTCCGAACGAGCCACTCCGGCAGTGGTCTGGTCGTGCCCCTCCTGCT???????????????CATCCAGATTAGTCAGCCTACACGGTAATGTTAATATATGTTATTCTTAATCGTACATCCTACTCTTGTAGCATCAAGCTCCGACCTTCGGGGAAGAGCCTTAGACCAGACAATCGTCTGCTCTGCTGACTTTGGGTTCAGCTCTGCCGGAGTATTTCAGTTATCCTCGTCGATGGTACGCGATATCCTACCATGTTTAAGGCCAAAACTACGCCCTGACAGGAAAACATACGGGACTCTTTCGCCCGTATTAACTTTTCCTTTAAGTTTAGTTAGACTGTCTCGGGTCCGGACGGCGTCCCGTTCG?CGGGACTGCGTTCCGACCTCCTGCCGGTTCCCTTGGGCTCACTAGTCCTGGGTGGCCGGAACGTAATTATCAAAAGGCTTGCCTGAATATCGATAGGTGCCTCGGTTCTATTTGTTTTTCGGAACCGAGGTAAGGGAGGACGGTAGTTACGGTTTTGTCGCCTATGATGCTAGTCGAATTTTAAGAACATGTGCACGACAGCTAGGATTGCGGGATTGTTTCGTGACTCCGCCACACCTTCCGACAAGCTTTGGCCATAGTGATTAG?ATCCCC?CGCTTA?TGGTGGGGCTCTAGCATATTCGCGGTGACCGCGTAACAAGTGCGTTTCACAGTTTCGCCAAGGAATGCGTTGTTCTTGGCCCGAGGCTGTCCGTTACCTCCTTCTATTGGGCTTCTCCCATGGACAGGGATAGTCCTTTTGATGATTTGTGGCCCTCGTTGGTCCAGCGGGG????????CTTTGGTGTGAGGACGGCCA??????????????CGGATTCGCAGGCTAAAAATTTCCATTGGTGGCTATTGTGGTAACAGAGATTTATTTTCATT??CTACTGTATAGTAATTATGAAACCATAAATTACCTGAAAAATACTGGCTTAAAGATTCCCCC???????????????????CCTCTTTAGCACGACTAAAGCACAACTAAACCTCTTAGAATATTAACCTTGTCTGCCTGATCACTATAAGTCATAAGCTATTCTCCTTAGGCCAACGACAGGAGAGCGGCGCAGGGTTACCTTGTAACAACCC?????????CTAAGCCTATGCGGTGACGACCGCCCCAGTCGAG?CTGCGGGACCGTGTTAGGAGAGCCTCTGGTGTGTGCGACTGGCGTC?GGTCCTCCTGT?GGGGTTACAGACTCGTCACGCCCCGGTC?GTCGCATCTTGACTCCTCGCGTAGAGCAGTGTAACATCTGCTGACACTCGGATTAACAGCGTTTAGAGGCGGATAGGACGCAAAGTCTACCCGTGATTACTGGTTCGGCGGTGGTTCCG??GGATCCCTGCTCTG?TCGGCGGGCGCACTATCGCGGGGAGCCCACGACGGTTTCGGCCGTCAGAGCCCGGTGGAAGAGCCCTCCGGTAGGGTGTTATAGACACT?GGTGGTGGGCCGGTTGGAGGACCAGACT???TGCCGTCGAGGGGCGTGGGT??TGTGTCCGTTCGACTGGAGGACTGCGCATGCTCCGACCGCGGTCTTGCCTGGCCCTTTAGGGTCAGTGGCGTCGGTCG?CACCCTATCAAGCTAAATTGCGCATGGGTTCTACGAAACCTAAAGCATGAGTGAAGGCCTTCCTCTAGGGCTTAGTGGTCCTGCTTGCGGGCCCATGCCGCTCTATCGTAACCGATGAGGCGGACAACGTACACTTGGGTACCTATTTATAAGCTAGACTAGACAACTTGGTTAACTCCTGCGCTCTTCGAGTATCGTACCTGACGAACACATTAAGTCCGTGTTGATTACCGGGCGATCGAGTGCCGCCCTACCCCTACTACGAAGATCTCATAATAATTTGTGAATACAACGCCGATCCGTGTCACTAGGGTCGGGTAGGGCGTCAGACATAGGGGTGGCGTCGTGACGGGAGGCCCGTGGAACAGGCCTTGGCTAGCTGGGGCGCACGGTCATACGGATATGTTGAACGTAGATAGCGACGCGTTCCGAATCGGGGCAATGCCGTGCCTGTA?CCTATATCGTATCGACCGGACATGGAGATGGCCTCTCGGGGTCAAGCGTACAACGAACCAGTGCTCGGCGGTATCCCGGACTTGAGACG?ACTCTGTCGGCTTGCCGATGACGT?GTTCTCTAC?GCT?CCCGGGCGTGCACTG?CGTCGGCTAAACTGGGGGTGGTGTGATTTCATGCCGGACTGGAGCATCGATAACAGATTATCATCGAGAGGCTGTCATCTGGGGTACGAGCGGCCTGGAACTGGCAAGGGCTGGTGAGCCT???TGGGCGACTCGCCATCGTCGGGACCTTCCCGTGGAGTGCTCACTGGCCCCGGTCG?GTCGTGGGCTTCGGCTGGGTCTAAAGCCAATTTAACAAATGCTGCGTAGCTGGTGTGATTAAAATCCGGATCTAACGGAGAATCAGCTATCTGTCGACTCTTAGAGAATGAGAGCACGGGCAATTTTTTACGTTTCTTATTTATAAGCGGAGCATGGGTGAGCCCTTTGCTGGAACGTCGCCGTCG???CGGCGCGTCCCTCTAAAGTGTCAGCGGATAGAGCTTGAAGCAGGCGCTACACATCACTTTTTCGAAGTGTTAACTATTTATTAAGGATTATAACCTTTGATCGCCGTTAATCCCTCCCGTACATATATTCTTGCTATAACAATTGGATTAGGATTGAC

**SABLR**_*Gunnarea-Sabellaria*

AGGATAGGCACCTGCTGAACTGCGAATATACATTATTATAGGACATCCCATTAATGCAGACGACCACTTTGTTATGACCTTCATCGAATTTTCTATATAAGAATAATCGACATAGACTAATAGGAGCTCTTAGATTCATCTTTAAATTGCTCTCCCTTTCCATCCTCTCCACTTCTGCTGCAGAGAAAGTTTTTACGTTCCCTTAAGAAACTTAGCCTGCCCCCTTCAGACTTCTCCTCTTTAGAAATCGCCTCTAGAGCAGATTACTCTCGTATTCCAGGTATAAACCTAGAACAAATCCCCTTGTTATCAATTAAAATAGCAATCACTCTCTTCTCCGACAGGACTACCACTCTCACGAACTCATTACGTTTTCTAGAAGTCAACCTACCGTTATAT??????????????????????????????????????????????????????????????????????????????????????????????????????????????????????????????????????????????????????????????????????????????????????????????????????????????????????????????????????????????????????????????????????????AGGGCGGCCCATTGTCACGGGTGCTACTTCCTCTGCTTGATACTCAAAGTACTATGGCATCTTCTCCAATCCGCTTCATCTTACCATTCTCTGTACTAGCGTTCCAGAATTAAAAGCCCTCTCACCTGTGGGCCACATCTACCATCAGCATGAGACCAACAGCCAGGCCAATTGAAACACCAGAGTCCACATGCGGAATTAATTGAGAATTCATTGTCACTAGGCACACCTGGAGCCATCTGCAAATGGGATGTACAAAGGATGGAAAATTGTTGGAAGCTTATAACATTGCACCAATCCAGACAGACAAAGCCTCTCTCCCGTACGAGGATTAGGAAACTAGTTACTCTGCTTATGGTTACACGCAGCYAATACTCCTAAATGAGTCACTCAGCTGATCAACTGAATTCYTCCTGCAATGATCACACCGCAAAAATATGTGGAGTTCCAAATTCACCAAGGAAGCTGCCAACTCACTGAGCCACTGCTTACAGATGCTGCTATGCGAGTGTCCAGCCTTCAACTCTGAACGAAGGAGAATCTCTCGTTCGCCGAAGCTCAGACAACMAAATTCGCATCTTTGTGCCACGTCTTAATCCCCAGACCAGCGTAGGCCTCCAGATGACGCTTCTACGCTATTACACAAAAATGCCGCTCCGAGCACGCTTAGGATAGGAAACTCCTAAAAACGGAGCAGCTCCAGACCAGCTTATCTGCCGCGTTCGCACGAGCCTCTCCGGCAGCGGTCTGTTCTCATCCTCCCCGAA?????????????GAGCTGCTAATACAAGCCTAAACACGGTAATGTTAATAGTTGAGGTCCTTAATCGTACATCCTACTTTTATAACACCAAGCTCTGACCTCACGGGAAGAGCCTTAGACCAAACAATCGGGTCCCTGTCTGGCTTTGAGTTCAGCTTTGCCGGAATATTTCAACTCTCCTCTTCGATGGTACGTGATATCCTACCATGTTAAGGGCCAAAACTACGCCCTGACAGGAAAACAGAGGGGACTC?TACGCCCGCATTAACTTTTCCTTTAAGTTTAGTTAGACTGTCTCGGTCGTGGACAGTGTCCGCCTCACGGGTACTGCGCTCCGGACTCCTTCGGCTCGGGTTTCGCTAACTAGTGCCGGGTGGCCGGAACGTAATTATCAAAAGGCTAGCCTGAATAATGGTAGGTGCCTCGGTTCTATTTGTTTTTCTGAAACGAGGTAAGGGAAGACGGTCGTTGCGGCTTTGTCGCCCAGAATACTAATCGAATTTTAAAAACATGTGCACGACAGCTCGGATTCTCGGATTGTTGAATGACTCGACGACACCGTCCGACAAGCTTTTGCCATAGTGATTAG?ATTTCAGAACCTATTGGTGGGGCTCTAGCGCATCGGGTGA?TCATTCAGACTAGCGCGTTTCGCAGTTTCTCCAAGGAATGCGTTCTTCTTGGCCCGAGGTCGTCCGTTACCTCCTTCTATTGAGCCCATCTCATGGACAGGGATCGTCCTTTTGATGGTTTGTGATCCTCGTTGGCCCCGCCGTGTCGCTTGCCGTGTGGGCGAGGACGCTCACCTGAT?ATCA???CAGATTCGCGGGCCAAACATTTTCTCCAGAGACTGATGAGATAACGAAGGCTCAACAACTGATAAAGATAAATATTGTTCAGGAGAACCTGAATAGTAAAAAAAGTATTGGCTTTATTTTAACTTTAGAAC???TTAACAATATGAAAAATTGGCGCGGCCAAGGTTTATTTTTCTTCCTTAAA?GCTAGTATAAGTCAGCCAAGATCAAACAAGCCATAAGCTATCTTTCTTGAGTCAACGACAGAAAGGGGGCGCAGGATTTCCTTGTAACTTATC????????????????????????????????????????GCGGCAGTGGGAACGTGTTAGGAGAGCCTCTGTGGCATGCAGCGGACGTCCAGTCCTCCTGTTGGGGTTGTATACTTGTCACGCATCTGCTGTGTGTCGCTTGACTCCTTCCGTAGAGCTGTGTAACATCTGCTGGCACACGGATTAACAGCGTTTAGAGGTGGATGGGACGCATAGTCGACTGGCGGGTGCG?GCTGGGCAGGCGTAGTGGCGGATCTTAG?TCTGTTTTGTGCGTGCACTTCCGTCGGGAACCCACGACGGTTCGGTCCGTCAAAGCCTCTTGGAAGAGCTCTCGGTGCGAGTATTATAGCCAGGAAGGTGTTGGCCGGGCTCGGGACTAGATT???CCTTGCCGGGACAGATTGACCCTCTGTGCGTTCGACTGGTGGACTGCGCATACTCCGACCGCGGAGTGGTGGTGTTTCTTAGGGTCTGTGGCGCCAGTCGGCACCCTATCAAGCTAAATTGCACATTGGTG?TACGAAACCTAAAGCATGAGTAAAGACCTATCGTTCGGGTTTAGCAGTCCCGCCTCTGGGCCCGCGCCGCTCGACTTCCGTAAGTGAGGCGGACAACGTACACTTGGGTACCTATTTACAAGCTGGACTAGACGACTTGGTTAACTCCTGCGCTCAAATAGTATCGTACCTGCCGAACACATTAAGCGGGTGTTGATTATTTCGCAATAGAGTGCCGCCCTACTCCTGCCACGATGATCTCATAATAATTTGTGAATACAACGCCGATCCGTGTCACTAGGTTCGGGTAGGACCTCGGCTAACGGGGCATGTGGCCGAGGGGAGGCTCGGGGAACGGGTCTCGGCTAGCCGAGGCGCTCGGCCATACGGATATGTTGAACATAGATAGCGACGCGTTCTGAAGCGGGGCAATGCCGTGCCCGCAGTCGATATCGTATCGACTGGACACGGAGATGTCTCGTTCGGGTCTAGTGTACAACGAACCAGAGCCCGGCGGTGTCCCGGACTTGAGTCATACTCTGTCGGCTTGCCGATGACGTTGCAACCTACCGCTCCCCGGGCGTGCACCGGCGTCGGCTAATCTGGGGACGGTGTGATTTCGTGCCAGACTGGACCATCGATAACAGAT?ATCATCGAGAGACTGTCGTCTGGCTTGAGAGCAGCGTGGGACCGGCGGGGGCTGGTGAGACCGGTGGGTCGACCCGCTACAGCCGGGACCTGTTTGTCGACCGGTCACTGGCGGGTGTCGGCCATTTCGCTTCGGCCGGGCTCAAAGTCGATTTAACAAATGCTGCGTTGCTGGTGTGATTAAGATCCGGATCTAACGCAGAATTAGCTATCTGCCGACTCTTAGAGAATGAGAGCAGGCGCAATTTTTTACGTTTCTTATTTAAAAGCGGAGGGTGGGCAAGCCCTTGCCTGGAACTGGCCGCTCGGCGGCTGGCGTCCCTCTAAAGTGTCAGCGGATAGAGCTTGAAGCAGGCGCTACACATCACTTTTTCGAAGTGTTAACTATTTTTTAAGGATTATAACCCTTGATCGTTGTTAATCCCTCCCGTACATTTATTCTTGCCATCGCAATTGAATTGGGATTGAC

**SACCO**_*Saccocirrus*

????????????????????????????????????????????????????????????????????????????????????????????????????????????????????????????????????????????????????????????????????????????????????????????????????????????????????????????????????????????????????????????????????????????????????????????????????????????????????????????????????????????????????????????????????????????????????????????????????????????????????????????????????????????????????????????????????????????????????????????????????????????????????????????????????????????????????????????????????????????????????????????????????????????????????????????????????????????????????????????????????????????????????????????????????????????????????????????????????????????????????????????????????????????????????????????????????????????????????????????????????????????????????????????????????????????????????????????????????????????????????????????????????????????????????????????????????????????????????????????????????????????????????????????????????????????????????????????????????????????????????????????????????????????????????????????????????????????????????????????????????????????????????????????????????????????????????????????????????????????????????????????????????????????????????????????????????????????????????????????????????????????????????????????????????????????????????????????????????????????????????????????????????????????????????????????????????????????????????????CCTGTTTCCTCTTATGCTG?TAATACATACTTTTACACAGTAACGTTAATAGTTATGGTCCTTAATCGTTCATCCTACTTTTATAACCCTCAGCTCCGACCTTTAGGGAAGAGCCTTAGATCAAGCAATCGGTCCCTTTGCTGGCTTTGTGCTCAGCTCCGCCGGAGTATTTCAATTCTCCTCTTCGACGGTAAGTGCTATCTTACCGTGTTAAAGGTCAAAACTACGCCCTGGCAGGAAAACATACGGGACTCTTTCGCCCGTATTAACTTTTCCTTTAATTCTAGTTAGACTGTCTCGGGTCCAGGCTGCGTCCACCTCGCGGGCACTGCGTCCTGACCTCCTCCCAGTTCCCTTGGGCTGACTAGTTCTGGGGGGCTGGAACGTAATTATCAAAAGCGTTGCCTGAATAATGGTAGGTGCCTCGGTTCTATTTGTTTTTCGGAACCGAGGTAAGGGAAGACGGTCGTTACGGTTTTGTCGCCTAGAATACTAATCGAATTTTAAAAACATGTGCACAACAACTACGATCCCCGGATTGTTTCATGACTCGGCGGCAGCCTCCGACAAGTCTTGGCCATAGTGATTAGAATCCCCCCGCTTATTGGTGGGGCTCTAGCACATTCGAGATGTTCTCGTAACAAGTGCGTTTCACAGTTTCGCCAAGGAATGCGTTGTCACTGGTCCGAGGTCGTCCGTTATCTCCTTCTATTGGGCTTCTCCCATGGACAGGAATAGTTCTTTTAATGGTTTGTAATCCTCGTCGGTCCTTTAGTGGTCACACCCGCAGTGGTGAGGACGATTGCTTGATCATTAGGACACATTCGCGGGCCAAACATTTCCTTTGAGGGCTATTGTGACCACGAGAGTGACCCGCCCTTACCAAATAAAAAACCTGCAGGCGGGCCTGCATTAAATGAAAAATATCGGCTTAACTTAACCTCTATACCTTATTAAAACCAGATAAAGATTGTGCGACACAAGCAAACAAAACCTCTATCTAGACACGACTCGCGCTGCCCGATCAAACTAAGCCGTAAACTATCCTTTCTGAGCCATTGACGAAAGGGAGGCGCAGAACCCCCTTGTAACAAGTT??CAACAAACTAAGCTTATGTGGAGACGACCGCGCCTGCCGAGGTAGCGCGAACGTGTTAGGACAGCC?TTGAGGCTGCGTACCGGCGTCCAGTCCTCCTGTCGGGGCTTCATACTCGTTACGGTGCCGGTCGCGGTCCTCTTGGTCCTCGCGTGGAGCAGCGTAACATTTGCTGACACACGGATT????????????????????????????????????????????????????????????????????????????????????????????????????????????????????????????????????????????????????????????????????????????????????????????????????????????????????????????????????????????????????????????????????????????????????????????????????????????????????????????????????????????????????????????????????????????????????????????????????????????????????????????????????????????????????????????????????????????????????????????????????????????????????????????????????????????????????????????????????????????????????????????????????????????????????????????????????????????????????????????????????????????????????????????????????????????????????????????????????????????????????????????????????????????????????????????????????????????????????????????????????????????????????????????????????????????????????????????????????????????????????????????????????????????????????????????????????????????????????????????????????????????????????????????????????????????????????????????????????????????????????????????????????????????????????????????????????????????????????????????????????????????????????????????????????????????????????????????????????????????????????????????????????????????????????????????????????????????????????????????????????????????????????????????????????????????????????????????????????????????????????????????????????????????????????????????????????????????????????????

**SCALI**_*Scalibregma*

??????????????????????????????????????????????????????????????????????????????????????????????????????????????????????????????????????????????????????????????????????????????????????????????????????????????????????????????????????????????????????????????????????????????????????????????????????????????????????????????????????????????????????????????????????????????????????????????????????????????????????????????????????????????????????????????????????????????????????????????????????????????????????????????????????????????????????????????????????????????????????????????????????????????????????????????????????????????????????????????????????????????????????????????????????GCCACGGGAGCTGCTTTCTCTGCTCCGTGCTGCTAATATTACGACATCTTCTCTCTTCCGCTCATTTTTTCCACGTGCTGTGTTTACGTTCGAGAATGGCCCATTCTCCCACCTGAGGGCCATGTGAGCCGGCTCCACGAGACCCACAGTCAGGCTCCTCCAGACCAGAGACTCCACTCACGGACTTAATAGACACTTGTTTGTGCTTAGGCATGCCCTGAGGCAGCAAAACTGACCTGTTCAAGGAATGGAACAACCCTTGGAAGCTTGCAGCGTTGAACCAAGCACCAGTGGCAAGCCCTCCCTGAGGTCCATGGATTAGGCGACTCGTTATCCGGCTAATGACGACATGTAGTCAATGCACTTGAGCGGGTCACAGCCTGGACCAACTTCATACTTCCTGGAACGCTGGCGGCGCCTCAATAGCCAGTGCAGCGAATCCCGCAGAGAAGCAGAAAACTCACTGCGGCATCGCCTTGAGACCGTGCTGTGCCCACCCCCAGTCTTCAGCAAGGAGCTCTCGAGGATCCCTCGTTCTGAGAAACTTAGATAACAGAGAAGGGGTCTTTGTGCCATGAGTCAATCCTGGCGCT?????????????????????????????CTATCACGCCAGAGTGTCGTCCCGAGCCTGCTTAGGCTAGGCTCGTCCTAAGAACGATCTCGCGCCCCGCGAGCTTACCTGCTGTGCCTGCCCGAGCCCCTCGGGCAGCGGTCTGCACCCGCCCCCCCCGCT??????????CTTATGCTGGTAATCCATACTATCAAACGGTAATGTTAATAGTCAAGCTCCTTAATCGTACATCCTACTTTTGTAACAAGAAGCTCCGACCTGGAGGGAAGAGCCTTAGATCAAACAATCGAGTTCTTTGCTGACTTTGGGTTCAGCTCCGCCGGAGTATTTCAATTCTCCTCTTCGATGGTACGTGATATCCTACCATGTCAAAGGCCAAAACTACGCCCTGACAGGAAAACATATGAGCGTCTATCGCTCATATTAACTTTTCCTTTAAGTCTCGTTAGACTGTCTCGGGCGCCGGCGGCGTCCACCCTCCGGGTACTGCGCCCGGGCCTCCATCCGATCCGGTTTGGCTGGTTAGTCCCGTCTGGTCGGAACGTAATTATCAAAAGTCGAGCCTGAATAATGGTAGGTGCCTCGGTTCTATTTGTTTTTCGGAACGGAGGTAAGGGAAGACGGTCGTTACGGTTTTGTCGCCTAGAATACTAATCGAATTTTAAGACCATGTGCACGACAACTACGATCCCCGGTTTGTTCTTTGACTCGGCGGCAGCTCCCGACTGGTGTTGGCCATAGTGATTAGAATCCCCCCGCCTGTCGGTGGGGCTCTAGCATCTTCAACCTGCGGTTGTAAGAAGTGCGTTTCACAGTTTCGCCAAAGAATACGTTGTCTCTGGTCCGTGGTCGTCCGTTACCTCTTTCTATTGGGCTTGTCCCATGGACAGGGATAGTCCTTTTGATGGTTTGTGATCCTCGTTGGTCCCGCGCGGGTTCTCCTGGCGGTGGCGAGGACGATCACTTGATCATCAAAACAGATCCGCGGGCCAAACATTCCCCTCAAGGGATTTTGTGGTAACGTAAAAGACGCGCCATGCACTTATAAACAACCTTCAGGAGGGCCTGAAAGAAATAAAGAATATTGGCTTTATTAGACACTAACCCCTC?CAAAAAACAAATATAATTGGTGCGACCGAGGTTAAACCTACTTCCTTTAATTTAAGTACAAGTCAGGCCGACTCTACCAAGCCATAAGCTATCCTCCTTAAGTTACTGACAGGAGGGCGGCGCAAGGCACCTTAGTAACT????TTTCACAAACTAAGTCTATGCAGATACGACCGCCCTGTTTCGCGGACAGGGAACGCGTGGGGACATCCCTTGCTTGGTGTACTTGACGCTCGGTTCTCTTGTTGGGGTTGCAGATTTGTGACGGCGTTGTAGTCACCTGGCTGAGTCCCAGCGTGGAGCTGCGTAACATCTGCCGACACTCGGATTAGCAGCGTTTAGAGGCGGATGGCACGTGCAGTTGACCCGTGCATGCTGGGTTTGGAGAGT??????TGGATCCTCC?GGATTGCGACGAGTGCATTTGCGCGCGCGAACCACGACGCTTCCGGCGGCCAAAGCCCTGGGGAAGGCCTCCGGAGTAGAGTGTTATAGACCCGGTGGTATTGGCCCGCTGCCGGAGCAGTAC?CTTTTAGTCGGCGTCTGCCCGCGC?CTTTCAGTTCGACTGGTTCACTGTTCATGTTCCGACCGC?GA?GGTGCTCGGGCCATAAGGTCTGTGGCGTCCATCGGCAGGCTATCAAGCTAAATTGCGCATGGGTTCTACGAAACCCAAAATATGAGTGAAGGGCTGT?????AGTCCCAGTAGCCGCT??TAGGCGCCTACGCCGCCGAGATTCTGCAATCTGGGCGGACAACGTACACTTGGGTACTCATATATAAGCTAGTCTAGACAACTTGGTTAACTCCTGCGCTCAAAGAGTATCGTATCTATCGAACATATTAAGTCCGTGTTAATTACCGGGCGATCGAGTGCCGCCCTACGGCTATTACGAGGATCTCAAAATAATTTGTGAATACAACGCCGATCCGTGTCACCAGGGTCGGATAGCCCGTCGGACACCGGGCCTTCGTCGCGTCGGGAGGTCTGCGGATTAGGCCTTGGCTAGCTGGGGCGCGCGGTCATACGGATATGTTGAACGTAGATAGGAACTCGTTCTGAAGAGGGGCAATTTCT?GCCCGATTTCTATGTCGTATCGACCGGACACGGAGAGGGCCCTTCGGGGTCAAGTGTACAACGAACTAGAGCTCGGCGGAGTCCCGGACTTGAGACGGACTCTGTCGGCTTGCCGATGACGTTGTTTCCTACCGCTCCTCGGGCGTGCATCTCCGTCGGCTAATCTAGGGAAAGTGTGAGTTCGTGCCGGGCTGGACCAT?GATAACAGATTATCATCGAGAGGCTGTCGTCTGGGGCACAAGCAGACCGGTACTGGTGGGGGCTGGTGAGACCCGGGGGTCGACTCGCCGCAACCGGGACCTTCTTGTGGACTGCTCACTGGCGGCGCTGTATCTCACCGCTTCGGCCGGACTCAAAGTCAATTTAACAAATGCTGCGACGCCGGTGTGATTAAAACCCGGATCTAACGGAGAATCAGTTATCTGTTGACTCTTAGACAATGAGAGCGTGAGTCTTCTTTTACGTTTCTTATTTGCAAGCGGGGGCTTGTCGCGCCCACGCCTGGAATCATTCTGTCTGCAGAGTGCGCCCACCCAAAGTGTCAGCGGATAGCGCTTGAAGTAGGCACTACACATCACTTTTTCGAAGTGTTAACTATTTCTGAAGAATTATAACCCTTGATCGTCGTTAATCCCTCCCGTACATGCATTCTTGCTATAACAATTGGATTAGGATTGAC

**SCLER**_*Sclerolinum*

????????????????????????????????????????????????????????????????????????????????????????????????????????????????????????????????????????????????????????????????????????????????????????????????????????????????????????????????????????????????????????????????????????????????????????????????????????????????????????????????????????????????????????????????????????????????????????????????????????????????????????????????????????????????????????????????????????????????????????????????????????????????????????????????????????????????????????????????????????????????????????????????????????????????????????????????????????????????????????????????????????????????????????????????????????????????????????????????????????????????????????????????????????????????????????????????????????????????????????????????????????????????????????????????????????????????????????????????????????????????????????????????????????????????????????????????????????????????????????????????????????????????????????????????????????????????????????????????????????????????????????????????????????????????????????????????????????????????????????????????????????????????????????????????????????????????????????????????????????????????????????????????????????????????????????????????????????????????????????????????????????????????????????????????????????????????????????????????????????????????????????????????????????????????????????????????????????????????????????????????????????????????????????????????????GTTAATGGTCACTCTTATTGATCGTT?ATCCTACTTTTATAACAACCGGTTCCGACCGCCGGCGAAGAACCTTAGATCAAACAATCGGTTCCTTTGCTGGCATTGGGTTCAGCTCAGCCGGAGTATTTCAGTTCCCCTCTTCGATGGTACGTGATATCCTACCACGCTAAGGGCCAAAACTACGCCCTGGCAGGAAAACATACGGGTCTCTTTCGTCCGTATTAACTTTTCCTTTAATCTTAGTTAGACTGTCTGGGGTCCAGGCGGCGTCCACCGCGCGGGTACTGCGTCCTGACCTCCTGCCGGTTCCGTGGAGCTCGCCAGTTCTGGGTGGCCGGAACGTAATTATCAAAAGGCGCGCYTRAATAATGGTGGRTGCCTCGGTTCTGGTTGTTTTTYGGAACCGAGGCGAGGGAGGACGGTCGTTACGSCTTTGTCGCCTAGAATACAAATCGAACTTTAAAAACACGTGCACGATGATTACGATCCCCGGTTTGTTTCATGACTCGGCGGCAGCTTCCGACAAATCTTGACCATATTGATTAGAATCCCCCCGCTTATTGGTGGTGCTCTAGCACATCCACGTGGCGCGTGCGACAAGTGCGTTTCACAGTTCCGCCAAGGAATGCGTTGTTTCTGGTCCCAGGCCGTCCGTTACCTCCTTCTATTGGGCTTCTCCCATGGACAGGGACAGTCCTTTTAGCGATTTGTGATCCTCGTTGGACCCGCGGGGGCGACCGTCGCGGTGGCGAGGACGAGCACTTGACCGCTA???CAAATATGCGGGCCAAACATTTCCCCTAGGGGCTATTGAGACAACGAAAGCTTCCCGCCTGTAGTAACCAAATAATCTTCAAGAAAGCTTGAAAAAATTGTAGAGCGTTGGCTTAATTTTTAATAGATTATTTAATAATTCTTAATAAAATTAGTGTGACTAAGGTACCAAAAACTTCCTCTCCACCCAGCATTCTCCTGCCAGATCAAAATAAGCCACAAGCTATCTTTCTTAAGTTAATGTCAGAAAGGAGGCGCAGGGGCCCCTAGTAACTAACC??????????????????????????????????????????????????????????????????????????????????????????????????????????????????????????????????????????????????????????????????????????????????????????????????????????????????????????????????????????????????????????????????????????????????????????????????????????????????????????????????????????????????????????????????????????????????????????????????????????????????????????????????????????????????????????????????????????????????????????????????????????????????????????????????????????????????????????????????????????????????????????????????????????????????????????????????????????????????????????????????????????????????????????????????????????????????????????????????????????????????????????????????????????????????????????????????????????????????????????????????????????????????????????????????????????????????????????????????????????????????????????????????????????????????????????????????????????????????????????????????????????????????????????????????????????????????????????????????????????????????????????????????????????????????????????????????????????????????????????????????????????????????????????????????????????????????????????????????????????????????????????????????????????????????????????????????????????????????????????????????????????????????????????????????????????????????????????????????????????????????????????????????????????????????????????????????????????????????????????????????????????????????????????????????????????????????????????????????????????????????????????????????????????????????????????????????????????????????????????????????????????????????

**SERPU**_*Protula-Salmacina-Serpula*

????????????????????????????????????????????????????????????????????????????????????????????????????????????????????????????????????????????????????????????????????????????????????????????????????????????????????????????????????????????????????????????????????????????????????????????????????????????????????????????????????????????????????????????????????????????????????????????????????????????????????????????????????????????????????????????????????????????????????????????????????????????????????????????????????????????????????????????????????????????????????????????????????????????????????????????????????????????????????????????????????????????????????????????GTGGCCCGCCGTCGCGGGCGCCGAATCCTTCGCCCGGTGCGAAGGATACTATGATATTTCTCTTAATCCGCTAGGTCCTGCGACACTCCGTGTGTATATTATAGAATTGTCTGCTCGCGCTCATGTCGGCGACATGCGTTATCAACACGAGACCTACAGCGAGTCTCTTCAATGCAAGAAGAACCACCTACGGATCCAACCGATTTTGCGTTGTTTCCCGGCATACCTGGAACAGTCTGACAATGAAATGTGGAGTGGGACCAAGTTTGCTCGACGCTCGCAATATCGTGCCAAGAGCCCCTAATAGACGCGCTCTGAAGCCCGTGGCTTATGTCTCTCGTAACTACGCCTATGGCCACGTGGGGCCGCTCTTCACAGGTGCGCTACTACTTGGCTGAACTCTAAACCCTCTGCAACGCTGGGATCGTCTCAACTGCTGGTGCAGCGAACTCACCAAGGAAGCCGATCACTTAAATAGGCACTGTCCTGAGATCGCAAGTCGCTTTGTCCCAGGCTGCAATGTCGAGCGCTGGAGAATTTCTCGTTCGGCAAAGCTTAGACAATGGAGAAAGCATCTCTGCGCTATGTGACAAAACCGGTGGTAGTGTCGGCCTTTCCGACAC?????????CAGATAAGTAAGAGCCCCGAAAAGAGTTGGTTAACGACAGGAATGTCCTAAAAGTAGAGCCACGCCCCATATGCACTTCTGTAATGTCCTCTCGAGTAGTGCGAAAAGCGGCTTGAACGTGCGCCTACTGCC???TGTTCCTTTTATGCCGGTAATACACGCCTTTTTAAAGCAACGTTAATATATGTGATTCTTAATCGTACGTCCCCTCTTTGTAACAATAAGCTCAGACCTTCGGGGACGAGCCTCAGACCAGGCAACCTGTGAGCGTGCTGGTCATCAGTTCAACTTCGTCGGAGTATTTCAGCTATCCTCGTCGACGTTAGGATAGTCCCTATCGTGTTAAGGGCCACAATGACGCAGCGGCAGGAAAACATACGGGACTCTTTTGCCCGTATCAAATCTTCCTTTAGAT?TAGTCAGACTGTCTCGGGTATCGTCGGTGTCCTGTTCA?CGAGACTGC??GATGACCTACCGATGGCACTCCGAGGCCCGCTGGTCTCGAGTGGCCATTGCGTAATTATCAAAAGTTTTGCTCGAATAGTCGTAAGATTACCGGCACATTTTTT?CCTTTGTGATGGTATAAGGGATGCCGGTGGTGGCGGGTGTGCCCTCCCCGACAAAAATCGAATCTTAAAAACATGTGCACGACAGCTTGCTGTATGGGATTGATTTATGACTTCATTAAGCCCCCCGACAAGCGTCGGCCTTAGTGATTAGGATCCCCCCGCTCATTGGCGGAGTTCTGGCACATTCGCGGTGTGCGTAAAACAAGTGCGCTTCACAGTTCCGCCAAAGAATGAGGTCTATTTAACCCAAGGTCGCTCGTGACCTCTTTCTACTGGGCTTATCCCATGGATAGAGATCGTCTTTTTGATGGTTTGTGACACTCGCCGGCCCGCTGGGTGTCTTCGCAGTGGTGGCGGGGAAGTTCACTTGACTGTCA???????????????????????????????????????????????????????????????????????????????????????????????????????????????????????????????????????????????????????????????????????????????????????????????????????????????????????????????????????????????????????????????????????????????????????????????????????????TACGGGGACGCCTGGCCTGCAGTGTCTGTTAGGAATG?GTAGGAGTGTC?TCTGAGGCGTTCGTGTATCGTCTGGTTCTCTTGGCGAGGTTACATACTAGTAATACTTTGCA???CATCG?TCAGGCTCCTTGCGTAGAGCAGTGTAACATCTGCAGGCACTCAAATTACGGCCGTTTACAGGTGGAAGGGACGCAAAGTCGGCTTCGGGGTATTATCCTCTGGCGTGTGCGGCTGGTTC??????TTCGT?GG???ACGCATTTCCCGTTGGAGCCCACAACGGTTTCGACCATCAAGGTACCGGACAAGAATCATGTCAGTGGAACTTATAGGTCCG???TGGAGTTGCTGCTGGTTGAGCAGACT????CGGGCCGTGCCCTG???CCCCC???????GTCGACTGGGGGACTGCGCATGCCTGTCCGACCGGGGCAGATCAGCTAGCAAGGGTTCGTGGCTATGTCGGCACCCCATCAAACTAAATTGCACAAAGGACTACCGAATCCCCAGGCATATGTAAAAAGGTGT?????GGGGCAACT?????????????GGCGCACCCCGCTCGTGACCTGTGTCTGAGGCGGAGTTCGTACATTTAGGTACCTATTTACAAGCTGGACTAGACGACTTGGTTAACTCCTGCACTCGGGGAGTACCGTACATGTCGAATACATTAGGTCCGTGTTAATTATCGGGCGCTCGAGAGGAGCCCTACTCGTACCACAT?GTTCTGATAATAATTTGTGAATACAACGCCGATCCGTGTCACTAGTGACGGGTACGACGCCGGGCATCGGGACGGTCCCTCGGTGGGAGGTCTGTGGACTTGGCCTTGGCTAGCCGGGGCGCACGGTCATACGGGTATGTTGAACGCAGATAGCGACGCGTTCTTAATCGTGGGGATGAACGCCACGGC??CTATATCGTATCGACCGGACGCCGAGATGTTCCTTCGGGAACCAGTGTACAACGAACCAGAGCCTGGCGGCACCGTGGACTTGAGACGGACGCTGTAGGCTTGACTATGATGTTGA?TCCTACCGCCCCCCGGGCCTGCGCTGTCGTCGGCTAATCTGGGGATAGTGTTATTTGGCGCCGGACTGGACCATTAATATCAGATTCTCATCGCGACGTCGCGATCTGGGCTGCAAGCCTCGTGGTACTCGTCTGGGCTGGTGAGCTTTGGCCGGCAACCCGCCGTGG?CGGGCGCTTTCGGTGGAGTGGTCACTAGTCCACGTCGCGCGCTGGATTTCGTCTCGGTGCAAGACGAATTTGACAAATGCTGCTTAG?GGGTGTGATTAAAATCCGGATCTAACGCAAAATTAGCTATCTGCTGACTCTTAGAGAACGAAAGCGCGGGCAATTTTCCGCGTTTCTTATCTATAAGCGGAAGAAGTCCATTTACAGCACTAGAATGAGCGCCTCG?GTGCTCGCGTCCCTCTAGAGTGTCAGCGGATAGCGCTTGAAGTAGGCACTACACATCACTTTTTCGAAGTGTTAACTATTGGTCAAGGATTATAACCTTTGATCGTTGATAATGCCTCCCTTACATTTACTTTTCCTACAAGAATTGAATTAGGATTGAC

**SIGAL**_*Sthenalanella*

????????????????GAACCTCTAAGCTATATAGCTACTGGGCAAAGCTCTACCGGAAGAGATCGCCCCACTACGGTCTCCTTCTGATTTCTTACATTCTCATAATTGATACTATCCAACGGGGGCTCTCGGGTTTCCTACTAAAATACTCCCACCTCCTATTCTCACTTCATCAAGGCTTGTGAAAAAGAGAAAGTTTTCTGCTCTAATATTGCCTGCAACCATCTGGCCTCCTCCCCCACGTGGTATCGTTCAGCTCTCATCATACATGTAATCCAGGCCTTCGTCTTGAACGTGTGCATATGTGGGCCGCTAAAATAACTACTACACCCAAGACAGCCACGTCTATTACCCACCCGAATTAATAGCATTTCCTGCGGTAC?????????????????????????????????????????????????????????????????????????????????????????????????????????????????????????????????????????????????????????????????????????????????????????????????????????????????????????????????????????????????????????????????????????????????????????AAGGTGGCCCACCATCACGGGTGCTACTTCCTTCGCTCCGCGCCGCCGGTACTACGGCATCTTCTATCTTCCGCAAAATTTTCCCACATGCTGTGTTTGCGTTCCAGAGCCGTTTACCCGCCCACCCGTGGGACACACGTGGCGGCTCCACGAGACCCGCAGCGAGAAACTTCTCAACGTAAGGCACCAGCCACGGACTCAACCGACCTTTCATCGTGCCCAGGCATATCTGGAGGCTTCCGCCAATGGGCTGTACAAGGGCTTCACCACCCCTCGATGCCCCCCTGATCGAGCCACAAGCCACCGACAAGCCCCCTCTGACGTCCATGGTTTAGCACTCTCGTAAGCACACTCATGGTTACCCGCCGCCAACCGACCTGCGTGAGCCACAGCCCTGTCGAGCCGTCTTCTTCCCGCAACGGTAGCACCGTCTAAACATGTGGCGCAGCGTCTCCACCAAGGAGGCTGGAGACTCAAGGTGCCACCTCCCAACGACCAGGCTACGCCACGCCCCAGTCTTCAGCAAGGAGCCGTCGAGGATCTCTCGTTCAGAGAAGCTCGGACAACACAGTCCGCGTCTACGAGCCACGCGAGAGTCCACAGGCACCCGTGGGCTTCTCCGAGACCCACCCCCG????????????????????????????????????????????????????????????????????????????????????????????????????????????????????????????????????TCTGTTTCCTCTTATGCTGGTAATACAGGCTGTACTATGGTAATGTTGATAGTTATGGTTATTGATTGTACACCTTACTTTTGCAATCGAAATGTCCGACTCTCGGGGAGGACTCTTAGATCAGACAATCGGGTCCCGTGCTGACTTCTGGCTCAGCTCAGCCGGAGCATTTTAATTCTACTTGTCGATGGTACGTGATATCCTACCATAGTAAAGGCCAAAACTACGCCCCGACAGGAAAACATACGGGACTCTTTCGCCCGTATTAACTTTTCCTTTAAGTCTAGTTAGACTGTCTCGGATCCGGGCGGCGTCCGCCTCGCGGGTACTGCTCCCGGTCTCCCCTCTGGTACCGTCGGGCTCACTAGTCCGCGGCGGTCAGGACGTGATTACTCAAAGGCGTGCCCGAATAATGGTAGGTGCCTCGGTTCTATTTGTTTTTCGGAACCGAGGTAAGGGAGGCCGGTCGTTGCGGTTTTGTCGCCCAGGATACTAATCGCATTTTAAAAACATCTGCACGACGACCACGATCCCCAGATTGTTTCATGACCCGGCGAAAGCTTCCGACAAGTCTTGGCCATAGTGACAGTACTCCTTCCGCCTCTTGGCGGGGCTCTAGCGCCTCCGTCGTGCCGGCGCGACAAGTGCTTTCCACGGTTTCACCAAGGAATACGTTCACGCTGGTCCGAGGCCGTCCGTTAACTCCTTCTATTGGGCCTCTCCCATGGACAGGGTTTTACCTTTTAATGGTTTGTGATCCTCGCCGGCCCCGCGG???TTTCCGTCGCGCGGGCGGGGACGATCGCTTGATCATTAAAA??????????????????????????????????????????????????????????????????????????????????????????????????????????????????????????????????????????????????????????????????????????????????????????????????????????????????????????????????????????????????????????????????????????????????????CCCCACAAACAAAGCCTATGCGGAGACGACCGCCCCTGTCGAGGCAGTGGGACCGTGTCAGGACAGCCTTTGGGGTTGCGCTGTGGCGTCCGGTCCTCCTGTCGGGGCTTCATACTCGTCACGGCGCGCGGCGGAGTCTCTTGAGTCCTAGCGTGGAGCAGTGTAACATCTGCCGACACTCGGATTAACAGCGCTCAGAGGCTGATGGGACGCAAAGTCGCCTCGCGGATACT?GCCCGTGCCGGGTGTGTCTCGATCCGTCGCCGGGCCGGCGGGCGCCCTTCCGCGAGGAGCCCACGACGGTCCCGGCGGCCAAAGTCCGGGGAAAGGACGTGTCGCCCGCGTGTTACAGTCCCTGGTTCGGAGGCCCGCCGTGGAACCAGAAACGCTTCCGCCTCGGGGGGGCTGCCCGTCGCGCGGGCGTCGAGTGGACTGTACATGTGCCGACCGCCG?CGCTGCGGGTCGCTCAGGGTCCGTGGCGTCGATCGGCACCCTATAAAGCTAAATTCTCCGTTGGCACTACGAAACCCGCAGCATGAGTGAAGGGCGTTCTC??CGTCCGAGCAGTCCGTCTCGGCGGCCTGCGCCGCTCGATCGCCGTCGATGAGGCGGACTACAAACACTTGGGTACTTATACATAGGCCAGTCTAGACGACTTGGTTAACTCCTGTGCTCATTGAGCATCGTACCTGACGAACTCATTGAGTCGGTGTTTTTTATTGGACCATCGAGTGCCGCCCTACTGGTACCGCGGTGCTCTGACCCGGAACATTCGGATTCAATTTAAGCATGACGGCTTGCGGAGGGCACCACGTCGAGCATAGCCGGACCGCTGCGACGGGAGGTTCGCGGAACAGGCCTCGGCCAGCCGGGGCGCGCGATCATACGGATGCGTTGAACGTAGATAGAGACTCGTTCCGAAACGGGGATATGACCATCCCGTACTCTATATCGTATCGACGGGCCACGGAGATGTCTCTCCGGGAGTCAGTGTACAGCGAACCAGTGCTCGGCGGGATCCCGGACTTGAGGCGGACTATGTCGGCTTGCCGATTACGTTGTACCCTACCGCTCCCCGGGCGAGCGCTCCCGTTGGCTAAACTGGGGACGGTGCGATTTCGTGCCCGACTGGACCGC?GATAACAGATTGTCATCGCGAGGCTGCCGTCTGGGGTGCGAGCGGCCTGGAACCGGCAGGGGCTGGCGAGACGGCGTCGTCGATTCGCCGTCGCCGGGACCTTCCCGTGGACCGCTCACTGGCGGCGTTCGGCGCATCCGCTTCGTCCGGGTCCAAAGCCGATTTAACGAATGCTGCGTAGCTGGTGTGATTAAAATCCGGATCTAACGTAGAATCAGCTATCTGCCGACTTTTAGAGAATAGGAGCGCGGGAAATTTTTTAAGTTTCTTATTTAGAAGCGGAGAGCGGGCAAGGCCTCCTCTGGAACGCCCGCCTGGGCCGGGCGCGTCCCTCTAAAGTGTCAGCGGATAGAGCTTGAAGCAAGTGCTACACATCACCTCTTCGAGGTGTTAACTAATTATTAAGGATTATAACCTTTGATCGTTGTTAATCCCTCCCGTGCATTCATTCTTGCTACGACAATTGCATCGGGATTGGC

**SIPUN**_*Phascolopsis*

TGAATTAGCAGACATAGCACCTCAAAACTCCTTTGCTACAGGTCAAATCATTACAGGAAGAGACCTTACTGTAATGTACCCCTTTCTATCTCAAATGTTCTTATAGTCGGCACCCTTCTATAGGGGCCCTTGTATTCAACATTAAAACACTTACTCAGTCAATTCTCAGATCCTCTGCAGCAGAGAAAAAATACAAGCCCTCTGTAATGCAATCGCTCGCACCCGTCTGACCACTTTTCTTTAGTTGTCAGATTCTCATCCCTCTATACTAGTATATTAGGTTTCTGAATATTCCGAGTCCATATGTAAGCAGTAATATAAACTACCTCTTACAGCCCAGTCTTGCCAACAACTCACATGTATCAATTTGTTTTCCAACACAGCAACCCTCAACCA?????????????????????????????????????????????????????????????????????????????????????????????????????????????????????????????????????????????????????????????????????????????????????????????????????????????????????????????????????????????????????????????????????????????CCGGCAGTTCGTCGTCGTAGGTACTACTTCCTCTGCCTGGCGCCACAAGTACTACACCATCTTTTATCTTCCGCAAAAACTAGTGATGTGCCGTGTAAATATTTAAGAATTAAAAACTCGCGTACTCGTAGACCACATGTGACGGTTCCACGAGCCCAACAGTCAGGCTCTTTGAGACAAGAGAGACCCAGTGGGGATGCAATAGAAGAATGATTGTGCTTTGGCATTCCTGGAGCAAAGCCAAAATGTCCTGTTCAAGGGATGGAAGAACCTTTGAGGCCCTCAGCATTGACCCAACAAAAACCGATAAGCACGCTCTGATGTGCGAGGATTAGGACACTCGCAACACTGCTTATGGTGAAGTGCCAAGAACACACCTAAGTGGATCACAGCTTGGACGAACTCTTTTTCACCCACAACGGTGGAACAGACTAAATATGTGGAGCAGCGAAAACCCTATGGAAGCCAAGGATTCAAGGCGTCACTGCTTAGAGACCGTGCCCTGCTATGGCCCAGTCCCTAACAATACCCTCTTGAGGATTCAACGTTCTGAGAAGTTGAGACAACCCAAATTGCGTCTACGCGCTATGAAACAATTCCTCTGCAAGTGTGAGCATCTCAGAATTCCTACGACGCTATCACGCCAGGGCTCCGCTCTAAGTACGCTTAGACTCTGAAAACGCTGCTCTCGGAGCTGCTCCAGACGCGCTCGCCTGTAGCGTCCCCCCGAGCTTCTCGGACAGTGGCCCGCACCTATCCTCCCTACT??????????CTTATGCTGGTAACACACGCTCTAGCACAGTAGTGTTGATGGCTACTATTATTAATCATA?CTGCTACATTTGCAACAATATGCTCTGACCGG?GGGGAAGAGCAGTAGATCAAACAACCGGCGTCATTGCAGACTTTGGTTTCAGCGAAGCTGGAGTTTTTCAATTCCCCTGTTCGTATGTCGGCGCAATCCCACATACTTTGAGGCCAAAACTACACCCTGACAGGAAAACATACGGGACTCTTACGCCCGTATTAACTTTTCCTTTAAGTATAGTTAGACTGTCTCGGGAGCCGGCGGCGTTCGCCTCGCGGGCACTGCGTTCCGGCCCCCTGCCGGTACCGTGTGGCCGGTTGGTCACCGGCGGCCGGGACGTAATTACCAGAAGGTT?GCCTGAATAATGCTAGATGCCTCGGTTCTATTTGTTTTTCTGAACAGAGGTAAGGGAAGACGGTCGTTACGGTTTTGTCGCCTAGAAGACTAATCGAATTTTAAAAACATGTGCACGACAACTACGATCGCGGGATTGTTTTTTGACTCCGCCGCAGCTTCCGACAAGTCTTGGCCATAGTGATTAGAATCCCCCCGCTTATTAGTAGGGCTCTAGCGCATAAGCCCGCAGGGCGGGACCTTTTGTGGAGATTTTGCCGTAAATGCATGCGTT?TTTTTCGCCCAAGGTCGTCCACTACCGCATTGTATCTGGATTATCCAGTGGACAGGGATCGTCCTTTTGAAGGTTTGTGGCACTCGCTGCTCTCGCGGGGGCGACCGCCGCTGC?TGGGGGAAGGCCACTTGACTTTCA??????????????????????????????????????????????????????????????????????????????????????????????????????????????????????????????????????????????????????????????????????????????????????????????????????????????????????????????????????????????????????????????????????????????????????????CCCACAAACCAAGCCTATGCGGAGACGGCTGCCCCCGTCCGGGCGAGGGGCACGCGTAGGGACGTGCTCCGCGGCGTACACCGGCGGCCTAGTCCTCCTGTCGGGGATACAGAATCGCGTTGGCCGCTTCGGGGTCGCCGCTGGTCCCAGCGTGGTGCAGTGTAACACCTGCTGGCACACGCATTAACAGCGTTCAGAGCGGGACGGGACGCAAAGTCGACGCG?GGGTACCGAGCCGCGGCAGCGTGATACGGACC????GGGCCGC?CCGCGGTGCACTCCCCGGTCGAGCCTGGGACGGCTGAGGCGGCTAACGGCC??GGGGAGAGTTGTGGTGAGATGTCTGACACCCCGT???GTCCGAGCCCGCCACCTGGCTAGATGCGTGCCCGCCCGCCCGGGCGCTTCTACGCCTCGGTGTTGCCGGGGACTGCACGTGCCTGGCCCGGGGCGGGCTCCCGGC????GGGGTCACCGGCGCCGATCGGCCACCCGTCAAGGCTGAACGCGCAATGGCTGTACGAACCCCAAAGCATGAGTGAAGGGCCG??CCCTGGCCCGAGTGGTCCCGCTTCCTGGCCCACGCCACTCGGCCCCTGCCCGCGAGGTGGACAGCGTGCTTCAGCGTACTTGCGTACAAGCTGGCCCAGACTGCCTGACTAGCGCCTGCATCCT?CGAGTATCGTACACGACGAAAACGTTAAGCCCGCGTTTTCTGCCGGGCGGTCTGATGCTATTCTACCCCTACTACATTGATCTCTCAAGAATTTGTGAATACAACGCCGATCCGTGTCACCAGGTTCAGGTAGGGCGCCGGGCAGTGGCGCGCCGCCACGGTGGGCG?GCCGCGGAGCAGGCCTGGCCCAGGCGGGGCGCGCGGTCATACGGATACGTTGAACGTATGCAGGCACCCGTTGGGGATAGGGCCAACGTTCTGGCCCGCCTGTATATCGTATCGACTGGACACGGAGACGTG?CCCTCGGGCCCAGCGCACAACGCACCAGTGCCTCGCCGGGCCCCCGACTTGAGGCGAACGCTGTCGAATCGCTGATGCCACCGCTCCCTAGCGGCCTTCGCGGGTGCGCCCCGGCGGGCTAAACTGGGAGCGGTGTGATTTCGTGCCAGACTGGACCGTTGGTACCAGACTGTCATCGCGGGACCGCTGTCTGGTGCGCGAGCAGCCTGGGCTGGGCCAGGGCTGGCGCGGCCTAACGGTTGACCCGACGGTGCCCAGCGCTTCCAGTGGACCGCCCACTGGCGGCGTC??GCCTCGCCTCTTCGGCCAGGCTCAAGGTCGATCCAACAAAAGCTGGGCCGATCCTGTGTTTAAAATCCGGATCCAACGCACAAACGGCTATCTGCGGATGCTTGGGGGATGTGAGCGGCCGCAAGCTCGCACGTTCTCCATCCGCGGGCGGAACGCGGGCTCCGCCACTCCTGGAACCACGCGGTCG??CGGTGGTGTCCCTCCGGAACATCCGTGGATAGGGCTTGAAGCAGACGCTACACTTCACTTTTCCCAAGTGGCAACTCATAATTAAGGGTCATAACCTTCGTTCGTCGTTCATCCCTCCCGTACATTCGTCCTTGTCATCGCAATCGGACTAGGATCGG?

**SIPUN**_*Phascolosoma*

????????????????GAACATCAAAACTCCACAGCAATAGGGCGAATCTCTTCAGGCAGAGATCGCACTGTTATGACCTCCTTCCAGTCTCTCGAGTTCTCACACTCGATATAACTCAAATGGCGCTCACAGCTTCCATACTAGACTATATGCTCGGCTATTACCCTCCGCCTCGAGAGCAGAGAAAAAAAAAACGCCCTCTTCGGCGCCTTAGCTTGCCGGCCTCAGATCCCTTCTCCTCAGCAGTAAGTTTCAGAGCCCATTATTCTAGTAACTTAAAACTATCTTGAGAGCGAATTCTTACGTCAGCAGCATTCATTGTTATCTCATATAGCATAGCCACGACAACCACCTACACGGATCAACGGCCCTTTC????????????????????????????????????????????????????????????????????????????????????????????????????????????????????????????????????????????????????????????????????????????????????????????????????????????????????????????????????????????????????????????????????????????????????????????????????????????????????????????????????????????????????????????????????????????????????????????????????????????????????????????????????????????????????????????????????????????????????????????????????????????????????????????????????????????????????????????????????????????????????????????????????????????????????????????????????????????????????????????????????????????????????????????????????????????????????????????????????????????????????????????????????????????????????????????????????????????????????????????????????????????????????????????????????????????????????????????????????????????????????????????????????????????????????????????CCGCCAAGTAAAGGTGCCATACCATCTCAGCTTATGCTCTGTCACCCCTGAGCTCGGAGCCGCGCGCCGTGCGCTCTCAGATCGCGCCCCCACGAGCACCCCGGAGTCCGGTTCCCCCGTGCACCCCCTGCT?CTGTTTYCTCTTATGCTGCTAACACAGGCCTAAACACGGTAGTGTTAAAGGCTATGATTATTGACAATACTAGTTACTTTTGTAACAACCGGCTCCGACCCCGCAGGAAGAGCAGTAGATCGTATAAACCAACCCATTGATGGCTTTGGTTTCAGCCTCGCCGGAGTATTGCAGTTCCCCTCCGAGTCGGTAGGCGCCTTCCTACCGAGGTTAAGGCCAAAACTACGCCCTGACAGGAAAGCATACGGGACTCTTACGCCCGTATTAACTTTTCCTTTAAGTTTAGTTAGACTGTCTCGGGCGGAGACGGCGTCCTCCCTTC??GCACTGCCCTCCGGCCCCCTGCCGGTCCCATCGGGCTAACTAGTCCTGGGCGTCCGGGACGTAATTACCAAGAGTTGGGCCTGTATAATGCTAGGTGCCTCGGTTCTATTTGTTTACCGGAACCGAGGTAAGGGATGCCGGTCGTTACGGCTGTGCCGCCTAGGATACTAATCGAATTTTAAAAACAAGTGCACGACAACTACGATCGCGGGCTTGCTCGACGACCTTGCCGCAGCTTCCGACAAGTCTTGGCCATAGTGATTAGAATCCCCTCGCTTATTGGTAGGGCTCGAACACCTAGGACCTGGGGACATAACAGGTCATGGAGACAGTTCCGCCACAACATGCAGTGTCCATGGCCCAGGGTCGACCTTTACCGTTGTGCATCGGGTTTATCCCGTGGACAGGGACTGTCCTTTTGAAGGTTTGTGGCCATTGTTGGGCCGCC??GGGCAACCACTTTGGCGCTGAGGAAGGCCGCTTGACTTTCAGAACGGATTCGCGGGCCTGACATTTCCCTTGTGGGCTTTTGTGTTAGCAAGAGCTCGCCGCCAGAAGCATTTAAATAACCTTTAGGCGGGCCTAAATCTAGTAAAAAGTATCGGCTTTAATCTACTTTTATAA???????????????AAGATTTTATGCAATATAGGCAAACAAAGCTTCCTAAAAACCACAAGTACTTTAGACATTATTCAAATAGCCGTAAACTATCCCCCTTGAGTCACTGACAGGGGGGCGGCGCAAGATAACCTAGTAACTTATC???????????????????????????????????????????????????????????????????????????????????????????????????????????????????????????????????????????????????????????????????????????????????????????????????????????????????????????????????????????????????????????????????????????????????????????????????????????????????????????????????????????????????????????????????????????????????????????????????????????????????????????????????????????????????????????????????????????????????????????????????????????????????AAGCTAAATTGCGCAGTGGCTCT?A?CGAAACCAAAGATGAGTGAAGGCACCCGCGAGTCCCCGAGTGGTCCCCCCCTGGGGCCCACGCCGCTCGCCCCGCGTCCGGGAGGCGGAAGTCGTACACTTGGGTACCTACGCACAAGCTGGTCTAGACAACTTGGTTGA?????????????????????????????????????????????????????????????????????????????????????????????????????????????????????????????????????????????????????????????????????????????????????????????????????????????????????????????????????????????????????????????????????????????????????????????????????????????????????????????????????????????????????????????????????????????????????????????????????????????????????????????????????????????????????????????????????????????????????????????????????????????????????????????????????????????????????????????????????????????????????????????????????????????????????????????????????????????????????????????????????????????????????????????????????????????????????????????????????????????????????????????????????????????????????????????????????????????????????????????????????????????????????????????????????????????????????????????????????????????????????????????????????????????????????????????????????????????????????????

**SPHAE**_*Sphaerodoropsis*

??????????????????????????????????????????????????????????????????????????????????????????????????????????????????????????????????????????????????????????????????????????????????????????????????????????????????????????????????????????????????????????????????????????????????????????????????????????????????????????????????????????????????????????????????????????????????????????????????????????????????????????????????????????????????????????????????????????????????????????????????????????????????????????????????????????????????????????????????????????????????????????????????????????????????????????????????????????????????????????????????????????????????????????????????????????????????????????????????????????????????????????????????????????????????????????????????????????????????????????????????????????????????????????????????????????????????????????????????????????????????????????????????????????????????????????????????????????????????????????????????????????????????????????????????????????????????????????????????????????????????????????????????????????????????????????????????????????????????????????????????????????????????????????????????????????????????????????????????????????????????????????????????????????????????????????????????????????????????????????????????????????????????????????????????????????????????????????????????????????????????????????????????????????????????????????????????????????????????????????????????CTTATGCTGCTAATGCAAGCCCATGTACAGTAACGTTAATGGTTATGGTCCTTGATGGTAACCCCTAATTTTGAAACAACAAGCTCCGACCGCGGGGGAGGGGTCTTTGATCAAACAACGTGGGCGCTTGCTGGTTTCT?GGTCAGCTCAGCCGGAGCTTTTGAATTCCACCGGCCGTTGGTAAGCGCCATCCTACCAACCTAAAGGCCAAAACTACACCCTAGAGTGAAAGCATACGGGACTCGTTCGCCCGTATTAACTTTTCCTTTAAGTTTAGTTGGACTGTCTCGGCTCGCGGCGCCCTTCGCCTAACGGGTTCGGGGTCCCG??CTGCAGCCGGTACCGGGGGGCCCGCTGGCCCCCGGCGGCCGGCACGTGATTATCAAAAGCGCTGCCTGAACGGTGGTAGGTGCCTCGGTTCTATTTGTCTTTCGGAAACGAGGTAAGGGAGGACGGTCGCTACGGTCCTACCACCTAGCATACTAATCGGATTTTAAAGACATCTGCACGATGACTACGGTGCCCGGCTCGTTTCTTGACCCGGCGTCAGTGCTCGACAAGTCTTGACCATAGTGATTGGGCTTCCATCGTCTGCTGTTAAGCCTCCAACACTGCTAGCTGGTTGACCTAGCCGGCGCGTCTTGCAAAGCCTTAAAGGAATGCGGA???CATAGTCCAGGGCCGTACGATACCTCCTTGTTCTAGGCCTC?CCTACGGACAGGGGGAGCGCTTTTAGTGGCTCGTRGCTCTCSTCGGCGTCCCSGGGGCAACCGA??SGGGAACGAARA????????????????????????????????????????????????????????????????????????????????????????????????????????????????????????????????????????????????????????????????????????????????????????????????????????????????????????????????????????????????????????????????????????????????????????????????????????????????????????????????????????????????????????????????????????????????????????????????????????????????????????????????????????????????????????????????????????????????????????????????????????????????????????????????????????????????????????????????????????????????????????????????????????????????????????????????????????????????????????????????????????????????????????????????????????????????????????????????????????????????????????????????????????????????????????????????????????????????????????????????????????????????????????????????????????????????????????????????????????????????????????????????????????????????????????????????????????????????????????????????????????????????????????????????????????????????????????????????????????????????????????????????????????????????????????????????????????????????????????????????????????????????????????????????????????????????????????????????????????????????????????????????????????????????????????????????????????????????????????????????????????????????????????????????????????????????????????????????????????????????????????????????????????????????????????????????????????????????????????????????????????????????????????????????????????????????????????????????????????????????????????????????????????????????????????????????????????????????????????????????????????????????????????????????????????????????????????????????????????????????????????????????????????????????????????????????????????????????????????????????????????????????????????????????????????????????????????????????????????????????????????????????????????????????????????????????????????????????????????????

**SPION**_*Marenzelleria-Polydora*

????????CAGACTTAGAACATCAAAACTTCTTAGCAACTGGACAACTCATTGCTGGCAGAGACCACCTCACTATGTACTCCTTCTAATCTCTAGTACCTTCATGCTTGGTACTCTTCTACCGGAGCGCATGGATTTCTCTTTAAAACACTCTCTCATACTACCCCCGGTTCATCTGCAGCAGTGAAAAAAAAAACGATTTCAGAGGCAACCTTGCTTGCCACCTTCTGATCTCTTTTCTTCTGAGGTTTCAATCTGTGCCCTCTATACCAGTTATTTGGGCCTACGACTAGAACGTATCCTTACGTCAGCTGTAGTTATAGCGGTCTCTCTCATCTCTGTCAGGGCTATAGCTCTCACGTATCAACATCACTCCCAGCAGAGCTATCGATCACCACA???????????????????????????????????????????????????????????????????????????????????????????????????????????????????????????????????????????????????????????????????????????????????????????????????????????????????????????????????????????????????????????????????????????AGGGCAGTCCGCAGTCACGGGTGCTGAGTCCCTTGCTCGGCGCCGAGGGTACTATGGCATCTTCCCTAGTCCGCCAACTCCTGCCACCTGCTGTGTCAGCGTTCCAGAACCGCCTGCTCGCCCTCCCTTGTGTGACATGAGCCGGCAACATGAGACCAACAGCAAGATTCCTCAACACACAAGGGTCCAGCCACGGACACAACCGACTCTGCCTCGTCCCTCGGCACACCTGGAAGAATCTGACAATGGGATGTTCACAGGATGGAAGACCCCTGCAGGCCCTCTGCATCTTCCCCAACCCCCCTGACAAGGTCCCTCTGCCGTGCGAGGATTAGCTGTCTGGTAATCGCGCCTATGGTGACCTGTCCCCATGACACCTATGTGGGCCACCGCCCGGAATCTCTTCCTTCCTCCCATAACGCCTGCACAAGCTAAACGTGCGGAGCAGCGAACCCACCAAGGCTGCCGACAGCTCAAGGTGGCACTGCCTTCAGACCACTGTACGCATGTGTCCCGCCTCCAGCCAGGAACGCTGGAGGATCTCTCGTTCTGCGAAGCTGAGACAACTCAGGCCGCGTCTACTCGCCACGCAACAGTGCCCAGGGCAGCGTCGGCTTCTCAGACACGCTCCTACGCTACTAAGTTCTGGCGCCGTCAGGAGCCGGCAAAGGCCAAGTTTCTCCTAAGAACGATCTCGCTCCCCACGAGCTCGGAGATAGCGTCCTCCCGTCATTTCCTGGTAGCGGTCTGTCCGCGCCCCTCCCGCT????????????????????????????????????????????????????????????CTTAAKCATAAATCCTCCTTTTATAACACCGAGCTCCGACCCTCGGGGAAAGACCTTAGACCAAACAATCGGYCCCATTGCTGGCTTCA?GATCAGCACCGCCGG?GTATTTCAATTCTCCTGTTCGATGGTTGGTGACTT?CAACCATCTTAAGGGCCAAAACTACGGCCTAGCTGGAAAACATACGGGACTCTTACGCCCGTATTAACTTTTCCTTTAAGTTTAGTTAGACTGTCTCGGGGGCGGTCGGCGTCCTCCTCGCGGATACTGCGTTTCGCCCTCTAACCGGCTCCCCGGGGCTCGTTAGTC?GGGGCGGCCGGTATGTAATTATCAAAAGGTTCGCCTGAATATTGGCAGGTGCCTCGGTTCTATTTGTTTTTCGGAAGCGAGGTAGTGGAGACCGGTCGTTGCGCGTTTGTCCGCCAGAATACAGGCCGTGCCTTAAAGCCATGTGCACGACAGCTAGGATGGCGGACTTGTATTTCGACTCCTCCCGACCTTYCGACAAGCTTTGGCCATAGTGGTTAGGATCCCCCCGCCTATTGATGGGGCTCTAGCGCATTCGTCGTGCCGGCG?AACAAGTGCGTTCCACGGTTTCGCCAAGGACTGCTGT??CCTTGGCCTAAAGTCGCCCGTTACCTCCTTCTATTGGGGCTCTCCCATGGACGGGGATCGTCCTCTCGATGGTTTGTGAYCCTTGCTGGTCCCGCAGGGGCAACCGCCTGCGTGGCGGGGAAGGTCGCTTGACTATCA??????????????????AACATTTCCGTTGGTGG?TGATGTAATATCGAGAGCTCTACGCCCTTTACAAATAAATGACCCTCAAGAGGACTTGAATTTCCAAAAAGATATAGGCTTTACCTAACCCCTACTA???????????????AAAGGTTAGTGCAACTAGAGAAACTAAAACCTCTCTTTC?????????AAATCTGTCCGATAAAAAAAAGCCTTAAGCTATCCTCTTTAAGACATTGACAAGAGGGT??????????????????????????CTCCACAAACTAAGCCTATACGGTGACGACCGCCCCTGCCACGGCAGCGGGAATGTGTTAGGAGAGCCTGTGGGG?CGGGCGGAGGCGTCCGGTCCTTATGAAGGGGTGTGAGACTCTTCAGACGCCGCCGTCCCGTCGCTAGTCTCCTCGCGTAGTGCAGTGTAACATCTGCTGGCACACAAATTAACAGCGTATAGAAGTGGATGGGACGCGTAGTGGACCGGCGGGTGTT?GGTGGATGCGGC???????GGATCGGA??TCGTCC?AGGAATG?CACTTCCGCGGGCAGCCCACGACTGTTCGGGGCCGCCAGGCCCGGGGGAAGAGCTCCTCGGCAGAGTGTTATAGCCCTTGGTGTTCCGGCCGGCCTCGGGACAAGATCCTTACCCGCCG??CCCGGCAGCGCTCGTGGCAACTCGACTGTAGGACTGCGCATGCT?CGGCGACCGTCTGGGTTTGGCGCTCAGGGTCAGTGGCGAAAGTTGGCTCCCCATCAAGCTAAATTGCACATTGGCGCTACGAAACCTAAAGTATGAGTAAAGGCTCTCTTACGGGGCTGAGTGTTCCGGCCTGTCGGCACACGCCGCTCGAGGGCCGTCCCTGAGGCGGCCAACGTACACTTAGGTCCCTATTCACAAGCTGGACTAGACGACTTGGTTGACTCCTGCGCTCGATGAGTATCGTATCCGACGAACTCGTTAAGTCAGTGCTAGTCACCTGGCAGTCAGGTGCCGCCCTACTCCTATTCCGAAGATCTCATAATGATTTGTGAATACAACGCCGCTCCGTGTCACTAGGGTCGGATAGGACGTCGGGCACTCGCGAGTCCCCCCGACGGGAGGCCCTCGGAATCGGCGTTCGCTAGTGCGGGCGCGAGGTCATACGGATATGTTGGACGTGGATAGCGACGCGTTCTTAATGGGTGCAATTCCATACCCACA?CCTATAACGTATCGTCTGGACGGGGAGATGTG?CTCCGGCACCGAGCGCACAACGAGCCAGAGCTCGGCGGCATCGCGGACTTGAGACAAACTCTGTGGGTTGAGCCATGACGTAGCTACCTACCGCCCCCCGGGCCTGCGCTGCCGTCGGCTAATCTGGGGGTTGTGTGATTTCCCACCAGTCTGGACCATCGATAAAAGATTATCATCGTGGGGCTGTCGTCTGCGGTGCGAGC?GGCCT?AATCGGCGAGGGCTGGTGAGGCCTCGGGGTCGACCCGCCGACGCTGAGACCTTCTCGTGGAGCGCGCACTG?CGAGGCTCGGGATGTCCTCTTCGGCCGGAGTTAGAGCCAATCTGACTAATGCTGCTCAGACGGTGTGATTAAAACCCGGATCCAACGCAAAATTAGCTATCTTCTGACGTTTAGAGAACGAGAGCACGGGCAATTTGTCGCGTTTCTTATGAGTAAACGGAGAGCGGGCGAGGCCTATCCTAGAATTCCGCATTCGCGTCGGGACGTCCCTTCTCAGCGTCAGAGGATAGCCCCCGAAGAAGGCTCTAGACTTCGCTTTTTCGAAGTGGTCACTGTTGATCAAGGATTATAACCCTTGATCGCCGTTTATCCCTCCCGTGCCCTCAATCTCGCTATAACGATTGAGTCAGGCTCGAC

**STERN**_*Sternaspis*

?????????????????????????????????????????????????????????????????????????????????????????????????????????????????????????????????????????????????????????????????????????????????????????????????????????????????????????????????????????????????????????????????????????????????????????????????????????????????????????????????????????????????????????????????????????????????????????????????????????????????????????????????????????????????????????????????????????????????????????????????????????????????????????????????????????????????????????????????????????????????????????????????????????????????????????????????????????????????????????????????????????????????????????AGGGTGGCCCGTTGTCATGGGCGCTACTTCCTTCGCTCCGTACCAACAGTATTACATCACCTTCTCTCATCCATTCAGTTTAGTGATGTGCTGCGTGCACGCACCAGAACCGTCTGCTCCTGTACCCGTGAGAGACATGCGTCAGCAACATGAAACCCACAGCGAGGACCATCAAGACAAGAAAGTCTGCCCACGAACCTAACCGACACCGCATCGTTAATCGGGTTTCTTGGAGGGTAGTAGCAATGGGTTGTTCAAGAGCTGGCAAATCGTTCGATGCTTGCAGTGTTGACCCCAACTAACCAGACAAGCTCCCTCTGACGTATATGGATTACGCTCAAGGTTAGTTGACTTATGGGGACGTGCCGTTAACTGACATATAGGGGTTTGTGCCTGGAGGCTCCGAACACTTCTCGCAACGCTCACACCGTCAATACTGCCGGCGTTCAGCAACCTCCAGGGAAGCGCAAGAATCAAGGTGTTACCGTTTTCAGATAACGCAGTGCGTGCGCTCAGTTCCCAGCAGGGAACGATCGAGGATTTCTCGTAGTGCGACTCTGGACTACACGCCAGCATATCTTTGCGCCACGTAACAGTGCCCAAGCAAGTGTCAACCTCAATGAAACGCCTCTACGAGATTTCACACTGGTGCCGATCTGAGTCAGTTAATATCAAGAATTTCCTGCTCTCGGAGCAACGCCAGGCGAGCCTGGCTACTATGCTCGCACGAGCATTTCCAGATCAGGCCTGTACTCGCCCCCCCCGCT??????????CTTATGCTGCTAATACAGACTTTAACATAGTAATGTTAATAGTTGTGGTCCTTAATCGTTCTTACTACTTTTATAACACGAAGCTCCGACCTCCGGGGAAGAGCCTTAGATCAAACAATACGGTCCCTTGCTGACTTTGGGTTCAGCACAGCCGGAGTATTTCAACTCTCCTCTTCGTCGGTAGGTGATATCCTACCGAGTTAAAGGCCAAAACTACACCCTGAAAGGAACACATACGGGACTCTATCGCCCGTATTAACTTTTCCTTTAAGTCTAGTTAGACTGTCTGAGGTGCAGGCGGCGTCCACCTCGCGGGTACTGCGTCCTGACCTCCATCCGGTTCCCTTGGTCTCACTAGTTCTGGGTGGCCGGAACGTAATTATCAAAAGTAGTGCCTGAATAATGGTAGGTGCCTCGGTTCTATTTGTTTTTTTGGAAGGAGGTAAGGGAAGATGTTTGTTACGGTTTAGTCATCTAAAACACTAATAAAATTTTAAAAACATGTGCACGACGACTACGATCGCCGGATTGTTTCATGACCCGGCCACAGCTTCCGACAAGTCTTGGCCATAGTGATTAGAATCCCCCCGCCTGTTGGTGGGGCTCTAGCATATTCAGCGTAACCGCTGAACAAGTGCGTATCACAGTTTCGCCAAGAAATGCCGTTCCTCTGGATCGCGGCTGTCCGCTACCTTCTTCTATTGGGCTTTTCCCATGGACAGGGATAGTCCTTTTGATGGCCTGTGATATTCGCTGGTCCCTTAGGGGCATCCGCCGCGGTGGCGGAGATTCTCGCTGTGTCATCA???CAGATACGCGGGCCTAACATTTCCTCTAGTGGCTATTGTGACAACGAAAATTCCTCGCCTAAAGCCAATGAATAATCCTCAGGAGAGCCTGAATATGTTAAAAAATATTGGCTTAATTCTAATATTATTAAAA?ATAAAATCACCAAGAATTTGCGCAGCAAAGGCAACCAAAAATTCCTTCTTTCAAAGTATAAGTCTGTCTGATTAAAACTAGCCATAAGCTATTTTTCTCTAGCCAATGACAGAAAAGAGGCGCAGGATATCC????????????CCCCACTAACTCAGTCTATGAGGCCACGACCGCCCCCTGGGAGGCCGGGGAACCGTGTTAGGAGAGCCTTTGCGGCGG?TCTGGGGCGTTCGGTCCTCCTGTCGGGGTCACAGAATCGTCAGCCTGACCTGGGCCGTCGCTTGCCTCCTTGCGTAGTGCAGCATAACATCTGCTGACACTCGGATTAACA?CGTTTAGAGGCGGATAGAACGTTCAGTCTGCCCGTGGATGCCTGTTTCGCGTTCCTGGTCCTGGATC?????????ACCGGGAGGTGCACTTCCTCGGGGAGCCCACAACGGCTCCGGCCGTCTATGTGTGCCCGAAGAGCTTGGCGGCTGAGTGTTATAGTGGGCGACGGACCGGC?CG?TTCCGGGCCAGA????????CGATCGGACACGCGGTCTACGACGCTTGCCGGCTGGAGGACTGTACATGCTCCGGCCGGTGTTAGGAGAGG?C?CCCAGGGTTAGTGGCGTCGGTCGGCAACCTATCAAGCTAAATTGCGCGTTGGTTGGTCGAAACCCGAAGCATGAGTGAAGGCCTTCCACTTTGGCTTAGTAGTTCGCGCTGGCGACCTACGACGCTCGG?CTATGTCCGAAAGGCGGACATCGTACATTTGGGTACCTATATACAAGCTGGTCTAGACAACTCGGTTAACTCCAGCACTCGTCGAGTATTGTATCTGCCGAACACATTAAGTCGGTGTTTACTATCCGACTATCGAG?GCTGCCCTACTACTAATAAGGCGATCTCATAATAATTTGTGAATACAACGCCGATCCGTGTCACTAGGGTGATTTAGCACGTCGGGCAAGTTTATGGCGCCGCGACGGGAGGTCCGTGGAGACAGCCCTGGCTAGCTGGGGCGCACGGTCATACGGATATGTTGAACATAGAAAGGCAAGCCTTATGAAGTGGGGCTCTGA???ACCCCACATCGATATCGTATCGACAGGACATGGAGATGGCCCTTCCGGGTCCAGTGTACAACGAGCTAGAGCTCGGCGGCATCCCTGAATTGAGGCGGACTATGTCGGCTTGGCGATTACGTTGTTTCCTACCGCTCCCCGGGAGTGCACTGTCGTCGGCTAATCTAGTTACGGTGTTATTTCATGCCTGACTGGACCATCAACAACAGATTGTCATCGAGAGGCTATCGTCTGGGGCTCGAGCGTCCTGGAACTGGCAGGGGCTGGTGAGACTGTTCGGTTCACTCGCCGTCTCTGGGACCTGTCCGTGGAGTGCTCACTAGTGGGGGTAGATCTTCTCACTTCGGCCGGGTGTGAAGCCAAGTTAACAAATGCTGAGTAGCGCTTGTGATTAAAATCCGGATTTAGCGCAGAATTAGCTATCTGCCGACTCTTAGAGAATGAGAGCACGGGTCAGTTTTTACGTTTCTTATTTAAAAGCGGAGGCTGGGAAACCCCTACCCTGGAATTGATCCGTCGGCCGGGAACGTCCCTCTAAAGTGTCAGCGGATATAGATTGAGGCAGGCGCTACACTTCACTTTTTCAAAGTGCTAACTACTTATTAAGGATTATAACCTCTGATCGTCGTTACTCCCTCCCGTACATTTATTCTTGCTATCACAATTGGATTCTTATCGGC

**SYLLI**_*Eusyllis-Exogone-Proceraea-Typosyllis*

???????ACGGTCTTAGGACGTCAAAGATTTATAGCTGTGGGTCAAGTCTTTACGGGAAGAGATCATATTACAATGTTCATCATTTAATTTTATATATTATAATGATTGTTGTAATTCAACAGGGGCCCATAGTTTTTTTATTAAAATATATACCCTTTTAACTTATGCGGCCAGCGCTACAGAGAAAAGGAAGATGTCCTTATGGGTAATATTGCCTGCTCCCATCAGTCTACATTTCTCTAGTAGTAAGGTTTAGGGCATATTACACTTATTATTCAGGGTTGCGCTTAGAGCGAGTTCTTATATTATCTGTAAAAACTGCTGATGTATATATCTCAGACTAGACCATTATACTCTCGTACTAACTGCCTTTTCCTCG???????????????????????????????????????????????????????????????????????????????????????????????????????????????????????????????????????????????????????????????????????????????????????????????????????????????????????????????????????????????????????????????????????????????????????????????????????????TCACGGGTACTACTTCCCTCGTCCGGCGCGGCCAGTACTACGGCATCCTTTATCTTCCGCTCACTTTAGTGACTTGCTGTACTTGCACTCAAGAACTACCTGCTCGCGCACCCCTGAACCACGAGTGCCGGCTCCACGAACAAGACTCCGAGAACCGTCAACGCCAGAAGGTCTGCCCACGGACACAACTGATGTGGCGTCGTGCCTCGGCACACCTGGAGGCTTCCACCAATGAGCTGTTCAAGGAATGGAAGATCGCTCGATGCTCGCAACATTGATCCCGACCCCGCTGACAAGGCCGCTCTGACGTGCGTGGATCAAGCGTCTTGTTGTCCGGCGTATGGGGACCCGTCCCCAACGGACCCGCGCGGGCCCAGGCACGGAGGAGCTCTGCACCTCCCGAAACGGCCGTATCGTCTAAACGTGCGGAGCTCCGAATTCTCCAAGGGCGCTGACACCTCCTCGCGTTACCGCCTACAAACTCGGCTACGCGTGCGTCCTGGTTCCAGCGTTGAGCGCAGGAGGATCTCTCGTTCTGCGAAGCTGAGACAAGGGCCCACGCGTCATCGCGCCAGGTGACAAAGCCCAGGCGAGCGTTGGCTTTGCCGACATGCAGCTAGG??????????????????????????????????????????????????????????????????????????????????????????????????????????????????????????????????????????????CTTATGCTGGTAATACATGCTTGTACATAGTAATGTTAATAGTTGTGGTCCTTAATCGT??ACACAGTTTTTATAACACCAAGCTCTGATCCCAGGAGAAGAGCCATAGATCAGACAATGTATGACTGTACTGGCTTTGGGTTCAGTTACACCGGAGTATTTCAATTCTCCTAGTCGAAGGGAGGTGCTATCCTCCCTTTTTAGAGGCCAAAACTACGCCCTGACAGGAACACATACGGGACTCTTTCGCCCGTATTAACTTTTCCTTTAAGTTTAGTTAGACTGTCTGAGGCTGTCAGAGGGTCCAGGTTCCTGGCACTCCGTTGATACCTACTGGTGGGGCTGTGGGGCCGACTGGCCCTCTGCTCCTACTCAATAATTATCAAAAGTCTCGCCTGAATAATGGTAGGTGCCTCGGTTCTATTTGTTTTTCGGAACTGAGGTAAGGGAAGACAGTCGCTGCAGTCTTGCCACTCAGAATAGTGATCGAATTTTAAAAACATCTGCACGATAACTACGATCTCCGGCTTGATCTATGACCTGGCAGCAGCTTCCGACAAGTTTTGACCATAGTGGTTAGGATCCCCCCGCCTGTTGGTGGGGTTCTAACACATTTGCCTTGCAGGCATAACAAGTGCGCTTCACAGTTCCGTCAAAGAATGCCGT?TTGTTGGTCTGAAGCTGTCCTGTACCTCTTTCTACTGGCCCTATGCCATGGACAGAGATCGTCTTTTTAATGGTTTGTGACCCTTGTTTGTCCCACGGGGGTCTCTACCTTGGTGGCGAGGACGGTCGCTTGATCATTA???CAGATCCGCGGGCCAAACATTTCCTTTGAAGGCTAATGAAATAACACGAATCTATCTCCACTATTCTCTAAATATTCTTTAGGAGAACCTAAACTTTTAAAAAAGTATCAGCTTCATATTTTCTAAATT????????????????AAATATTGGTGCGATTAATGAATTTCTACTCTTCCATAATTAAAGCATTTATCTAT?ATTTAAAAAAAAGCTGTAAGCTATATCTTCTAAGTCAATAAAGAAGATGAGGCGCAGGATTACCTTATAATAACTCCTCCACAAACTAAGCCTATGCGGAGACGACCGCCCTCAGCCTGGCTGCGGGAACGTGTTAGGACAGCACTTGGGGTGG?TGGCTGGTGTCCGGTCCTCCTGTCGGGGCTACAGACTTGTCAGGTGCCGGCT?GCCATCTCTGTAGTCCTTGCGTGGAGCAGTGTAACATCTGCTGACAATTGAATTGGCAGCGTTTAGAGGCGGATGGGACACAAAGTCGACCCGCGGATACTGGTAGAGCGGTGGGTGCTC?TGATCCTACACCGTCTTTGTCAGTGCACTTCCGTGGGGAGCCCACGACAGCTCTGGCTGCCTAAGCCTCCTGGAAGAGCACCGGTGGGGTGTGTTACAGACGGGAAGGTATCGGCCAGTTGGGGGGCTAGATCCGCACCTGCTCGGCTCGGTCCGCTCTCGGCGAGTTTAACTAGTGGACTGTACATGCACTGACCACTTTAGGACTGGGTTGCTCTAGGTCAGTGGCGTCGGTTGGCACCCCATCAAGCTAAATTTCGCACTGGTTCTACGAAACCTAAAGCATGAGTGAAGGCCCGCTGTAGGAGCCTAGTAGTCCCGCTCCTGGGCCTAYGCCGCTTGTCTTCCGTAAGTGAGGCGGACAACGTACACTTGGGTACCTATTTACAGGCCGGACTAGACAACTTGGTTAACTCCTGCACTCAACGAGTATCGTATATGCCGARCACATTAAGCCTGCGTCGATTGCCAGGCTTTAGAGTGCAACTCTACTCGTATTACGAGGATCTGATAATAATTTGTGAACTCAACACCGATCTGTGTCACTAGGGTTGGATACGACGTCAAGCATCGGGCAGTCTCCCTGACGGGAGGCCGGTGGATCCGGCCTTGGCCAGCCGGGACGCACCGTCATACGGATATGTTGAACGTAGATAGGAACTCGTTCTGAAAGCAGGCAGTTACCAGCCTGTCACCTATATTGTATCAACTGGTCACGGAGATGGCCCCTCGGGGTCCAGTGCACAACGAACCAGAGCTCGGCAGGATCCCGGACTTGAGGCGGACTCTGGCGGCTTGCCGATGTCGTTGATCCCTACCGCTCCCCGGGCGTGCACTCCTGTCGGCTAATCTGGGGAGGGTGTGATTTCGTACCAGACTGGACCATCGATAACAGATTATCATCGAGAGACTGCCGTCTGGGGTACGAGCAGCCTGGAACTGGCAGGGGCTGGCGAGACC??CGGGTTGACTCGCCATCGCCGGGACCTTCCTGTGGACTGTCCACTGGCTGTGGCGTGGTTCTCAGCTTCGTCCGGATTCAAAGTCGATTTAACAAATGCTGCGTCACCGGTGTGATCAAAATCCGGATCTAACGCAGAAACAGCTATCTGCCGACTCTTAGARAATGAGAGCACCGGCAATTTTTTACGTTTCTTATTTATAAGCGGAGAGCGGGCAAAGCCTCGTCTGGAATTCCCGACTCGGCCGGGGGCGTCTCTCTAAAGTGTCAGCGGATAAAGTTTGAAGTAGGCACTACACATCACTTTTTCGAAGTGATAACTATTTATTAAGGATTACAGCCTTTGTTCGTCGTTAATCCCTCCCGTACATTTATTCTTGCTATAACAATTGGATTAGGATTGAC

**TEREB**_*Amphitrite-Lanice-Pista*

????????????????????????????????????????GGGCATATCATTCTAGGAAGAGACCACACTACTGTGTACTTCTTCCAATCCTAGATATTTTAATAGTTGTCACTATACTATAGGGGCACCCAGATTTAAAATTAAAATACTCGCCCAGTCTATACTTACAAGATCCGCAGCAGAGAAAAGAAACATGCTTTCTGAAGAAACCTTGCCTGCTCCCTTCAGATTACTTCCTACTAGAAATTTCAATCAGTDSNNNTTACACAAGTTGCCTAGGCCTCCGTTTAGAACGAGTTCTTATGTAAGCTGTCAATATTGTAGTCTCTCTCTTCTCAGCCGAGACAATAACCCACATGAATGTATTTCTTTTTCGGCTTTTCTACCTATCACTATACAGCCCCGTTGAAAATGGCAACTTTGTAAAATTCGAATAATAGACAGCTGAAATTTTTTCTCAAGTTATATGAACCAGCAATAGAAGCTTAATATTTCATAATAAATAAAATGTCTTCTCGCATTCATACAATGCTTTACTTACTTCTCTTCTAGAAAAA???????????????????????????????????????????????????????????????????????????????????????????????????????????AGGGCAGCGCGCGGTCGTGGGCGCTACTTCCTTCGCCCGGTGCCCCCAGTTCTTTGCCATCCCCTATAATCCGCTCAATTTTGCGACTGGCTGTACTAGTGAAACAAAATCGTCCGCTCACCATTCCTTGGGCCACATGAGCCAGCTCGACGAGACCAACAGTGAGAATCTTCAAAACGAGAAGCTCCACTCACGGATGCAACCGCAACCTCGTCGTGCTCGGGAATACCCCGAGGAAAGTCCAAATGAAGTGTTCAAGGGATGGATGACTGGTAATGGCCTGCAACATTGAACTGCAAGCAACTGACAAGCACACTCTGACGTACATGGATCAAGTTCCTGGGCATCACGCTTATGGGCACCTGTCGCCAACCCACCTGTACGAATCAGCGCTCGGAGAAGCTGAGTTCCTCCCGCAACGCCCGTATCGTCACAACCGCTGGCGCAGCGAATTCTCCAAGGAAACGGATGAGTCCGTTAGCCACCCCCAACAGAGTGTGCTGCGCCATGCTTCCGCCTCCAGCCTCGAATGGTCAAGAATCTCTCGAAGTGCAGAGGTCAGACAAGCCAACATGCGTCTTTGTGCAATGCGAAAAAGTGCAGGGCTGAGTGGGCTACATTGTT???????????????GACACCAGAACGCCGTCCAGAGCCGGCCTAGGCTCTGCACGTCCTGCTAGCGGTCCCGCCCGCCGCGCGCTCGCCTACCGCGCCCCCACGAGCCCCCCCGGCAGCGGCCTGCACCCGCCCC???????TCTGGTTCCCGTTGTGCAGTTACTACAAGCTTTTACACAGTAACGTTGATAGTTAATATTATTAATCGTACATCCTACTTTTGTAAGAAAAAACTCCGACCTTCGGGGAAGAGTCTTAGATCAAACAATCGGGGCGCTTGATGACTTTGTGTTCAGCTCAGCCGGAGTATTTCAGTTCTCCTCTTCGAAGGTATGTGATATCTTACCTTGTTAAAGGCCAAAACTATGCCCTGGCAGAGAAACATACGGGACTCTATCGCCCGTATTAACTTTTCCTTTAAGTCTAGTTAGACTGTCTCGGGTCCAGGCTGCGTTCTGCTCA?TGAAACTGCGTCCTGACCTCCTGCCGGTTCCCTTGGGCCGACTGGTTCTGGGTGGCCGGAACGTAATTATCAAAAGAGCTGCCTGAATAATGGTAAGAGCCTCGGTTCTATTTGTTTTTCGGAACTGAGGTAAGGGAAGACGGTCGTTACGGTTTTGTCGCCTAGAATACTAATCGAATTTTAAAAACATGTGCACGACAACTCCGATTCCCGACTTGTTTCTTGACTCGGCGGAAGCGTCCGACAAGTCTTGGCCATAGTGATTAGAATCCCCCCGCCTATTGGTGGGGCTCTAGCATATTCGCCGTGTCGGCGCAACAAGTGCGTTTCACAGTTTCGCCAAGGAATACGTTGTCTCTGATCCAAGGTCGTCCGTTACCTCCTTCTATTGGGCTTTTCCCATGGACAGGGATAGTCCTTTTGATGGTTTGTGATCCTCGTTGGTCCTGAGGGGGCAACCGCCGCAGCGGCGAGGACGATCGCTTGATCATCAAAA???????????????GGAATTCCCCTTGTGGGCTAATGAGACAACGAAAGATTAACGCCTACTATCAATAAATGACCTACAGGAGGACCTGTAAAAAATAAAAAGCGTAGGCTTCGTTTTCTCTTAATAACTATTTTAAGCCACAAAAAACTAGCGCGGCTAAGGAACATAATCCTCCATTTTTTACAAGTATAAGCCTGCCAGATCAAATATAGCCTCGAATTATCCTTCTAGAGCTACTGACAGAAGGGA??????????????????????????CTTCACAAACTGAGCTCATGTGGAGACGACCGCCCCTGCCCTGGCGGCGGGAACGTGTTGGGACGTCGCTGCGTGGACGTGTCGCTTGTCCGGTCCTCCTGTTGGGGTTTCAGATTCGTCACGCAACG?ACCGTTCTCGCTTAGGTCCTTGCGTGGAGCAGTGTAACATCTGCTGACACTCAGATTAACATCGTTTAGAGGTGGATGGGACGCAA?GTCTCCCCATTGATACCCGTGTCTGA?????GAAGTTCGATCCTTCGGCGAGGCGGTGGGCGCCCTTCTTTGGGATGTCCAGGACGGTTCGGGTGGCCAAAACCGTGTACCAGAGCTTGTGAGGTAGCGTTTAGGTGCTGGGCGGAGGCGGCCGCTTCTCGGACCAGACA????CCCGCCGGATGCGGGTCGAACGTCGTATGTTCGACCTGTGGACTGTACATGCTCTAACTGCGGT?GCGTGTTGGCACTCAGGGTCTCTGGCTTCGGTCGGCACCCCATCAAGCTAAATTGCGCATTGGTGG???CAAACCTACAGCATGAGTGAAGGCAGGCTT???CTGCCCAGTAGTCCC?CCTTGGGGCCTATGCCGCTCGATCTCCGTAGATGAGGCGGACAACGTACACTTGGGTATCTATACACAAGCTGGTCTAGACAACTTGGTTAACTCCTGCACTCTTCGAGTTTCGTATATGACGAACACATTAAGTCCGTGTCGGTTATCGGGCGATCGAGGGCCGCCCTACTCCTACTACGAGGATCTCATAATAATTTGTGAATACAACGCCGATTCGTGTCACCAGGGTCGGGTAGGACTTCGGGCACCGGGGTGCCTCCTCGAAGGGAGGTCTGCGGAACTGGCTTCGGCCAGC?GGGGCGCGCAGTCATACGGATATGTTGAACGCAGATAGAAACTCGTTCCGAAACGGGGCAATGTATCGCCCTTC?CCTATATCGTATCGACCGGACGTGGAGATGGCCCTTCGGGGTCTAGTGTACAATGAACCAGAGCTCGGCGGAACCCGAGACTTGAGACGGACTCTGTCGGCTTGCCGATGACGTTGTTTCCTATCGCTCCCCGAATGTGCGCTTCCGTCGGCTAATCTGGGGACAGTGTGATTTCACGCCGGGCTGGACCAT?GGTAACAGATTATCATCGAGAGGTTGCCGTCTGGGGTCCGAGCGGCGTGGAACTGGCAGGGGCTGGTGAGACCGAAAGGTCGACTCGCCGTCGCCGGGACCTTCCCGTGGACTGCTCACTGGCGGCGGTCG?GCCGTTCGCTTCGGCCGGATGTAAAACCAATTTAACAAATGCTGCGTAGCTGGTGTGATTAAAATCCGGATCTAACGCAGAATCTGCTATCTGCCGACTCTTAGAGAATGAGAGCAC?GGAAATTTTTTACGTTTCTTATTTATAAACGGAGAGTGGTTCGGCCCTTGCCTGGAACGCCCGACTCGGCCGGGCGCGTCCCTCTAAGGTGTCAGCGGATAGAGCTTGAAGCAGGCGCTACGCATCATTTTTTCGAAGTGTTAACTATTTATTAAGGATTATAACCCTAGATCGTCGTTAATCCCTCCCGTACATTTATTCTTGCTATAACAATTGGATTAGGATTGAC

**TOMOP**_*Tomopteris*

?????????????????????????????????????????????????????????????????????????????????????????????????????????????????????????????????????????????????????????????????????????????????????????????????????????????????????????????????????????????????????????????????????????????????????????????????????????????????????????????????????????????????????????????????????????????????????????????????????????????????????????????????????????????????????????????????????????????????????????????????????????????????????????????????????????????????????????????????????????????????????????????????????????????????????????????????????????????????????????????????????????????????????????GTGGAGGATCACAGTTATGGGCACTAAATTTTTTGCTTGATACTAAACCTACTTCGATATCTTCTTCAATCCACTTGTATTTGTGACGAGCCGAACTTACATATCAAGATAGTAAATTCTTATATTTGAGAACTACAAGAGACAGCAGTATGAATCCTATAGTGAATCTAATTAATATAAGAAATGAAGGCTACGAGCATAACAAAAGCCTGATTGTATTCATTCATGTCACGAAGCTTCTACCAATGCCTTGTTCAAGAAATGGGTAATCCCTTAACGCTTGCAGTATTCTTCCTTAAAAATCCGACAAGGTTGAATTGTTGTCCGTGGTTCAATACTAATATTATCATGCACATGGTTACTCGTATGTGGAAAATGGGAATGTATCGAATCCTACCTGAACTTTATTTTTACCGCAACGATAACATAAAAAAAATTGCTTCTGCAGCAAATCCTGCATGGAAGTTGCCGAATTCTTGCGTTGTTGCTTTCAAATAATGCTTTACTTCTATTTTGCCTCTAATCAAGAACTAAGGAAGATTTCACGATCTGTGAAATTAAGATAATTAAATTTGCATCTTTGCTGTATGAAACAGTGCAACCACTAGTGTTAGCTTTACTAATATCCTTCATCG??????????????????????????????????????????????????????????????????????????????????????????????????????????????????????????????????????????TCCTCTTATGCTGATAATACATACTTACACATAGTAACGTTAATAGTTATGGTCCTTAATCGTAAAATCCACTTTTGTAACATCAAACACTGACATTATTGTAAGTGTCTTAGAACAAATAATAGGAATCTTTGCTGGCTTTGGGTTCAGCTTTGCCGGAGTATTTCAATTCTCCTCTTCGAAGGTAGATGATATCCTACCTTGTTAAAGGTCACAACGACGCCCTGGAAGGAAAACATATGAATCTCATTTGTTCATATTAACTTTTCCTTTAAGTATAGTTAGACTGTTTCTAGTCTTGACTGCATTCGCCTAGCGGGTACTGTGTTTGGACTTCCTCCCGGTTCTCTTGTTCTCATTAGTGCTGAGTGACTGGAACATAATTATTAAAAGAGTTGCTTGAATATCTGTAAGTGCCTCAGTTCTATTTGTTTTTCGGAATTGAGGTAGGGGAAGATGGTCGTTACGGTTTTGTCGCCTAGAATACTAATCGAATTTTAAAAATATCTACATGATAACTACGATCCTTGGATTGTTTCTTGACCTGGCGGCAGCTTCCGACAAGTCTTGGCCATAGTGATTAGAATCCCCCCGCTTATTGATGGGGTTCTAGCGTATTTGCTGTGTCAGTGAAACAAGTGTGCTCCACAGTTTCGCCAAGGATGATGAT?GTGCTTGTCTAAGGTTGTTTGTTACCTCCTTCTTTTGGGTCTACCCCATGGACAGGAATAGTTTTTCTATCGGATCGTAATAAATGTCGCTATCACGGGGGTTTCCACTATGT?AGGGAGGTTTATTGCTAATACGATA?????????????????????????????????????????????????????????????????????????????????????????????????????????????????????????????????????????????????????????????????????????????????????????????????????????????????????????????????????????????????????????????????????????????????????????CTCAACAAGCTAAGCCTATGCGGAGACGACCGCTTCCTCCTTGGGTGGACGAATGTGTTAGGACGTTCTTCATTGCATTGCTATGGTGTCTGGTCCTCCTGTTGGGGTATCATATTAGTCACTGATGCTGAGCTTTGCAATTGAGTCCTTGCGTGGAGCAGTGTAACATTTGCTGACCTTTGGATTAACATCGGTTAGAGGTGAATAGAATACATTGTTGATTGTGTGATACTGTTTCGTTCTTCG??????CAGATCTTCT?GATTCGTGGTGAGTGCATTTCCGCATG?TTTCCGTGAAGGTTTAGGTGG?CTATATCGGCTTGAAGAGCTTCGGTAGGAAGTGTTATAGCTTGC?ACGAGGTGGCCACCTTGGA?ACTAGATAGTCGCCAGCTGAGCGCAG?TCGTTT?TCTACTGTTGTCCGCGAGGACTGTACATGCTCAAGCAGTGAT?GGAGTCACTGATTAATGGTCTACGGT?TTGATTGGCAATCTAT?AAGCTAAGTTGTACATGAGTTTTACGAAACTTACAAAATATGTGAAGAGCAGCT?C??TGCTTGAGTGGTCATT???ATGTGTCCATGTTGCTCGTAATCTGAATTTGAGGTGGACAACATGCACTTAGGTACCTATATACAAGCTGGTCTAGATGACTTGGTTAACTCTTGCACTGT?AGAGTATCGTATATGACGAATACATTAAGTCTGTTTTTATTACCAGGCAATCATGTGCTGCCCTACTCTTATCATGATGATCTCATAATAATTTGTGAACACAACGCCGATCTGTGTCACTAAGGTTGGATAAGATGTTGGACATCGGGATAGCTTCGCAACAAGAGGTCTGTGGAATAGGTCTTTGTTAACTGGGGTGCATGGTCATATGGATGCGTTGAACATAGATAGAAAGTTGTTCTGAAA?GGGGCAATGCCATGCCTA?ATTGTATGTCGTATCGACCGGATGCGGAGATTGCACTTCTGTTGCAAATGTACAGCGAACCAGAGTTTGGCAGAATTCTGGACTTGAGATGGAATCTGTCGGCTTGTCGATGACGTTGTTACCTACCGCTCCTCGGGCGTGCACTTCTGTTGACGAATCTGGGGGTGGTGTTATTTCGTGCTGGACTGGACCGC?AATAACAGATTATTATTGAGAGGCTGTCGTCTGTTGCAAGAGCAGATTGGGACTGATTTGGGCTGGTGAGGCTTCAAGGTCTACTCGCTGTTGTTGGGACCTACCAGTGGAATGCTCACTGGTGGTGAT??GGTTATTTACTTCGGCCGGATTCAAAGCCAATTTAACAAATGCTGCAAGGTTGGTGTGATTAAAATCCGGATCTAACGCAGAATTAGCTATCTGCTGACTTTTAGAGAATGAAGGCGCGGGCAATTTTTTACGTTTCTTATTTATAAGCGGAAAGTGGGCAAACCCAAATCTAGAATACAGGATTAT???CTGTGTGTCTCTCTAAAGTGTCAGCAGATAGAGCTTGAAACAGGCGTTACACATCACTTTTTCAAAATGTTAACTACTTATTAAGGATTATAACCTTTGATCGTTGTTAATCCCTCCCGTACATTTATTCTTGCTAAAACAATTGGATTAAGATTGAC

**TRICH**_*Artacamella-Terebellides*

AGTGCGAGGAGCCATAGTACCTCAAAACTTCATAATTACTGGTCAAAGCTTACCGGGAAGAGACCATGCCACTACGATCCTCATTTAACTCTAAATGTTTTCACAATCGACGTGATATAACTGGAGCTCTTAGATTCAGAACTAAAACACCCGCACTGACTATTCTCTGTAGATCTGCTGCAGTGAAAAATTCAATGCCCTCATTGACAATATGGCCTGCTACCATCAGTTTACTTCTCTCTAGATATCTCAGTCTGCTCAATTTATACATGTAGCATAGGCCTTCGATTAGAACGAGTTCTTATGTTAGCTGCCGTTATAACTGCCACTCTCATCCCAGTTATGACAACTACCCACATGAATATATTTCTTTTTCTGCAAAGCTATTAATCATCAC?????????????????????????????????????????????????????????????????????????????????????????????????????????????????????????????????????????????????????????????????????????????????????????????????????????????????????????????????????????????????????????????????????????????????????????TCCACGGGTGCTACTCCCCCTGCTCGGTGCTGAAAGTACTACACCATCTTCTTTCTTTCGCTTTCTTTGGTGATCTGCTGAACTTGTGCTTCAGAACTGCAAGTCCGCCTTCTCTTAAGCAACACGTGCCGGCTCCAAGAGCCCTACAGCGAGAGCCTTCACCGCAAGAAGGTCCTCGCACGGACACAACAGATACTTGTTTGTGACCAGGCATTCCCCGAGCCTAGCGCCAATGTCGTGTTCAAGGGATGGGTGACTGTTCGCAGCTTGCAACATCTCCGAAAAAAAGCCAGACAAGCCCCCTCTGTGGTACGTGGATTAAGCGACTGGTCATCAGGCTTCTCACTACCTGTCTGCAATACACCTACGTGGGTCTGTTCCCGGATAAACTGAATCCCGCCCGAAACGCTGGCACCGTCAGTACTTGCGGCGTTCCGAATCGTCTCTGGAAGCTGCTACTTCCTTGCGTTGTCGCCTTCAGAACAGGCTCTGCAAGTGCCTTGTCTCCAACAGTGAGCGACCAAGAATTTCCCGTAGCGCGGACCTGAGACAACACAACACACGTCTTTGTGCTCGGGGAGAGTCCCTCCGCCAGCGTGGGCCTTGCTGACATCCC??????CTATCACGCCAGAGCGCCGACAAGAGCCAGCCTCGGCTCTGCACCTCCTGCTCTCGGAGCCGCTCGCTGTGCGCTCGCAAACCGCGATCGCCCGAGCCCCCCCGGTAGCGGTCTGCTCGCGCCCCCCCCGCTTCTGTTTCCTCTTATGCTGCTAATACAAGCTCGAACTTGGTAACGTTAATAGTTGATATTATTAATCGTACCAGTTACTTTTGTAAACGCATGCTCCGACCCGAGGGGAAGAGTCTTAGATCAGACAACAGAGGTCATTGATGGCTTTGGGTTCAGCTCAGCCGGAGTATTTCAATTCTCCTCTTCGATGGTACGTGATATCCTACTATGTTAGGGGCCAAAACTATGCCC?GGCAGAGACACATACGGGACTCTATCGCCCGTATTAACTTTTCCTTTAAGTCTAGTTAGACTGTCTCGGGTCCAGGCCGCGTTCTGCTCATTGAAACTGCGTCCTGACCCCTCGCCGGTTCCCTTGGGCCAACTGGTTCTGGGTGGCCGGAACGTAATTATCAA?AGTGTCGCCTGAATAATGGTAAGTGCCTCGGGTCTATGTGTTTTTCGGAAACGAGGTAAGGGAGGACGGTCGTTACGGTTTTGTCGCCTAGAATACTAATCGGATTTTAAGAACATGTGCACGACAACTAAGATCCCCGGTTTGTTTCTTGACTCGGCGGCATCTTCCGACTAGTCATGGCCATAGTGATTAGAATCCCCCCGCCTATTGGTGGGGCTCTAGCATATTCGCCGTGTCGGCGCAAC?AGTGCTTTTCACGGTTCCGCCAAGGGATGCGTTGTCTCTGGCCCGAGGTCGTCCGTTACCTCCTTCTATTGGGCTTTTCCCATGGACAGGGATAGTCCTTTTGATGGTTTGTAACCCTCGTCGGTCCCTCGGAGGCAACTGCCGCGGTGGCGAGGACGGTTGCTGTATCATCAA??CAGATACGCGGGCCTAAAAATCCCCTTGTGGGCTTTTGTGATAACGAAGGCTTAACGCCTTATACTAATAAATGGCCTTTAGGAAGACCTAAAAAAGTTAGAGAACGTAGGCTTGGTATCTTAGTTATAAAAA?TAAACTCTATTAGATTCTGGCGCGGCTATGGTAAAT??ACCCTCCATAAATAAAGATACAAATCTGTCAGATCAAAAATAGCCTCGAATTATCCCTTTTGAGTTACTGACAAAGGGTTGACGCAGGGTCTCCTAGTAACTAACCCTTCACAAACTCAGCTCATGCGGAGACGACCGCCCCCGCTAGGGCGGAGGGACCGTGTTGGGACGTCCCCGGCGCGGGCGCCTGC??GCCCGGTCCTCCTGTAGGGGTCACAGGCTCGTCACGGCGTGGGCCCCGCGCGC?ACGGTCCTCGCGTGGAGCAGCGTAACATCTGCTGCCACTCAGATTAACACCGTTTAGAGGCGGATGGGACGCAGTCGCACCCCGCGGATACC?TTTCGCCGTCGGTGCGCACCGATCCCCCG?GCTGCCGGGG?GCGCACTTCCGCGGG?AGACCAGGACGGCTCAGGCGGCGAAGGCCGTGCTCAAGAGTCCGCTGGCGGGGGCGTTTAGGAGCGGCGGGGGCCGCCGCCCTCTGGGCCAGACT???CCCTTCAGGACGCGGACCCC?CGCGGCCGACCCGACTCGCGGACTGTTCATGTCCGGACTGACGGAGGGGGGGGTCCGTGAGGGTCCCTGGCCCTTGTCGGCACCCCATCAAGCTAAATTGCGCAAGGGTGG???CAAACCCAGAGCATGATTGAAGGGCCGCTT???GGCCTCGGTGGTCCCCCCTGGGGGCCCACGCCGCTCGGACCCTG?GGTCGAGGCGGACGACGCACCCTTGGGTACTTACCTACAAGCTGGGCTAGACAACTTGATTGACTCCTGCACTCATCGAGTATCGTATATAACGAACATATTAAGCCTGTGTTGGTTATCGCGACATCGAGGGCTGCCCTACTCCTACCACGAGGATCTGATAATAATTTGTGAATACAACGCCGACCCGTGTCACCAGGGTCGGGTAGGACGTCGGGCAACGGGACGCCTCCCCGACGGGAGGTCCGCGGAATGGGCCTCGGCTGGCCGGGGCGCGCGGTCATACGGATATGTTGAACGCAGATAGGAACTCGTTCGGAATAGGGGATATGACGTCCTCGGC??TTATATCGTATCGACCGGACGTGGAGATGCCCTCT?GGGGTCCAGCGTGCAACGAACCAGAGCCCGGCGGGACCCCGGACTTGAGGCGGACCCTGTCGGCTTGGCGATGGCGCTGTACCCTACCGCTCCCCGGGCGTGCGCTCTCGTCGGCTAATCTGGGGCCAGTGTGAATTCACGCCGGGCTGGACCTGTGATGCCAGATTATCATCGAGAGGTCGCCGTCTGGGGTCCGAGCGGCGTGGAACCGGCAGGGGCTGGTGAGGCCGCGAGGTCGACTCGCCGTCGCCGGGACCTGCCCGTGGACCGCTCACTGGCGGCGTTCCCGGCGTCAGCTTCGGCCGGGTTCGAGACCGGTTTAATAAATGCGGCGACGCCGGTGAGATTAAAATCCGGATTTAACGCGAGGACTGCTATCTGTTGACTCTTAGAGAATGAGAGCAGCTGGAGTTTTTTACGTTTCTTATTCATGAGCGGGAGGTGGTCAGCCCCTCGCCTGGAACGGCCGACTCGGCCGGCCGCGCCCCTCTGACGTGTCAGCGGATAGAGCTTGAAGCAGGCGCTACGCATCATTTTTTCGAAGTGCTAACTA?TTGTCAAGGATTATAACCCTAGTCCGTTGTTGATCCCTCCCGT??????????????????????????????????????

**TROCH**_*Trochochaeta*

?????????????????????????????????????????????????????????????????????????????????????????????????????????????????????????????????????????????????????????????????????????????????????????????????????????????????????????????????????????????????????????????????????????????????????????????????????????????????????????????????????????????????????????????????????????????????????????????????????????????????????????????????????????????????????????????????????????????????????????????????????????????????????????????????????????????????????????????????????????????????????????????????????????????????????????????????????????????????????????????????????????????????????????AGGGAGGCCCGTCGTCACGGGAGCTGAGTCCTTTGCTCCGTACTGCCAGTACTATGCCATCTTTTCCAATCCGCTTACTTTTGCCACTTGCTGTGTGCGCGTTCCAGAACCGCCTGCCCGCTCGGCCTTCGGCGACATGAGCCGGCTCGACGAGCCCCACAGCCAAGCTCTTTGAGACAAGAGATGAAGCCCACGGACTCGACCAAGGCTAGTTCGTGACAGGGCATACCAGGAGGCATCCTCAAATGAACTGTTCAAGGGATGGGTCACCGTTACAGGCCCGTGAGATCCTTCCTAGACAACCAGACAAGCCCGCTCTCACGTCCGTGGATTAGCTGTCTTGTTATCCCGCTTACCGCCACCCGCAGCCAACGAACCTGCGTGGGCCACCGCATGACAGAGCACTCCTCTTTCCGGAACGGCCGCACCGTCTTAATCTGTGGAGCAGCGTCTCCCCCAAGGCTGCCACCTCCTCAACGCGTCACCGCTTTCAGACAATGCTATGCCAGCCTCTCGTCTTCAGCAGGGAACTTGTGAGGATGCCTTGTTCTGCAAAGATGAGATAACAGCCAGTGAGTCTTTGCGCCATGTGAGAGTTCACAAGCAAGCGTGGTCCTCACAGAGACCCCTCTTCG??????????????????????????????????????????????????????????????????????????????????????????????????????????????????????????????????????CTGTTCCTCTTACGCTGCTAATACAAGCCTTAACACGGTAACGTTAATAGTTATGGTCCTTAATCGTACATCCTACTTTTATAACAAGAAGCTCCGACCTTCGGGGAAGAGCCTTAGACCAAACAATCGGGGTCTCTGCTGGCTTTGAGTTCAGCACAGCCGGAGTATTTCAATTCTCCTCTTYGTAGGTAGTTGATATACTACCTAGTTAAAGGCCAAAACTACGCCCTGGCAGGAAAACATACGGGACTCTTTTGCCCGTATTAACTTTTCCTTTAAGTTTAGTTAGACTGTCTCGGGTGCAGGCGGCGTCCGCCTCGCGGGTACTGCGTCCTGCCCTCCAGCCGGTTCCCTTGGGCTCACTAGTCCTGGGTGGCCGGAACGTAATTATCAAAAGAATTGCCTGGATAATAGTAGGTGCCTCGGTTCTATTTGTTTTTCGGAGCCGAGGTAAGGGAGGTCGGTTGTTACGGTTTTGTCGCCTAAAATACTAATCGCATTTTAAAAACATGTGCACGACAGCTAGGATTCTCGGATTGTTTCATGACTCGACGACACCTTCCGACAAGCTTTGGCCATAGTGATTAGGATCCCCCCGCTTATTGGTGGGGCTCTAGCACATTCGCAGTGTCTGCGTAACAAGTGCGTTTCACGGTTTCGCCAAGGAATGCGTTGTCCTTGGCCAAATGTTGTCCGTTACCTCCTTCTATTGGGCCTCTCCCATGGACAGGGATCGTCCTTTTGATGGTTTGTGACCTTCGTTGGTCCCGCGGGGGCAACCACCACGAGGGCGAGGATGTTCACTTGACTATCA?????????????????????????????????????????????????????????????????????????????????????????????????????????????????????????????????????????????????????????????????????????????????????????????????????????????????????????????????????????????????????????????????????????????????????????CTCCACAAACTAAGCCTATGCGGAGACGACCGCCCCTGCGTGCGTAGCGGGACCGTGTTGGGAGAGCCTCTGAGGCGTTCGACGGTCGTCCGGTCCTCCTGTCGGGGTTACAGACTAGCAACACTTCCGTC?TTCGTCTCTTGTCTCCCTGCGTTGAGCAGTGTAACATCTGCTGGCACTCGGATTAACAGCGCATAGAGGCGGATGGGACGCATAGTCGACCTTCAGATTCT?GGCGTTACTCTTCATCGAGGGATGCTG??TCTTTCCTCTGAGTGCACTTCTGGAGGGAGCCTACGACGGTTTGGGCCGTCAAAGCCCGGGGAAAGAGCTCCTGGAGAGAGTGTTATAGTCCCCGGTGTGTCGGCCGGCCTGGAGACCAGATC??CACCCGCCTCGGCGAGCCGGTCGTGCGCCCGTTCCACCTGAGGACTGCGCATGCTCTAACCGCGGTAGGGTCGCGGCGTTCAGGGTCTGTAGCGTCGGTCGGT?TTCCATCAAGCTAAATTGCGCATGGGTGTTACGAAACCTAAAGCATGAGTGAAGGATTATCTCAGGATCCTAGTGGTCCCGCTTTTGGGCCCACGCCGCTCGTCCGCCGTCGGTGAGGCGGACAACGTACACTTGGGTACCTATTTACAAGCTGGACTAGACGACTTGGTTAACTCCTGCGCTCGAGGAGTATCGTACCTGATGAACTCATTAAGTCCGTGTTGATTAACGGGCAGTCGAGTGCAGCCCTACTCTTACCACGAGGATCTCATAATAATTTGTGAATACAACGCCGATCCGTGTCACTAGGGTCGGGTAAGACGTCGCGCATCGGGTGC?CTCGGCGACGGGAGGCCTGGGGAGTCGGGCTTCACTAGTGGCGGCGCTCAGTCATACGGATATGTTGAACGTAGATAGCGACGCGTTCTGAAACGGGGCAATGTCGTGCCCGTA?CCTGTATCGTATCGACTGGACGCGGAGATGGCCCTTCGGGGTCCAGTGTACAACGAACCAGAGCTCGGCGGTATCCCGGACTTGAGACGGACTCTGTCGGTTTGGCGATGACGTTGTTTCCTACCGCTCCCCGGGCGTGCACTGCCGTCGGCTAATCTGGGGAGTGTGTGATTTCGCGCCAGTCTGGACCATCGATAATAGATTATCATCGAGAGGGCGCCGTCTGAGGTACGAGCGGCCTGGAACTGGCGAGGGCTGGTGAGGCCTCGCGGTCGACTCGCCGATGCCGGGACCTTCCCGTGGAGTGCTCACTGGGGTGTTCCGGCTCTCATCTTTCGGCCGGGTCTAAGTCCAATTTAACAAATGCTGCGATGGCGGTGAGATTAAAATCCGGATCTAAGCCAAAATTAGCTATCTGCTGACTCTTAGAATATGAGCGCGCGGGCAATTGCTCACGATTCTTATTTAGAAGCAGAGAG?TGGCAACTCCTTTCCTGGAAGGCCTGCTTCGAGCAGGCTCGTCCCTCTAAAGTGTCAGCGGATAGAGCTTGAAGCAGGCGCTACACATCACTTTTTCGAAGTGTTAACTATTTATTAAGGATTATAACCCTTGATCGCCGTTTATCCCTCCCGTGCATTTATTCTTGCTATAACACTTGAATCAAGTTAGAC

**VESTI**_*Lamellibrachia-Riftia*

TGAATTAACAGTTAGACCACTAGAAAACTCTATAGAGACAGGACAAAACTCTTTAGGAGACGACCAATCTTGCTAATACCCGACGCTGGCTGATCTATTCTAAGACTCGACATAGTTTAGCAGGGGCACTTGGTTTCTAATTCCAGACATAATCTCAGAGAATTCTCAGAATATCTGCTTTTATGAAAAAAAAAATGGTATCTGATCTAATATTGCTTGCCTCCATGTATTCACTTTTCTTCATAAATTTCAATTACCTCAATTTATACACGTTATATAGGTCTCCGTCTAGAACGAGTTCTCTCGTAAAGAGTTAAATATGCCGGCTCCCACTTCAAAGCCACGAGATAAACATACTCGAATCAACTTCTCTCTCTGCAATACAGCCAATCACTACATAACTTTGTTATAAACGATAAATTAGTCTAATTCGCTAAATAGATAACAGACATCATTTCAAAAATCATACGACACTCCTATACCAGTTCTATATTCCATAGTAAATAAAATGAGAGGACTGCTATATATTATGAACCACTAGTACTCCTCCTAGGCAATGATGCTATTGGGTCTACACATACTCTCATTACATGTAACTCTTCTTCTCGATGATCTAAATCTCCTTCATGGTAAAATCATCCTCAATCCAACTAGATATAATACTA????????????????ACGGGGGCTACTTCCTCTGCCCAGCGCGAACAGTACTATGGGATCCTTTATCTTCCGCTTTTTTTTGCGACTTGCTGTGTTAGCGTTCAAGAACTGCCAATCCGCTCACCCTTCGGAGACATGTGCCGGCAACATGAAACCTACAGCGAGACTCTTTGAGACAAGAAGTGCCCAACACGGGTAYAACAGACACTTGGTTGTGACACGGCATACCCTGAGGGAAGCCCCAATGGCCTGTTCAAGGGGTGGAACATCGATGGATGCCCCCAATGTGCAGCAGATCRCTCCAGACAAGCTCTCTCTGTGGTCCATGGACTATGAGCCTTGGTATCAGGCATATGGTGACACGTTGCTAACCGACTTGGGCGCGCCACAGCTCGACCGAGCACTGTTCTTCCCACAACGGCGGTGTCGCCACTTTGTGTGGTGCTCGGAACCCACACTGGAGACAGACGAGTCCTTGTGATACCGCCCAACGACCAGGCTACGCATCCCCCCGGCCTGCAGCAAGGAGCGAAGGAGGATCTCCCGTAGTGCGGTGCTCAGCCAACCCAGAGTGGGTCTCTGTGCCAGGCGAGAATCCCCAGGGCAGCGTCGAAGTCTCCGAGATGCCACCT??????CTCGCGAGGGCGGCGGGCCAAGCAAGTCTAAGATCCGGTCCTTCTGCTCTCGGAGCCGCGCCCCGCGCGCCCGGCTACAGCGCTCGCTCGAGCGCCCCGGGTAGCGGTCTGCTCACACGCT?????????????????TTTATGCTGCTAATACATACCTCGACACGGTAACGTTAATGGTCACTCTTATTGATCGTT?ATCCTACTTTTATAACAATCGGTTCCGACCGCAGGGGAAGAACCTTAGATCAAACAATCGGTCCCTTTGCTGGCTTTGGGTTCAGCTCAGCCGGAGTATTTCAGTTCCCCTCTTCGATGGTACGTGATATCCTACCACGCTAAGGGCCAAAACTACGCCCTGGCAGGAAAACATACGGGTCTCTTTCGTCCGTATTAACTTTTCCTTTAATCTTAGTTAGACTGTCTGGGGTCCAGGCGGCGTCCGCCGCGCGGGTACTGCGTCCTGACCTCCTGCCGGTTCCGCGGAGCTCGCC?GTTCTGGGTGGCCGGAACGTAATTATCAAAAGGCGCGCCTGAATAATGGTGGGTGCCTCGGTTCTAGTTGTTTTTCGGAACCGAGGTGAGGGAGGACGGTCGTTACGGCTTTGTCGCCTAGAATACAAATCGAACTTTAAAAACACGTGCACGATGATTACGATCCCCGGTTTGTTTCATGACTCGGCGGCAGCTTCCGACAAATCTTGACCATATTGATTAGAATCCCCCCGCTTATTGGTGGTGCTCTAGCATATCCGCGTGGCGCGCGCGACAAGTGCGTTTCACAGTTCCGCCAAGGAATGCGTTGTTGCTGGCGCGAGCTCGTCCGTTACCTCCTTCTATTGGGCTTCTCCCATGGACAGGGATAGTCCTTTTAGCGGTTTGTGGCCCTCGTTGGACCCGCGGGGGCAACCGTCGCGGTGGCGAGGACGGCCGCTTGACGGCTA????????????????????CACTTCCCCTAGGGGCTGCTGAGACACCGAAAGCTTCCCGCCAGTAACAAATAAATAATCTTTAAGAAAGCTTAAAATTATTGCAGAGCGTTGGCTTCATTCTCAATAGATAAAATACATAACCTAAAAAGAATTAGTGTGACTAAGGCATCAAAAACTTCCTTTTATTATAGAATTTTCCTGCCTGATCAAAATAAGCCACAAGCTATCTTTCTTAAGCCAATGTCAGAAAGGAGGCGCAGGGGCCCCTAGT????????CTCCACAGCCTCAGCTCACGCGGATCCGACCGCCCCCGCCCGGACGGCGGGAACGTGATAGGACGGCCTCTGGCGCGCGACGCGAGCGTCCAGTCCCCCTGTCGGGGTTACAGAATCGTGACGCCCTCGCGT?CCGTGTCTTGTGTCCTCGCGTGGAGCAGCGTAACATCTGCGGACACTCGGATTAACACCGTTCAGAGGTGGATGGGACGCAAAGTCGGCCCGTGGATGCCGGTCGTCGT??CGTCTCC??GGATCCTCGGGG??GGCGGCGGGTGCCCTTCCGCGGGGAGCCCACGACGGTTTCGGCGGTCTAAGCCCGAGAGAAGGATCGTCCGCCACGGAGTTATAGCTCTCGTGGCGTTGGCCCGCCGGTGGACCAGACAGTTGCCCGCCGGGCGCGTCCCGCCACTCGGACGTTCGACTGCAAGACTGCTCATGTTCCGACCGCGCG?GGATCCGGTCGCTCAGGGTCTGTGGCGTCGGTCGGCCGCCTATCAGGCTAAATTGCGCACGAGTCGCACTAAGCTCGGAACATGAGTGAAGGTCCCCCCTGTGGGCCCAGTAGTCCCGCGCTCGGGCCTACGCCGCTCGTCCGCCGTCGGTGGGGCGGACAACGTACACTTGGGTACCTATTCATAAGCTAGACTAGACAACTTGGTTGACTCCTGCGCTCGGAGAGTTTCGTACCTGTCGAACACATTAGGTCCGTGTCGATTATCGGGCGTTCGAGTGCAGCCCTACTCCTACCACGAGGATCTCATAATAATTTGTGAATACAACGCCGATCCGTGTCACTAGGGTCGGGTAGGACGTCGCGCAACGGGGCGACTCGCCGACGGGAGGCCCGTGGAACCGGCCTTGACCAGTCGGGGCGCACGGTCATACGGGTATGTTGAACGCAGATAGCGACGCGTTCCGAAGCGGGGCAATGTCGTGCCTGCA?TCTATATCGTATCGACCGGACACGGAGATGGC?CCTCCGGGTCTAGTGTACAACGAGTGAGAGCTCGGCGGGACCCCGGACTTGAGACGGACTCTGTCGGTTTGCCGATGACGTTGTTTCCTACCGCCCCCCGGGCGTGCGCTCTCGTCGGCTAATCTCACGACGGTGTGATTTCGTGCCGGGCTGGACCATCACTGATAGATTCTCATCGCGAGGCTGTCGTCTGGGGTACGAGCGTCTTGGAACCGGCAGGGGCTGGCGAGGCC??GTGGTCGACTCGCCGTCGCCGGGACCTGTCCGTGGAGTGCTCACTGGCGGCGCTCGCTCGCGTCTCTTCGGCCGGGTCTAAAGCCGATTTAACAAATGCTGCGCAGCTGGTGTGATTAAAAATCGGATCTAACGCGGAATTAGCTATCTGCCGACTCTTAGAGAATGAAAACGTGAGCAGTTTTTTACGTTTCTTATTTAAAAGCGGAAAGCGGCTACGGCCAGACCTGGAACGCGCCGGTCGCCGGTGCGCGTCCCTCTAAACTGTCAGCGGATAGAGCTTGAAGCAGGCGCTACACATCACTTTTTCGAAGTGTTAACTATTTATTAAGGATTATAACCTTTAATCGTCGTTAATCCCTCCCGTACATTCATTCTTGCTATAACAATTGAATTGGGATTGAC
