# Supplementary material for: Phylogeny of Annelida (Lophotrochozoa): total-evidence analysis of morphology and six genes
Source: BMC Evol Biol. 2009 Aug 6;9:189. doi: 10.1186/1471-2148-9-189 (PMC2732625; doi:10.1186/1471-2148-9-189)
Supplement: Additional file 4 — List of taxa used in the analyses. The data provided include a list of taxa used in the analyses with GenBank accession numbers. [file 1471-2148-9-189-S4.doc]

**Additional File 4**

**List of taxa used in the analyses with GenBank accession numbers**

The terminals used in the phylogenetic analyses are indicated by ‘>’.

-----------------------------------------------------------------------------------------------

**18S rRNA 28S rRNA EF1α 16S rRNA COI H3**

-----------------------------------------------------------------------------------------------

**Outgroups**

-----------------------------------------------------------------------------------------------

**> Brachiopoda**

***Terebratalia*** AF025945 AF342802 DQ813416 *** AB026508 ***

***Terebratulina*** *** *** *** *** ***DQ779768

**Mollusca**

**> Bivalvia**

***Nuculana*** *** *** *** DQ280030 *** ***

***Yoldia*** AF120528 AY145424 DQ813417 *** AF120642 ***

**> Gastropoda**

***Ilyanassa*** AY145379 AY145411 DQ813414 NC_007781 IOU86322 ***

***Nassarius*** *** *** *** *** ***AF033702

**> Polyplacophora**

***Chaetopleura*** AY145370 AY145398 U90062 *** *** ***

***Katharina*** *** *** *** AY377604 EF201385 AY377754

***-----------------------------****------------------------------------------------------------------*

**Possible annelid subtaxa**

-----------------------------------------------------------------------------------------------

**Echiurida**

**> Echiuroinea**

***Arhynchite*** AY210441 AY210455 *** *** *** ***

***Bonellia*** *** *** *** *** *** AF185263

***Listriolobus*** *** *** DQ813371 *** -- ***

**> *Urechis*** AF119076 AF519268 DQ813410 AF315059 NC_006379 X58895

**> Myzostomida**

***Myzostoma*** AF260584 AY210462 AF260590 DQ238178 DQ238199 AY218149

**Sipunculida**

**> *Phascolopsis*** AF342796 AF342795 AF063421 AF37433 DQ300134 AF519297

**> *Phascolosoma*** X79874 DQ300046 -*** EF521184 EF521190 DQ300087

***-----------------------------****------------------------------------------------------------------*

**Annelid subtaxa**

-----------------------------------------------------------------------------------------------

**> *Aberranta*** AY834760 *** *** *** *** ***

**> Acoetidae**

***Panthalis*** AY839572 *** *** ********* AY839584 ***

**> Acrocirridae**

***Macrochaeta*** DQ779658 DQ779696 *** *** *** DQ779741

**> Aeolosomatidae**

***Aeolosoma*** Z83748 DQ790019 DQ813345 DQ779600 AF054188 ***

**> Alciopidae**

***Alciopina*** DQ790073 DQ790021 DQ813347 *** *** ***

**> Alvinellidae**

***Paralvinella*** DQ790089 DQ790051 DQ813385 *** *** ***

**> Ampharetidae**

***Ampharete*** *** *** ***DQ779601 *** ***

***Auchenoplax*** DQ790077 DQ790026 DQ813352 *** *** ***

***Isolda*** *** *** *** ***AF342677 AF342699

**> Amphinomidae**

***Eurythoe*** *** *** *** *** ***AF185252

***Paramphinome*** AY838856 AY838865 DQ813383 DQ779629 AY838875 ***

**> Aphroditidae**

***Aphrodita*** AY894295 DQ790024 DQ813350 *** AY839578 ***

**> *Apistobranchus*** AF448150 DQ779675 ***DQ779603 *** ***

**> Arenicolidae**

***Abarenicola*** ***DQ790025 *** *** *** ***

***Arenicola*** DQ790076 *** DQ813351 AF108108 AY366522 DQ779718

**> Capitellidae**

***Notomastus*** DQ790084 DQ790044 DQ813379 AY340469 *** DQ779747

**> Chaetopteridae**

***Chaetopterus*** U67324 AY145399 *** DQ779607 AM503094 U96764

***Spiochaetopterus*** *** ***DQ813400*** *** ***

**> Chrysopetalidae**

***Dysponetus*** AY839568 DQ442599 *** DQ442570 AF221568 ***

**> Cirratulidae**

***Cirratulus*** AY708536 DQ790029 *** DQ779609 AF342672 DQ779724

***Cirriformia*** *** ***DQ813355 *** *** ***

**Clitellata**

**> *Capilloventer*** AY365455 AY340384 ***AY340448 *** ***

**> Lumbriculida + Hirudine**a

***Hirudo*** *** *** *** *** *** DQ779738

***Lumbriculus*** AY040693 DQ790040 AF063422 AY885578 AY519464 ***

**> *Lumbricus*** AJ272183 DQ790041 DQ813372 DQ388665 DQ092908 AF185262

**> *Stylaria*** U95946 DQ790065 DQ813403 DQ459947 AF534860 ***

**> Cossuridae**

***Cossura*** DQ779646 DQ779684 *** DQ779610 DQ779610 DQ779726

**> Ctenodrilidae**

***Ctenodrilus*** AF508119 AY364864 *** AY340452 AY340452 DQ779727

**> Dinophilidae**

***Dinophilus*** AF412805 *** *** AF380116 *** ***

***Trilobodrilus*** *** AY894292 *** *** AY598743 ***

**> Dorvilleidae**

***Ophryotrocha*** *** *** *** *** EF464548 ***

***Parougia*** AF412798 DQ790053 DQ813387 AY838841 *** ***

***Protodorvillea*** *** *** *** *** ***DQ779759

**> Eunicidae**

***Eunice*** AF412791 AY732229 DQ813362 DQ317917 AY838870 DQ779731

**> *Euphrosine*** DQ779649 DQ779687 *** DQ779613 *** DQ779732

**> Fauveliopsidae**

***Fauveliopsis*** AY708537 DQ790034 DQ813364 AY340456 *** AF185243

**> Flabelligeridae**

***Diplocirrus*** AY708534 DQ790031 DQ813358 *** *** ***

***Flabelligera*** *** *** *** DQ779614 *** ***

**> Glyceridae**

***Glycera*** AY995208 AY995207 DQ813366 DQ779615 AY995209 AF185236

**> Goniadidae**

***Goniada*** DQ790080 DQ790037 DQ813368 DQ779616 *** ***

**> Hesionidae**

***Neopodarke*** *** *** DQ813375 *** DQ442567 ***

***Ophiodromus*** DQ790086 DQ790046 *** DQ442579 *** ***

**> Histriobdellidae**

***Histriobdella*** AY527053 *** *** *** *** ***

**> *Hrabeiella*** AJ310501 AY364867 *** *** AY364854 ***

**> Lumbrineridae**

***Lumbrineris*** AY525623 AY366512 DQ813373 DQ779621 AY366520 DQ779740

**> *Magelona*** AY611454 AY611441 *** DQ779622 *** ABG79399

**> Maldanidae**

***Clymenella*** *** DQ790030 DQ813356 *** NC_006321 ***

***Clymenura*** AF448152 *** *** AY340449 *** DQ779725

**> Nephtyidae**

***Nephtys*** DQ790082 DQ790042 DQ813376 *** *** AAF02742

**> Nereididae**

***Ceratonereis*** *** *** *** *** AAS99584 AAF02743

***Nereis*** DQ790083 DQ790043 DQ813377 AY340470 *** ***

**> Nerillidae**

***Paranerilla*** AY859539 DQ279948 *** DQ280023 *** ***

**> Oenonidae**

***Drilonereis*** AY838847 AY838860 DQ813359 AY838828 AY838869 ***

**> Onuphidae**

***Diopatra*** AY838845 AY838858 DQ813357 AY838826 AY838867 ***

**> Opheliidae**

***Ophelia*** *** *** *** ***AY364856 ***

***Ophelina*** DQ790085 DQ790045 AB003708 AY340471 *** DQ779749

**> Orbiniidae**

***Orbinia*** DQ790087 DQ790048 DQ813381 AY532334 *** ***

***Phylo*** *** *** *** *** AY583703 AF185260

**> Oweniidae**

***Myriochele*** *** *** *** AY340468 *** AF185234

***Owenia*** AF448160 DQ790049 DQ813382 *** AY428839 ***

**> *Paralacydonia*** DQ790088 DQ790050 DQ813384 DQ779628 *** DQ779751

**> Paraonidae**

***Aricidea*** ***DQ790052 *** *** *** ***

***Cirrophorus*** *** *** ***AF185234 *** ***

***Paraonis*** DQ790090 *** DQ813386 *** *** ***

**> *Parapodrilus*** AF412800 *** *** *** *** ***

**> Parergodrilidae**

***Stygocapitella*** AF412810 AY366516 *** *** AY364852 ***

**> Pectinariidae**

***Pectinaria*** DQ790091 DQ790054 DQ813388 DQ319865 DQ209258 DQ508962

**> Pholoidae**

***Pholoe*** AY176301 *** *** *** AY839585 ***

**> Phyllodocidae**

***Eteone*** *** *** *** DQ779612 *** DQ779730

***Phyllodoce*** DQ790092 DQ790055 DQ813389 *** AY583697 ***

**> Pilargidae**

***Ancistrosyllis*** DQ790075 DQ790023 DQ813349 *** *** ***

***Sigambra*** *** *** *** AY340481 AF221574 ***

**> Pisionidae**

***Pisione*** AY525628 *** *** *** AF221575 ***

**> *Poecilochaetus*** AY569652 DQ779705 *** DQ779630 *** DQ779754

**> *Poeobius*** AY708526 DQ790058 DQ813392 DQ779631 *** DQ779755

**Pogonophora**

**> Frenulata**

***Galathealinum*** *** *** *** ***GBU74066 ***

***Siboglinum*** X79876 DQ790061 DQ813398 AF315039 *** DQ779765

**> *Osedax*** AY577885 *** *** AY586458 AB259569 ***

**> *Sclerolinum*** AF315061 *** *** AF315046 *** ***

**> Vestimentifera**

***Lamellibrachia*** *** *** *** *** ***AF185235

***Riftia*** AF168739 Z21534 DQ813394 AF315049 AY645989 ***

**> Polygordiidae**

***Polygordius*** AF412809 DQ779707 *** DQ779633 *** DQ779757

**> Polynoidae**

***Lepidonotus*** AY894301 DQ790039 DQ813370 DQ779620 AY894317 DQ779739

**> *Potamodrilus*** AY527056 *** *** *** AY598737 ***

**> *Protodriloides*** AY527058 *** *** ***AY366521 ***

**> Protodrilidae**

***Protodrilus*** AJ310506 AY340411 *** AY340474 *** DQ779760

**> *Questa*** AF209464 AY340413 *** AY340476 *** ***

**> Sabellariidae**

***Gunnarea*** *** *** *** ***DQ209263 ***

***Sabellaria*** AY732223 AY732226 DQ813395 AY340479 *** DQ779763

**> Sabellidae**

***Eudistylia***  *** *** DQ813361 *** *** ***

***Sabella*** *** *** *** AY340482 AY436349 DQ779762

***Schizobranchia*** AY732222 AY732225 *** *** *** ***

**> *Saccocirrus*** AF412808 AY340415 *** AY340478 *** ***

**> Scalibregmatidae**

***Scalibregma*** DQ790093 DQ790060 DQ813397 AY532331 *** DQ779764

**> Serpulidae**

***Protula*** *** *** *** *** ***DQ779761

***Salmacina*** *** ***DQ813396 *** *** ***

***Serpula*** AY732224 AY732227 *** *** *** ***

**> Sigalionidae**

***Sigalion*** *** *** *** ***AY583699 AF185248

***Sthenalanella*** AY894306 DQ790064 DQ813402 *** *** ***

**> Sphaerodoridae**

***Sphaerodoropsis*** EF123871 *** *** *** *** ***

**> Spionidae**

***Marenzelleria*** *** *** *** ***EF137727 ***

***Polydora*** U50971 DQ790059 DQ813393 DQ779632 *** DQ779756

**> *Sternaspis*** AY532329 DQ790063 DQ813401 AY532329 *** DQ779766

**> Syllidae**

***Eusyllis*** *** *** *** ***EF123749 ***

***Exogone*** AF474290 DQ790033 DQ813363 *** *** ***

***Proceraea*** *** *** *** AF474265 *** ***

**> Terebellidae**

***Lanice*** *** *** *** AY340466 *** ***

***Pista*** AY611461 DQ790057 DQ813391 *** EU239688 AF185242

**> Tomopteridae**

***Tomopteris*** DQ790095 DQ790067 DQ813405 *** *** ***

**> Trichobranchidae**

***Artacamella*** *** *** *** *** ***DQ779720

***Terebellides*** DQ790094 DQ790066 DQ813404 AY577884 DQ209261 ***

**> *Trochochaeta*** DQ790097 DQ790070 DQ813408 *** *** ***

***-----------------------------------------------------------------------------------------------***
